# Supplementary material for: Construction 7-membered ring via Ni–Al bimetal-enabled C–H cyclization for synthesis of tricyclic imidazoles
Source: Nat Commun. 2021 May 24;12:3070. doi: 10.1038/s41467-021-23371-x (PMC8144396; doi:10.1038/s41467-021-23371-x)
Supplement: Supplementary file 1 — Supplementary Information [file 41467_2021_23371_MOESM1_ESM.pdf]

Supplementary Information

Construction of Seven-Membered Ring via Ni–Al Bimetal-Enabled C–H Cyclization for Synthesis of Tricyclic Imidazoles

Li et al.

Supplementary Methods

General Information

Unless stated otherwise, all reactions were conducted under N_2 atmosphere in glove box. All solvents were received from commercial sources without further purification. Commercially available reagents were used as received. Non-commercially available substrates were synthesized following reported protocols. Melting points were measured on X-4B microscope melting point apparatus and uncorrected. Thin-layer chromatography (TLC) was performed by UV absorbance (254 nm). 200-300 mesh silica gel was used for column chromatography separation. NMR spectra were recorded on Bruker AV 400 spectrometer at 400 MHz (^{1}H NMR), 100 MHz (^{13}C NMR), 376 MHz (^{19}F NMR), 162 MHz (^{31}P NMR) using $CDCl_3$ as solvent. Proton and carbon chemical shifts are reported relative to the solvent used as an internal reference ($CDCl_3$: δ_H = 7.26 ppm; δ_C = 77.16 ppm). All coupling constants (*J* values) were reported in Hertz (Hz). Multiplicities are reported as follows: singlet (s), doublet (d), doublet of doublets (dd), triplet (t), triplet of doublets (td), quartet (q), and multiplet (m). High resolution mass spectra (HRMS) were recorded on an Agilent 6520 Q-TOF LC/MS with Electron Spray Ionization (ESI) resource. Chiral high-performance liquid chromatography (HPLC) analysis was performed using an Agilent 1260 with commercial ChiralPak 4.6 × 250 mm columns. Optical rotations were determined by a Rudolph Autopol VI polarimeter. Single crystal X-ray diffraction data were collected on Rigaku Saturn70 diffractometer at with Mo-Kα radiation.

Supplementary Note 1

Substrate Preparation

Method A:

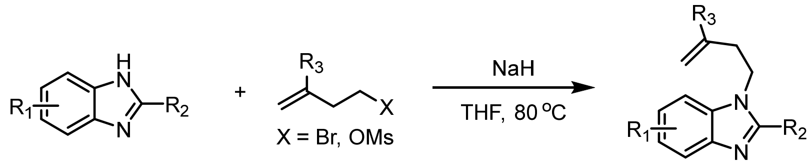

To an oven-dried 3-necked flask was added 2-(trifluoromethyl)-1H-benzo[*d*]imidazole (0.5 M) in THF. The solution was cooled to 0 °C and NaH (2 equiv, 60% in mineral oil) was slowly added. The resulting mixture was warmed to room temperature and stirred for 10 min before homoallyl halide or methanesulfonate (1.2 equiv) was added. The resulting solution was heated to reflux and monitored by TLC until the total disappearance of benzoimidazole. After that, the reaction was quenched with H_2O and concentrated in *vacuo*. The residue was dissolved in CH_2Cl_2 and washed with H_2O. The aqueous layer was separated and back-extracted with CH_2Cl_2. The combined organic extracts were dried over anhydrous Na_2SO_4 and concentrated in *vacuo*. The crude product was purified by flash chromatography on silica gel, eluting with EtOAc/n-hexane.

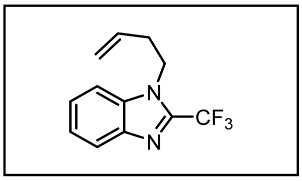

1-(But-3-en-1-yl)-2-(trifluoromethyl)-1*H*-benzo[*d*]imidazole (1a)[1]

Yellow oil (95% yield). **^{1}H NMR** (400 MHz, $CDCl_3$) δ 7.88 (d, *J* = 8.0 Hz, 1H), 7.51 – 7.32 (m, 3H), 5.85 – 5.75 (m, 1H), 5.10 (d, *J* = 13.2 Hz, 2H), 4.36 (t, *J* = 7.6 Hz, 2H), 2.62 (q, *J* = 7.2 Hz, 2H). **^{13}C NMR** (100 MHz, $CDCl_3$) δ 141.3, 140.5 (q, $^2J_{C-F}$ = 38.2 Hz), 135.4, 133.0, 125.4, 123.7, 121.8, 119.3 (q, $^1J_{C-F}$ = 269.6 Hz), 118.6, 110.7, 44.6, 34.2. **^{19}F NMR** (376 MHz, $CDCl_3$) δ -57.5.

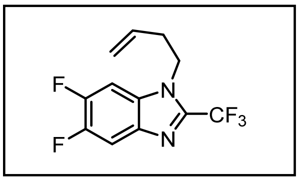

1-(But-3-en-1-yl)-5,6-difluoro-2-(trifluoromethyl)-1H-benzo[*d*]imidazole (1h)

White solid (95% yield). m.p. 91-93 °C. **^{1}H NMR** (400 MHz, $CDCl_3$) δ 7.64 (dd, *J* = 10.0, 7.6 Hz, 1H), 7.26 (dd, *J* = 9.6, 7.6 Hz, 1H), 5.85 – 5.72 (m, 1H), 5.21 – 5.04 (m, 2H), 4.33 (t, *J* = 7.6 Hz, 2H), 2.61 (q, *J* = 7.6 Hz, 2H). **^{13}C NMR** (100 MHz, $CDCl_3$) δ 150.1 (dd, $^1J_{C-F}$ = 246.5,16.0 Hz), 148.9 (dd, $^1J_{C-F}$ = 243.1,15.1 Hz), 142.1 (q, $^2J_{C-F}$ = 38.8 Hz), 136.3 (d, $^3J_{C-F}$ = 10.3 Hz), 132.6, 131.0 (d, $^3J_{C-F}$ = 10.4 Hz), 119.1, 118.8 (q, $^1J_{C-F}$ = 269.9 Hz), 109.0 (d, $^2J_{C-F}$ = 19.5 Hz), 98.8 (d, $^2J_{C-F}$ = 23.2 Hz), 45.1, 34.1. **^{19}F NMR** (376 MHz, $CDCl_3$) δ -61.9, -136.3, -140.3. **HRMS (ESI)** *m/z:* $[M+H]^+$ Calcd. for $C_{12}H_{10}F_5N_2$ 277.0759; Found: 277.0761.

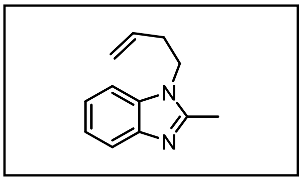

1-(But-3-en-1-yl)-2-methyl-1*H*-benzo[*d*]imidazole (1k)

Yellow oil (82% yield). **^{1}H NMR** (400 MHz, $CDCl_3$) δ 7.72 – 7.65 (m, 1H), 7.32 – 7.26 (m, 1H), 7.24 – 7.17 (m, 2H), 5.82 – 5.71 (m, 1H), 5.14 – 5.01 (m, 2H), 4.15 (t, *J* = 7.2 Hz, 2H), 2.59 (s, 3H), 2.53 (q, *J* = 7.2 Hz, 2H). **^{13}C NMR** (100 MHz, $CDCl_3$) δ 151.4, 142.6, 134.8, 133.6, 121.9, 121.7, 119.0, 118.2, 109.1, 43.3, 33.8, 14.0. **HRMS (ESI)** *m/z:* $[M+H]^+$ Calcd. for $C_{12}H_{15}N_2$ 187.1230; Found 187.1230.

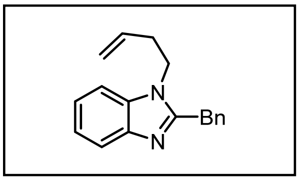

2-Benzyl-1-(but-3-en-1-yl)-1*H*-benzo[*d*]imidazole (1l)

White solid (83% yield). m.p. 77-78 °C. **^{1}H NMR** (400 MHz, $CDCl_3$) δ 7.84 – 7.70 (m, 1H), 7.35 – 7.16 (m, 8H), 5.70 – 5.58 (m, 1H), 5.12 – 4.86 (m, 2H), 4.32 (s, 2H), 4.03 (t, *J* = 7.6 Hz, 2H), 2.22 (q, *J* = 7.2 Hz, 2H). **^{13}C NMR** (100 MHz, $CDCl_3$) δ 153.1, 142.8, 136.5, 135.3, 133.7, 128.9, 128.6, 127.1, 122.4, 122.0, 119.7, 118.1, 109.5, 43.6, 34.8, 33.7. **HRMS (ESI)** *m/z:* $[M+H]^+$ Calcd. for $C_{18}H_{19}N_2$ 263.1543; Found 263.1545.

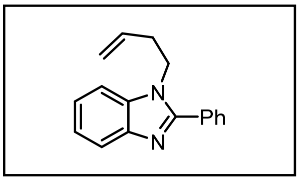

1-(But-3-en-1-yl)-2-phenyl-1*H*-benzo[*d*]imidazole (1m)

White solid (88% yield). m.p. 60-61 °C. **^{1}H NMR** (400 MHz, $CDCl_3$) *δ* 7.87 – 7.80 (m, 1H), 7.74 – 7.68 (m, 2H), 7.56 – 7.48 (m, 3H), 7.46 – 7.40 (m, 1H), 7.36 – 7.28 (m, 2H), 5.72 – 5.60 (m, 1H), 5.06 – 4.85 (m, 2H), 4.30 (t, *J* = 7.6 Hz, 2H), 2.55 (q, *J* = 7.2 Hz, 2H). **^{13}C NMR** (100 MHz, $CDCl_3$) *δ* 153.7, 143.1, 135.4, 133.4, 130.6, 129.7, 129.3, 128.7, 122.7, 122.4, 120.0, 117.9, 110.0, 44.0, 33.9. **HRMS (ESI)** *m/z:* $[M+H]^+$ Calcd. for $C_{17}H_{17}N_2$: 249.1386; Found 249.1388.

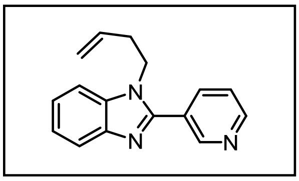

1-(But-3-en-1-yl)-2-(pyridin-3-yl)-1*H*-benzo[d]imidazole (1n)

Yellow oil (66% yield). **^{1}H NMR** (400 MHz, $CDCl_3$) *δ* 8.98 (s, 1H), 8.77 (d, *J* = 3.6 Hz, 1H), 8.09 (d, *J* = 7.6 Hz, 1H), 7.85 (d, *J* = 7.6 Hz, 1H), 7.54 – 7.44 (m, 2H), 7.40 – 7.30 (m, 2H), 5.70 – 5.58 (m, 1H), 5.05 – 4.95 (m, 2H), 4.33 (t, *J* = 7.6 Hz, 2H), 2.58 (q, *J* = 7.2 Hz, 2H). **^{13}C NMR** (100 MHz, $CDCl_3$) *δ* 150.82, 150.77, 149.9, 143.4, 137.2, 135.6, 133.2, 127.2, 123.7, 123.4, 122.9, 120.4, 118.6, 110.4, 44.4, 34.1. **HRMS (ESI)** *m/z:* $[M+H]^+$ Calcd. for $C_{13}H_{11}F_6N_2^+$:250.1339; Found 250.1337.

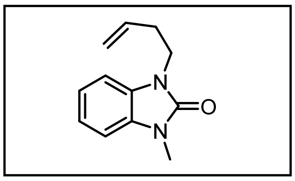

1-(But-3-en-1-yl)-3-methyl-1,3-dihydro-2*H*-benzo[*d*]imidazol-2-one (1s)

Yellow oil (88% yield). **^{1}H NMR** (400 MHz, $CDCl_3$) *δ* 7.12 – 7.06 (m, 2H), 7.02 – 6.92 (m, 2H), 5.89 – 5.78 (m, 1H), 5.16 – 4.97 (m, 2H), 3.95 (t, *J* = 7.6 Hz, 2H), 3.42 (s, 3H), 2.51 (q, *J* = 7.2 Hz, 2H). **^{13}C NMR** (100 MHz, $CDCl_3$) *δ* 154.4, 134.6, 130.1, 129.4, 121.2, 121.2, 117.6, 107.7, 107.5, 40.7, 32.9, 27.2. **HRMS (ESI)** *m/z:* $[M+H]^+$ Calcd. for $C_{12}H_{14}N_2NaO$ 225.0998; Found 225.0994.

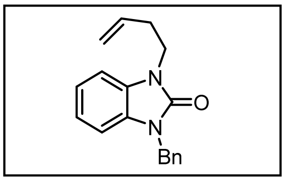

1-Benzyl-3-(but-3-en-1-yl)-1,3-dihydro-2*H*-benzo[*d*]imidazol-2-one (1t)

Yellow oil (90% yield). **^{1}H NMR** (400 MHz, $CDCl_3$) *δ* 7.43 – 7.25 (m, 5H), 7.16 – 6.94 (m, 3H), 6.86 (d, *J* = 7.6 Hz, 1H), 5.91 – 5.78 (m, 1H), 5.21 – 4.76 (m, 2H), 3.97 (t, *J* = 7.6 Hz, 2H), 2.53 (dt, *J* = 15.4, 7.5 Hz, 1H). **^{13}C NMR** (100 MHz, $CDCl_3$) *δ* 154.5, 136.5, 134.6, 128.8, 127.7, 127.5, 121.4, 121.2, 117.7, 108.4, 107.6, 44.9, 40.8, 32.9. **HRMS (ESI)** *m/z:* $[M+Na]^+$ Calcd. for $C_{18}H_{18}N_2NaO$ 301.1311; Found 301.1315.

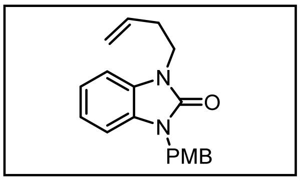

1-(But-3-en-1-yl)-3-(4-methoxybenzyl)-1,3-dihydro-2*H*-benzo[*d*]imidazol-2-one (1u)

Yellow oil (86% yield). ^{1}**H NMR** (400 MHz, $CDCl_3$) δ 7.25 (s, 1H), 7.24 (s, 1H), 7.09 – 7.03 (m, 1H), 7.02 – 6.94 (m, 2H), 6.89 – 6.87 (m, 1H), 6.85 – 6.80 (m, 2H), 5.96 – 5.74 (m, 1H), 5.11 – 5.02 (m, 2H), 5.00 (s, 2H), 3.98 (t, *J* = 7.2 Hz, 2H), 3.76 (s, 3H), 2.53 (q, *J* = 7.2 Hz, 2H). ^{13}**C NMR** (100 MHz, $CDCl_3$) δ = 159.2, 154.4, 134.6, 129.5, 129.3, 129.0, 128.6, 121.3, 121.2, 117.6, 114.2, 108.4, 107.6, 55.3, 44.4, 40.7, 32.8. **HRMS (ESI)** *m/z:* $[M+Na]^+$ Calcd. for $C_{19}H_{20}N_2NaO_2$ 331.1417; Found 331.1421.

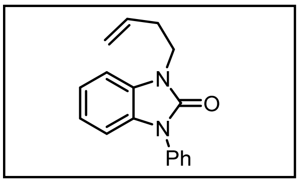

1-(But-3-en-1-yl)-3-phenyl-1,3-dihydro-2*H*-benzo[*d*]imidazol-2-one (1v)

Yellow oil (90% yield). ^{1}**H NMR** (400 MHz, $CDCl_3$) δ = 7.69 – 7.46 (m, 4H), 7.42 – 7.36 (m, 1H), 7.21 – 7.01 (m, 4H), 5.91 – 5.78 (m, 1H), 5.21 – 5.02 (m, 2H), 4.11 – 3.93 (m, 2H), 2.51 (q, *J* = 7.2 Hz, 2H). ^{13}**C NMR** (100 MHz, $CDCl_3$) δ = 154.5, 134.8, 134.6, 129.6, 127.7, 126.1, 122.0, 121.4, 117.7, 108.9, 108.0, 40.86, 32.78. **HRMS (ESI)** *m/z:* $[M+Na]^+$ Calcd. for $C_{17}H_{16}N_2NaO$ 287.1155; Found 287.1158.

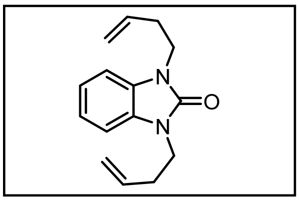

1,3-Di(but-3-en-1-yl)-1-methyl-1,3-dihydro-2*H*-1l4-benzo[*d*]imidazol-2-one (1w)

Yellow oil (80% yield). ^{1}**H NMR** (400 MHz, $CDCl_3$) δ 7.08 (dd, *J* = 5.6, 3.2 Hz, 2H), 6.99 (dd, *J* = 5.6, 3.2 Hz, 2H), 5.83 – 5.77 (m, 2H), 5.09 – 5.00 (m, 4H), 3.94 (t, *J* = 7.2 Hz, 4H), 2.50 (q, *J* = 7.2 Hz, 4H). ^{13}**C NMR** (100 MHz, $CDCl_3$) δ 154.1, 134.5, 129.4, 121.0, 117.5, 107.7, 40.6, 32.7. **HRMS (ESI)** *m/z:* $[M+Na]^+$ Calcd. for $C_{16}H_{21}N_2NaO$ 280.1546; Found 280.1551.

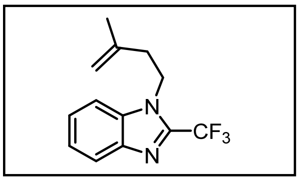

1-(3-Methylbut-3-en-1-yl)-2-(trifluoromethyl)-1*H*-benzo[*d*]imidazole (1a′)

Yellow oil (96% yield). ^{1}**H NMR** (400 MHz, $CDCl_3$) δ 7.88 (d, *J* = 8.0 Hz, 1H), 7.48 – 7.40 (m, 2H), 7.39 – 7.34 (m, 1H), 4.88 (s, 1H), 4.77 (s, 1H), 4.33 (t, *J* = 7.6 Hz, 2H), 2.61 (t, *J* = 7.6 Hz, 2H), 1.83 (s, 3H). ^{13}**CNMR** (100 MHz, $CDCl_3$) δ 141.1, 140.9, 140.2(q, $^{2}J_{C-F}$ = 38.2 Hz), 135.1, 125.3, 123.6, 119.2 (q, $^{1}J_{C-F}$ = 269.5 Hz), 121.6, 113.1, 110.4, 43.8, 37.7, 22.3. ^{19}**F NMR** (376 MHz, $CDCl_3$) δ -62.0. **HRMS (ESI)** *m/z:* $[M+H]^+$ Calcd. for $C_{13}H_{14}F_3N_2$ 255.1104; Found 255.1103.

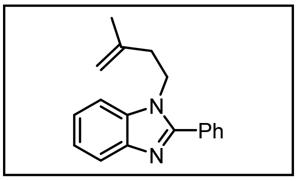

1-(3-Methylbut-3-en-1-yl)-2-phenyl-1*H*-benzo[*d*]imidazole (1r′)

White solid (83% yield), m.p. 89-90 °C°C. **^{1}H NMR** (400 MHz, $CDCl_3$) *δ* 7.89 – 7.78 (m, 1H), 7.75 – 7.66 (m, 2H), 7.57 – 7.48 (m, 3H), 7.46 – 7.40 (m, 1H), 7.34 – 7.28 (m, 2H), 4.77 – 4.68 (m, 1H), 4.57 (s, 1H), 4.33 (t, *J* = 7.6 Hz, 2H), 2.49 (t, *J* = 7.6 Hz, 2H), 1.61 (s, 3H). **^{13}C NMR** (100 MHz, $CDCl_3$) *δ* 153.9, 143.3, 141.3, 135.5, 130.7, 129.8, 129.5, 128.8, 122.8, 122.5, 120.2, 113.2, 110.1, 43.4, 37.69, 22.37. **HRMS (ESI)** *m/z:* $[M+H]^+$ Calcd. for $C_{18}H_{19}N_2$ 263.1543; Found 263.1545.

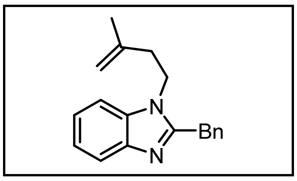

2-Benzyl-1-(3-methylbut-3-en-1-yl)-1*H*-benzo[*d*]imidazole(1s′)

White solid (87% yield), m.p. 85-86 °C. **^{1}H NMR** (400 MHz, $CDCl_3$) *δ* 7.85 – 7.69 (m, 1H), 7.43 – 7.14 (m, 8H), 4.79 (s, 1H), 4.59 (s, 1H), 4.32 (s, 2H), 4.18 – 3.96 (m, 2H), 2.22 – 2.00 (m, 2H), 1.68 (s, 3H). **^{13}C NMR** (100 MHz, $CDCl_3$) *δ* 153.9, 143.3, 141.3, 135.5, 130.7, 129.8, 129.5, 128.8, 122.8, 122.5, 120.2, 113.2, 110.1, 43.4, 37.7, 22.4. **HRMS (ESI)** *m/z:* $[M+H]^+$ Calcd. for $C_{19}H_{21}N_2$ 277.1699; Found 277.1703.

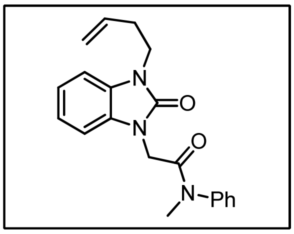

2-(3-(But-3-en-1-yl)-2-oxo-2,3-dihydro-1*H*-benzo[*d*]imidazol-1-yl)-*N*-methyl-*N*-phenylacetamide(1x)

White solid (85% yield). m.p. 148-149 °C. **^{1}H NMR** (400 MHz, $CDCl_3$) *δ* 7.49 (t, *J* = 7.6 Hz, 2H), 7.40 (t, *J* = 7.2 Hz, 1H), 7.33 (d, *J* = 7.6 Hz, 2H), 7.10 – 7.02 (m, 2H), 6.99 – 6.94 (m, 1H), 6.91 – 6.74 (m, 1H), 5.91 – 5.78 (m, 1H), 5.14 – 4.98 (m, 2H), 4.39 (s, 2H), 3.90 (t, *J* = 7.6 Hz, 2H), 3.30 (s, 3H), 2.53 (dt, *J* = 15.2, 7.6 Hz, 2H). **^{13}C NMR** (100 MHz, $CDCl_3$) *δ* 166.5, 154.2, 142.6, 134.6, 130.4, 129.8, 129.5, 128.7, 127.4, 121.5, 121.4, 117.6, 108.1, 107.9, 43.1, 40.9, 37.9, 32.8. **HRMS (ESI)** *m/z:* $[M+Na]^+$ Calcd. for $C_{20}H_{21}N_3NaO_2$ 358.1526; Found 358.1529

Method B:

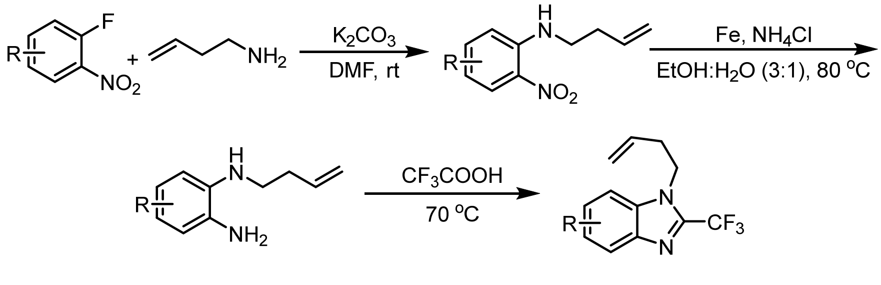

To a 25 mL round bottom flask were added 1-fluoro-4-methyl-2-nitro benzene (1.55 g, 10 mmol) in DMSO (6 mL) and but-3-en-1- amine (1.1 g, 15 mmol) in DMSO (6 mL). After stirring for 3 h at room temperature, the solution was added to H_2O and extracted with EA. The organic layers were combined, dried over anhydrous Na_2SO_4, and concentrated in *vacuo* to obtain the crude *N*-(but-3-en-1-yl)-4-methyl-2-nitroaniline, which was used in the next step without further purification.

To a suspension of *N*-(but-3-en-1-yl)-4-methyl-2-nitroaniline (2.0 g, 9.6 mmol) and iron powder (5.36 g, 96.0 mmol) in EtOH (90 mL) was added aq. HCl (0.6 M, 21 mL, 12.5 mmol), and the resulting suspension was refluxed for 2 h with vigorous stirring. The mixture was cooled to ambient temperature and filtered through a pad of Celite. The filtrate was diluted with EtOAc, washed with sat. aq. $NaHCO_3$, dried over anhydrous Na_2SO_4 and concentrated in *vacuo*, affording N^1-(but-3-en-1-yl)-4-methyl benzene- 1,2-diamine.

To a flame-dried 200 mL round bottom flask equipped with a stir bar was added N^1-(but-3-en-1-yl)-4-methyl benzene-1,2-diamine (1.69 g, 9.6 mmol) in CF_3COOH (10 mL). The mixture was heated to 70 °C until the reaction was complete. Then the solution was diluted with EA, washed with saturated aq. $NaHCO_3$, extracted with ethyl acetate. The organic phases were combined, dried over anhydrous Na_2SO_4 and concentrated in *vacuo*. The residue was purified via flash chromatography on silica gel, eluting with EtOAc/hexane (1:20).

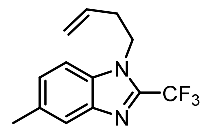

1-(But-3-en-1-yl)-5-methyl-2-(trifluoromethyl)-1*H*-benzo[*d*]imidazole (1b)

Yellow oil (73% yield). **^{1}H NMR** (400 MHz, $CDCl_3$) δ 7.64 (s, 1H), 7.33 (d, *J* = 8.4 Hz, 1H), 7.24(d, *J* = 7.2 Hz, 1H), 5.90 – 5.68 (m, 1H), 5.13 – 5.03 (m, 2H), 4.34 (t, *J* = 7.6 Hz, 2H), 2.62 (q, *J* = 8.8 Hz, 2H), 2.49 (s, 3H). **^{13}C NMR** (100 MHz, $CDCl_3$) δ 141.6, 140.4 (q, $^2J_{C-F}$ = 38.2 Hz), 133.6, 133.5, 133.1, 127.1, 121.2, 119.2 (q, $^1J_{C-F}$ = 269.5 Hz), 118.5, 110.2, 44.6, 34.2, 21.6. **^{19}F NMR** (376 MHz, $CDCl_3$) δ -57.4. **HRMS (ESI)** *m/z:* $[M+H]^+$ Calcd. for $C_{13}H_{14}F_3N_2$ 255.1104; Found 255.1105.

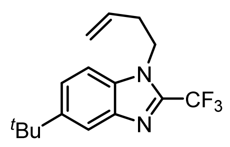

1-(But-3-en-1-yl)-5-(tert-butyl)-2-(trifluoromethyl)-1*H*-benzo[*d*]imidazole (1c)

Yellow oil (70% yield). **^{1}H NMR** (400 MHz, $CDCl_3$) δ 7.89 (s, 1H), 7.52 (d, *J* = 8.8 Hz, 1H), 7.39 (d, *J* = 8.8 Hz, 1H), 5.87 – 5.75 (m, 1H), 5.18 – 5.03 (m, 2H), 4.34 (t, *J* = 7.6 Hz, 2H), 2.62 (q, *J* = 8.8 Hz, 2H), 1.39 (s, 9H). **^{13}C NMR** (100 MHz, $CDCl_3$) δ 147.3, 141.3, 140.4 (q, $^2J_{C-F}$ = 38.2 Hz), 133.3, 133.1, 123.8, 119.3 (q, $^1J_{C-F}$ = 269.5 Hz), 118.5, 117.7, 110.0, 44.6, 34.9, 34.3, 31.7. **^{19}F NMR** (376 MHz, $CDCl_3$) δ -61.8. **HRMS (ESI)** *m/z:* $[M+H]^+$ Calcd. for $C_{16}H_{20}F_3N_2$ 297.1573; Found 297.1577

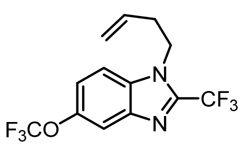

1-(But-3-en-1-yl)-5-(trifluoromethoxy)-2-(trifluoromethyl)-1*H*-benzo[d]imidazole (1d)

Orange solid (65% yield). m.p. 62-64 °C. **^{1}H NMR** (400 MHz, $CDCl_3$) δ 7.76 (s, 1H), 7.47 (d, *J* = 8.8 Hz, 1H), 7.34 (d, *J* = 8.8 Hz, 1H), 5.85 – 5.75 (m, 1H), 5.20 – 5.07 (m, 2H), 4.39 (t, *J* = 7.6 Hz, 2H), 2.63 (q, *J* = 8.8 Hz, 2H). **^{13}C NMR** (100 MHz, $CDCl_3$) δ 145.5, 142.3 (q, $^2J_{C-F}$ = 38.8 Hz), 141.3, 133.9, 132.7,

120.7 (q, $^{1}J_{C\text{-}F}$ = 255.3 Hz), 119.7, 119.0 (q, $^{1}J_{C\text{-}F}$ = 269.9 Hz), 118.9, 114.4, 111.6, 44.9, 34.2. **^{19}F NMR** (376 MHz, $CDCl_3$) δ -57.8, -54.0. **HRMS (ESI)** *m/z:* $[M+H]^+$ Calcd. for $C_{13}H_{11}F_6N_2O$ 325.0770; Found 325.0776.

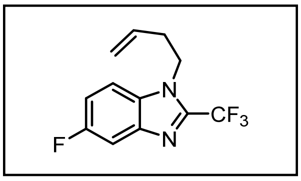

1-(But-3-en-1-yl)-5-fluoro-2-(trifluoromethyl)-1*H*-benzo[*d*]imidazole (1e)

Yellow oil (80% yield). **^{1}H NMR** (400 MHz, $CDCl_3$) δ 7.54 (dd, *J* = 9.6, 2.4 Hz, 1H), 7.40 (dd, *J* = 8.8, 4.4 Hz, 1H), 7.25– 7.17 (m, 1H), 5.87 – 5.72 (m, 1H), 5.16 – 5.04 (m, 2H), 4.37 (t, *J* = 7.6 Hz, 2H), 2.62 (q, *J* =7.2 Hz, 2H). **^{13}C NMR** (100 MHz, $CDCl_3$) δ 159.9 (d, $^{1}J_{C\text{-}F}$ = 240.2 Hz), 141.7 (q, $^{2}J_{C\text{-}F}$ = 38.3 Hz), 141.5, 132.7, 131.9, 119.0 (q, $^{1}J_{C\text{-}F}$ = 269.7 Hz), 118.8, 114.3 (d, $^{2}J_{C\text{-}F}$ = 26.7 Hz), 111.4 (d, $^{3}J_{C\text{-}F}$ = 10.2 Hz), 107.1 (d, $^{2}J_{C\text{-}F}$ = 24.1 Hz), 44.8, 34.2. **^{19}F NMR** (376 MHz, $CDCl_3$) δ -57.6, -114.1. **HRMS (ESI)** *m/z:* $[M+H]^+$ Calcd. for $C_{12}H_{11}F_4N_2$ 259.0853; Found 259.0854.

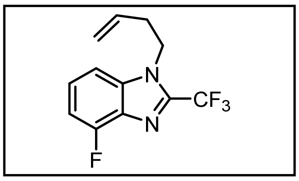

1-(But-3-en-1-yl)-4-fluoro-2-(trifluoromethyl)-1*H*-benzo[*d*]imidazole (1f)

Yellow oil (83% yield). **^{1}H NMR** (400 MHz, $CDCl_3$) δ 7.37 (dd, *J* = 12.4, 6.0 Hz, 1H), 7.29 – 7.23 (m, 1H), 7.05 (t, *J* = 9.6 Hz, 1H), 5.86 – 5.73 (m, 1H), 5.17 – 5.03 (m, 2H), 4.38 (t, *J* = 7.6 Hz, 2H), 2.62 (q, *J* = 7.2 Hz, 2H). **^{13}C NMR** (100 MHz, $CDCl_3$) δ 154.5 (d, $^{1}J_{C\text{-}F}$ = 255.9 Hz), 140.4 (q, $^{2}J_{C\text{-}F}$ = 38.9 Hz), 137.9 (d, $^{3}J_{C\text{-}F}$ = 7.0 Hz), 132.5, 130.1 (d, $^{2}J_{C\text{-}F}$ = 17.1 Hz), 125.7 (d, $^{3}J_{C\text{-}F}$ = 7.1 Hz), 118.8 (q, $^{1}J_{C\text{-}F}$ = 269.7 Hz), 118.3, 108.5 (d, $^{2}J_{C\text{-}F}$ = 17.0 Hz), 106.8 (d, $^{4}J_{C\text{-}F}$ = 4.3 Hz), 44.7, 33.8. **^{19}F NMR** (376 MHz, $CDCl_3$) δ -61.7, -125.6. **HRMS (ESI)** *m/z:* $[M+H]^+$ Calcd. for $C_{12}H_{11}F_4N_2$ 259.0853; Found 259.0854.

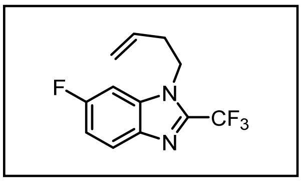

1-(But-3-en-1-yl)-6-fluoro-2-(trifluoromethyl)-1*H*-benzo[*d*]imidazole (1g)

Yellow oil (85% yield). **^{1}H NMR** (400 MHz, $CDCl_3$) δ 7.82 (dd, *J* = 9.6, 4.8 Hz, 1H), 7.20 – 7.06 (m, 2H), 5.87 – 5.72 (m, 1H), 5.21 – 5.04 (m, 2H), 4.33 (t, *J* = 7.6 Hz, 2H), 2.61 (q, *J* = 7.6 Hz, 2H). **^{13}C NMR** (100 MHz, $CDCl_3$) δ 161.1(d, $^{1}J_{C\text{-}F}$ = 242.4 Hz), 141.2 (q, $^{2}J_{C\text{-}F}$ = 38.5 Hz), 137.7, 135.7 (d, $^{3}J_{C\text{-}F}$ = 13.1 Hz), 132.8, 123.0 (d, $^{3}J_{C\text{-}F}$ = 10.4Hz), 119.0 (q, $^{1}J_{C\text{-}F}$ = 269.9Hz), 118.9, 112.8 (d, $^{2}J_{C\text{-}F}$ = 25.4 Hz), 97.2 (d, $^{2}J_{C\text{-}F}$ = 27.6 Hz), 44.8, 34.0. **^{19}F NMR** (376 MHz, $CDCl_3$) δ -69.2, -121.6. **HRMS (ESI)** *m/z:* $[M+H]^+$ Calcd. for $C_{12}H_{11}F_4N_2$ 259.0853; Found 259.0856.

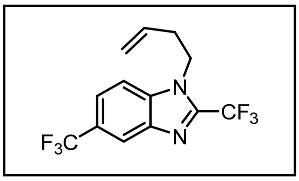

1-(But-3-en-1-yl)-2,5-bis(trifluoromethyl)-1*H*-benzo[*d*]imidazole (1i)

Yellow oil (77% yield). **^{1}H NMR** (400 MHz, $CDCl_3$) δ 8.17 (s, 1H), 7.69 (d, J = 8.8 Hz, 1H), 7.58 (d, J = 8.8 Hz, 1H), 5.81 – 5.57 (m, 1H), 5.16 – 5.04 (m, 2H), 4.42 (t, J = 7.6 Hz, 2H), 2.64 (q, J = 8.8 Hz, 2H). **^{13}C NMR** (100 MHz, $CDCl_3$) δ 142.5 (q, $^{2}J_{C\text{-}F}$ = 38.7 Hz), 140.6, 137.3, 132.6, 126.4 (q, $^{2}J_{C\text{-}F}$ = 32.5 Hz), 124.4 (q, $^{1}J_{C\text{-}F}$ = 270.3 Hz), 122.2 (q, $^{3}J_{C\text{-}F}$ = 3.4 Hz), 119.6 (q, $^{3}J_{C\text{-}F}$ = 4.2 Hz), 119.1, 119.0 (q, $^{1}J_{C\text{-}F}$ = 270.1 Hz), 111.6, 44.9, 34.2. **^{19}F NMR** (376 MHz, $CDCl_3$) δ -61.2, -62.2. **HRMS (ESI)** *m/z:* $[M+H]^+$ Calcd. for $C_{13}H_{11}F_6N_2^+$: 309.0821; Found 309.0822.

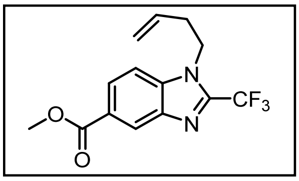

Methyl 1-(but-3-en-1-yl)-2-(trifluoromethyl)-1*H*-benzo[*d*]imidazole-5-carboxylate (1j)

White solid (72% yield). m.p. 88-89 °C. **^{1}H NMR** (400 MHz, $CDCl_3$) δ 8.59 (s, 1H), 8.15 (d, J = 8.8 Hz, 1H), 7.50 (d, J = 8.8Hz, 1H), 5.95 – 5.64 (m, 1H), 5.13– 5.06 (m, 2H), 4.40 (t, J = 7.6 Hz, 2H), 3.97 (s, 3H), 2.62 (q, J = 8.8 Hz, 2H). **^{13}C NMR** (100 MHz, $CDCl_3$) δ 166.9, 142.2 (q, $^{2}J_{C\text{-}F}$ = 38.6 Hz), 140.8, 138.3, 132.6, 126.5, 126.0, 124.1, 118.9 (q, $^{1}J_{C\text{-}F}$ = 270 Hz), 118.8 110.6, 52.3, 44.8, 34.1. **^{19}F NMR** (376 MHz, $CDCl_3$) δ -57.8. **HRMS (ESI)** *m/z:* $[M+H]^+$ Calcd. for $C_{14}H_{14}F_3N_2O_2$ 299.1002; Found 299.1007.

Method C:

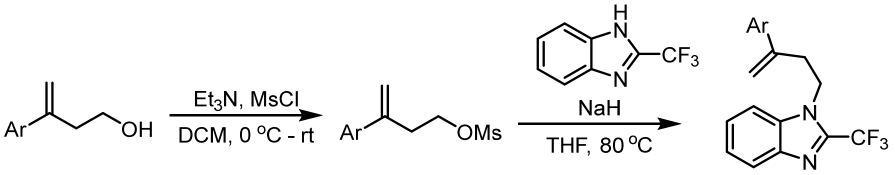

Synthesis of alcohol:

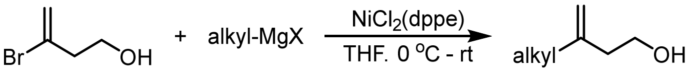

To a solution of 3-bromobut-3-en-1-ol[2] (7.0 mmol, 1.0 equiv) and $NiCl_2$(dppe) (350 μmol, 0.05 equiv) in 20 mL dry THF at 0 °C was added alkly magnesium bromide or alkly magnesium chlorine (usually 1M in THF, 17.5 mL, 2.5 equiv) dropwise. The reaction mixture was allowed to slowly warm to room temperature and stirred for 36 h. The resulting mixture was quenched with saturated aq. NH_4Cl (3 mL) and extracted with Et_2O (2 × 10 mL). The organic layers were combined and dried over anhydrous Na_2SO_4 and concentrated in *vacuo*. The crude residue was purified by flash column chromatography on silica gel (EtOAc/hexanes, 1:10 to 1:5) to afford product as colorless oil.

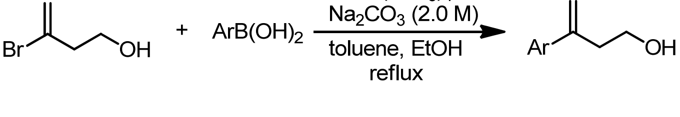

Following the literature protocols:[3,4] To a stirred mixture of $Pd(PPh_3)_4$ (12.0 mg, 0.09 mmol, 0.03 equiv), Na_2CO_3 (2.0 M aqueous solution, 3.0 mL), toluene (6.0 mL) and 3-bromo-3-buten-1-ol (0.3

mL, 3.0 mmol, 1.0 equiv) was added dropwise to a solution of $ArB(OH)_2$ (3.03 mmol, 1.01 equiv) in absolute EtOH (3.0 mL). The resulting mixture was then heated to reflux for 10 h. Upon completion, the mixture was cooled to rt, filtered through a pad of Celite and washed with EtOAc. The combined organic filtrates were concentrated in *vacuo* and the residue was purified by flash column chromatography on silica gel (EtOAc/hexanes) to afford the product.

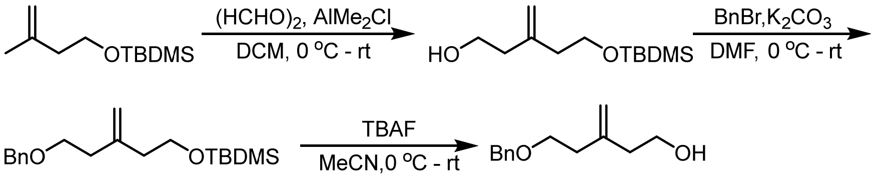

Step 1 Literature protocols for 5-(benzyloxy)-3-methylenepentan-1-ol:[5] To a Lewis acid solution (Al Me_2Cl, 2.0 M solution in hexanes, 22 mL, 44 mmol, 2.2 equiv) was added a solution of paraformaldehyde (10 mmol, 1.0 equiv) and isopentenyl alcohol (10 mmol, 1.0 equiv) in dry DCM (15 mL) at 0 °C. After the addition, the ice bath was removed, and the solution was stirred and monitored by TLC ($KMnO_4$). Upon completion, a saturated aqueous solution of NaH_2PO_4 (40 mL) and of Et_2O (40 mL) was slowly added to the reaction mixture. And the precipitated alumina complex was dissolved by the addition of 10% HCl dropwise. The organic layer was separated by decantation, and the aqueous phase was extracted with Et_2O (3 × 40 mL). The combined organic layers were dried over anhydrous $MgSO_4$, concentrated in *vacuo*. The residue was purified by flash chromatography on silica gel (hexane/EtOAc, 1:8 to 1: 4) to afford the product in 73 % yield as colorless oil.

Step 2 To a suspension of NaH (16.8 mmol, 1.2 equiv) in DMF (80 mL) was added 4-penten-1-ol (14 mmol, 1.0 equiv) at 0 °C. After 30 min, benzyl bromide (14 mmol, 1.0 equiv) was added dropwise. The resultant mixture was allowed to room temperature and stirred for 12 h. The reaction was quenched by adding a saturated aqueous solution NH_4Cl (100 mL). The aqueous phase was extracted (Et_2O, 3 × 50 mL), washed (H_2O, 2 × 50 mL), dried ($MgSO_4$), and concentrated in *vacuo*. The residue was purified by chromatography on silica gel (hexane/EtOAc, 1:6) to afford the product in 80 % yield as colorless oil.

Step 3 To the solution of the crude product (11.2 mmol, 1 equiv) in 30 mL of dry tetrahydrofuran was added tetrabutylammonium fluoride (33.6 mmol, 3 equiv). The resulting solution was stirred at room temperature for 4 h and then quenched by adding a saturated aqueous solution of NH_4Cl (40 mL). After removal of THF, the aqueous layer was extracted with EtOAc (3 × 40 mL). The organic layers were combined, washed with brine (20 mL), dried over anhydrous Na_2SO_4, and concentrated in *vacuo*, affording the desired product in 82 % yield as colorless oil.

Synthesis of imidazoles:

MsCl (1.5 equiv) was added to a stirred solution of alcohol (1.0 equiv) and Et_3N (3.0 equiv) in CH_2Cl_2 at 0 °C. After stirring for 30 min at 0 °C, the reaction mixture were quenched by adding saturated aqueous $NaHCO_3$. The organic layer was separated and the aqueous layer was further extracted with CH_2Cl_2. The combined organic layers were then dried over $MgSO_4$, filtered, concentrated under high vacuum, and the residue was used without further purification.

To an oven-dried 3-necked flask was added 2-(trifluoromethyl)-1H-benzo[*d*]imidazole (0.5 M) in THF. The solution was cooled to 0 °C and NaH (2 equiv, 60% in mineral oil) was slowly added. The

resulting mixture was allowed to warm to room temperature and stirred for 10 min. Then 3-phenylbut-3-en-1-yl methanesulfonate (1.2 equiv) was added, and the mixture was heated to reflux and monitored by TLC until the total disappearance of benzoimidazole. Then the reaction was quenched with H_2O, and concentrated in *vacuo*. The resulting oil was dissolved in CH_2Cl_2 and washed with H_2O, extracted with CH_2Cl_2. The combined organic extracts were dried over anhydrous Na_2SO_4 and concentrated in *vacuo*. The residue was purified by flash chromatography on silica gel, eluting with EtOAc/*n*-hexane.

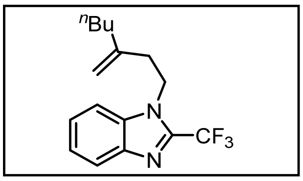

1-(3-Methyleneheptyl)-2-(trifluoromethyl)-1*H*-benzo[*d*]imidazole (1b′)

Yellow oil (60% yield). **^{1}H NMR** (400 MHz, $CDCl_3$) δ 7.89 (d, *J* = 8.0 Hz, 1H), 7.46 – 7.44 (m, 2H), 7.40 – 7.36 (m, 1H), 4.90 (s, 1H), 4.81 (s, 1H), 4.40 (t, *J* = 8.0 Hz, 2H), 2.54 (t, *J* = 8.0 Hz, 2H), 2.09 (t, *J* = 8.0 Hz, 2H). 1.48 – 1.41 (m, 2H), 1.39 – 1.26 (m, 2H), 0.92 (t, *J* = 7.2 Hz, 3H). **^{13}C NMR** (100 MHz, $CDCl_3$) δ 145.4, 141.3, 140.5 (q, $^2J_{C-F}$ = 38.3 Hz), 135.3, 125.5, 123.8, 122.0, 119.4 (q, $^1J_{C-F}$ = 269.6 Hz), 112.0, 110.5, 44.4, 36.2, 36.0, 29.9, 22.5, 14.0. **^{19}F NMR** (376 MHz, $CDCl_3$) δ -62.1. **HRMS (ESI)** *m/z:* $[M+H]^+$ Calcd. for $C_{16}H_{20}F_3N_2$ 297.1573; Found 297.1576.

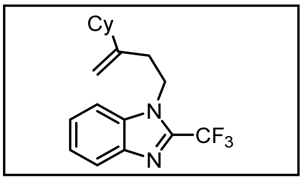

1-(3-Cyclohexylbut-3-en-1-yl)-2-(trifluoromethyl)-1*H*-benzo[*d*]imidazole (1c′)

Yellow oil (73% yield). **^{1}H NMR** (400 MHz, $CDCl_3$) δ 7.89 (d, *J* = 8.12 Hz, 1H), 7.46 – 7.42 (m, 2H), 7.40 – 7.34 (m, 1H), 4.91 (s, 1H), 4.80 (s, 1H), 4.39 (t, *J* = 8.0 Hz, 2H), 2.54 (t, *J* = 8.0 Hz, 2H), 2.01 – 1.60 (m, 6H), 1.38 – 1.05 (m, 5H). **^{13}C NMR** (100 MHz, $CDCl_3$) δ 150.8, 141.3, 140.5 (q, $^2J_{C-F}$ = 38.3 Hz), 135.3, 125.4, 123.7, 121.8, 119.3 (q, $^1J_{C-F}$ = 269.6 Hz), 110.5, 110.1, 44.8, 44.6, 34.7, 32.3, 26.7, 26.3. **^{19}F NMR** (376 MHz, $CDCl_3$) δ -61.1. **HRMS (ESI)** *m/z:* $[M+H]^+$ Calcd. for $C_{18}H_{22}F_3N_2$ 323.1730; Found 323.1738.

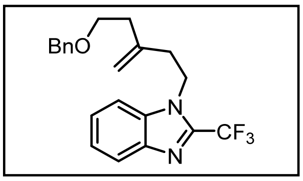

1-(5-(Benzyloxy)-3-methylenepentyl)-2-(trifluoromethyl)-1*H*-benzo[*d*]imidazole (1d′)

Yellow oil (68% yield) . **^{1}H NMR** (400 MHz, $CDCl_3$) δ 7.91 – 7.84 (m, 1H), 7.45 – 7.35 (m, 3H), 7.34 – 7.31 (m, 4H), 7.30 – 7.27 (m, 1H), 4.96 (s, 1H), 4.89 (s, 1H), 4.53 (s, 2H), 4.39 (t, *J* = 8.0 Hz, 2H), 3.62 (t, *J* = 6.4 Hz, 2H), 2.58 (t, *J* = 8.0 Hz, 2H), 2.42 (t, *J* = 6.4 Hz, 2H). **^{13}C NMR** (100 MHz, $CDCl_3$) δ 142.7, 141.3, 140.5 (q, $^2J_{C-F}$ = 38.3 Hz), 138.2, 135.3, 128.6, 127.9, 127.8, 125.5, 123.8, 121.9, 119.3 (q, $^1J_{C-F}$ = 269.6 Hz), 113.9, 110.6, 73.3, 69.0, 44.2, 36.5, 36.5. **^{19}F NMR** (376 MHz, $CDCl_3$) δ -62.0. **HRMS (ESI)** *m/z:* $[M+H]^+$ Calcd. for $C_{21}H_{22}F_3N_2O$ 375.1679; Found 375.1676.

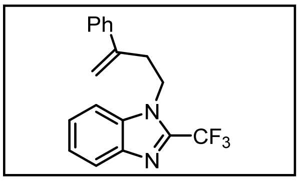

1-(3-Phenylbut-3-en-1-yl)-2-(trifluoromethyl)-1*H*-benzo[*d*]imidazole (1e′)

Yellow oil (40% yield). **^{1}H NMR** (400 MHz, $CDCl_3$) *δ* 7.77 (d, *J* = 7.6 Hz, 1H), 7.50 – 7.27 (m, 8 H), 5.40 (s, 1H), 5.15 (s, 1H), 4.39 (t, *J* = 7.6 Hz, 2H), 3.06 (t, *J* = 7.6 Hz, 2H). **^{13}C NMR** (100 MHz, $CDCl_3$) *δ* 144.0, 141.2, 140.4 (q, $^2J_{C\text{-}F}$ = 38.2 Hz), 139.5, 135.4, 128.8, 128.3, 126.1, 125.4, 123.8, 121.8, 119.3 (q, $^1J_{C\text{-}F}$ = 269.8 Hz), 115.5, 110.6, 44.5, 36.0. **^{19}F NMR** (376 MHz, $CDCl_3$) *δ* -61.3. **HRMS (ESI)** *m/z:* $[M+H]^+$ Calcd. for $C_{18}H_{16}F_3N_2$ 317.1260; Found 317.1258.

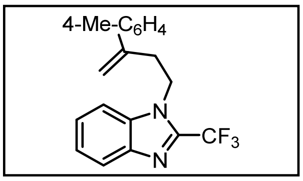

1-(3-(*p*-Tolyl)but-3-en-1-yl)-2-(trifluoromethyl)-1*H*-benzo[*d*]imidazole (1f′)

Yellow oil (46% yield). **^{1}H NMR** (400 MHz, $CDCl_3$) *δ* 7.87 (d, *J* = 7.6 Hz, 1H), 7.44 – 7.29 (m, 5H), 7.20 (d, *J* = 8.0 Hz, 2H), 5.37 (s, 1H), 5.09 (s, 1H), 4.33 (t, *J* = 7.6 Hz, 2H), 2.61 (t, *J* = 7.6 Hz, 2H), 2.38 (s, 3H). **^{13}C NMR** (100 MHz, $CDCl_3$) *δ* 143.8, 141.2, 140.4 (q, $^2J_{C\text{-}F}$ = 38.6 Hz), 138.2, 136.5, 135.4, 129.5, 125.9, 125.4, 123.8, 121.8, 119.3 (q, $^1J_{C\text{-}F}$ = 269.8 Hz), 114.7, 110.6, 44.5, 36.0, 21.3. **^{19}F NMR** (376 MHz, $CDCl_3$) *δ* -62.3. **HRMS (ESI)** *m/z:* $[M+H]^+$ Calcd. for $C_{19}H_{18}F_3N_2$ 331.1417; Found 331.1418.

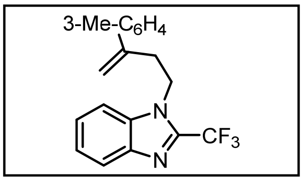

1-(3-(*m*-Tolyl)but-3-en-1-yl)-2-(trifluoromethyl)-1*H*-benzo[*d*]imidazole (1g′)

Yellow oil (43% yield). **^{1}H NMR** (400 MHz, $CDCl_3$) *δ* 7.87 (d, *J* = 7.2 Hz, 1H), 7.42 – 7.33 (m, 2H), 7.31 – 7.25 (m, 2H), 7.23 (d, *J* = 7.0 Hz, 2H), 7.15 (d, *J* = 7.2 Hz, 1H), 5.39 (s, 1H), 5.13 (s , 1H), 4.38 (t, *J* = 8.0 Hz, 2H), 3.05 (t, *J* = 8.0 Hz, 2H), 2.38 (s, 3H). ^{13}C NMR (100 MHz, $CDCl_3$) *δ* 144.1, 141.2, 140.3(q, $^2J_{C\text{-}F}$ = 38.3 Hz), 139.5, 138.4, 135.4, 129.0, 128.7, 126.9, 125.4, 123.8, 123.2, 121.8, 119.3 (q, $^1J_{C\text{-}F}$ = 269.6 Hz), 115.3, 110.6, 44.5, 36.1, 21.6. **^{19}F NMR** (376 MHz, $CDCl_3$) *δ* -61.9. **HRMS (ESI)** *m/z:* $[M+H]^+$ Calcd. for $C_{19}H_{18}F_3N_2^+$: 331.1417; Found 331.1419.

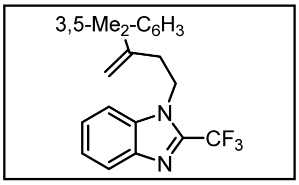

1-(3-(3,5-Dimethylphenyl)but-3-en-1-yl)-2-(trifluoromethyl)-1*H*-benzo[*d*]imidazole (1h′)

Yellow oil (39% yield). **^{1}H NMR** (400 MHz, $CDCl_3$) *δ* 7.87 (d, *J* = 7.2 Hz, 1H), 7.45 – 7.27 (m, 3H), 7.02 (s, 2H), 6.98 (s, 1H), 5.38 (s, 1H), 5.12 (s, 1H), 4.37 (t, *J* = 8.0 Hz, 2H), 3.04 (t, *J* = 8.0 Hz, 2H), 2.34 (s, 6H). **^{13}C NMR** (100 MHz, $CDCl_3$) *δ* 144.2, 141.2, 140.4 (q, $^2J_{C\text{-}F}$ = 38.2 Hz), 139.4, 138.3, 135.4, 129.9,

125.3, 124.0, 123.7, 121.8, 119.3 (q, $^{1}J_{C\text{-}F}$ = 269.6 Hz), 115.0, 110.7, 44.5, 36.1, 21.5. ^{19}**F NMR** (376 MHz, $CDCl_3$) δ -62.0. **HRMS (ESI)** *m/z:* $[M+H]^+$ Calcd. for $C_{24}H_{20}F_4N_2$ 345.1573; Found 345.1580.

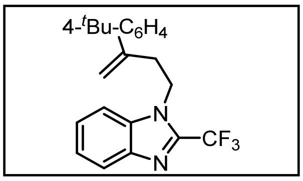

1-(3-(4-(*tert*-Butyl)phenyl)but-3-en-1-yl)-2-(trifluoromethyl)-1*H*-benzo[*d*]imidazole (1i')

White soild (60% yield), m.p. 55-56 °C. ^{1}**H NMR** (400 MHz, $CDCl_3$) δ 7.90 (d, *J* = 7.6 Hz, 1H), 7.46 – 7.28 (m, 7H), 5.44 (s, 1H), 5.14 (s, 1H), 4.44 (t, *J* = 8.0 Hz, 2H), 3.09 (t, *J* = 8.0 Hz, 2H), 1.39 (s, 9H). ^{13}**C NMR** (100 MHz, $CDCl_3$) δ 151.4, 143.7, 141.2, 140.4 (q, $^{2}J_{C\text{-}F}$ = 38.2 Hz), 136.5, 135.4, 125.7, 125.4, 123.7, 121.8, 119.3 (q, $^{1}J_{C\text{-}F}$ = 269.6 Hz), 114.7, 110.6, 44.6, 35.9, 34.7, 31.4. ^{19}**F NMR** (376 MHz, $CDCl_3$) δ -62.0. **HRMS (ESI)** *m/z:* $[M+H]^+$ Calcd. for $C_{24}H_{20}F_4N_2^+$: 373.1886; Found 373.1893.

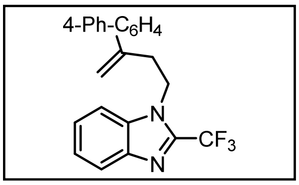

7-([1,1'-Biphenyl]-4-yl)-1-(trifluoromethyl)-6,7,8,9-tetrahydro-2,9a-diazabenzo[*cd*]azulene (1j')

White soild (38% yield). m.p. 83-84 °C. ^{1}**H NMR** (400 MHz, $CDCl_3$) δ 7.87 (d, *J* = 7.2 Hz, 1H), 7.72 – 7.58 (m, 4H), 7.56 – 7.30 (m, 8H), 5.46 (s, 1H), 5.17 (s, 1H), 4.43 (t, *J* = 8.0 Hz, 2H), 3.10 (t, *J* = 8.0 Hz, 2H). ^{13}**C NMR** (100 MHz, $CDCl_3$) δ 143.5, 141.2, 141.1, 140.5, 140.4 (q, $^{2}J_{C\text{-}F}$ = 38.2 Hz), 138.3, 135.4, 129.0, 127.7, 127.5, 127.1, 126.4, 125.4, 123.8, 121.8, 119.3 (q, $^{1}J_{C\text{-}F}$ = 269.6 Hz), 115.5, 110.6, 44.5, 35.9. ^{19}**F NMR** (376 MHz, $CDCl_3$) δ -61.9. **HRMS (ESI)** *m/z:* $[M+H]^+$ Calcd. for $C_{24}H_{20}F_4N_2$ 393.1573; Found 393.1572.

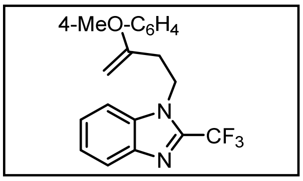

1-(3-(4-Methoxyphenyl)but-3-en-1-yl)-2-(trifluoromethyl)-1*H*-benzo[*d*]imidazole (1k')

White soild (53% yield). m.p. 59-61 °C. ^{1}**H NMR** (400 MHz, $CDCl_3$) δ 7.87 (d, *J* = 7.6 Hz, 1H), 7.46 – 7.23 (m, 5H), 6.92 (d, *J* = 8.8 Hz, 2H), 5.31 (s, 1H), 5.04 (s, 1H), 4.38 (t, *J* = 8.0 Hz, 2H), 3.84 (s, 3H), 3.03 (t, *J* = 8.0 Hz, 2H). ^{13}**C NMR** (100 MHz, $CDCl_3$) δ 159.7, 143.3, 141.2, 140.4 (q, $^{2}J_{C\text{-}F}$ = 38.2 Hz), 135.4, 131.9, 127.2, 125.4, 123.7, 121.8, 119.3 (q, $^{1}J_{C\text{-}F}$ = 269.6 Hz), 114.1, 113.8, 110.6, 55.4, 44.5, 36.0. ^{19}**F NMR** (376 MHz, $CDCl_3$) δ -62.5. **HRMS (ESI)** *m/z:* $[M+H]^+$ Calcd. for $C_{19}H_{18}F_3N_2O$ 347.1366; Found 347.1367.

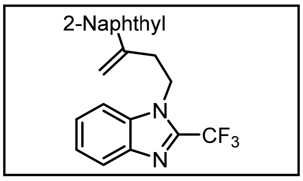

1-(3-(Naphthalen-2-yl)but-3-en-1-yl)-2-(trifluoromethyl)-1*H*-benzo[*d*]imidazole (1l')

White soild (33% yield). m.p. 81-84 °C. **^{1}H NMR** (400 MHz, $CDCl_3$) *δ* 7.91 – 7.79 (m, 5H), 7.58 (dd, *J* = 8.4, 1.6 Hz, 1H), 7.55 – 7.47 (m, 2H), 7.42 – 7.33 (m, 2H), 7.32 – 7.27 (m, 1H), 5.55 (s, 1H), 5.24 (s, 1H), 4.44 (t, *J* = 8.0 Hz, 2H), 3.17 (t, *J* = 8.0 Hz, 2H). **^{13}C NMR** (100 MHz, $CDCl_3$) *δ* 143.7, 141.2, 140.5 (q, $^{2}J_{C-F}$ = 38.3 Hz), 136, 135.4, 133.5, 133.2, 128.6, 128.3, 127.8, 126.7, 126.5, 125.4, 124.9, 124.3, 123.8, 121.9, 119.4 (q, $^{1}J_{C-F}$ = 269.6 Hz), 116.0, 110.6, 44.5, 36.0. **^{19}F NMR** (376 MHz, $CDCl_3$) *δ* -61.9. **HRMS (ESI)** *m/z:* $[M+H]^+$ Calcd. for $C_{24}H_{20}F_4N_2$ 367.1417; Found 367.1424.

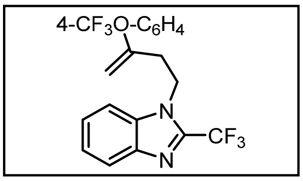

1-(3-(4-(Trifluoromethoxy)phenyl)but-3-en-1-yl)-2-(trifluoromethyl)-1*H*-benzo[*d*]imidazole (1m')

Yellow oil (45% yield). **^{1}H NMR** (400 MHz, $CDCl_3$) *δ* 7.72 (d, *J* = 7.6 Hz, 1H), 7.32 – 7.19 (m, 4H), 7.14 (d, *J* = 8.4 Hz, 1H), 7.08 (d, *J* = 8.0 Hz, 2H), 5.26 (s, 1H), 5.05 (s, 1H), 4.25 (t, *J* = 8.0 Hz, 2H), 2.91 (t, *J* = 8.0 Hz, 2H). **^{13}C NMR** (100 MHz, $CDCl_3$) *δ* 149.1, 142.8, 141.2, 140.3 (q, $^{2}J_{C-F}$ = 38.4 Hz), 138.2, 135.3, 127.4, 125.4, 123.7, 122.0 (q, $^{1}J_{C-F}$ = 269.7 Hz), 121.8, 121.1 , 119.9 (q, $^{1}J_{C-F}$ = 255.6 Hz), 116.3, 110.4, 44.2, 35.7. **^{19}F NMR** (376 MHz, $CDCl_3$) *δ* -65.0, -69.2. **HRMS (ESI)** *m/z:* $[M+H]^+$ Calcd. for $C_{19}H_{18}F_3N_2$ 401.1083; Found 401.1089.

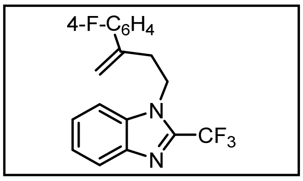

1-(3-(4-Fluorophenyl)but-3-en-1-yl)-2-(trifluoromethyl)-1*H*-benzo[*d*]imidazole (1n')

White soild (44% yield). m.p. 73-74 °C. **^{1}H NMR** (400 MHz, $CDCl_3$) *δ* 7.72 (d, *J* = 7.6 Hz, 1H), 71 – 7.18 (m, 2H), 7.16 – 7.11 (m, 1H), 6.91 (t, *J* = 8.6 Hz, 1H), 5.20 (s, 1H), 4.98 (s, 1H), 4.23 (t, *J* = 8.0 Hz, 2H), 2.89 (t, *J* = 8.0 Hz, 2H). **^{13}C NMR** (100 MHz, $CDCl_3$) *δ* 162.8 (d, $^{1}J_{C-F}$ = 247.7 Hz), 143.1, 141.3, 140.4 (q, $^{2}J_{C-F}$ = 38.1 Hz), 135.7 (d, $^{3}J_{C-F}$ = 3.0 Hz), 135.4, 127.8 (d, $^{2}J_{C-F}$ = 8.1 Hz), 125.5, 123.8, 121.9, 119.4 (q, $^{1}J_{C-F}$ = 269.9 Hz), 115.8, 115.5 (d, $^{2}J_{C-F}$ = 9.1 Hz), 110.5, 44.4, 36.0. **^{19}F NMR** (376 MHz, $CDCl_3$) *δ* -61.9, -113.8. **HRMS (ESI)** *m/z:* $[M+H]^+$ Calcd. for $C_{18}H_{15}F_4N_2$ 335.1166; Found 335.1166.

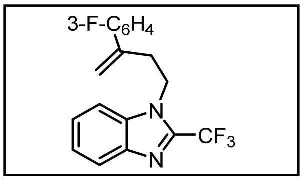

1-(3-(3-Fluorophenyl)but-3-en-1-yl)-2-(trifluoromethyl)-1*H*-benzo[*d*]imidazole (1o')

Yellow oil (50% yield). **^{1}H NMR (400 MHz, $CDCl_3$)** *δ* 7.74 (d, *J* = 8.0 Hz, 1H), 7.33 – 7.12 (m, 4H), 7.05 (d, *J* = 7.2 Hz, 1H), 6.96 (d, *J* = 10.0 Hz, 1H), 6.91 – 6.87 (m, 1H), 5.29 (s, 1H), 5.05 (s, 1H), 4.25 (t, *J* = 8.0 Hz, 2H), 2.90 (t, *J* = 8.0 Hz, 2H). **^{13}C NMR (100 MHz, $CDCl_3$)** *δ* 163.1 (d, $^{1}J_{C-F}$ = 244.6 Hz,), 142.8, 141.7 (d, $^{3}J_{C-F}$ = 7.3 Hz), 141.1, 140.3 (q, $^{2}J_{C-F}$ = 38.2 Hz), 135.2, 130.2 (d, $^{3}J_{C-F}$ = 8.4 Hz), 125.4, 123.7, 121.7, 121.6 (d, $^{4}J_{C-F}$ = 2.7 Hz), 119.2 (q, $^{1}J_{C-F}$ = 269.7 Hz), 116.5, 115.0 (d, $^{2}J_{C-F}$ = 21.2 Hz), 113.0 (d, $^{2}J_{C-F}$ = 22.1 Hz), 110.4, 44.2, 35.6. **^{19}F NMR** (376 MHz, $CDCl_3$) *δ* -61.9, -112.5. **HRMS (ESI)** *m/z:* $[M+H]^+$ Calcd. for $C_{18}H_{15}F_4N_2$ 335.1166; Found 335.1172.

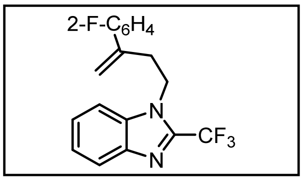

1-(3-(2-Fluorophenyl)but-3-en-1-yl)-2-(trifluoromethyl)-1*H*-benzo[*d*]imidazole (1p′)

Yellow oil (53% yield). **^{1}H NMR** (400 MHz, $CDCl_3$) δ 7.86 (d, *J* = 7.6 Hz, 1H), 7.44 – 7.25 (m, 5H), 7.18 – 7.07 (m, 2H), 5.35 (s, 1H), 5.33 (s, 1H), 4.38 (t, *J* = 8.0 Hz, 2H), 3.05 (t, *J* = 8.0 Hz, 2H). **^{13}C NMR** (100 MHz, $CDCl_3$) δ 159.9 (d, $^1J_{C-F}$ = 245.8 Hz,), 141.2, 140.6, 140.4 (q, $^2J_{C-F}$ = 38.3 Hz), 135.39, 130.1 (d, $^3J_{C-F}$ = 4.1 Hz), 129.8 (d, $^3J_{C-F}$ = 8.5 Hz), 128.2 (d, $^2J_{C-F}$ = 13.8 Hz), 125.4, 124.5 (d, J_{C-F} = 3.4 Hz), 123.8, 121.8, 119.3 (q, $^1J_{C-F}$ = 269.6 Hz), 119.1 (d, $^4J_{C-F}$ = 1.9 Hz), 116.1 (d, $^2J_{C-F}$ = 22.7 Hz), 110.5, 44.4, 36.7. **^{19}F NMR** (376 MHz, $CDCl_3$) δ -62.0, -114.7. **HRMS (ESI)** *m/z:* $[M+H]^+$ Calcd. for $C_{18}H_{15}F_4N_2$ 335.1166; Found 335.1172.

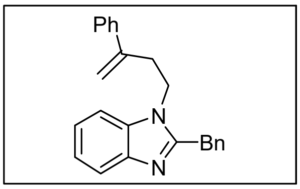

2-Benzyl-1-(3-phenylbut-3-en-1-yl)-1*H*-benzo[*d*]imidazole(1q′)

White solid (35% yield). m.p. 109-110 °C. **^{1}H NMR** (400 MHz, $CDCl_3$) δ 7.80 – 7.73 (m, 4H), 7.42 – 7.30 (m, 4H), 7.29 – 7.17 (m, 5H), 7.07 (d, *J* = 6.8 Hz, 2H), 5.29 (d, *J* = 6.0 Hz, 1H), 4.87 (s, 1H), 4.16 (s, 2H), 4.09 – 3.99 (m, 2H), 2.74 (t, *J* = 7.6 Hz, 2H). **^{13}C NMR** (100 MHz, $CDCl_3$) δ 153.2, 144.2, 142.8, 139.7, 136.5 , 135.2, 128.9, 129.8, 128.5, 128.2, 127.1, 126.1, 122.5, 122.1, 119.7, 115.3, 109.5, 43.1, 35.1, 34.6. **HRMS (ESI)** *m/z:* $[M+H]^+$ Calcd. for $C_{24}H_{23}N_2$ 339.1856; Found 339.1859.

Method D:

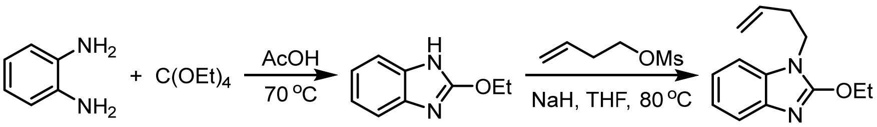

A mixture of benzene-1,2-diamine (800 mg, 7.39 mmol), tetraethyl orthocarbonate (1.49 g, 7.76 mmol) and acetic acid (444 mg) was heated at 70 °C for 2 h. Then the solution was cooled to room temperature, and 5% aqueous potassium hydroxide solution was added slowly. The reaction mixture was stirred for 1 h. The precipitation was collected by filtration, washed with water, and dried to give 2-ethoxy-1H-benzo[d]imidazole (560 mg) as a white solid in 47% yield.

To an oven-dried 3-necked flask was added 2-ethoxy-1H-benzo[*d*]imidazole (486 mg, 3 mmol) in THF, and then NaH (240 mg, 6 mmol) (60% in mineral oil) was slowly added at 0 °C. The resulting mixture was allowed to warm to room temperature and stirred for 10 min, and then methanesulfonate (541 mg, 3.6 mmol) was added. The mixture was heated to reflux and monitored by TLC until the total disappearance of 2-ethoxy-1H-benzo[*d*]imidazole. Upon completion, the reaction was quenched with H_2O, concentrated in *vacuo*. The resulting oil was dissolved in CH_2Cl_2, washed with H_2O, extracted with CH_2Cl_2. The combined organic extracts were dried over anhydrous Na_2SO_4 and concentrated in *vacuo*. The residue was purified by flash chromatography on silica gel (EtOAc/hexane = 1:3), affording the product as colorless oil (390 mg, 60% yield).

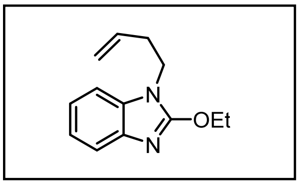

1-(But-3-en-1-yl)-2-ethoxy-1*H*-benzo[*d*]imidazole (1q)

Yellow oil (63% yield). **^{1}H NMR** (400 MHz, $CDCl_3$) δ 7.65 – 7.46 (m, 1H), 7.21 – 7.04 (m, 3H), 5.83 – 5.70 (m, 1H), 5.07 – 4.94 (m, 1H), 4.59 (q, *J* = 7.2 Hz, 2H), 3.99 (t, *J* = 7.2 Hz, 2H), 2.48 (q, *J* = 7.2 Hz, 2H), 1.47 (t, *J* = 7.2 Hz, 3H). **^{13}C NMR** (100 MHz, $CDCl_3$) δ 157.3, 140.1, 134.2, 133.4, 121.3, 120.6, 117.53, 117.52, 108.1, 66.0, 41.4, 33.3, 14.7. **HRMS (ESI)** *m/z:* $[M+H]^+$ Calcd. for $C_{13}H_{17}N_2O$ 217.1335; Found 217.1334.

Method E:

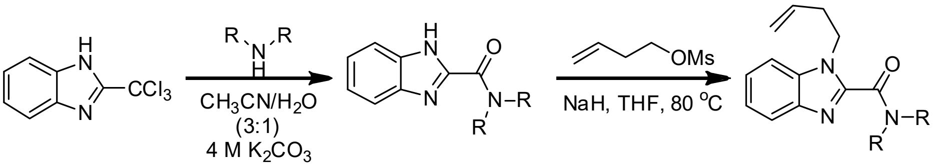

To a suspension of 2-(trichloromethyl)-1H-benzo[d]imidazole (1 g, 4.2 mmol) in acetonitrile and water (3:1 ratio, 20 mL) was added amine (8.4 mmol) and aq. K_2CO_3 (3.0 mL, 4M). The reaction was stirred for 24 h before diluting with $NaHCO_3$ (3 mL) and extracting with dichloromethane. The organic extract was dried and evaporated to give a crude product which was purified by chromatography on silica gel (4% MeOH/CH_2Cl_2) to afford amide product.

To an oven-dried 3-necked flask was added amide (462 mg, 2 mmol) in THF. The solution was cooled to 0 °C, and then NaH (160 mg, 4 mmol) (60% in mineral oil) was slowly added. The resulting mixture was allowed to warm to room temperature and stirred for 10 min, and then methanesulfonate (361 mg, 2.4 mmol) was added. The mixture was heated to reflux and monitored by TLC. Upon completion, the reaction was quenched with H_2O, and the resulting solution concentrated in *vacuo*. The resulting oil was dissolved in CH_2Cl_2, washed with H_2O, extracted with CH_2Cl_2 and the combined organic extracts were dried over anhydrous Na_2SO_4 and concentrated in *vacuo*. The residue was purified by flash chromatography on silica gel (EtOAc/hexane = 1:1).

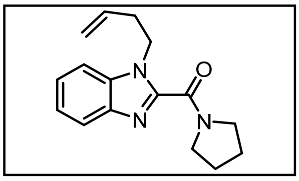

(1-(But-3-en-1-yl)-1*H*-benzo[*d*]imidazol-2-yl)(pyrrolidin-1-yl)methanone (1o)

White solid (70% yield). m.p. 60-62 °C. **^{1}H NMR** (400 MHz, $CDCl_3$) δ 7.79 (d, *J* = 7.6 Hz, 1H), 7.44 (d, *J* = 8.0 Hz, 1H), 7.40 – 7.29 (m, 2H), 5.83 – 5.70 (m, 1H), 4.51 (t, *J* = 7.1 Hz, 2H), 4.04 – 3.94 (m, 2H), 3.84 (q, *J* = 7.3 Hz, 4H), 3.79 – 3.72 (m, 2H), 2.61 (q, *J* = 7.2 Hz, 2H). **^{13}C NMR** (100 MHz, $CDCl_3$) δ 160.4, 144.3, 141.6, 134.8, 134.1, 124.4, 123.1, 121.0, 118.1, 110.5, 67.3, 66.9, 48.0, 44.2, 42.8, 34.5. **HRMS (ESI)** *m/z:* $[M+H]^+$ Calcd. for $C_{16}H_{20}N_3O$ 270.1601; Found 270.1601.

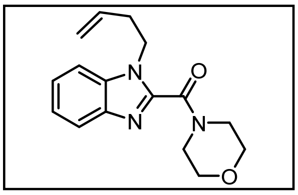

(1-(But-3-en-1-yl)-1*H*-benzo[*d*]imidazol-2-yl)(morpholino)methanone (1p)

White solid (76% yield). m.p. 68-70 °C. **^{1}H NMR** (400 MHz, $CDCl_3$) δ 7.80 (d, *J* = 8.0 Hz, 1H), 7.44 (d, *J* = 7.6 Hz, 1H), 7.41 – 7.30 (m, 1H), 5.83 – 5.70 (m, 1H), 5.09 – 4.89 (m, 2H), 4.51 (t, *J* = 7.2 Hz, 2H), 4.03 – 3.95 (m, 2H), 3.84 (q, *J* = 7.6 Hz, 4H), 3.81 – 3.63 (m, 2H), 2.61 (q, *J* = 6.8 Hz, 2H). **^{13}C NMR** (100 MHz, $CDCl_3$) δ 160.4, 144.3, 141.6, 134.8, 134.2, 124.4, 123.1, 121.0, 118.1, 110.5, 66.9, 67.3, 48.0, 44.3, 42.8, 34.5. **HRMS (ESI)** *m/z:* $[M+H]^+$ Calcd. for $C_{16}H_{20}N_3O_2$ 286.1550; Found 286.1552.

Method F:

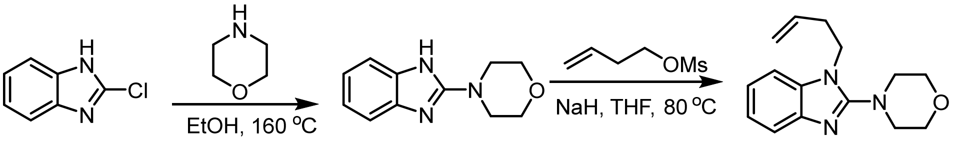

A mixture of 2-chloro-1H-benzimidazole (5 g, 33 mmol), pyrrolidine (9.3 g, 130 mmol) and ethanol (20 mL) were heated at 160-170°C for 6 hours and then the solvent was evaporated. The resulting residue was mixed with water. The resulting solid was isolated by filtration, washed with water and then dried to provide a crude product as tan powder (6.2 g).

To an oven-dried 3-necked flask was added 2-(pyrrolidin-1-yl)-1H-benzo[*d*]imidazole (374 mg, 2 mmol) in THF. The solution was cooled to 0 °C, and then NaH (160 mg, 4 mmol) (60% in mineral oil) was slowly added. The resulting mixture was allowed to warm to room temperature and was stirred for 10 min, and then methanesulfonate (361 mg, 2.4 mmol) was added. The mixture was heated to reflux and monitored by TLC until the total disappearance of 2-(pyrrolidin-1-yl)-1H-benzo[*d*]imidazole. Then the reaction was quenched with H_2O and concentrated in *vacuo*. The resulting oil was dissolved in CH_2Cl_2, washed with H_2O, extracted with CH_2Cl_2. The combined organic extracts were dried over anhydrous Na_2SO_4 and concentrated in *vacuo*. The reaction was purified by flash chromatography on silica gel (EtOAc/hexane = 1:1), affording the product as a white solid (386 mg, 80% yield).

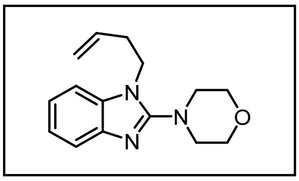

1-(But-3-en-1-yl)-2-(pyrrolidin-1-yl)-1*H*-benzo[d]imidazole (1r)

White solid (33% yield). m.p. 89-91 °C. **^{1}H NMR** (400 MHz, $CDCl_3$) δ 7.63 (d, *J* = 7.6 Hz, 1H), 7.25 (d, *J* = 3.2 Hz, 1H), 7.22 – 7.16 (m, 2H), 5.83 – 5.73 (m, 1H), 5.15 – 5.00 (m, 2H), 4.21 – 4.04 (m, 2H), 3.98 – 3.85 (m, 4H), 3.40 – 3.27 (m, 4H), 2.58 (q, *J* = 7.2 Hz, 2H). **^{13}C NMR** (100 MHz, $CDCl_3$) δ 157.6, 141.6, 134.7, 134.0, 121.9, 121.6, 118.6, 117.8, 109.3, 66.8, 51.4, 43.4, 33.2. **HRMS (ESI)** *m/z:* $[M+H]^+$ Calcd. for $C_{13}H_{17}N_2O$ 258.1601; Found 258.1607.

Supplementary Note 2

Supplementary Table 1. Catalyst examination

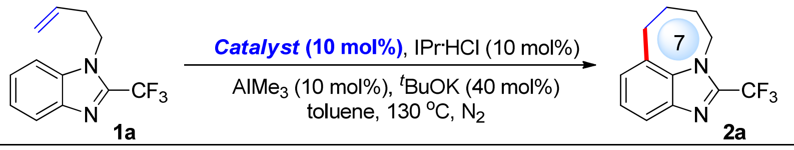

entry	*Catalyst* (10 mol%)	yield (%) [a,b]
1	Ni(cod)$_2$	98
2	NiCl$_2$	0
3	Ni(OAc)$_2$	0
4	NiBr$_2$·DME	0
5[c]	NiBr$_2$·DME/Zn	42
6	Ni(acac)$_2$	20
7[d]	Ni(acac)$_2$	60
8[e]	Ni(acac)$_2$	96

[a]Reaction condition: Substrate (0.2 mmol), toluene (1.0 mL), under N_2 for 3 h; [b]Yield was dertermined by ^{1}H NMR analysis with CH_2Br_2 as an internal standard. [c]Zn (20 mol%); [d]Al(Me)$_3$ (30 mol%); e: Al(Me)$_3$ (50 mol%)

Supplementary Table 2. Lewis acid loading effect

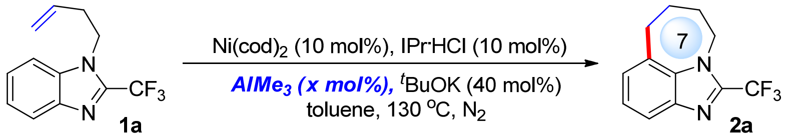

entry	*AlMe$_3$ (x mol%)*	yield (%) [a,b]
1	0	38
2	10	98
3	20	98
4\	30	98

[a]Reaction condition: Substrate (0.2 mmol), toluene (1.0 mL), under N_2 for 3 h; [b]Yield was dertermined by ^{1}H NMR analysis with CH_2Br_2 as an internal standard.

Supplementary Table 3. Ligand effect

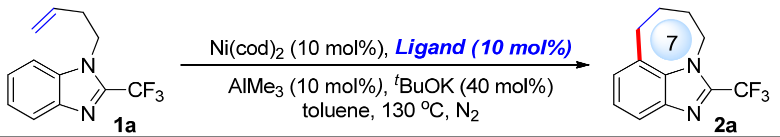

entry	*Ligand (10 mol%)*	yield (%) [a,b]
1	IMes·HCl	82
2	IPr·HCl	98
3	SIPr·HCl	86
4	dppe	0
5	BINAP	0
6	PPh$_3$	0
7	PCy$_3$	0
8	1,10-phen	0

[a]Reaction condition: Substrate (0.2 mmol), toluene (1.0 mL), under N_2 for 3 h; [b]Yield was dertermined by ^{1}H NMR analysis with CH_2Br_2 as an internal standard.

Supplementary Table 4. Lewis acid effect

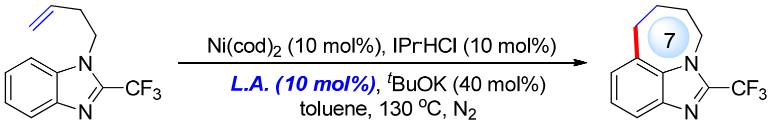

1a **2a**

entry	***L.A. (10 mol%)***	yield (%) [a,b]
1	$AlMe_3$	98
2	$AlEt_3$	97
3	$Al(^iBu)_3$	98
4	$Al(C_8H_{17})_3$	97
5	$AlMe_2Cl$	92
6	$B(C_6F_5)_3$	55
7	$BF_3{\cdot}OEt_2$	0
8	$AlPh_3$	80
9	Mg^nBu_2	88
10	$ZnMe_2$	92

[a]Reaction condition: Substrate (0.2 mmol), toluene (1.0 mL), under N_2 for 3 h; [b]Yield was dertermined by ^1H NMR analysis with CH_2Br_2 as an internal standard.

Supplementary Table 5. Temperature effect

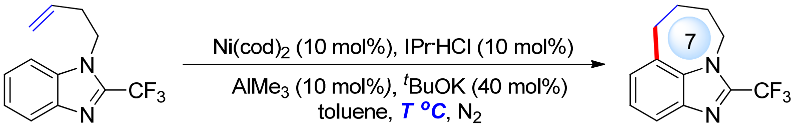

1a **2a**

entry	***T °C***	yield (%) [a,b]
1	130	98
2	120	92
3	110	68
4	100	52

[a]Reaction condition: Substrate (0.2 mmol), toluene (1.0 mL), under N_2 for 3 h; [b]Yield was dertermined by ^1H NMR analysis with CH_2Br_2 as an internal standard.

Supplementary Table 6. Base effect

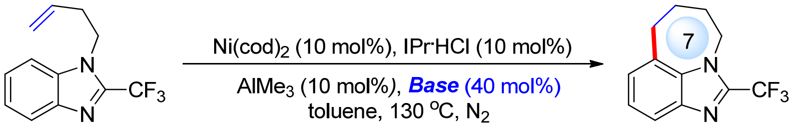

1a **2a**

entry	***Base*** (40 mol%)	yield (%) [a,b]
1	KO^tBu	98
2	NaO^tBu	98
3	LiO^tBu	0
4	K_2CO_3	0
5	Cs_2CO_3	0
6	CH_3ONa	0
7	KOH	0

[a]Reaction condition: Substrate (0.2 mmol), toluene (1.0 mL), under N_2 for 3 h; [b]Yield was dertermined by ^1H NMR analysis with CH_2Br_2 as an internal standard.

Supplementary Table 7. Lewis acid loading effect for substrate 1k

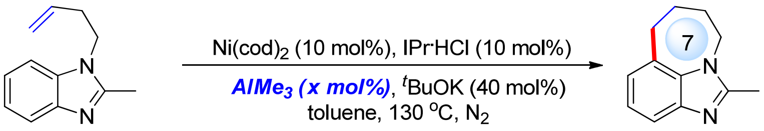

1k **2k**

entry	$AlMe_3$ (x mol%)	yield (%) [a,b]
1	10 mol%	trace
2	50 mol%	21
3	100 mol%	48
4	150 mol%	61
5	200 mol%	83
6	250 mol%	84

[a]Reaction condition: Substrate (0.2 mmol), toluene (1.0 mL), under N_2 for 3 h; [b]Yield was dertermined by ^{1}H NMR analysis with CH_2Br_2 as an internal standard.

Supplementary Note 3

General Procedure for Ni-Catalyzed C7–H Cyclization

To a 15 mL oven dried tube in glove box were added Ni(cod)$_2$ (5.5 mg, 10 mol%), IPr·HCl (8.6 mg, 10 mol%), ^{t}BuOK (9 mg, 0.08 mmol, 40 mol%), benzoimidazole **1** (0.2 mmol), dry degassed toluene (2.0 mL), and $AlMe_3$ (1.0 M/hexane, 10 mol% or 60 mol% or 200 mol%). The tube was capped, taken outside the glove box, and stirred at 130 °C for 3 h. After that, the mixture was cooled to r.t., quenched with 2 mL of 5% EDTA disodium salt solution, and filtered through a short plug of silica gel, eluting with EtOAc. The filtration was concentrated in *vacuo* to afford the crude product, which was further purified by flash column chromatography on silica gel (EtOAc/hexanes).

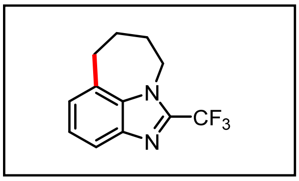

1-(Trifluoromethyl)-6,7,8,9-tetrahydro-2,9a-diazabenzo[*cd*]azulene (2a)
White solid (44.9 mg, 93% yield), m.p. 110-111 °C. **^{1}H NMR** (400 MHz, $CDCl_3$) δ 7.71 (d, J = 8.0 Hz, 1H), 7.24 (d, J = 7.8 Hz, 1H), 7.18 (d, J = 7.2 Hz, 1H), 4.41 – 4.36 (m, 2H), 3.32 – 3.01 (m, 2H), 2.25 – 2.17 (m, 2H), 2.15 – 2.07 (m, 2H). **^{13}C NMR** (100 MHz, $CDCl_3$) δ 142.0, 141.1(q, $^2J_{C-F}$ = 37.6 Hz), 135.7, 127.7, 125.6, 123.4, 119.4 (q, $^1J_{C-F}$ = 269.7 Hz), 119.2, 48.2, 34.0, 28.3, 27.2. **^{19}F NMR** (376 MHz, $CDCl_3$) δ -57.9. **HRMS (ESI)** *m/z:* $[M+H]^+$ Calcd. for $C_{12}H_{12}F_3N_2$ 241.0947; Found 241.0948.

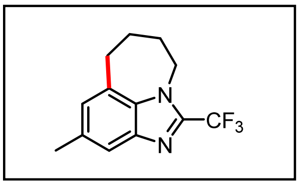

4-Methyl-1-(trifluoromethyl)-6,7,8,9-tetrahydro-2,9a-diazabenzo[*cd*]azulene (2b)

White solid (49.8 mg, 98% yield), m.p. 92-94 °C. **^{1}H NMR** (400 MHz, $CDCl_3$) δ 7.47 (s, 1H), 7.02 (s, 1H), 4.41 – 4.27 (m, 2H), 3.19 – 3.04 (m, 2H), 2.44 (s, 3H), 2.23 – 2.14 (m, 2H), 2.12 – 2.04 (m, 2H). **^{13}C NMR** (100 MHz, $CDCl_3$) δ 142.4, 141.0 (q, $^2J_{C\text{-}F}$ = 37.3 Hz), 133.8, 133.1, 127.4, 127.1, 119.4 (q, $^1J_{C\text{-}F}$ = 268.7 Hz), 118.6, 48.3, 34.1, 28.3, 27.2, 21.4. **^{19}F NMR** (376 MHz, $CDCl_3$) δ -57.9. **HRMS (ESI)** *m/z:* $[M+H]^+$ Calcd. for $C_{13}H_{14}F_3N_2$ 255.1104; Found 255.1106.

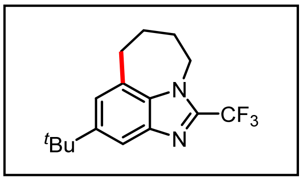

4-(Tert-butyl)-1-(trifluoromethyl)-6,7,8,9-tetrahydro-2,9a-diazabenzo[*cd*]azulene (2c)

White solid (56.9 mg, 96% yield), m.p. 99-101 °C. **^{1}H NMR** (400 MHz, $CDCl_3$) δ 7.72 (s, 1H), 7.26 (s, 1H), 4.41 – 4.23 (m, 2H), 3.26 – 3.10 (m, 2H), 2.25 – 2.16 (m, 2H), 2.14 – 2.05 (m, 2H), 1.37 (s, 9H). **^{13}C NMR** (100 MHz, $CDCl_3$) δ 146.9, 142.2, 141.2 (q, $^2J_{C\text{-}F}$ = 37.5 Hz), 133.6, 126.8, 124.0, 119.4 (q, $^1J_{C\text{-}F}$ = 269.5 Hz), 115.1, 53.5, 48.5, 34.7, 31.7, 28.4, 27.3. **^{19}F NMR** (376 MHz, $CDCl_3$) δ -62.3. **HRMS (ESI)** *m/z:* $[M+H]^+$ Calcd. for $C_{16}H_{20}F_3N_2$ 297.1573; Found 255.1577.

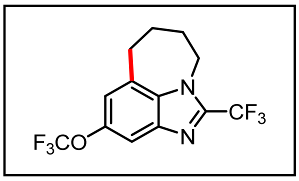

4-(Trifluoromethoxy)-1-(trifluoromethyl)-6,7,8,9-tetrahydro-2,9a-diazabenzo[*cd*]azulene (2d)

White solid (62.9 mg, 97% yield), m.p. 92-94 °C. **^{1}H NMR** (400 MHz, $CDCl_3$) δ 7.57 (s, 1H), 7.08 (s, 1H), 4.43 – 4.37 (m, 2H), 3.21 – 3.15 (m, 2H), 2.27– 2.19 (m, 2H), 2.17 – 2.09 (m, 2H). **^{13}C NMR** (100 MHz, $CDCl_3$) δ 145.3, 142.7 (q, $^2J_{C\text{-}F}$ = 38.3 Hz), 142.2, 134.2, 129.2, 120.7 (q, $^1J_{C\text{-}F}$ = 255.2 Hz), 119.5, 119.1 (q, $^1J_{C\text{-}F}$ = 269.8 Hz), 111.4, 48.5, 34.0, 28.1, 26.9. **^{19}F NMR** (376 MHz, $CDCl_3$) δ -58.1, -62.5. **HRMS (ESI)** *m/z:* $[M+H]^+$ Calcd. for $C_{13}H_{11}F_6N_2O$ 325.0770; Found 325.0774.

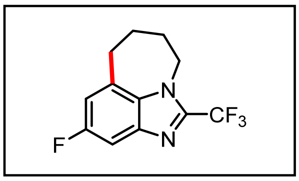

4-Fluoro-1-(trifluoromethyl)-6,7,8,9-tetrahydro-2,9a-diazabenzo[*cd*]azulene (2e)

White solid (50.0 mg, 97% yield), m.p. 73-75 °C. **^{1}H NMR** (400 MHz, $CDCl_3$) δ 7.35 (d, J = 8.8 Hz, 1H), 6.96 (d, J = 10.0 Hz, 1H), 4.44 – 4.32 (m, 2H), 3.20 – 3.10 (m, 2H), 2.30 – 2.21 (m, 2H), 2.20 – 2.11 (m, 2H). **^{13}C NMR** (100 MHz, $CDCl_3$) δ 159.4 (d, $^1J_{C\text{-}F}$ = 239.8 Hz), 142.2 (d, $^2J_{C\text{-}F}$ = 37.9 Hz), 141.5, 132.2, 129.1 (d, $^3J_{C\text{-}F}$ = 9.2 Hz), 119.1 (q, $^1J_{C\text{-}F}$ = 269.9 Hz), 114.3 (d, $^2J_{C\text{-}F}$ = 25.9 Hz), 104.2 (d, $^2J_{C\text{-}F}$ = 23.8 Hz), 48.3, 33.9, 28.1, 26.9. **^{19}F NMR** (376 MHz, $CDCl_3$) δ -58.2, -115.3. **HRMS (ESI)** *m/z:* $[M+H]^+$ Calcd. for $C_{12}H_{11}F_4N_2$ 259.0853; Found 259.0855.

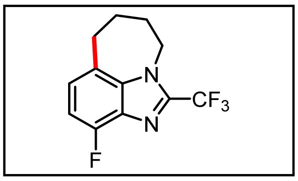

3-Fluoro-1-(trifluoromethyl)-6,7,8,9-tetrahydro-2,9a-diazabenzo[*cd*]azulene (2f)

White solid (44.9 mg, 87% yield), m.p. 89-91 °C. **^{1}H NMR** (400 MHz, $CDCl_3$) δ 7.14 – 7.03 (m, 1H), 6.92 (t, J = 9.2 Hz, 1H), 4.47 – 4.36 (m, 2H), 3.17 – 3.09 (m, 2H), 2.19 – 2.09 (m, 2H), 2.18 – 2.08 (m, 2H). **^{13}C NMR** (100 MHz, $CDCl_3$) δ 153.2 (d, $^1J_{C\text{-}F}$ = 250.9 Hz), 141.3 (q, $^2J_{C\text{-}F}$ = 38.1 Hz), 137.9, 131.0 (d, $^2J_{C\text{-}F}$ = 17.3 Hz), 125.6 (d, $^3J_{C\text{-}F}$ = 6.8 Hz), 123.6 (d, $^3J_{C\text{-}F}$ = 4.8 Hz), 119.1 (q, $^1J_{C\text{-}F}$ = 269.9 Hz), 108.3 (d, $^2J_{C\text{-}F}$ = 16.5 Hz), 48.3, 33.3, 28.2, 27.0. **^{19}F NMR** (376 MHz, $CDCl_3$) δ -62.2, -130.7. **HRMS (ESI)** *m/z:* $[M+H]^+$ Calcd. for $C_{12}H_{11}F_4N_2$ 259.0853; Found 259.0856.

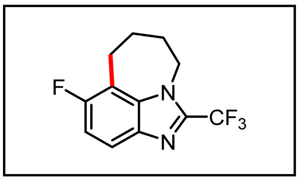

5-Fluoro-1-(trifluoromethyl)-6,7,8,9-tetrahydro-2,9a-diazabenzo[*cd*]azulene (2g)

White solid (50.1 mg, 97% yield), m.p. 108-109 °C. **^{1}H NMR** (400 MHz, $CDCl_3$) δ 7.64 (dd, J = 8.8, 4.4 Hz, 1H), 7.08 (dd, J = 10.0, 9.2 Hz, 1H), 4.47 – 4.26 (m, 2H), 3.25 – 3.03 (m, 2H), 2.19 – 2.09 (m, 2H), 2.18 – 2.08 (m, 2H). **^{13}C NMR** (100 MHz, $CDCl_3$) δ 158.3 (d, $^1J_{C\text{-}F}$ = 239.8 Hz), 141.5 (q, $^2J_{C\text{-}F}$ = 37.8 Hz), 138.2, 136.0 (d, $^3J_{C\text{-}F}$ = 7.7 Hz), 119.7 (d, $^3J_{C\text{-}F}$ = 10.7 Hz), 119.1 (q, $^1J_{C\text{-}F}$ = 269.4 Hz), 113.8 (d, $^2J_{C\text{-}F}$ = 21.7 Hz), 112.2 (d, $^2J_{C\text{-}F}$ = 27.5 Hz), 48.9, 28.0, 26.6, 26.1. **^{19}F NMR** (376 MHz, $CDCl_3$) δ -62.5, -118.1. **HRMS (ESI)** *m/z:* $[M+H]^+$ Calcd. for $C_{12}H_{11}F_4N_2$ 259.0853; Found 259.0858.

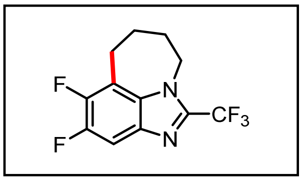

4,5-Difluoro-1-(trifluoromethyl)-6,7,8,9-tetrahydro-2,9a-diazabenzo[*cd*]azulene (2h)

White solid (44.2 mg, 80% yield), m.p. 129-130 °C. **^{1}H NMR** (400 MHz, $CDCl_3$) δ 7.53 – 7.39 (m, 2H), 4.43 – 4.33 (m, 2H), 3.24 – 3.10 (m, 2H), 2.19 – 2.09 (m, 2H), 2.18 – 2.08 (m, 2H). **^{13}C NMR** (100 MHz, $CDCl_3$) δ148.6 (dd, $^1J_{C\text{-}F}$ = 241.9,15.9 Hz), 147.4 (dd, $^1J_{C\text{-}F}$ = 240.4,14.6 Hz), 142.1 (q, $^2J_{C\text{-}F}$ = 38.8 Hz), 136.5 (d, $^3J_{C\text{-}F}$ = 11.2 Hz), 131.6 (d, $^3J_{C\text{-}F}$ = 6.3 Hz), 119.0 (q, $^1J_{C\text{-}F}$ = 269.7 Hz), 115.9 (d, $^2J_{C\text{-}F}$ = 18.1 Hz), 10.5.8 (d, $^2J_{C\text{-}F}$ = 19.4 Hz), 49.0, 28.0, 26.2, 26.5. **^{19}F NMR** (376 MHz, $CDCl_3$) δ -62.5, -140.3, -141.0. **HRMS (ESI)** *m/z:* $[M+H]^+$ Calcd. for $C_{12}H_{10}F_5N_2$ 277.0759; Found 277.0761.

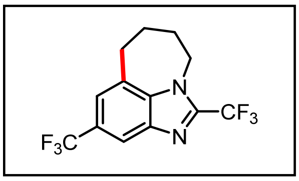

1,4-Bis(trifluoromethyl)-6,7,8,9-tetrahydro-2,9a-diazabenzo[*cd*]azulene (2i)

White solid (57.9 mg, 94% yield), m.p. 75-79 °C. **^{1}H NMR** (400 MHz, $CDCl_3$) δ 7.99 (s, 1H), 7.42 (s, 1H), 4.46 – 4.39 (m, 2H), 3.28 – 3.21 (m, 2H), 2.30 – 2.21 (m, 2H), 2.20 – 2.11 (m, 2H). **^{13}C NMR** (100 MHz, $CDCl_3$) δ 142.9 (q, $^2J_{C\text{-}F}$ = 38.2 Hz), 141.5 , 137.6, 128.5, 127.6 (q, $^2J_{C\text{-}F}$ = 32.3 Hz), 122.1 (q, $^3J_{C\text{-}F}$ = 3.1 Hz), 120.5 (q, $^1J_{C\text{-}F}$ = 270.4 Hz), 119.1 (q, $^1J_{C\text{-}F}$ = 270 Hz), 116.9 (q, $^3J_{C\text{-}F}$ = 4.1 Hz), 48.4, 34.0, 28.1, 26.8. **^{19}F NMR** (376 MHz, $CDCl_3$) δ -62.5,-118.1. **HRMS (ESI)** *m/z:* $[M+H]^+$ Calcd. for $C_{13}H_{11}F_6N_2$ 309.0821; Found 309.0823.

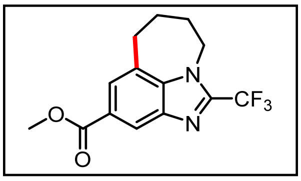

Methyl 1-(trifluoromethyl)-6,7,8,9-tetrahydro-2,9a-diazabenzo[*cd*]azulene-4-carboxylate (2j)
White solid (47.7 mg, 80% yield), m.p. 101-103 °C. **^{1}H NMR** (400 MHz, $CDCl_3$) δ 8.38 (s, 1H), 7.88 (s, 1H), 4.47 – 4.36 (m, 2H), 3.95 (s, 3H), 3.26 – 3.16 (m, 2H), 2.30– 2.19 (m, 2H), 2.17 – 2.06 (m, 2H). **^{13}C NMR** (100 MHz, $CDCl_3$) δ 167.2, 142.7 (q, $^{2}J_{C\text{-}F}$ = 38.2 Hz), 141.7, 138.7, 127.7, 126.5, 125.6, 121.5, 119.1 (q, $^{1}J_{C\text{-}F}$ = 270 Hz), 52.3, 48.4, 33.9, 28.1, 26.9. **^{19}F NMR** (376 MHz, $CDCl_3$) δ -58.2. **HRMS (ESI)** *m/z:* $[M+H]^+$ Calcd. for $C_{14}H_{14}F_3N_2O_2$ 299.1002; Found 299.1003.

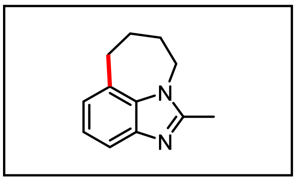

1-Methyl-6,7,8,9-tetrahydro-2,9a-diazabenzo[*cd*]azulene (2k)
White solid (27.2 mg, 79% yield), m.p. 126-128 °C. **^{1}H NMR** (400 MHz, $CDCl_3$) δ 7.52 (d, *J* = 8.0 Hz, 1H), 7.11 (t, *J* = 7.6 Hz, 1H), 7.00 (d, *J* = 7.2 Hz, 1H), 4.17 – 4.05 (m, 2H), 3.23 – 3.07 (m, 2H), 2.59 (s, 3H), 2.19 – 2.11 (m, 2H), 2.11 – 2.02 (m, 2H). **^{13}C NMR** (100 MHz, $CDCl_3$) δ 152.2, 143.3, 134.9, 125.9, 122.7, 121.6, 116.5, 48.1, 34.9, 28.6, 27.5, 14.7. **HRMS (ESI)** *m/z:* $[M+H]^+$ Calcd. for $C_{12}H_{15}N_2$ 187.1230; Found 187.1233.

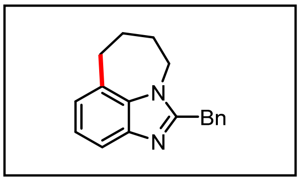

1-Benzyl-6,7,8,9-tetrahydro-2,9a-diazabenzo[*cd*]azulene (2l)
White solid (38.3 mg, 73% yield), m.p. 66-68 °C. **^{1}H NMR** (400 MHz, $CDCl_3$) δ 7.61 (d, *J* = 8.0 Hz, 1H), 7.32 – 7.25 (m, 1H), 7.21 (t, *J* = 6.8 Hz, 1H), 7.14 (t, *J* = 7.6 Hz, 1H), 7.01 (d, *J* = 7.2 Hz, 1H), 4.32 (s, 2H), 4.00 – 3.93 (m, 2H), 3.14 – 3.08 (m, 2H), 2.01 – 1.94 (m, 4H). **^{13}C NMR** (100 MHz, $CDCl_3$) δ 153.5, 143.4, 136.4, 135.3, 128.9, 128.5, 126.9, 126.3, 123.0, 121.7, 117.0, 48.0, 35.1, 34.5, 28.4, 27.3. **HRMS (ESI)** *m/z:* $[M+H]^+$ Calcd. for $C_{18}H_{19}N_2$ 263.1543; Found 263.1547.

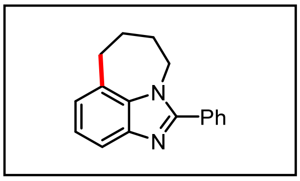

1-Phenyl-6,7,8,9-tetrahydro-2,9a-diazabenzo[*cd*]azulene (2m)
White solid (37.7 mg, 76% yield), m.p. 96-98 °C. **^{1}H NMR** (400 MHz, $CDCl_3$) δ 7.70 (dd, *J* = 7.6, 2.0 Hz, 2H), 7.65 (d, *J* = 8.0 Hz, 1H), 7.54 – 7.46 (m, 3H), 7.17 (t, *J* = 7.6 Hz, 1H), 7.03 (d, *J* = 6.8 Hz, 1H), 4.00 – 3.89 (m, 2H), 3.19 – 2.97 (m, 2H), 2.22 – 2.00 (m, 4H). **^{13}C NMR** (100 MHz, $CDCl_3$) δ 154.7, 143.6, 136.3, 130.7, 129.7, 129.6, 128.7, 127.1, 123.0, 122.4, 117.5, 48.1, 32.6, 28.4, 27.2. **HRMS**

(ESI) *m/z:* $[M+H]^+$ Calcd. for $C_{17}H_{17}N_2$ 249.1386; Found 249.1388.

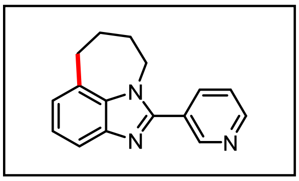

1-(Pyridin-3-yl)-6,7,8,9-tetrahydro-2,9a-diazabenzo[*cd*]azulene (2n)

White solid (22.4 mg, 46% yield), m.p. 58-60 °C. **^{1}H NMR** (400 MHz, $CDCl_3$) δ 8.94 (s, 1H), 8.73 (s, 1H), 8.08 (d, *J* = 6.4 Hz, 1H), 7.66 (d, *J* = 8.0 Hz, 1H), 7.52 – 7.42 (m, 1H), 7.25 – 7.17 (m, 1H), 7.09 (t, *J* = 6.8 Hz, 1H), 4.32 – 4.20 (m, 2H), 3.28 – 3.14 (m, 2H), 2.27 – 2.08 (m, 4H). **^{13}C NMR** (100 MHz, $CDCl_3$) δ 151.5, 150.6, 150.0, 143.7, 137.2, 136.3, 127.3, 127.0, 123.7, 123.6, 122.8, 117.7, 48.2, 32.7, 28.4, 27.1. **HRMS (ESI)** *m/z:* $[M+H]^+$ Calcd. for $C_{13}H_{11}F_6N_2$ 250.1339; Found 250.1340.

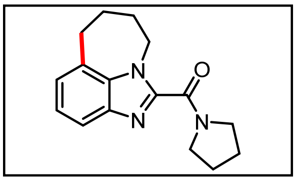

Pyrrolidin-1-yl(6,7,8,9-tetrahydro-2,9a-diazabenzo[*cd*]azulen-1-yl)methanone (2o)

White solid (33.9 mg, 63% yield), m.p. 127-129 °C. **^{1}H NMR** (400 MHz, $CDCl_3$) δ 7.61 (d, *J* = 8.0 Hz, 1H), 7.18 (t, *J* = 7.6 Hz, 1H), 7.09 (d, *J* = 7.2 Hz, 1H), 4.63 – 4.46 (m, 2H), 3.87 (t, *J* = 6.4 Hz, 2H), 3.69 (t, *J* = 6.4 Hz, 2H), 3.22 – 3.10 (m, 2H), 2.22 – 2.03 (m, 4H), 2.02 – 1.87 (m, 4H). **^{13}C NMR** (100 MHz, $CDCl_3$) δ 160.0, 146.6, 142.6, 135.0, 127.6, 124.3, 122.7, 118.2, 49.2, 48.2, 46.5, 33.7, 28.4, 27.3, 26.3, 24.2. **HRMS (ESI)** *m/z:* $[M+H]^+$ Calcd. for $C_{16}H_{20}N_3O$ 270.1606; Found 270.1605.

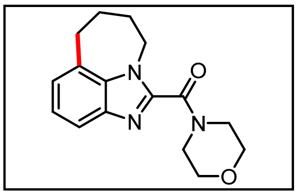

Morpholino(6,7,8,9-tetrahydro-2,9a-diazabenzo[*cd*]azulen-1-yl)methanone (2p)

White solid (40.0 mg, 72% yield), m.p. 59-61 °C. **^{1}H NMR** (400 MHz, $CDCl_3$) δ 7.62 (d, *J* = 8.0 Hz, 1H), 7.20 (t, *J* = 7.6 Hz, 1H), 7.11 (d, *J* = 7.2 Hz, 1H), 4.46 – 4.30 (m, 2H), 3.88 – 3.79 (m, 4H), 3.78 – 3.71 (m, 2H), 3.23 – 3.10 (m, 2H), 2.22 – 2.00 (m, 4H). **^{13}C NMR** (100 MHz, $CDCl_3$) δ 160.7, 145.3, 142.6, 134.8, 127.5, 124.5, 122.8, 118.2, 67.3, 66.8, 48. 4, 47.9, 42.7, 33.9, 28.3, 27.3. **HRMS (ESI)** *m/z:* $[M+H]^+$ Calcd. for $C_{16}H_{20}N_3O_2$ 286.1556; Found 286.1550.

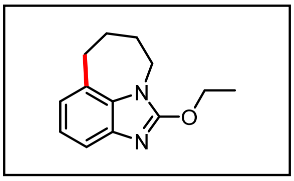

1-Ethoxy-6,7,8,9-tetrahydro-2,9a-diazabenzo[*cd*]azulene (2q)

White solid (21.6 mg, 65% yield), m.p. 60-61 °C. **^{1}H NMR** (400 MHz, $CDCl_3$) δ 7.39 (d, *J* = 7.6 Hz, 1H), 7.06 (t, *J* = 7.6 Hz, 1H), 6.91 (d, *J* = 7.2 Hz, 1H), 4.58 (q, *J* = 6.8 Hz, 2H), 4.00 – 3.89 (m, 2H), 3.19 – 2.97 (m, 2H), 2.14 – 1.88 (m, 4H), 1.48 (t, *J* = 7.2 Hz, 3H). **^{13}C NMR** (100 MHz, $CDCl_3$) δ 157.8, 140.9,

133.4, 125.1, 121.8, 121.2, 115.1, 66.1, 46.5, 35.2, 28.4, 27.8, 14.9. **HRMS (ESI)** *m/z:* $[M+H]^+$ Calcd. for $C_{13}H_{17}N_2O$ 217.1341; Found 217.1334.

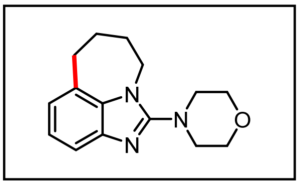

1-(Pyrrolidin-1-yl)-6,7,8,9-tetrahydro-2,9a-diazabenzo[*cd*]azulene (2r)

White solid (20.6 mg, 40% yield), m.p. 71-74 °C. **^{1}H NMR** (400 MHz, $CDCl_3$) δ 7.44 (d, *J* = 8.0 Hz, 1H), 7.07 (t, *J* = 7.8 Hz, 1H), 6.91 (d, *J* = 7.2 Hz, 1H), 4.00 – 3.94 (m, 2H), 3.96 – 3.81 (m, 4H), 3.42 – 3.23 (m, 4H), 3.14 – 3.00 (m, 2H), 2.14 – 2.00 (m, 4H), 1.25 – 1.21 (m, 2H). **^{13}C NMR** (100 MHz, $CDCl_3$) δ 158.8, 141.9, 135.6, 126.1, 121.9, 115.8, 66.7, 51.0, 47.5, 32.7, 29.8, 28.2, 27.4. **HRMS (ESI)** *m/z:* $[M+H]^+$ Calcd. for $C_{13}H_{17}N_2O$ 258.1601; Found 258.1607.

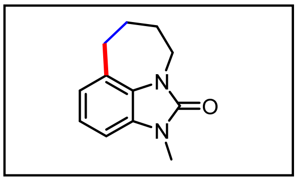

2-Methyl-6,7,8,9-tetrahydro-2,9a-diazabenzo[*cd*]azulen-1(2H)-one (2s)

White solid (34.4 mg, 85% yield), m.p. 95-96 °C. **^{1}H NMR** (400 MHz, $CDCl_3$) δ 7.00 (t, *J* = 7.6 Hz, 1H), 6.87 (d, *J* = 7.6, 1H), 6.82 (d, *J* = 8.0 Hz, 1H), 3.98 – 3.95 (m, 2H), 3.41 (s, 3H), 3.07 – 2.95 (m, 2H), 2.11 – 1.89 (m, 4H). **^{13}C NMR** (100 MHz, $CDCl_3$) δ 155.5, 130.7, 128.7, 124.6, 122.6, 120.9, 104.9, 45.6, 34.4, 28.0, 27.5, 27.2. **HRMS (ESI)** *m/z:* $[M+H]^+$ Calcd. for $C_{12}H_{14}N_2NaO$ 225.0998; Found 225.0994.

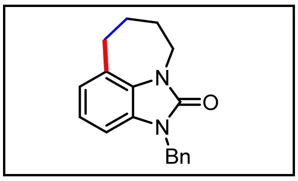

2-Benzyl-6,7,8,9-tetrahydro-2,9a-diazabenzo[*cd*]azulen-1(2*H*)-one (2t)

White solid (50.1 mg, 90% yield), m.p. 83-85 °C. **^{1}H NMR** (400 MHz, $CDCl_3$) δ 7.29 – 7.26 (m, 4H), 7.25 – 7.19 (m, 1H), 6.88 (t, *J* = 7.6 Hz, 1H), 6.82 (d, *J* = 7.6 Hz, 1H), 6.71 (d, *J* = 7.6 Hz, 1H), 5.04 (s, 2H), 4.15 – 3.83 (m, 2H), 3.10 – 2.86 (m, 2H), 2.21 – 1.82 (m, 4H). **^{13}C NMR** (100 MHz, $CDCl_3$) δ 155.0, 136.5, 129.8, 128.8, 128.7, 127.6, 127.5, 124.7, 122.7, 121.0, 105.8, 45.6, 44.9, 34.4, 28.0, 27.4. **HRMS (ESI)** *m/z:* $[M+Na]^+$ Calcd. for $C_{18}H_{18}N_2NaO$ 301.1311; Found 301.1315.

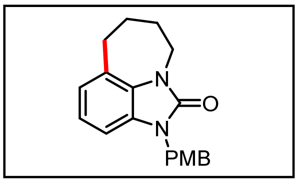

2-(4-Methoxybenzyl)-6,7,8,9-tetrahydro-2,9a-diazabenzo[*cd*]azulen-1(2*H*)-one (2u)

White solid (39.5 mg, 64% yield), m.p. 68-69 °C. **^{1}H NMR** (400 MHz, $CDCl_3$) δ 7.28 (s, 1H), 7.26 (s, 1H), 6.90 (t, *J* = 7.6 Hz, 1H), 6.83 (d, *J* = 8.2 Hz, 3H), 6.75 (d, *J* = 7.6 Hz, 1H), 5.00 (s, 2H), 4.07 – 3.95 (m, 2H),

3.76 (s, 3H), 3.10 – 2.89 (m, 2H), 2.06 – 1.82 (m, 4H). **^{13}C NMR** (100 MHz, $CDCl_3$) *δ* 159.2, 155.0, 129.9, 129.0, 128.9, 128.7, 124.8, 122.7, 121.0, 114.2, 105.9, 55.4, 45.7, 44.5, 34.5, 28.1, 27.6. **HRMS (ESI)** *m/z:* $[M+Na]^+$ Calcd. for $C_{19}H_{20}N_2NaO_2$ 331.1417; Found 331.1422.

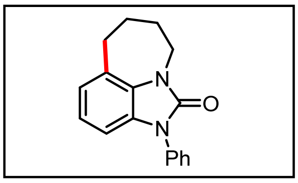

2-Phenyl-6,7,8,9-tetrahydro-2,9a-diazabenzo[*cd*]azulen-1(2*H*)-one (2v)

White solid (42.2 mg, 80% yield), m.p. 96-97 °C. **^{1}H NMR** (400 MHz, $CDCl_3$) *δ* 7.60 – 7.46 (m, 4H), 7.41 – 7.36 (m, 1H), 7.05 – 6.86 (m, 3H), 4.14 – 3.95 (m, 2H), 3.14 – 3.00 (m, 2H), 2.06 – 1.82 (m, 4H). **^{13}C NMR** (100 MHz, $CDCl_3$) *δ* = 159.2, 135.0, 130.2, 129.6, 129.0, 127.8, 126.4, 125.1, 123.4, 121.2, 106.4, 45.5, 34.2, 28.0, 27.5. **HRMS (ESI)** *m/z:* $[M+Na]^+$ Calcd. for $C_{17}H_{16}N_2NaO$ 287.1155; Found 287.1160.

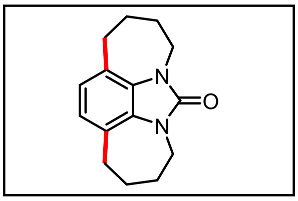

Tetracyclic 1*H*-benzo[d]imidazol-2(3H)-one (2w)

White solid (45.2 mg, 88% yield), m.p. 77-78 °C. **^{1}H NMR** (400 MHz, $CDCl_3$) *δ* 6.74 (s, 2H), 4.07 – 3.95 (m, 4H), 3.10 – 2.89 (m, 4H), 2.06 – 1.82 (m, 8H). **^{13}C NMR** (100 MHz, $CDCl_3$) *δ* 155.5, 129.4, 122.4, 122.3, 44.8, 33.4, 28.0, 27.4. **HRMS (ESI)** *m/z:* $[M+Na]^+$ Calcd. for $C_{16}H_{21}N_2NaO$ 280.1546; Found 280.1551.

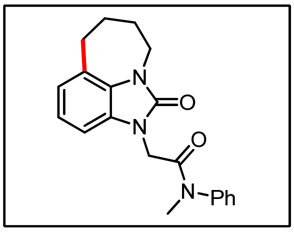

***N*-Methyl-2-(1-oxo-6,7,8,9-tetrahydro-2,9a-diazabenzo[*cd*]azulen-2(1*H*)-yl)-N-phenylacetamide(2x)**

White solid. m.p. 188-189 °C. **^{1}H NMR** (400 MHz, $CDCl_3$) *δ* 7.48 (t, *J* = 7.6 Hz, 2H), 7.41 (d, *J* = 7.2 Hz, 1H), 7.33 (d, *J* = 7.6 Hz, 2H), 6.95 (t, *J* = 7.6 Hz, 1H), 6.83 (d, *J* = 7.6 Hz, 1H), 6.69 (d, *J* = 7.6 Hz, 1H), 4.38 (s, 2H), 3.95 – 3.75 (m, 2H), 3.30 (s, 3H), 3.12 – 2.93 (m, 2H), 2.02 – 1.91 (m, 4H). **^{13}C NMR** (100 MHz, $CDCl_3$) *δ* 166.6, 154.8, 130.3, 130.2, 128.8, 128.6, 127.3, 124.8, 122.9, 121.1, 105.3, 45.6, 43.2, 37.9, 34.5, 28.0, 27.5. **HRMS (ESI)** *m/z:* $[M+H]^+$ Calcd. for $C_{18}H_{19}N_2$ 263.1543; Found 263.1546. **HRMS (ESI)** *m/z:* $[M+Na]^+$ Calcd. for $C_{20}H_{21}N_3NaO_2$ 358.1526; Found 358.1530.

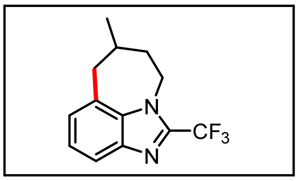

7-Methyl-1-(trifluoromethyl)-6,7,8,9-tetrahydro-2,9a-diazabenzo[*cd*]azulene (3a)

White solid (48.8 mg, 96% yield), m.p. 59-61 °C. **^{1}H NMR** (400 MHz, $CDCl_3$) δ 7.70 (d, J = 8.0 Hz, 1H), 7.24 (t, J = 7.6 Hz, 1H), 7.15 (d, J = 7.2 Hz, 1H), 4.62 – 4.54 (m, 1H), 4.27 – 4.12 (m, 1H), 3.19 (dd, J = 16.0, 3.2Hz, 1H), 2.93 (dd, J = 16.0, 10.0 Hz, 1H), 2.40 – 2.15 (m, 2H), 2.03 – 1.82 (m, 1H), 1.18 (d, J = 6.8 Hz, 3H). **^{13}C NMR** (100 MHz, $CDCl_3$) δ 141.8, 141.0 (q, $^2J_{C\text{-}F}$ = 38.0 Hz), 135.4, 126.3, 125.8, 123.5, 119.3 (q, $^1J_{C\text{-}F}$ = 269.6 Hz), 119.1, 46.1, 41.5, 36.0, 33.2, 23.1. **^{19}F NMR** (376 MHz, $CDCl_3$) δ -62.2. **HRMS (ESI)** *m/z:* $[M+H]^+$ Calcd. for $C_{13}H_{14}F_3N_2$ 255.1104; Found 255.1106.

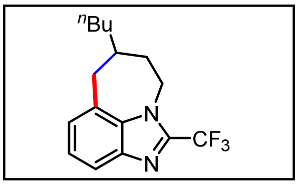

7-Butyl-1-(trifluoromethyl)-6,7,8,9-tetrahydro-2,9a-diazabenzo[*cd*]azulene (3b)

White solid (53.3 mg, 90% yield), 57-58 °C. **^{1}H NMR** (400 MHz, $CDCl_3$) δ 7.69 (d, J = 8.0 Hz, 1H), 7.23 (t, J = 7.6 Hz, 1H), 7.14 (d, J = 7.2 Hz, 1H), 4.60 – 4.54 (m, 1H) , 4.26 – 4.14 (m, 1H), 3.20 (dd, J = 16.0, 3.2 Hz, 1H), 2.94 (dd, J = 16.0, 9.6 Hz, 1H), 2.38 – 2.22 (m, 1H), 2.11 – 2.01 (m, 1H), 1.97 – 1.79 (m, 1H), 1.56 – 1.24 (m, 7H), 0.92 (t, J = 7.2 Hz, 3H). **^{13}C NMR** (100 MHz, $CDCl_3$) δ 141.8, 141.2 (q, $^2J_{C\text{-}F}$ = 38.2 Hz), 135.5, 126.4, 125.8, 123.5, 119.4 (q, $^1J_{C\text{-}F}$ = 269.6 Hz), 119.2, 46.1, 39.3, 38.0, 36.6, 34.3, 29.4, 22.9, 14.2. **^{19}F NMR** (376 MHz, $CDCl_3$) δ -62.2. **HRMS (ESI)** *m/z:* $[M+H]^+$ Calcd. for $C_{16}H_{20}F_3N_2^+$: 297.1573; Found 297.1578.

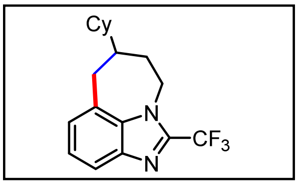

7-Cyclohexyl-1-(trifluoromethyl)-6,7,8,9-tetrahydro-2,9a-diazabenzo[*cd*]azulene (3c)

White solid (55.4 mg, 86% yield), m.p. 69-71 °C. **^{1}H NMR** (400 MHz, $CDCl_3$) δ 7.69 (d, J = 8.0 Hz, 1H), 7.23 (t, J = 7.6 Hz, 1H), 7.16 (d, J = 7.2 Hz, 1H), 4.61 – 4.54 (m, 1H) , 4.26 – 4.11 (m, 1H), 3.18 – 3.05 (m, 2H), 2.31 – 2.25 (m, 1H), 1.97 – 1.88 (m, 2H), 1.80 – 1.69 (m, 5H), 1.44 – 1.39 (m, 1H), 1.34 – 1.03 (m, 5H). **^{13}C NMR** (100 MHz, $CDCl_3$) δ 141.8, 140.4 (q, $^2J_{C\text{-}F}$ = 37.7 Hz), 135.5, 126.9, 125.7, 123.5, 119.4 (q, $^1J_{C\text{-}F}$ = 269.7 Hz), 119.1, 46.5, 43.7, 43.6, 36.7, 31.9, 30.0, 29.8, 26.7, 26.6. **^{19}F NMR** (376 MHz, $CDCl_3$) δ -62.2. **HRMS (ESI)** *m/z:* $[M+H]^+$ Calcd. for $C_{18}H_{22}F_3N_2$ 323.1730; Found 323.1733.

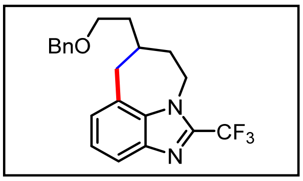

7-(2-(Benzyloxy)ethyl)-1-(trifluoromethyl)-6,7,8,9-tetrahydro-2,9a-diazabenzo[*cd*]azulene (3d)

Colorless oil (61.4 mg, 82% yield). **^{1}H NMR** (400 MHz, $CDCl_3$) δ 7.69 (d, J = 8.0 Hz, 1H), 7.39 – 7.27 (m, 5H), 7.23 (dd, J = 13.2, 5.6 Hz, 1H), 7.11 (d, J = 7.2 Hz, 1H), 4.60 – 4.53 (m, 1H), 4.52 (s, 2H), 4.28 – 4.16 (m, 1H), 3.59 (td, J = 6.0, 1.6 Hz, 2H), 3.22 (dd, J = 16.0, 3.2 Hz, 1H), 2.94 (dd, J = 16.0, 9.2 Hz, 1H), 2.41 – 2.20 (m, 2H), 2.03 – 1.85 (m, 1H), 1.83 – 1.63 (m, 2H). **^{13}C NMR** (100 MHz, $CDCl_3$) δ 141.8, 140.9 (q, $^2J_{C\text{-}F}$ = 37.8 Hz), 138.4, 135.5, 128.6, 127.84, 127.82, 126.0, 125.9, 123.6, 119.3, 119.0 (q, $^1J_{C\text{-}F}$ = 269.7 Hz), 73.2, 67.8, 46.0, 39.1, 36.5, 34.9, 34.2. **^{19}F NMR** (376 MHz, $CDCl_3$) δ -62.2. **HRMS (ESI)** *m/z:*

[M+H]$^+$ Calcd. for $C_{21}H_{22}F_3N_2O$ 375.1679; Found 375.1684.

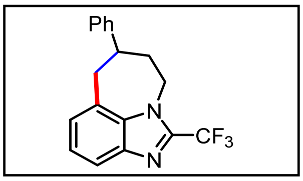

7-Phenyl-1-(trifluoromethyl)-6,7,8,9-tetrahydro-2,9a-diazabenzo[*cd*]azulene (3e)

White solid (54.4 mg, 86% yield), m.p. 105-106 °C. **^{1}H NMR** (400 MHz, $CDCl_3$) δ 7.75 (d, J = 8.0 Hz, 1H), 7.41 – 7.33 (m, 2H), 7.30 – 7.25 (m, 4H), 7.17 (d, J = 7.2 Hz, 1H), 4.79 – 4.64 (m, 1H), 4.33 – 4.15 (m, 1H), 3.54 – 3.25 (m, 3H), 2.69 – 2.50 (m, 1H), 2.52 – 2.25 (m, 1H). **^{13}C NMR** (100 MHz, $CDCl_3$) δ 146.5, 142.1, 141.2 (d, $^2J_{C\text{-}F}$ = 38.1 Hz), 135.4, 129.0, 126.9, 126.7, 126.0, 125.9, 123.7, 119.5, 119.3 (q, $^1J_{C\text{-}F}$ = 269.9 Hz), 46.7, 44.8, 41.8, 36.0. **^{19}F NMR** (376 MHz, $CDCl_3$) δ -69.4. **HRMS (ESI)** *m/z:* [M+H]$^+$ Calcd. for $C_{18}H_{16}F_3N_2$ 317.1260; Found 317.1263.

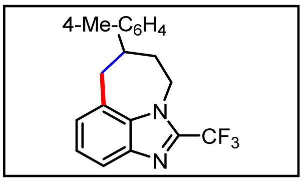

7-(*p*-Tolyl)-1-(trifluoromethyl)-6,7,8,9-tetrahydro-2,9a-diazabenzo[*cd*]azulene (3f)

White solid (59.4 mg, 90% yield), m.p. 88-89 °C. ^{1}H NMR (400 MHz, $CDCl_3$) δ 7.78 (d, J = 8.0 Hz, 1H), 7.24 (t, J = 7.6 Hz, 1H), 7.24 – 7.15 (m, 5H), 4.77 – 4.67 (m, 1H), 4.39 – 4.14 (m, 1H), 3.53 – 3.36 (m, 2H), 3.34 – 3.25 (m, 1H), 2.68 – 2.51 (m, 1H), 2.48 – 2.41 (m, 1H), 2.40 (s, 3H). **^{13}C NMR** (100 MHz, $CDCl_3$) δ 143.5, 142.0, 141.11 (q, $^2J_{C\text{-}F}$ = 37.9 Hz), 136.4, 135.3, 129.6, 126.5, 126.1, 125.9, 123.6, 119.4, 119.2 (q, $^1J_{C\text{-}F}$ = 269.5 Hz), 46.6, 44.5, 41.9, 36.0, 21.1. **^{19}F NMR** (376 MHz, $CDCl_3$) δ -62.3. **HRMS (ESI)** *m/z:* [M+H]$^+$ Calcd. for $C_{19}H_{18}F_3N_2$ 331.1417; Found 331.1417.

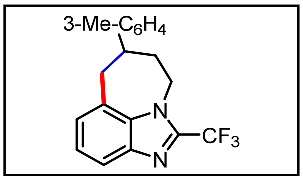

7-(*m*-Tolyl)-1-(trifluoromethyl)-6,7,8,9-tetrahydro-2,9a-diazabenzo[*cd*]azulene (3g)

White solid (56.8 mg, 86% yield), m.p. 99-101 °C. **^{1}H NMR** (400 MHz, $CDCl_3$) δ 7.78 (d, J = 8.0 Hz, 1H), 7.34 – 7.24 (m, 2H), 7.19 (d, J = 7.2 Hz, 1H), 7.15 – 7.08 (m, 3H), 4.73 – 4.61 (m, 1H), 4.31 – 4.24 (m, 1H), 3.50 – 3.37 (m, 2H), 3.32 – 3.26 (m, 1H), 2.65 – 2.54 (m, 1H), 2.51 – 2.41 (m, 1H), 2.40 (s, 3H). **^{13}C NMR** (100 MHz, $CDCl_3$) δ 146.5, 142.0, 141.2 (q, $^2J_{C\text{-}F}$ = 38.1 Hz), 138.7, 135.4, 128.9, 127.6, 127.5, 126.1, 125.9, 123.6, 119.5, 119.3 (q, $^1J_{C\text{-}F}$ = 269.7 Hz), 46.8, 45.0, 41.9, 36.1, 21.6. **^{19}F NMR** (376 MHz, $CDCl_3$) δ -62.1. **HRMS (ESI)** *m/z:* [M+H]$^+$ Calcd. for $C_{19}H_{18}F_3N_2$ 331.1417; Found 331.1422.

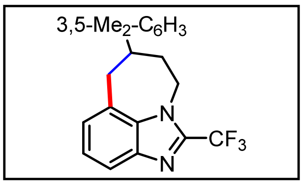

7-(3,5-Dimethylphenyl)-1-(trifluoromethyl)-6,7,8,9-tetrahydro-2,9a-diazabenzo[*cd*]azulene (3h)
White solid (55.1 mg, 80% yield), m.p. 121-122 °C. **^{1}H NMR** (400 MHz, $CDCl_3$) δ 7.74 (d, J = 8.0 Hz, 1H), 7.26 (t, J = 8.0 Hz, 1H), 7.15 (d, J = 7.2 Hz, 1H), 6.91 (s, 1H), 6.88 (s, 2H), 4.76 – 4.68 (m, 1H), 4.30 – 4.15 (m, 1H), 3.47 – 3.31 (m, 2H), 3.25 – 3.17 (m, 1H), 2.62 – 2.50 (m, 1H), 2.46 – 2.36 (m, 1H), 2.32 (s, 6H). **^{13}C NMR** (100 MHz, $CDCl_3$) δ 146.6, 142.1, 141.2 (q, $^2J_{C\text{-}F}$ = 38.1 Hz), 138.6, 135.4, 128.5, 126.2, 125.9, 124.5, 123.6, 119.5, 119.3 (q, $^1J_{C\text{-}F}$ = 269.7 Hz), 46.8, 45.0, 42.0, 36.1, 21.5. **^{19}F NMR** (376 MHz, $CDCl_3$) δ -62.1. **HRMS (ESI)** *m/z:* $[M+H]^+$ Calcd. for $C_{24}H_{20}F_4N_2$ 345.1573; Found 345.1579.

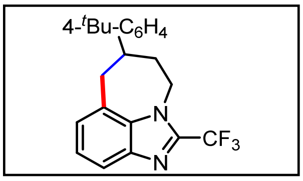

7-(4-(*tert*-Butyl)phenyl)-1-(trifluoromethyl)-6,7,8,9-tetrahydro-2,9a-diazabenzo[*cd*]azulene (3i)
White solid (58.8 mg, 79% yield), m.p. 118-119 °C. **^{1}H NMR** (400 MHz, $CDCl_3$) δ 7.74 (d, J = 8.0 Hz, 1H), 7.41 – 7.36 (m, 2H), 7.27 (d, J = 7.4 Hz, 1H), 7.20 (d, J = 8.3 Hz, 2H), 7.16 (d, J = 7.2 Hz, 1H), 4.73 – 4.61 (m, 1H), 4.33 – 4.19 (m, 1H), 3.54 – 3.34 (m, 2H), 3.32 – 3.25 (m, 1H), 2.65 – 2.52 (m, 1H), 2.50 – 2.35 (m, 1H), 1.33 (s, 9H). **^{13}C NMR** (100 MHz, $CDCl_3$) δ 149.8, 143.4, 142.0, 141.2 (q, $^2J_{C\text{-}F}$ = 38.1 Hz), 135.4, 126.4, 126.2, 125.9, 125.9, 123.7, 119.5, 119.4 (q, $^1J_{C\text{-}F}$ = 269.7 Hz), 46.7, 44.4, 41.9, 36.1, 34.6, 31.5. **^{19}F NMR** (376 MHz, $CDCl_3$) δ -62.1. **HRMS (ESI)** *m/z:* $[M+H]^+$ Calcd. for $C_{24}H_{20}F_4N_2$ 373.1886; Found 373.1892.

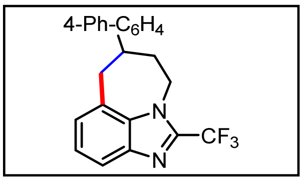

7-([1,1'-Biphenyl]-4-yl)-1-(trifluoromethyl)-6,7,8,9-tetrahydro-2,9a-diazabenzo[*cd*]azulene (3j)
White solid (61.2 mg, 78% yield), m.p. 159-160 °C. **^{1}H NMR** (400 MHz, $CDCl_3$) δ 7.76 (d, J = 8.0 Hz, 1H), 7.61 – 7.56 (m, 4H), 7.45 (t, J = 7.6 Hz, 2H), 7.35 (t, J = 8.0 Hz, 3H), 7.28 (t, J = 7.6 Hz, 1H), 7.19 (d, J = 7.2 Hz, 1H), 4.81 – 4.66 (m, 1H), 4.37 – 4.22 (m, 1H), 3.55 – 3.28 (m, 3H), 2.70 – 2.56 (m, 1H), 2.51 – 2.41 (m, 1H). **^{13}C NMR** (100 MHz, $CDCl_3$) δ 145.5, 142.1, 141.2 (q, $^2J_{C\text{-}F}$ = 38.1 Hz), 140.8, 139.8, 135.4, 128.9, 127.7, 127.4, 127.1, 127.1, 126.0, 125.9, 123.7, 119.5, 119.4 (q, $^1J_{C\text{-}F}$ = 269.7 Hz), 46.6, 44.6, 41.8, 36.0. **^{19}F NMR** (376 MHz, $CDCl_3$) δ -62.1. **HRMS (ESI)** *m/z:* $[M+H]^+$ Calcd. for $C_{24}H_{20}F_4N_2$ 393.1573; Found 393.1572.

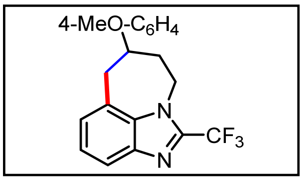

7-(4-Methoxyphenyl)-1-(trifluoromethyl)-6,7,8,9-tetrahydro-2,9a-diazabenzo[*cd*]azulene (3k)
White solid (34.6 mg, 50% yield), m.p. 119-120 °C. **^{1}H NMR** (400 MHz, $CDCl_3$) δ 7.72 (d, J = 8.0 Hz, 1H), 7.24 (t, J = 7.6 Hz, 1H), 7.19 – 7.10 (m, 3H), 6.88 (d, J = 7.6 Hz, 2H), 4.70 – 4.60 (m, 1H), 4.21 (t, J = 11.8 Hz, 1H), 3.80 (s, 3H), 3.46 – 3.28 (m, 2H), 3.29 – 3.19 (m, 1H), 2.64 – 2.44 (m, 1H), 2.42 –

2.25 (m, 1H). **^{13}C NMR** (100 MHz, CDCl$_3$) δ 158.4, 142.0, 141.1 (q, $^2J_{C\text{-}F}$ = 38.2 Hz), 138.6, 135.3, 127.6, 126.0, 125.9, 123.6, 119.4, 119.3 (q, $^1J_{C\text{-}F}$ = 269.7 Hz), 114.2, 55.3, 46.5, 44.0, 41.9, 36.2. **^{19}F NMR** (376 MHz, CDCl$_3$) δ -57.9. **HRMS (ESI)** *m/z:* [M+H]$^+$ Calcd. for $C_{19}H_{18}F_3N_2O$ 347.1366; Found 347.1366.

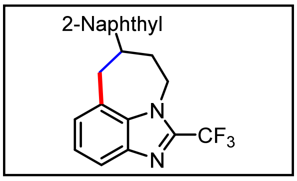

7-(Naphthalen-2-yl)-1-(trifluoromethyl)-6,7,8,9-tetrahydro-2,9a-diazabenzo[*cd*]azulene (3l)

White solid (57.2 mg, 78% yield), m.p. 59-61 °C. **^{1}H NMR** (400 MHz, CDCl$_3$) δ 7.88 – 7.74 (m, 4H), 7.68 (s, 1H), 7.53 – 7.42 (m, 2H), 7.38 (dd, *J* = 8.4, 1.6 Hz, 1H), 7.31 – 7.23 (m, 1H), 7.16 (d, *J* = 7.2 Hz, 1H), 4.73 – 4.61 (m, 1H), 4.37 – 4.19 (m, 1H), 3.67 – 3.26 (m, 3H), 2.75 – 2.55 (m, 1H), 2.54 – 2.31 (m, 1H). **^{13}C NMR** (100 MHz, CDCl$_3$) δ 143.8, 142.1, 141.2 (q, $^2J_{C\text{-}F}$ = 38.2 Hz), 135.4, 133.7, 132.5, 128.8, 127.8, 126.5, 126.0, 125.9, 125.3, 124.9, 123.7, 119.6, 119.3 (q, $^1J_{C\text{-}F}$ = 269.7 Hz), 46.7, 45.0, 41.7, 36.0. **^{19}F NMR** (376 MHz, CDCl$_3$) δ -62.1. **HRMS (ESI)** *m/z:* [M+H]$^+$ Calcd. for $C_{18}H_{15}F_4N_2$ 367.1417; Found 367.1414.

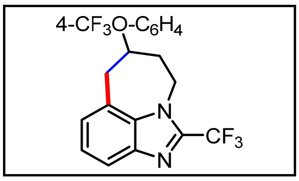

7-(4-(Trifluoromethoxy)phenyl)-1-(trifluoromethyl)-6,7,8,9-tetrahydro-2,9a-diazabenzo[*cd*]azulene (3m)

White solid (67.2 mg, 84% yield), 109-110 °C. **^{1}H NMR** (400 MHz, CDCl$_3$) δ 7.76 (d, *J* = 8.1 Hz, 1H), 7.31 – 7.26 (m, 3H), 7.21 – 7.16 (m, 3H), 4.74 – 4.64 (m, 1H), 4.42 – 4.15 (m, 1H), 3.53 – 3.25 (m, 3H), 2.69 – 2.52 (m, 1H), 2.50 – 2.21 (m, 1H). **^{13}C NMR (100 MHz, CDCl$_3$)** δ 148.1, 145.1, 142.1, 141.3 (q, $^2J_{C\text{-}F}$ = 38.1 Hz), 135.4, 128.1, 126.0, 125.5, 123.8, 121.6, 120.8 (q, $^1J_{C\text{-}F}$ = 255.6 Hz), 119.8, 119.3 (q, $^1J_{C\text{-}F}$ = 269.9 Hz), 46.5, 44.4, 41.6, 36.1. **^{19}F NMR** (376 MHz, CDCl$_3$) δ -57.9, -62.2. **HRMS (ESI)** *m/z:* [M+H]$^+$ Calcd. for $C_{18}H_{15}F_4N_2$ 401.1083; Found 401.1089.

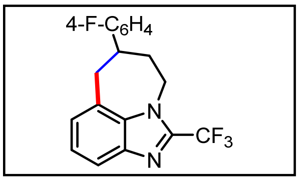

7-(4-Fluorophenyl)-1-(trifluoromethyl)-6,7,8,9-tetrahydro-2,9a-diazabenzo[*cd*]azulene (3n)

White solid (53.5 mg, 80% yield), m.p. 145-146 °C. **^{1}H NMR** (400 MHz, CDCl$_3$) δ 7.73 (d, *J* = 8.0 Hz, 1H), 7.27 – 7.20 (m, 3H), 7.14 (d, *J* = 7.2 Hz, 1H), 7.02 (t, *J* = 8.6 Hz, 2H), 4.80 – 4.57 (m, 1H), 4.39 – 4.14 (m, 1H), 3.49 – 3.16 (m, 3H), 2.61 – 2.46 (m, 1H), 2.43 – 2.15 (m, 1H). **^{13}C NMR** (100 MHz, CDCl$_3$) δ 161.6 (d, $^1J_{C\text{-}F}$ = 244.9 Hz), 142.2 (d, $^4J_{C\text{-}F}$ = 3.1 Hz), 142.0, 141.1 (q, $^2J_{C\text{-}F}$ = 38.1 Hz), 135.3, 128.1 (d, $^3J_{C\text{-}F}$ = 7.9 Hz), 125.9, 125.6, 123.7, 119.5, 119.3 (q, $^1J_{C\text{-}F}$ = 269.6 Hz), 115.7 (d, $^2J_{C\text{-}F}$ = 21.1 Hz), 46.5, 44.2, 41.8, 36.1. **^{19}F NMR** (376 MHz, CDCl$_3$) δ -62.4, -115.9. **HRMS (ESI)** *m/z:* [M+H]$^+$ Calcd. for $C_{18}H_{15}F_4N_2$ 335.1166; Found 335.1170.

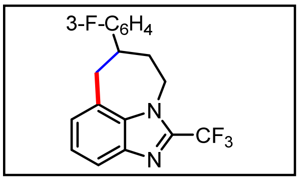

7-(3-Fluorophenyl)-1-(trifluoromethyl)-6,7,8,9-tetrahydro-2,9a-diazabenzo[*cd*]azulene (3o)
White solid (57.5 mg, 86% yield), 98-100 °C. **^{1}H NMR** (400 MHz, $CDCl_3$) δ 7.75 (d, *J* = 8.0 Hz, 1H), 7.36 – 7.24 (m, 2H), 7.16 (d, *J* = 8.0, Hz, 1H), 7.04 (d, *J* = 8.0 Hz, 1H), 7.00 – 6.93 (m, 2H), 4.73 – 4.67 (m, 1H), 4.32 – 4.17 (m, 1H), 3.45 – 3.36 (m, 2H), 3.38 – 3.20 (m, 1H), 2.63 – 2.49 (m, 1H), 2.47 – 2.29 (m, 1H). **^{13}C NMR** (100 MHz, $CDCl_3$) δ 163.1 (d, $^1J_{C\text{-}F}$ = 244.9 Hz), 148.9 (d, $^3J_{C\text{-}F}$ = 6.7 Hz), 142.0, 141.2 (q, $^2J_{C\text{-}F}$ = 38.1 Hz), 135.3, 130.54 (d, $^3J_{C\text{-}F}$ = 8.4 Hz), 126.0 , 125.5, 123.7, 122.4 (d, $^4J_{C\text{-}F}$ = 2.8 Hz), 119.7, 119.3 (q, $^1J_{C\text{-}F}$ = 269.7 Hz), 113.8 (d, $^2J_{C\text{-}F}$ = 20.9 Hz), 113.7 (d, $^2J_{C\text{-}F}$ = 21.3 Hz), 46.5, 44.7, 41.5, 35.9. **^{19}F NMR** (376 MHz, $CDCl_3$) δ -62.2, -112.4 **HRMS (ESI)** *m/z:* $[M+H]^+$ Calcd. for $C_{18}H_{15}F_4N_2$ 335.1166; Found 335.1172.

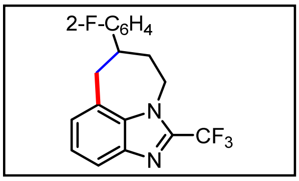

7-(2-Fluorophenyl)-1-(trifluoromethyl)-6,7,8,9-tetrahydro-2,9a-diazabenzo[*cd*]azulene (3p)
White solid (60.2 mg, 90% yield), m.p. 99-100 °C. **^{1}H NMR** (400 MHz, $CDCl_3$) δ 7.75 (d, *J* = 8.0 Hz, 1H), 7.32 – 7.21 (m, 3H), 7.20 – 7.03 (m, 3H), 4.73 – 4.61 (m, 1H), 4.45 – 4.16 (m, 1H), 3.74 – 3.42 (m, 2H), 3.25 – 3.14 (m, 1H), 2.78 – 2.38 (m, 2H). **^{13}C NMR** (100 MHz, $CDCl_3$) δ 160.2 (d, $^1J_{C\text{-}F}$ = 245.3 Hz), 142.1, 141.2 (q, $^2J_{C\text{-}F}$ = 38.1 Hz), 135.4, 132.9 (d, $^2J_{C\text{-}F}$ = 14.5 Hz), 128.4 (d, $^3J_{C\text{-}F}$ = 8.4 Hz), 128.0 (d, $^3J_{C\text{-}F}$ = 4.8 Hz), 125.9, 125.8, 124.6 (d, $^4J_{C\text{-}F}$ = 3.4 Hz), 123.7, 119.6, 119.3 (q, $^1J_{C\text{-}F}$ = 269.7 Hz), 116.0 (d, $^2J_{C\text{-}F}$ = 22.4 Hz), 46.6, 40.0, 38.4, 34.9. **^{19}F NMR** (376 MHz, $CDCl_3$) δ -62.1, -117.8. **HRMS (ESI)** *m/z:* $[M+H]^+$ Calcd. for $C_{18}H_{15}F_4N_2$ 335.1166; Found 335.1170.

Supplementary Note 4

General Procedure for Asymmetric Control

To a 15 mL oven dried tube in glove box were added $Ni(cod)_2$ (5.5 mg, 0.02 mmol, 10 mol%), carbene ligand[6] (0.02 mmol, 10 mol%), ^{t}BuOK (9.0 mg, 0.08 mmol, 40 mol%), benzoimidazole **1** (0.2 mmol), dry degassed toluene (1.0 mL), and $AlMe_3$ (1.0 M/hexane, 0.16 mmol, 80 mol%). The tube was capped, taken outside the glove box, and stirred at 130 °C for 3 h. Then the mixture was cooled to r.t., quenched with 2 mL of a 5% EDTA disodium salt solution, and filtered through a short plug of silica gel, eluting with EtOAc. The filtration was concentrated in *vacuo* to afford the crude product, which was further purified by flash column chromatography on silica gel (EtOAc/hexanes). The enantiomeric excess of pure product was then determined by chiral HPLC.

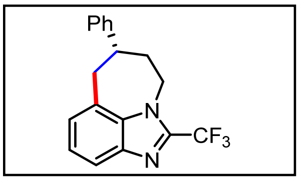

(*R*)-7-Phenyl-1-(trifluoromethyl)-6,7,8,9-tetrahydro-2,9a-diazabenzo[*cd*]azulene ((*R*)-3e)
56% yield (35.4 mg). **HPLC analysis**: Chiracel AD-H column, *n*-Hex/*i*-PrOH = 99:1, 1.0 mL/min, 254 nm, major enantiomer: 14.4 min, minor enantiomer: 13.1 min, 86% ee; $[\alpha]_D^{30}$ = +12.4° (c = 0.4, $CHCl_3$).

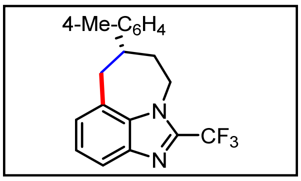

(*R*)-7-(*p*-Tolyl)-1-(trifluoromethyl)-6,7,8,9-tetrahydro-2,9a-diazabenzo[*cd*]azulene ((*R*)-3f)
60% yield (39.6 mg). **HPLC analysis**: Chiracel OD-H column, *n*-Hex/*i*-PrOH = 99:1, 1.0 mL/min, 254 nm, major enantiomer: 16.5 min, minor enantiomer: 15.0 min, 86% ee; $[\alpha]_D^{30}$ = +12.8° (c = 0.5, $CHCl_3$).

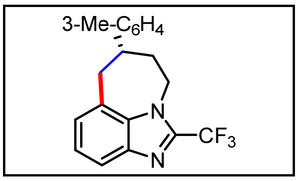

(*R*)-7-(*m*-Tolyl)-1-(trifluoromethyl)-6,7,8,9-tetrahydro-2,9a-diazabenzo[*cd*]azulene ((*R*)-3g)
60% yield (39.6 mg). **HPLC analysis**: Chiracel OD-H column, *n*-Hex/*i*-PrOH = 99:1, 1.0 mL/min, 254 nm, major enantiomer: 24.9 min, minor enantiomer: 21.2 min, 88% ee; $[\alpha]_D^{30}$ = +15.6° (c = 0.5, $CHCl_3$).

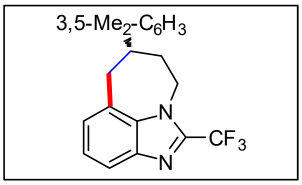

(*R*)-7-(3,5-Dimethylphenyl)-1-(trifluoromethyl)-6,7,8,9-tetrahydro-2,9a-diazabenzo[*cd*]azulene ((*R*)-3h)
50% yield (34.4 mg). **HPLC analysis**: Chiracel IA column, *n*-Hex/*i*-PrOH = 99:1, 1.0 mL/min, 254 nm, major enantiomer: 8.9 min, minor enantiomer: 10.6 min, 90% ee; $[\alpha]_D^{30}$ = +8.0° (c = 0.2, $CHCl_3$).

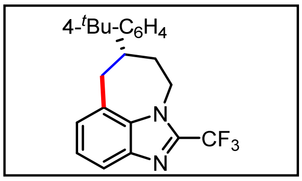

(*R*)-7-(4-(*tert*-Butyl)phenyl)-1-(trifluoromethyl)-6,7,8,9-tetrahydro-2,9a-diazabenzo[*cd*]azulene ((*R*)-3i)
52% yield (38.6 mg). **HPLC analysis**: Chiracel OD-H column, *n*-Hex/*i*-PrOH = 99.5:0.5, 1.0 mL/min, 254 nm, major enantiomer: 12.0 min, minor enantiomer: 13.7 min, 88% ee; $[\alpha]_D^{30}$ = +16.8° (c = 0.4, $CHCl_3$).

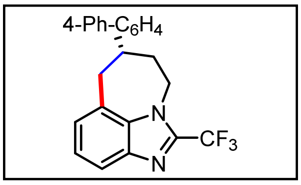

(*R*)-7-([1,1'-Biphenyl]-4-yl)-1-(trifluoromethyl)-6,7,8,9-tetrahydro-2,9a-diazabenzo[*cd*]azulene ((*R*)-3j)

56% yield (43.8 mg). **HPLC analysis**: Chiracel IA column, *n*-Hex/*i*-PrOH = 99:1, 1.0 mL/min, 254 nm, major enantiomer: 33.8 min, minor enantiomer: 30.9 min, 89% ee; $[\alpha]_D^{30}$ = +12.5° (c = 0.2, $CHCl_3$).

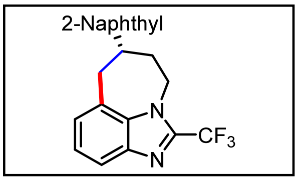

(*R*)-7-(Naphthalen-2-yl)-1-(trifluoromethyl)-6,7,8,9-tetrahydro-2,9a-diazabenzo[*cd*]azulene ((*R*)-3l)

46% yield (33.5 mg). **HPLC analysis**: Chiracel AD-H column, *n*-Hex/*i*-PrOH = 99:1, 1.0 mL/min, 254 nm, major enantiomer: 26.7 min, minor enantiomer: 21.9 min, 86% ee; $[\alpha]_D^{30}$ = +23.2° (c = 0.5, $CHCl_3$).

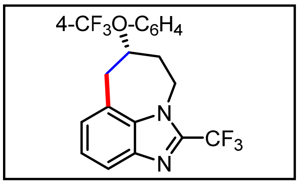

(*R*)-7-(4-(Trifluoromethoxy)phenyl)-1-(trifluoromethyl)-6,7,8,9-tetrahydro-2,9a-diazabenzo[*cd*]azulene ((*R*)-3m)

68% yield (54.5 mg). **HPLC analysis**: Chiracel AD-H column, *n*-Hex/*i*-PrOH = 99:1, 1.0 mL/min, 254 nm, major enantiomer: 15.1 min, minor enantiomer: 13.6 min, 87% ee; $[\alpha]_D^{30}$ = +11.2° (c = 0.1, $CHCl_3$).

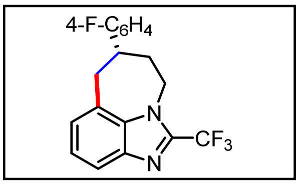

(*R*)-7-(4-Fluorophenyl)-1-(trifluoromethyl)-6,7,8,9-tetrahydro-2,9a-diazabenzo[*cd*]azulene ((*R*)-3n)

68% yield (45.4 mg). **HPLC analysis**: Chiracel AD-H column, *n*-Hex/*i*-PrOH = 99:1, 1.0 mL/min, 254 nm, major enantiomer: 18.9 min, minor enantiomer: 16.5 min, 86% ee; $[\alpha]_D^{30}$ = +29.0° (c = 0.2, $CHCl_3$).

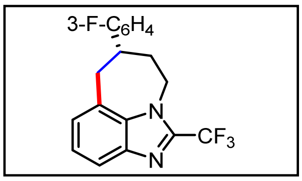

(*R*)-7-(3-Fluorophenyl)-1-(trifluoromethyl)-6,7,8,9-tetrahydro-2,9a-diazabenzo[*cd*]azulene ((*R*)-

3o)

58% yield (38.7 mg). **HPLC analysis**: Chiracel AD-H column, *n*-Hex/*i*-PrOH = 99:1, 1.0 mL/min, 254 nm, major enantiomer: 28.2 min, minor enantiomer: 26.0 min, 89% ee; $[\alpha]^{30}_{D}$ = +8.6° (c = 0.1, $CHCl_3$).

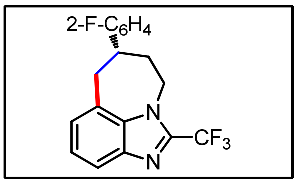

(*R*)-7-(2-Fluorophenyl)-1-(trifluoromethyl)-6,7,8,9-tetrahydro-2,9a-diazabenzo[*cd*]azulene ((*R*)-3p)

65% yield (43.4 mg). **HPLC analysis**: Chiracel AD-H column, *n*-Hex/*i*-PrOH = 99:1, 1.0 mL/min, 254 nm, major enantiomer: 11.7 min, minor enantiomer: 10.7 min, 83% ee; $[\alpha]^{30}_{D}$ = +10.2° (c = 0.1, $CHCl_3$).

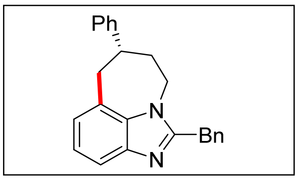

(*R*)-1-Benzyl-7-phenyl-6,7,8,9-tetrahydro-2,9a-diazabenzo[*cd*]azulene((*R*)-3q)

White solid (32.5 mg, 80% yield). **^{1}H NMR** (400 MHz, $CDCl_3$) δ 7.65 (d, *J* = 7.6 Hz, 1H), 7.35 – 7.11 (m, 11H), 6.99 (d, *J* = 7.2 Hz, 1H), 4.42 – 4.23 (m, 3H), 3.77 (t, *J* = 10.8 Hz, 1H), 3.33 (d, *J* = 9.6 Hz, 2H), 3.25 – 3.01 (m, 1H), 2.41 – 2.27 (m, 1H), 2.25 – 2.15 (m, 1H). **^{13}C NMR** (100 MHz, $CDCl_3$) δ 153.5, 147.0, 143.3, 136.2, 134.9, 128.9, 128.8, 128.4, 126.9, 126.6, 126.57, 124.5, 123.2, 121.9, 117.3, 46.4, 45.1, 42.5, 34.0, 34.9. **HRMS (ESI)** *m/z:* $[M+H]^+$ Calcd. for $C_{24}H_{23}N_2$ 339.1856; Found 339.1858. **HPLC analysis**: Chiracel IB column, *n*-Hex/*i*-PrOH = 80:20, 1.0 mL/min, 254 nm, major enantiomer: 13.0 min, minor enantiomer: 12.0 min, 87% ee; $[\alpha]^{30}_{D}$ = +14.6° (c = 0.1, $CHCl_3$).

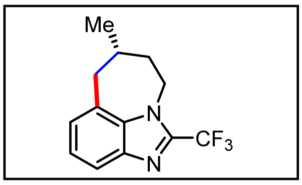

(*R*)-7-Methyl-1-(trifluoromethyl)-6,7,8,9-tetrahydro-2,9a-diazabenzo[*cd*]azulene ((*R*)-3a)

92% yield (46.8 mg). **HPLC analysis**: Chiracel AD-H column, *n*-Hex/*i*-PrOH = 99.5:0.5, 1.0 mL/min, 254 nm, major enantiomer: 11.9 min, minor enantiomer: 10.9 min, 68% ee; $[\alpha]^{30}_{D}$ = +29.6° (c = 0.5, $CHCl_3$).

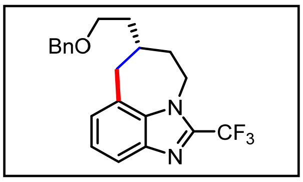

(*R*)-7-(2-(Benzyloxy)ethyl)-1-(trifluoromethyl)-6,7,8,9-tetrahydro-2,9a-diazabenzo[*cd*]azulene ((*R*)-3d)

54% yield (40.4 mg). **HPLC analysis**: Chiracel AD-H column, *n*-Hex/*i*-PrOH = 99:1, 1.0 mL/min, 254 nm, major enantiomer: 20.8 min, minor enantiomer: 22.3 min, 70% ee; $[\alpha]^{30}_{D}$ = +16.6° (c = 0.1, $CHCl_3$).

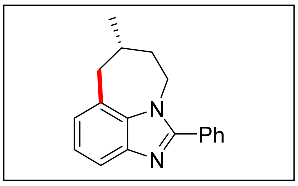

(*R*)-7-Methyl-1-phenyl-6,7,8,9-tetrahydro-2,9a-diazabenzo[*cd*]azulene((*R*)-3r)

White solid (35.6 mg, 68% yield). m.p. 108-109 °C. **^{1}H NMR** (400 MHz, $CDCl_3$) δ 7.72 (dd, *J* = 7.6, 2.0 Hz, 2H), 7.64 (d, *J* = 8.0 Hz, 1H), 7.55 – 7.47 (m, 3H), 7.17 (t, *J* = 7.6 Hz, 1H), 7.01 (d, *J* = 7.2 Hz, 1H), 4.45 – 4.35 (m, 1H), 4.15 – 4.09 (m, 1H), 3.18 (dd, *J* = 15.6, 4.0 Hz, 1H), 2.96 (dd, *J* = 15.6, 9.2 Hz, 1H), 2.37 – 2.29 (m, 1H), 2.27 – 2.16 (m, 1H), 1.84 – 1.71 (m, 2H), 1.15 (d, *J* = 6.8 Hz, 3H). **^{13}C NMR** (100 MHz, $CDCl_3$) δ 154.7, 143.5, 136.1, 130.6, 129.7, 129.7, 128.8, 125.7, 123.3, 122.5, 117.7, 46.3, 40.1, 36.5, 33.2, 23.2. **HRMS (ESI)** *m/z:* $[M+H]^+$ Calcd. for $C_{18}H_{19}N_2$ 263.1543; Found 263.1546. **HPLC analysis**: Chiracel OD-H column, n-Hex/*i*-PrOH = 90:10, 1.0 mL/min, 254 nm, major enantiomer: 26.9 min, minor enantiomer: 22.4 min, 67% ee; $[\alpha]_D^{30}$= +11.8° (c = 0.1, $CHCl_3$).

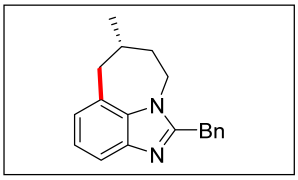

(*R*)-1-Benzyl-7-methyl-6,7,8,9-tetrahydro-2,9a-diazabenzo[*cd*]azulene((*R*)-3s)

White solid (44.2 mg, 80% yield). **^{1}H NMR** (400 MHz, $CDCl_3$) δ 7.61 (d, *J* = 8.0 Hz, 1H), 7.28 (dd, *J* = 12.8, 5.2 Hz, 2H), 7.21 (t, *J* = 8.8 Hz, 3H), 7.14 (t, *J* = 7.6 Hz, 1H), 6.98 (d, *J* = 7.2 Hz, 1H), 4.32 (q, *J* = 16.0 Hz, 2H), 4.22 – 4.19 (m, 1H), 3.76 – 3.69 (m, 1H), 3.12 (dd, *J* = 15.6, 4.0 Hz, 1H), 2.85 (dd, *J* = 15.6, 9.2 Hz, 1H), 2.37 – 2.29 (m, 1H), 2.06 – 1.97 (m, 1H), 1.79 – 1.67 (m, 1H), 1.10 (d, *J* = 6.8 Hz, 3H). **^{13}C NMR** (100 MHz, $CDCl_3$) δ 153.4, 136.5, 135.1, 128.9, 128.5, 127.0, 125.0, 123.1, 121.8, 117.1, 46.0, 42.3, 36.2, 35.0, 33.4, 23.5. **HRMS (ESI)** *m/z:* $[M+H]^+$ Calcd. for $C_{19}H_{21}N_2$ 277.1699; Found 277.1702. **HPLC analysis**: Chiracel AD-H column, *n*-Hex/*i*-PrOH = 92:8, 1.0 mL/min, 254 nm, major enantiomer: 11.7 min, minor enantiomer: 10.7 min, 76% ee; $[\alpha]_D^{30}$ = +24.7° (c = 0.1, $CHCl_3$).

Supplementary Note 5

Stereochemical model:

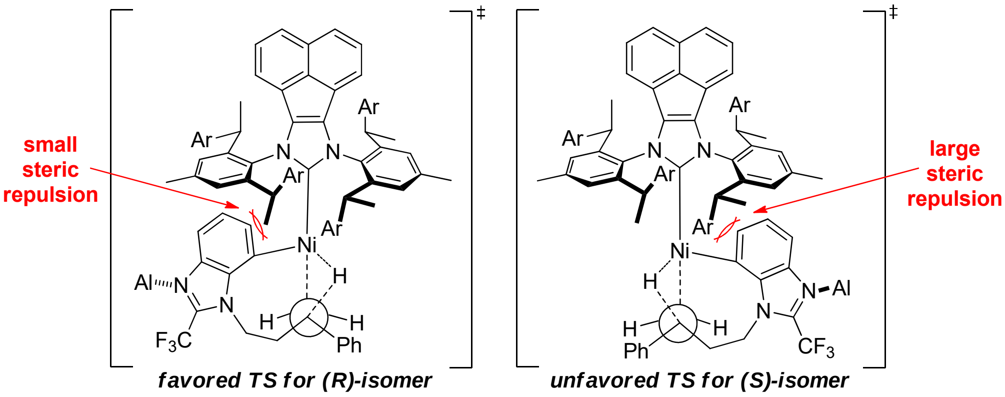

Supplementary Figure 1. Stereochemical model.

Chiral ligand examination:

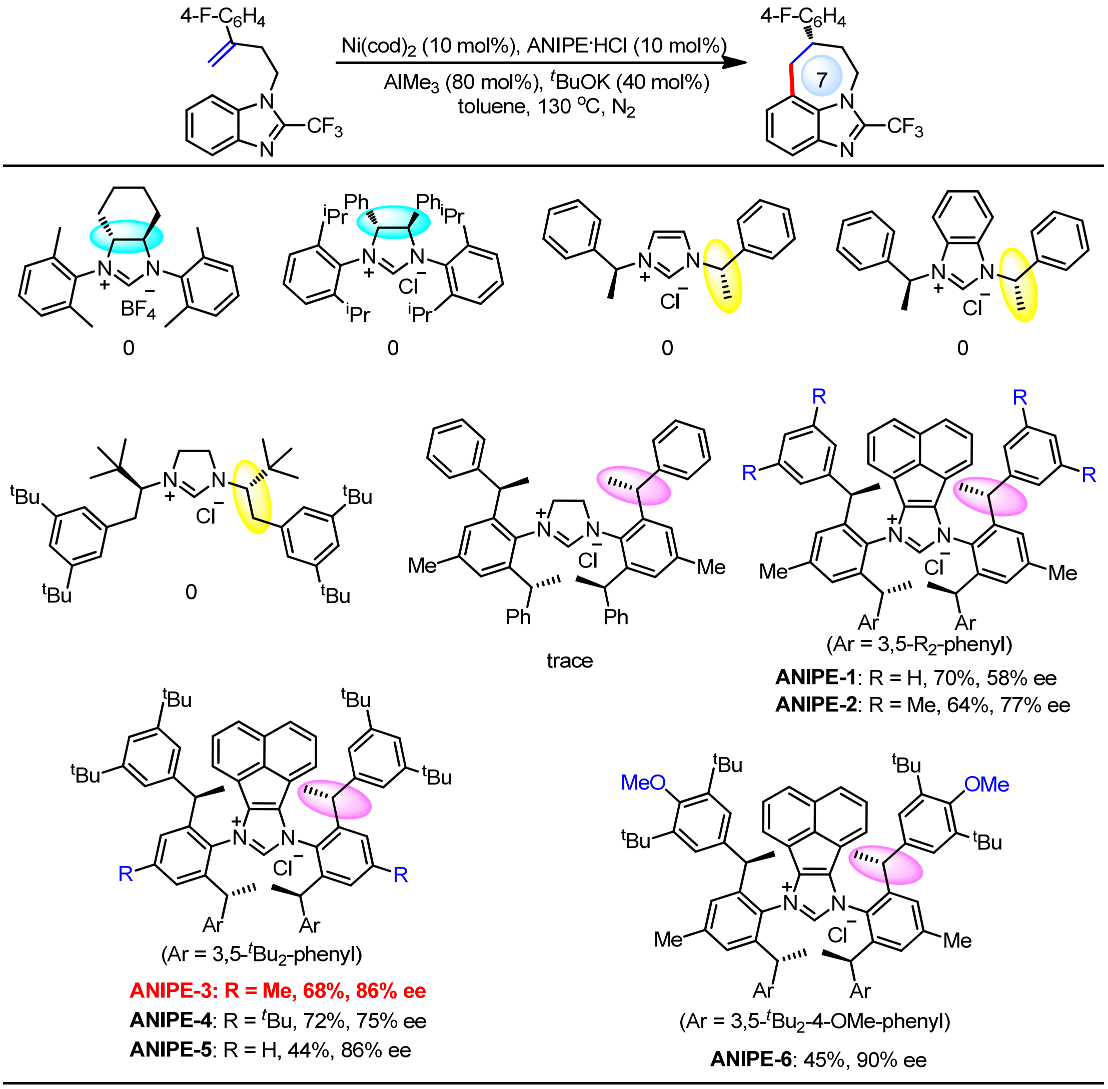

Supplementary Figure 2. Chiral carbene examination.

Supplementary Note 6

Gram-scale synthesis

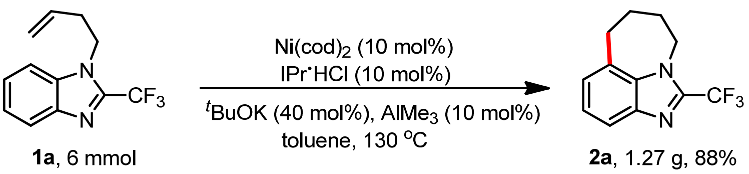

1a, 6 mmol　　**2a**, 1.27 g, 88%

To a 50 mL oven dried tube in glove box were added Ni(cod)$_2$ (165 mg, 10 mol%), IPr·HCl (258 mg, 10 mol%), ^{t}BuOK (270 mg, 40 mol%), benzoimidazole **1a** (1.44 g, 6 mmol), dry degassed toluene (10.0 mL), and AlMe$_3$ (1.0 M/hexane, 600 µL, 10 mol%). The tube was capped, taken outside the

glove box, and stirred at 130 °C for 3 h. Then the mixture was cooled to r.t., quenched with 2 mL of a 5% EDTA disodium salt solution, filtered through a short plug of silica gel, eluting with EtOAc. The filtration was concentrated in *vacuo* to afford the crude product, which was purified by chromatography (EtOAc/hexane) to give pure **2a** (1.27 g, 88% yield).

Product transformation

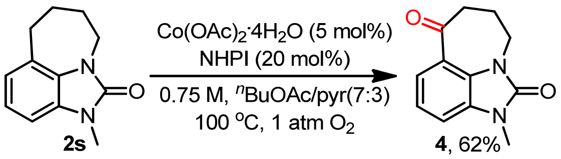

According to the literature procedure:[7] A 4 mL vial was charged with $Co(OAc)_2·4H_2O$ (2.5 mg, 0.0l mmol), *N*-hydroxyphthalimide (6.5 mg, 0.04 mmol), 2s (0.2 mmol), and BuOAc (0.7 mL), and pyridine (0.3 mL). The vial was sealed with a PTFE-lined cap. The headspace of the vial was purged with an O_2-filled balloon for 2 min. The vial was transferred to a heating block pre-set to 100 °C with the O_2-filled balloon still attached, and the reaction mixture was vigorously stirred for 12 h, then cooled to r.t, filtered through a short plug of silica gel (EtOAc). The filtration was concentration in *vacuo* to give the crude product, which was further purified by chromatography on silica gel (EtOAc/hexane), affording a yellow solid.[8] (26.8 mg, 62% yield). **^{1}H NMR** (400 MHz, $CDCl_3$) δ 7.83 – 7.78 (m, 1H), 7.18 – 7.13 (m, 2H), 4.15 – 4.11 (m, 2H), 3.47 (s, 3H), 3.10 – 3.04 (m, 2H), 2.27 – 2.21 (m, 2H). **^{13}C NMR** (100 MHz, $CDCl_3$) δ 197.3, 154.0, 131.2, 129.0, 122.8, 120.7, 118.7, 111.8, 45.6, 44.6, 27.5, 20.4.

Supplementary Note 7

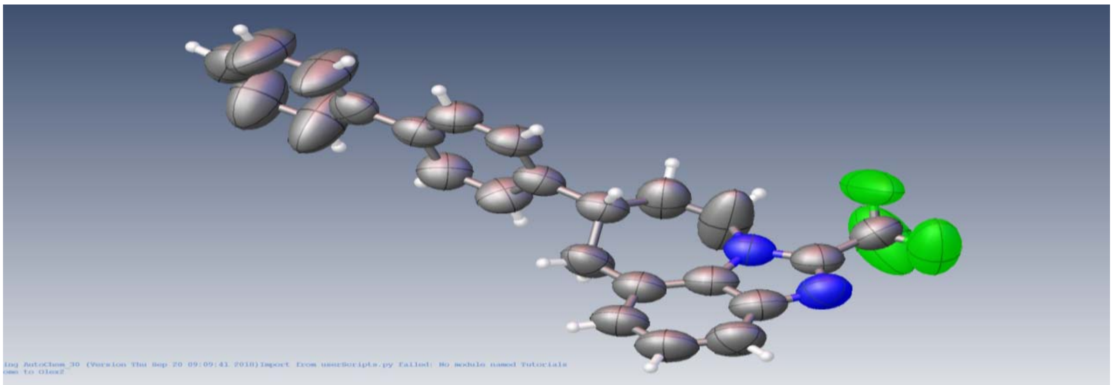

Supplementary Figure 3. Crystal structure information of **3g** (deposited as CCDC 2009572). Data block name: data_p20181121b, unit cell parameters: a 5.43740(10) b 15.3481(2) c 23.1542(3) P212121.

Supplementary Note 8

Deuterium-labeling experiment

To a 15 mL oven dried tube in glove box were added $Ni(cod)_2$ (5.5 mg, 10 mol%), IPr (8.6 mg, 10 mol%), ^{t}BuOK (9 mg, 0.08 mmol), benzoimidazole derivative *d*$_4$-**1a** (0.2 mmol, 1 equiv), dry degassed toluene (2.0 mL), and $AlMe_3$ (1.0 M/hexane, 20 µL, 10 mol%). The tube was capped, taken outside the glove box and stirred at 130 °C for 3 h. Then the reaction mixture was cooled to r.t.,

quenched with 2 mL of a 5% EDTA disodium salt solution, and filtered through a short plug of silica gel, eluting with EtOAc. The filtration was concentrated in *vacuo* to afford the crude d_4-**2a**, which was purified by flash column chromatography on silica gel (EtOAc/hexanes).

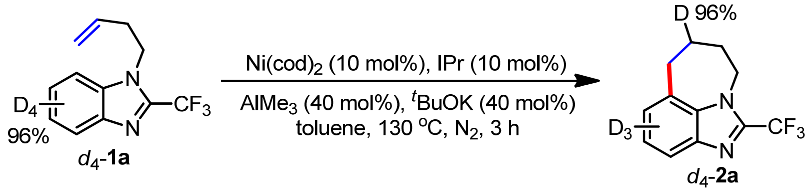

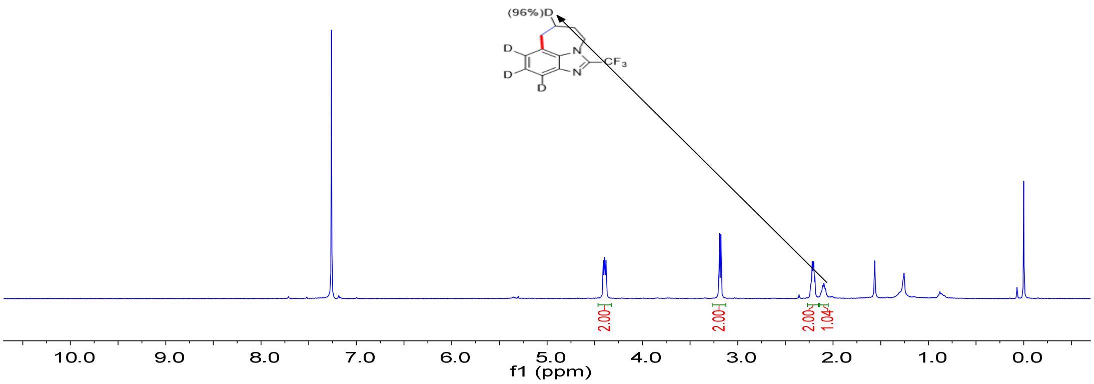

Supplementary Figure 4. Deuterium labeling experiment.

Competitive experiment

To a 15 mL oven dried tube in glove box were added Ni(cod)$_2$ (5.5 mg, 10 mol%), IPr (8.6 mg, 10 mol%), ^{t}BuOK (9 mg, 0.08 mmol), benzoimidazole **1a** (0.1 mmol, 0.5 eq.), d_4-**1a** (0.2 mmol, 1 eq.), dry degassed toluene (2.0 mL), and AlMe$_3$ (1.0 M/hexane, 20 µL, 10 mol%). The tube was capped, taken outside the glove box and stirred at 130 °C for 12 min. The mixture was then cooled to r.t., quenched with 2 mL of a 5% EDTA disodium salt solution and filtered through a short plug of silica gel. The filtration was concentrated and dissolved in 2.0 mL $CDCl_3$ with CH_2Br_2 (14 µL, 0.2 mmol) as the internal standard. The ratio of two products was determined by ^{1}H NMR.

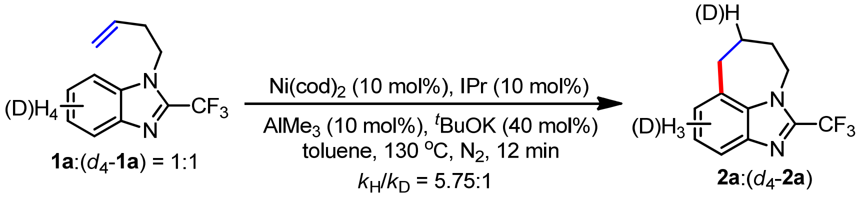

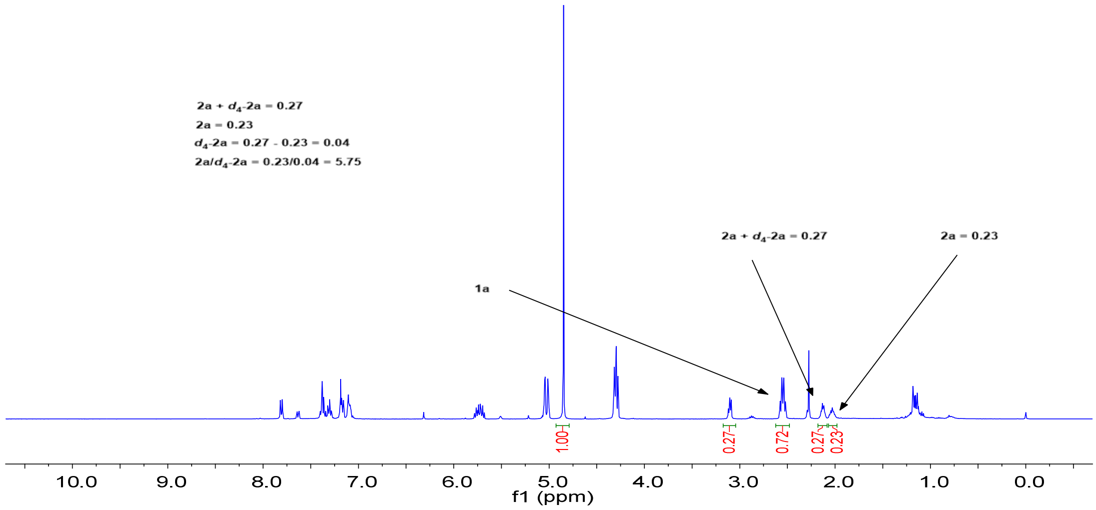

Supplementary Figure 5. Competitive experiment.

Parallel experiments for k_H/k_D

Parallel reactions were set up following the general procedure by using **1a** and **1a**-*d* as substrate, respectively. Aliquots were taken at 3 minute intervals for the first fifteen minutes. Product yield was determined by ^{1}H NMR using CH_2Br_2 as an internal standard.

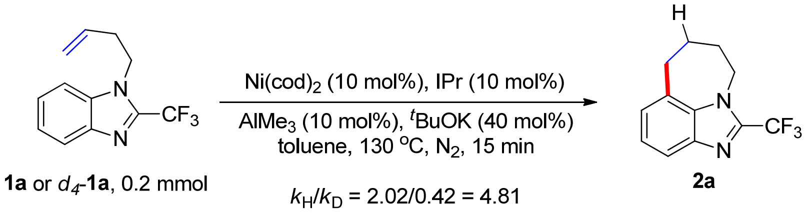

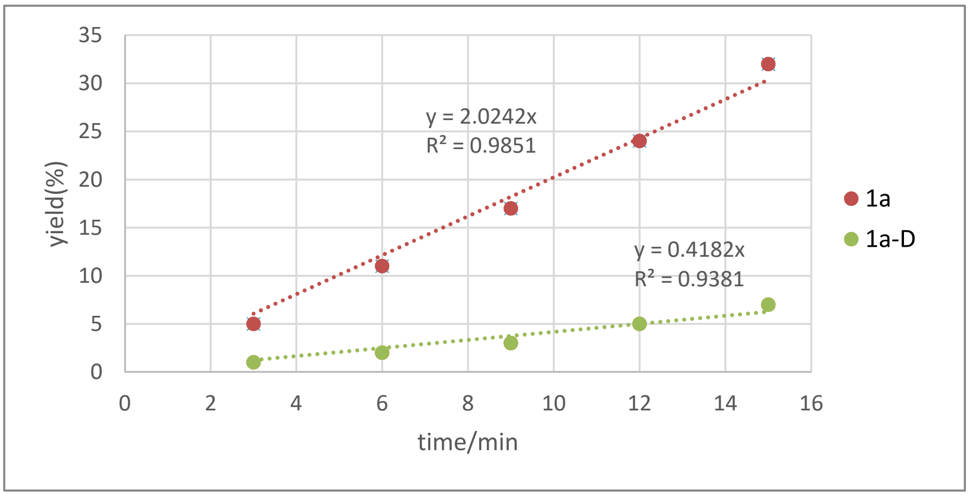

Supplementary Figure 6. KIE determination via parallel experiments.

^{19}F NMR tracking experiments

Single or mixed components of stoichiometric substrate **1a**, nickel, $AlMe_3$, carbene ligand and base in C_6D_6 with $PhCF_3$ as the internal standard were detected by ^{19}F NMR.

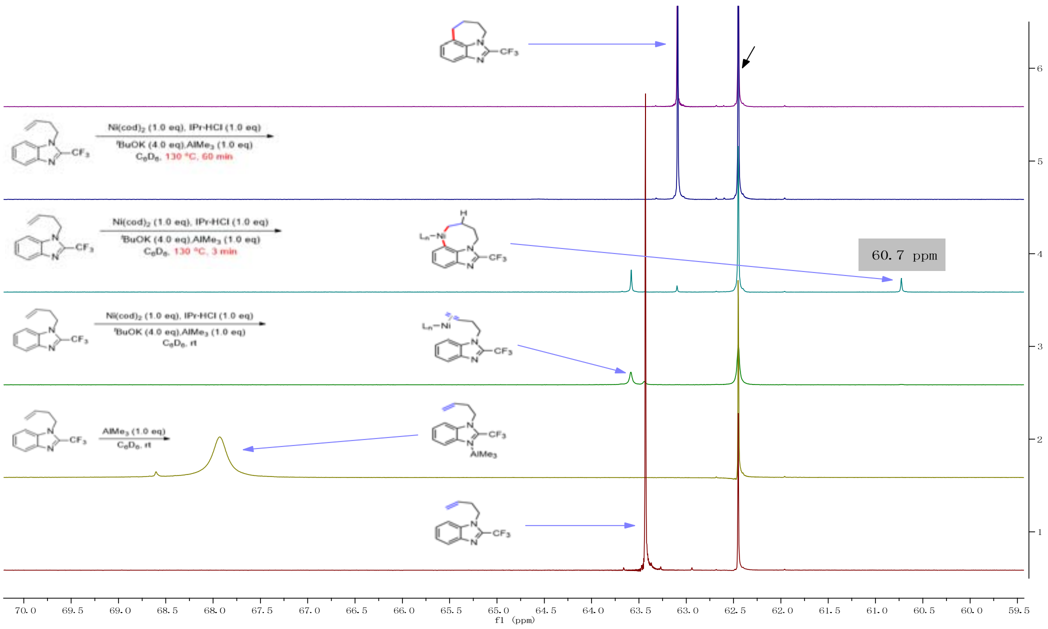

Supplementary Figure 7. ^{19}F NMR tracking experiments

In these stoichiometric experiments, the coordination of $AlMe_3$ with the substrate was not so apparent, likely due to the presence of stoichiometric IPr and ^{t}BuOK, which would have competitive coordination with $AlMe_3$. However, the coordination of nickel with the alkene was significant and rapid. To make a comparison, the substrate without an alkene motif was also examined and no coordination was observed (see the bottom figure).

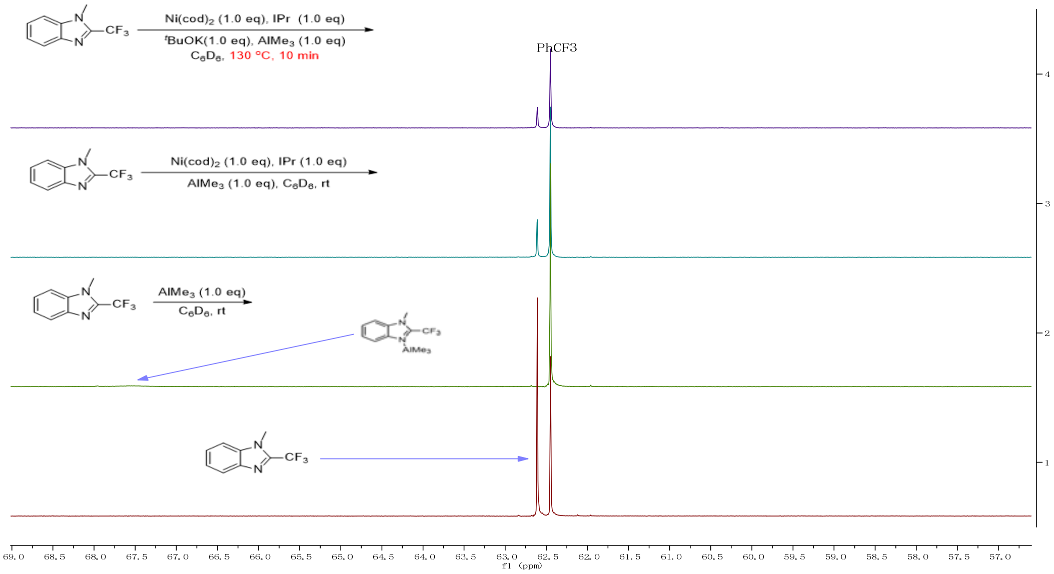

Supplementary Figure 8. ^{19}F NMR tracking experiments

Despite the lack of characterization because of unavailable isolation, the observed intermediate (a distinctive peak at 60.7 ppm) after heating for 3 min was proposed to be an 8-membered nickelacycle, which was rapidly formed and then gradually disappeared with the increase of the product.

Supplementary Figures

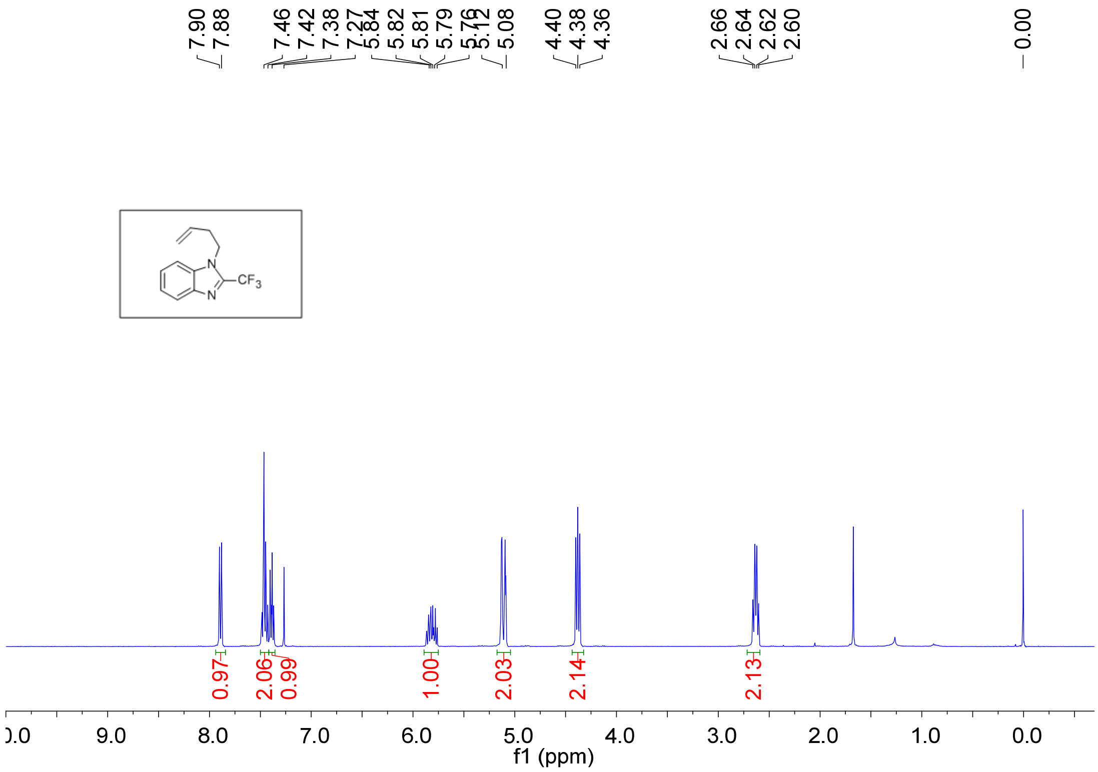

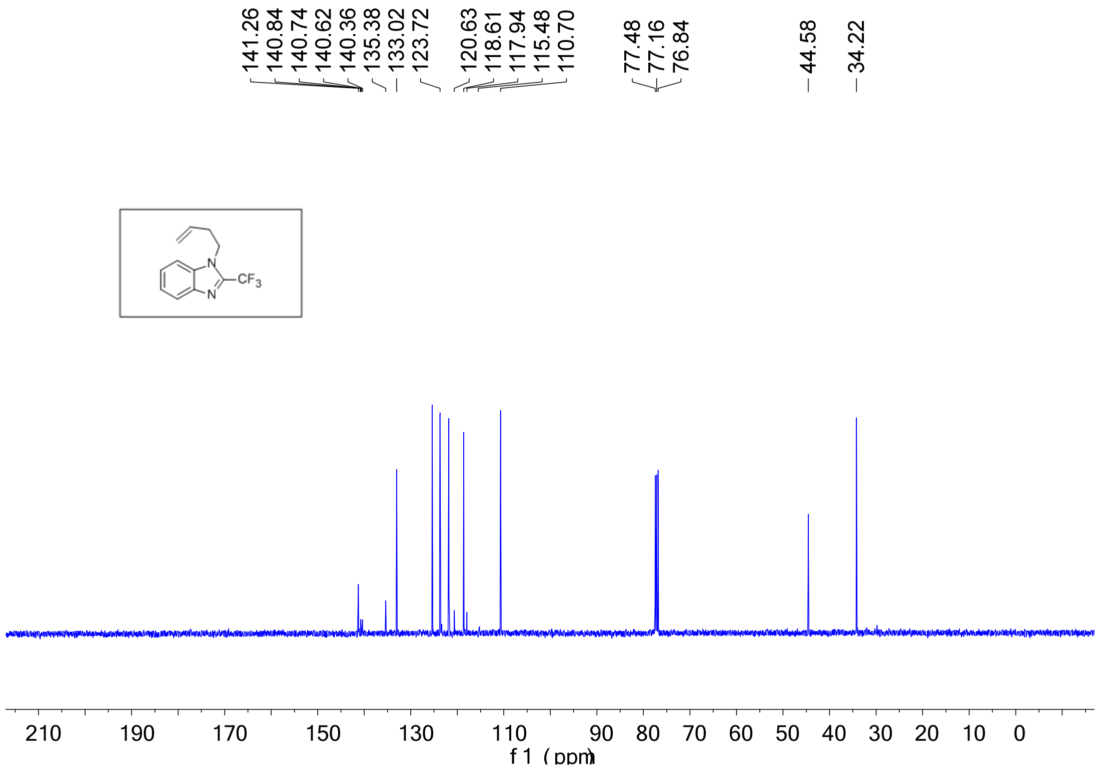

Supplementary Figure 9. ^{1}H and ^{13}C NMR spectra of compound **1a** in $CDCl_3$.

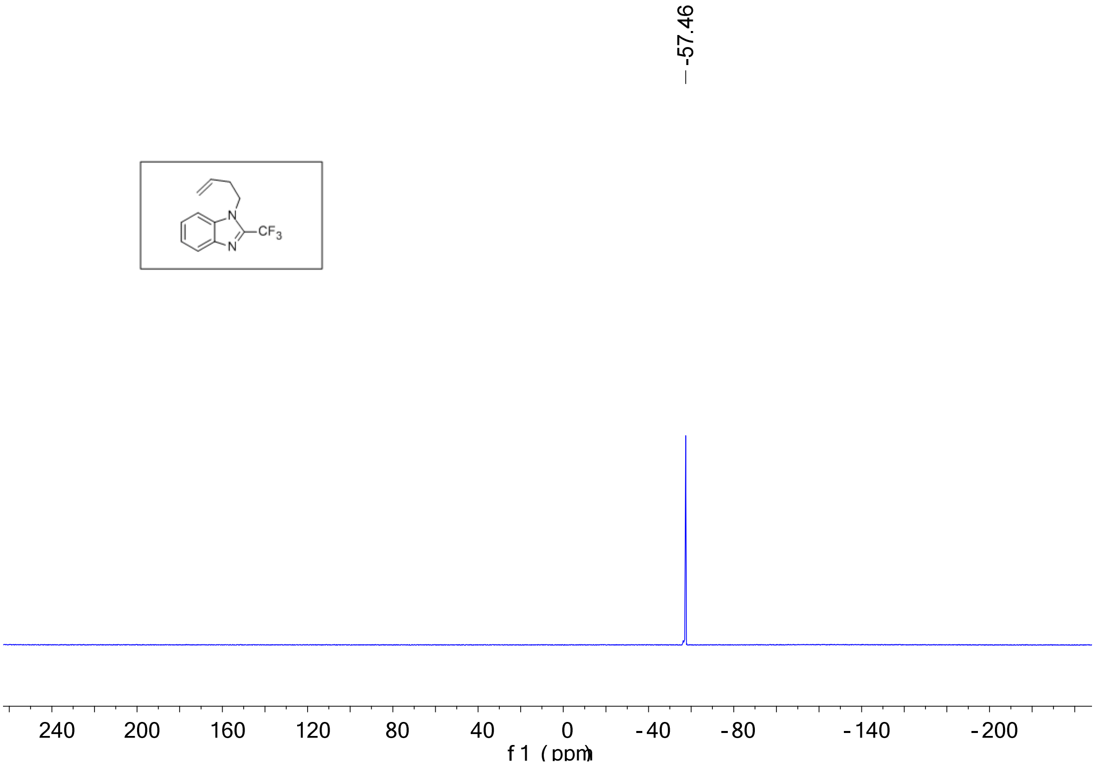

Supplementary Figure 10. ^{19}F NMR spectrum of compound **1a** in $CDCl_3$.

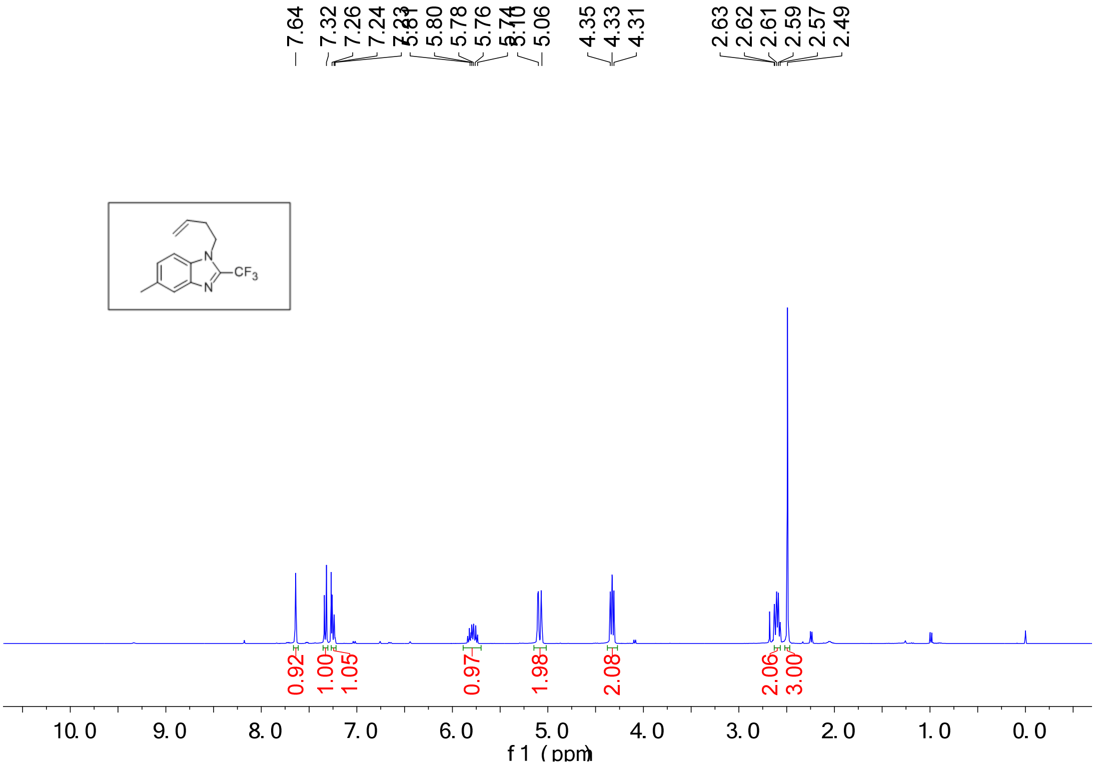

Supplementary Figure 11. ^{1}H NMR spectrum of compound **1b** in $CDCl_3$.

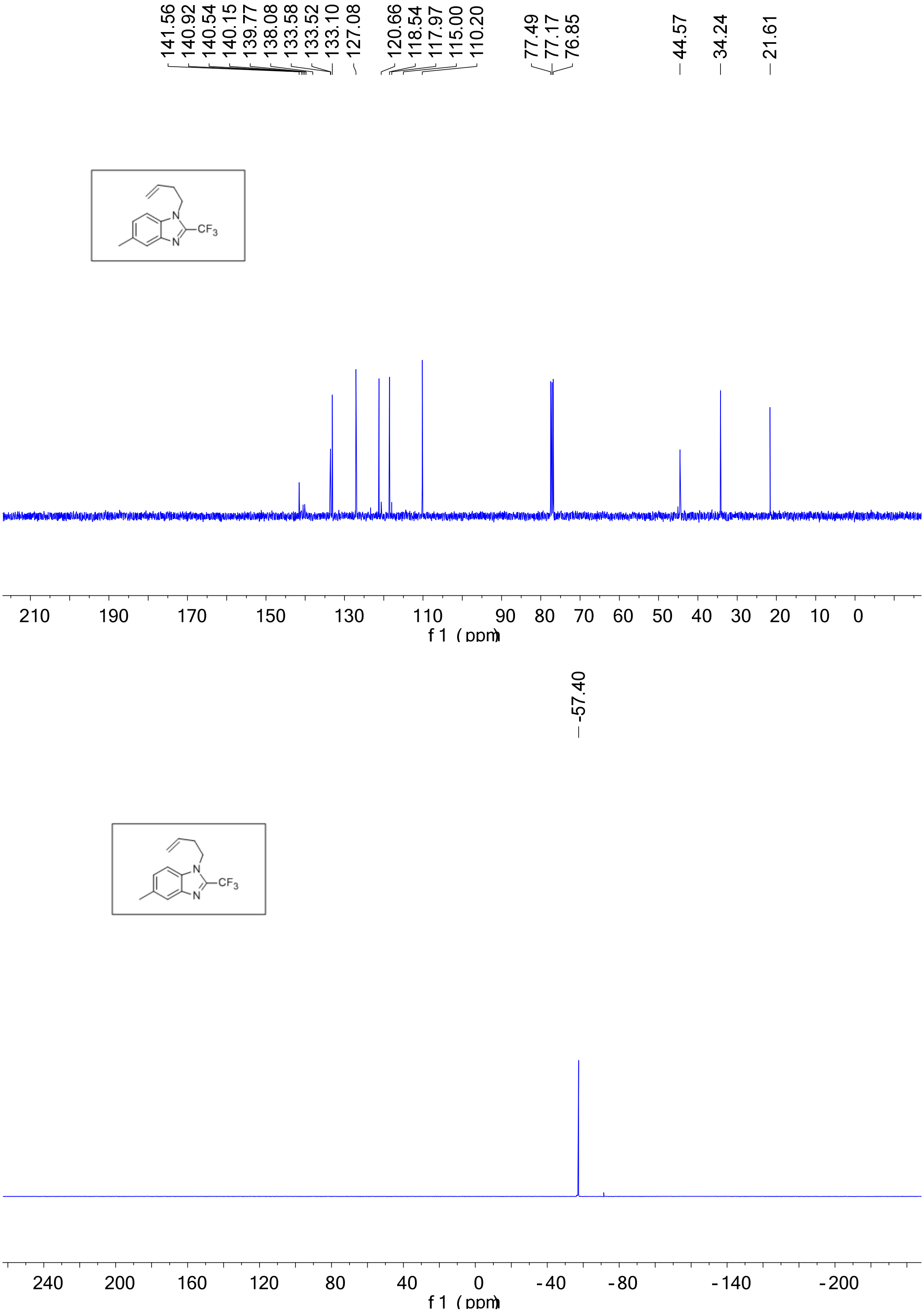

Supplementary Figure 12. ^{13}C and ^{19}F NMR spectra of compound **1b** in $CDCl_3$.

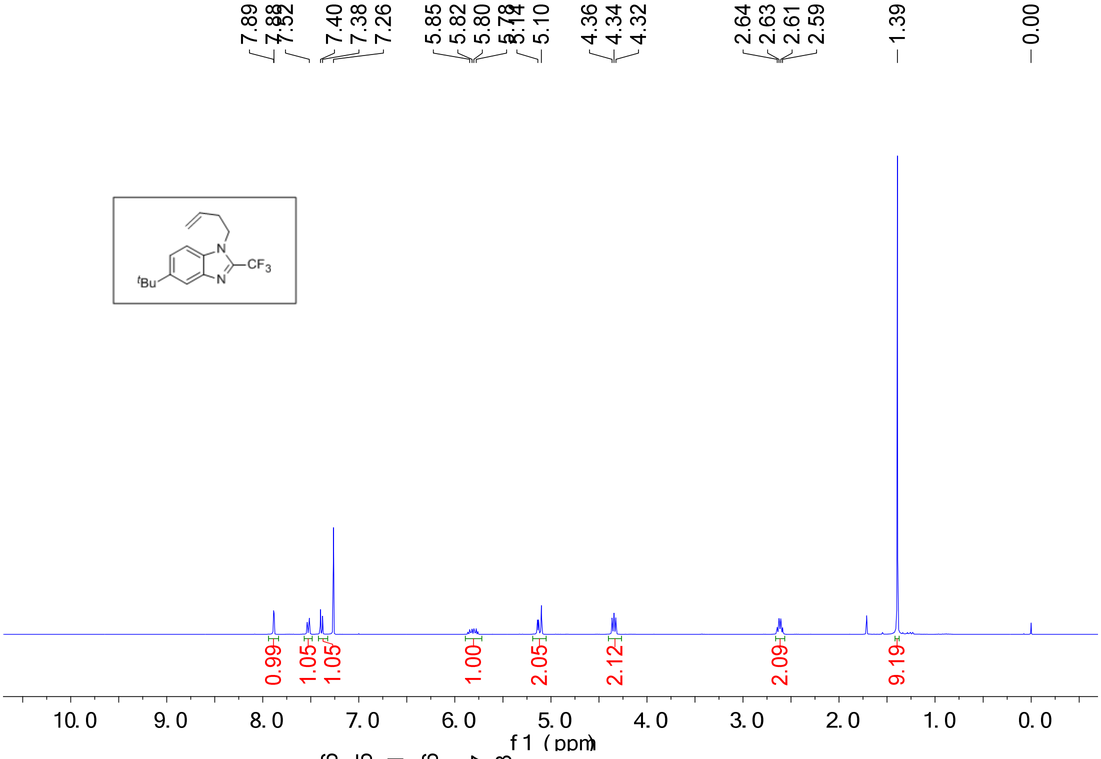

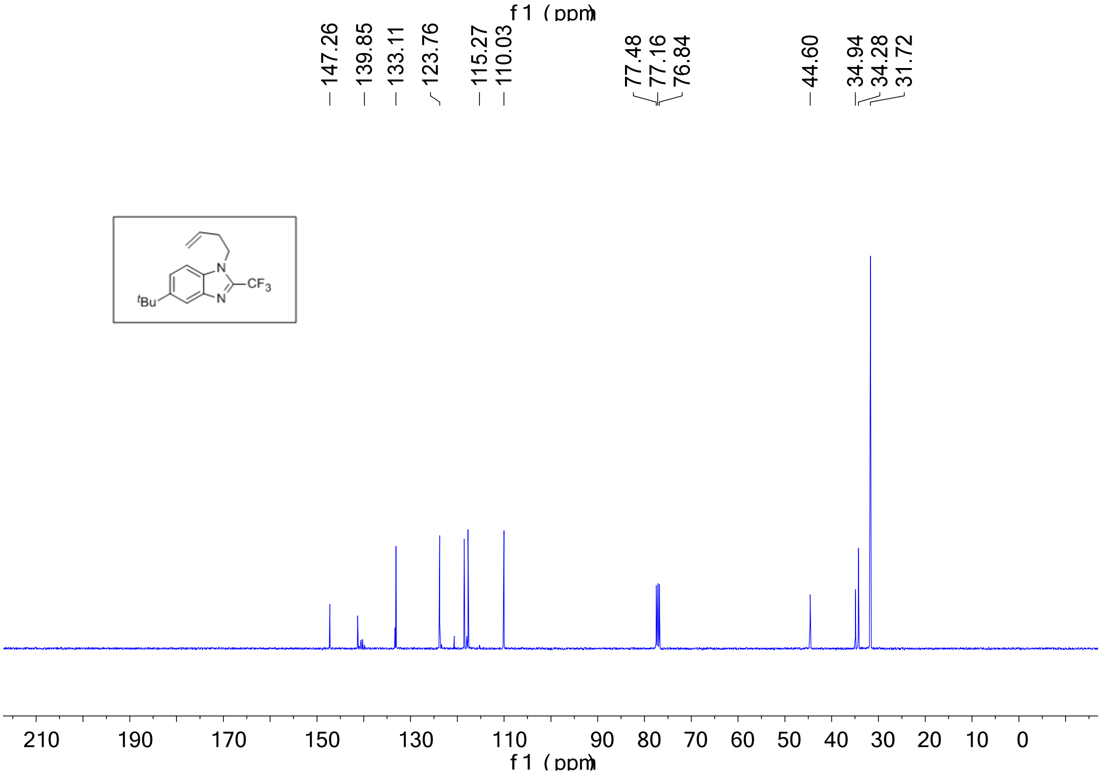

Supplementary Figure 13. ^{1}H and ^{13}C NMR spectra of compound **1c** in $CDCl_3$.

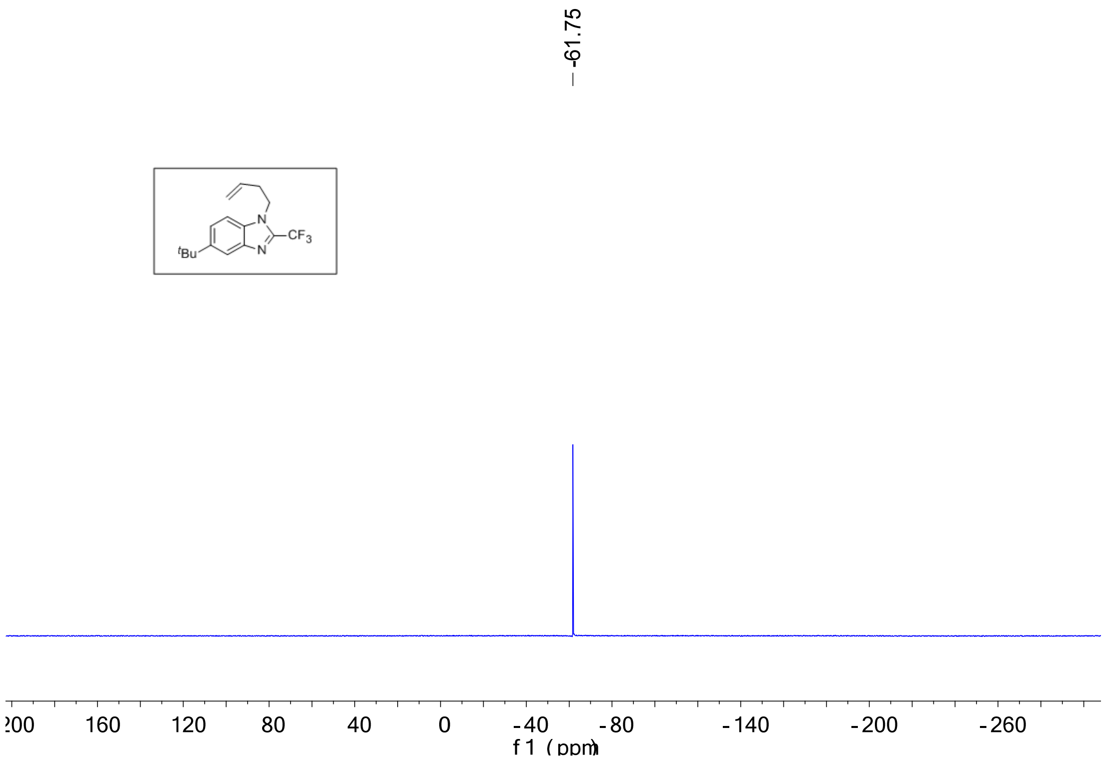

Supplementary Figure 14. ^{19}F NMR spectrum of compound **1c** in $CDCl_3$.

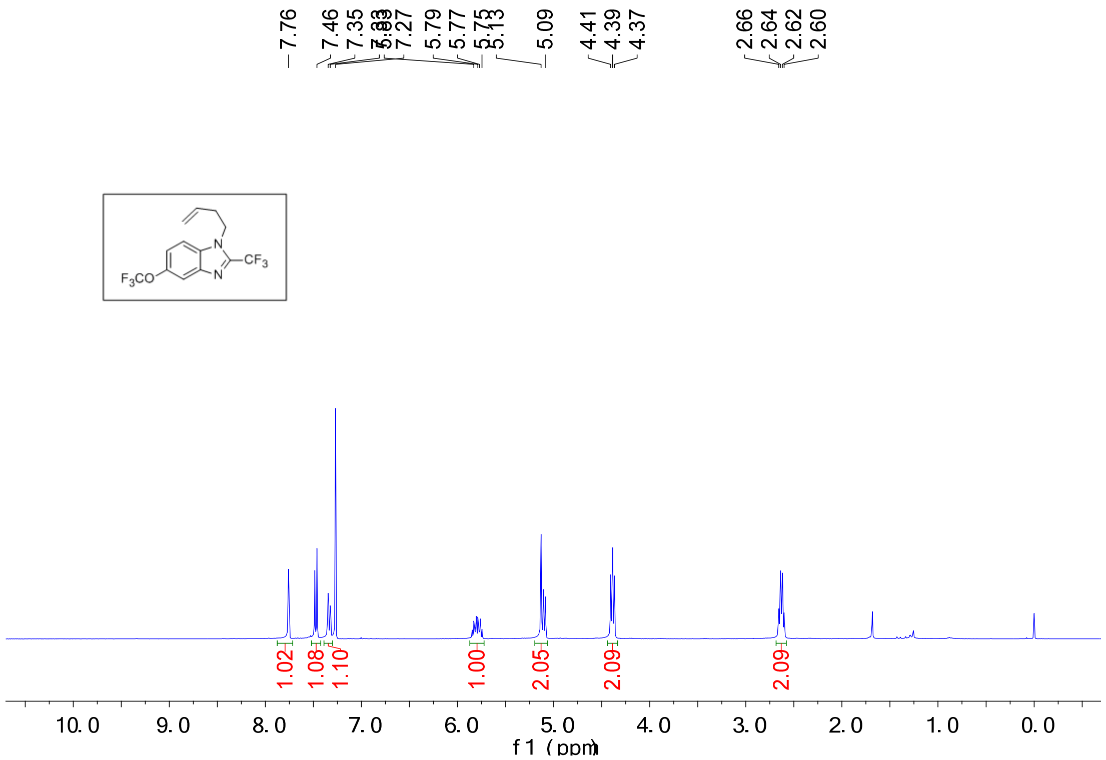

Supplementary Figure 15. ^{1}H NMR spectrum of compound **1d** in $CDCl_3$.

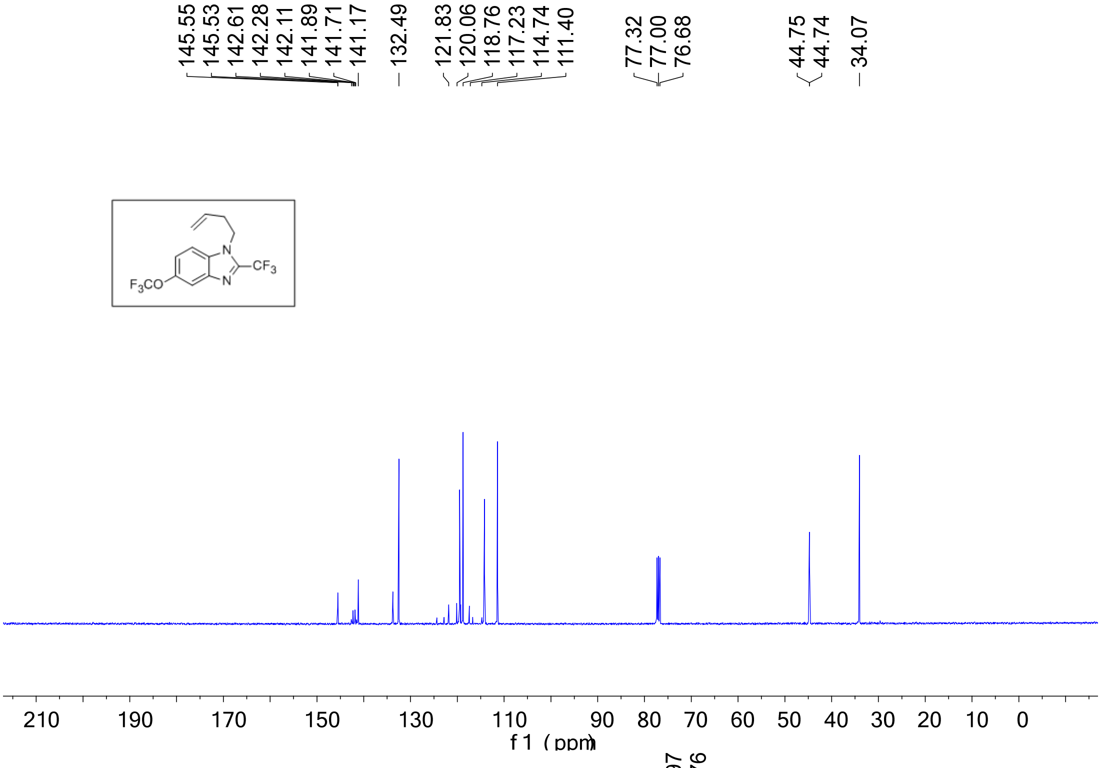

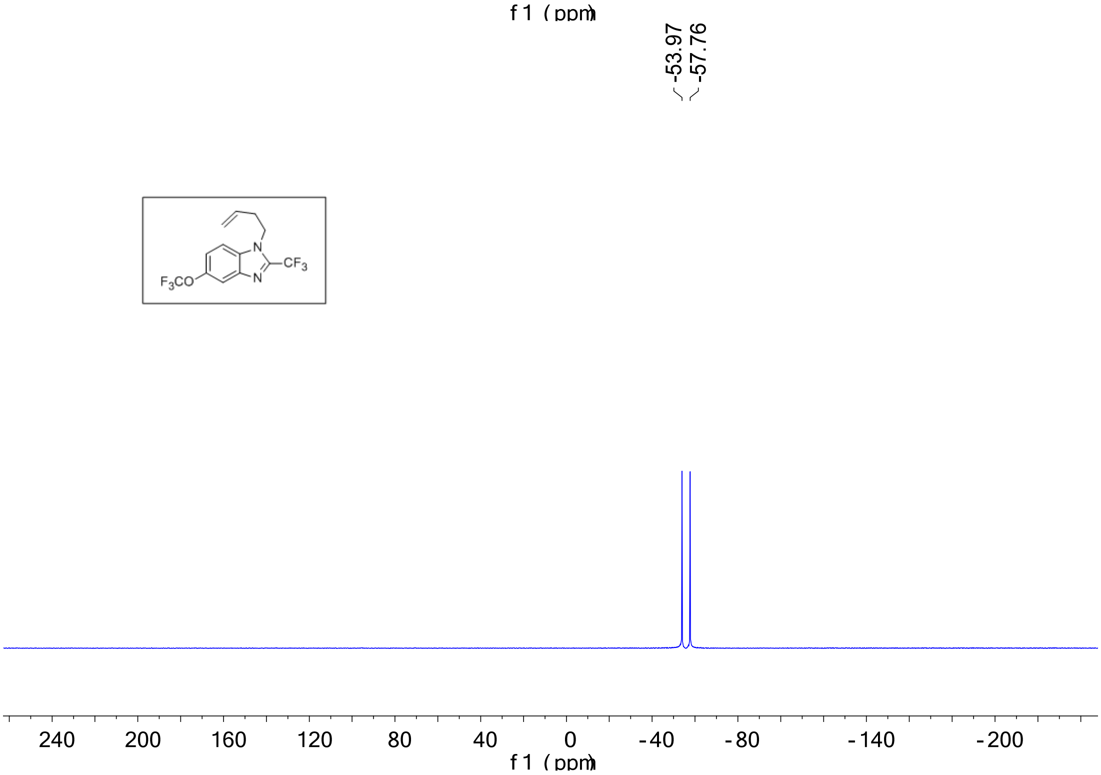

Supplementary Figure 16. ^{13}C and ^{19}F NMR spectra of compound **1d** in $CDCl_3$.

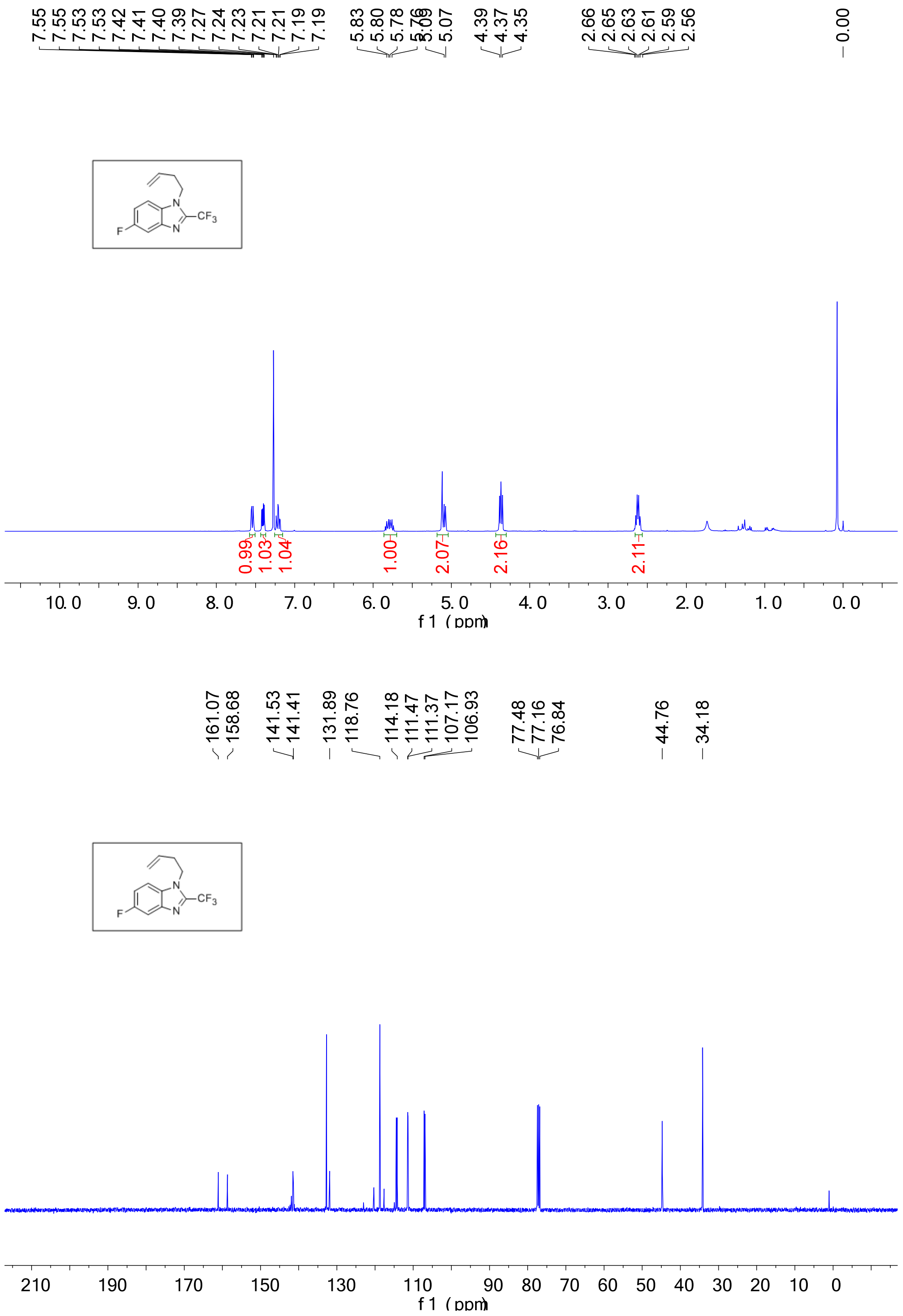

Supplementary Figure 17. ^{1}H and ^{13}C NMR spectra of compound **1e** in $CDCl_3$.

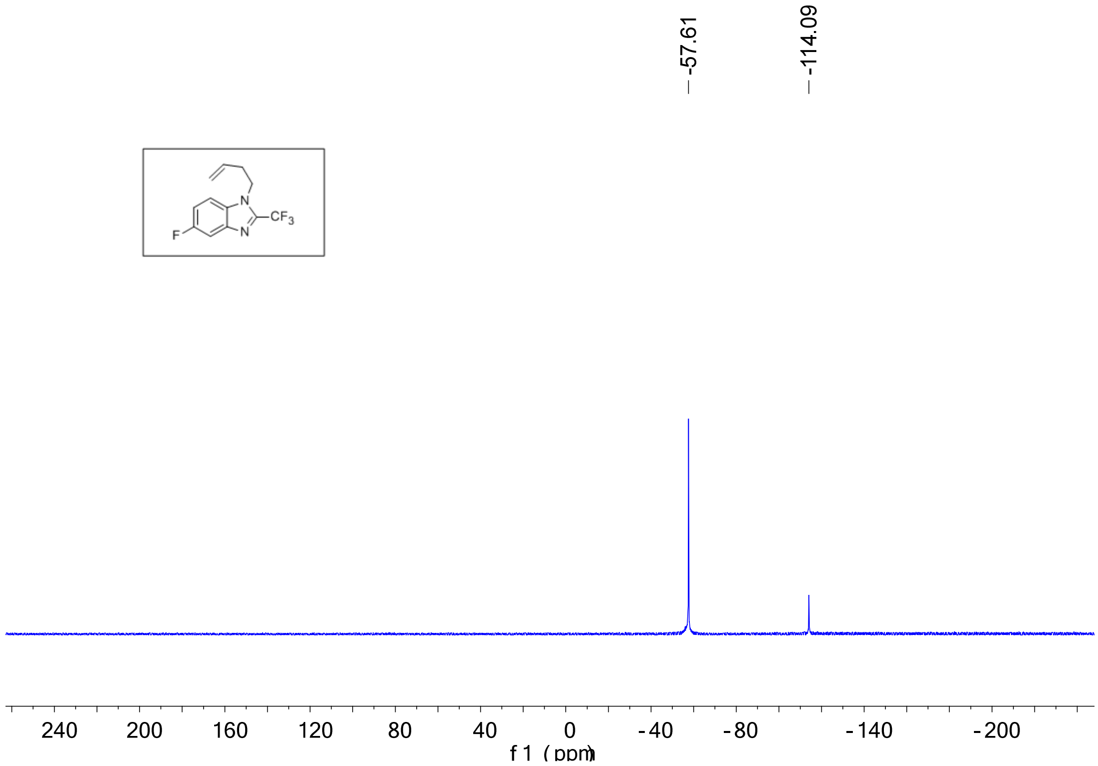

Supplementary Figure 18. ^{19}F NMR spectrum of compound **1e** in $CDCl_3$.

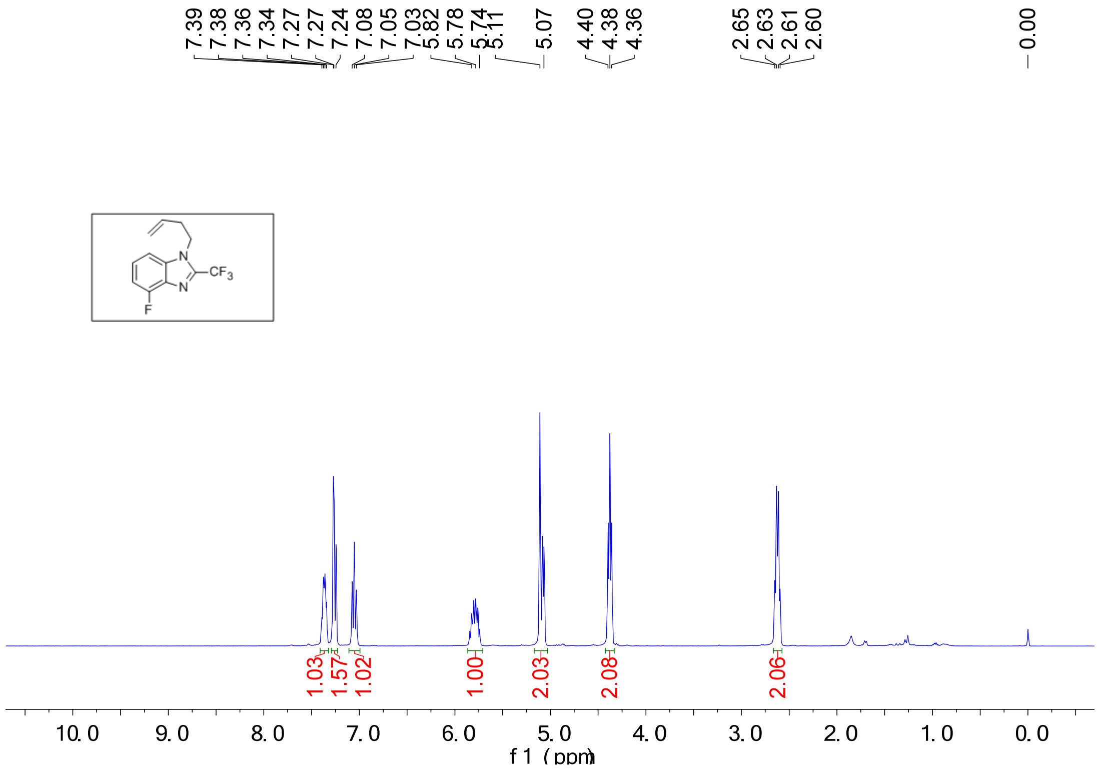

Supplementary Figure 19. ^{1}H spectrum of compound **1f** in $CDCl_3$.

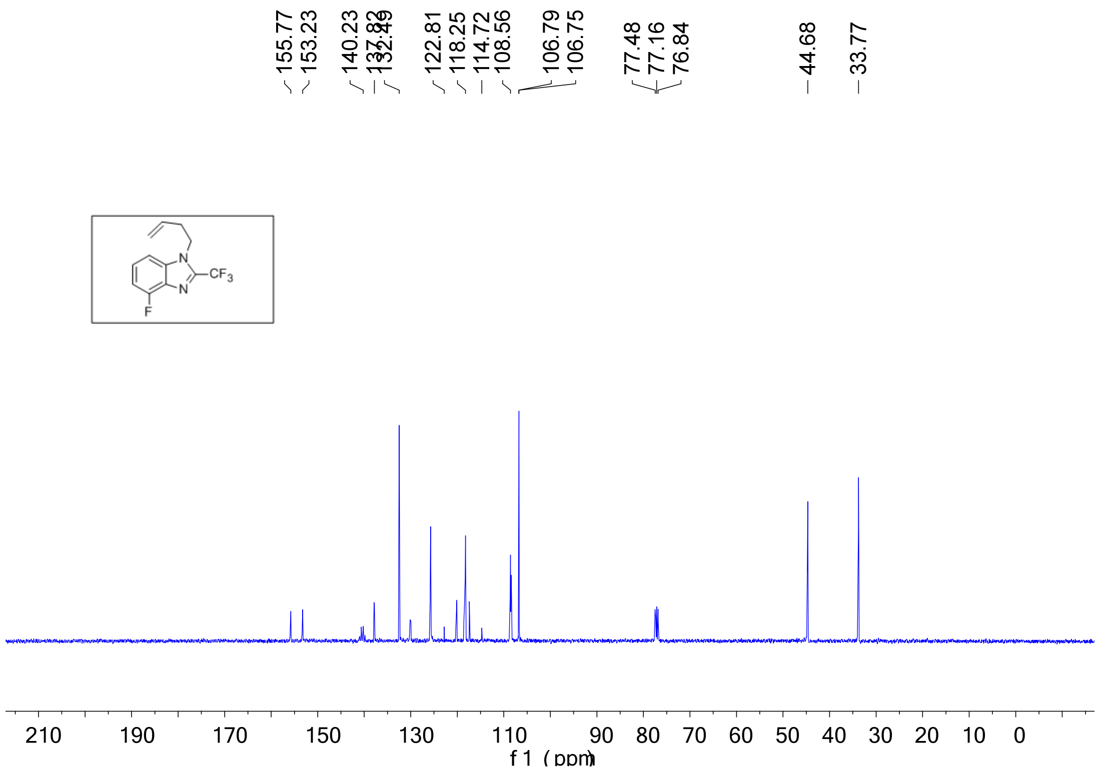

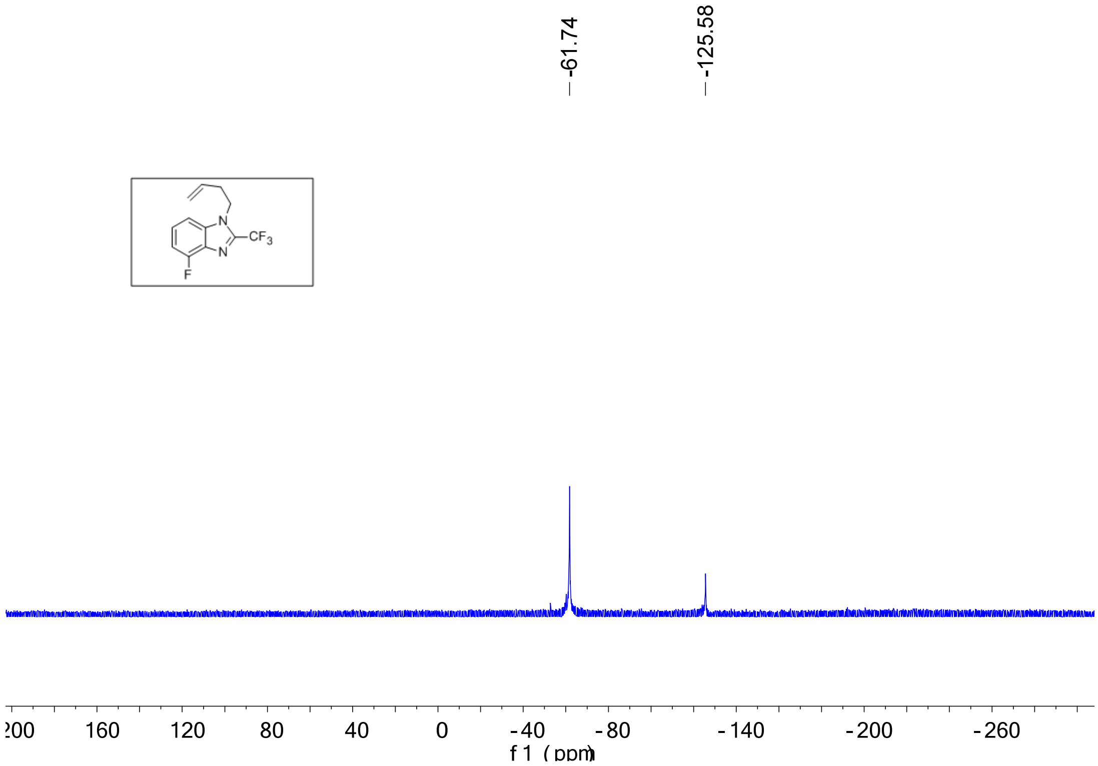

Supplementary Figure 20. ^{13}C and ^{19}F NMR spectra of compound **1f** in $CDCl_3$.

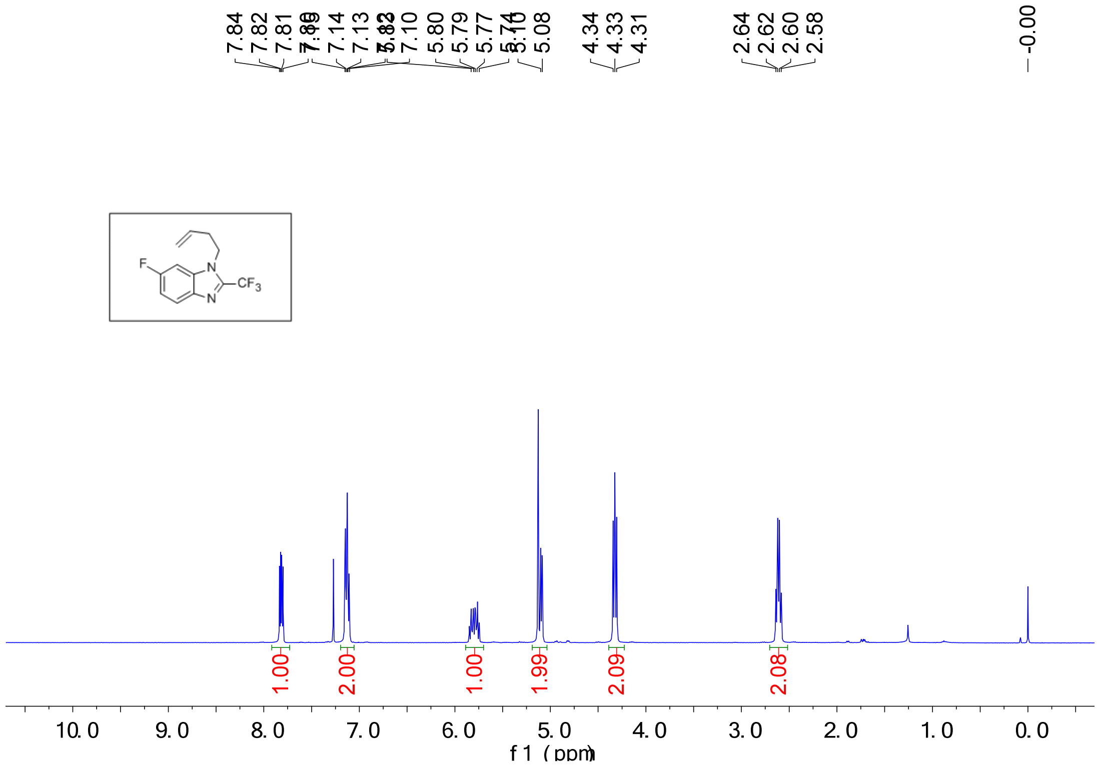

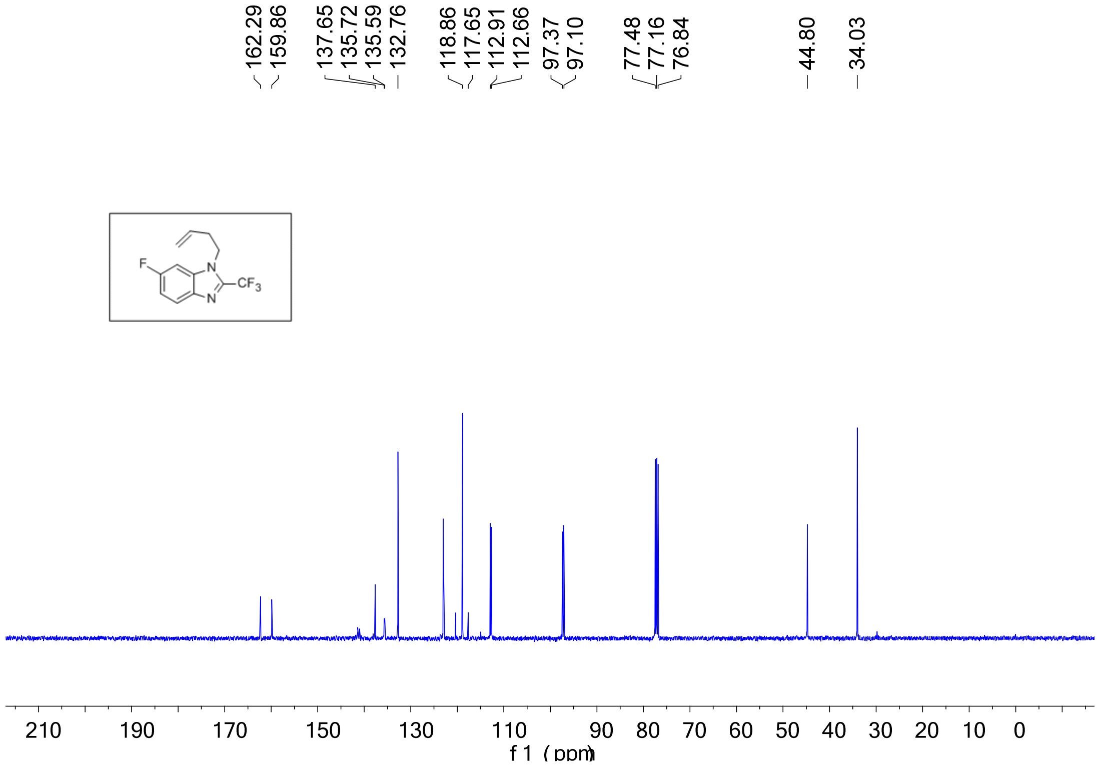

Supplementary Figure 21. ^{1}H and ^{13}C NMR spectra of compound **1g** in $CDCl_3$.

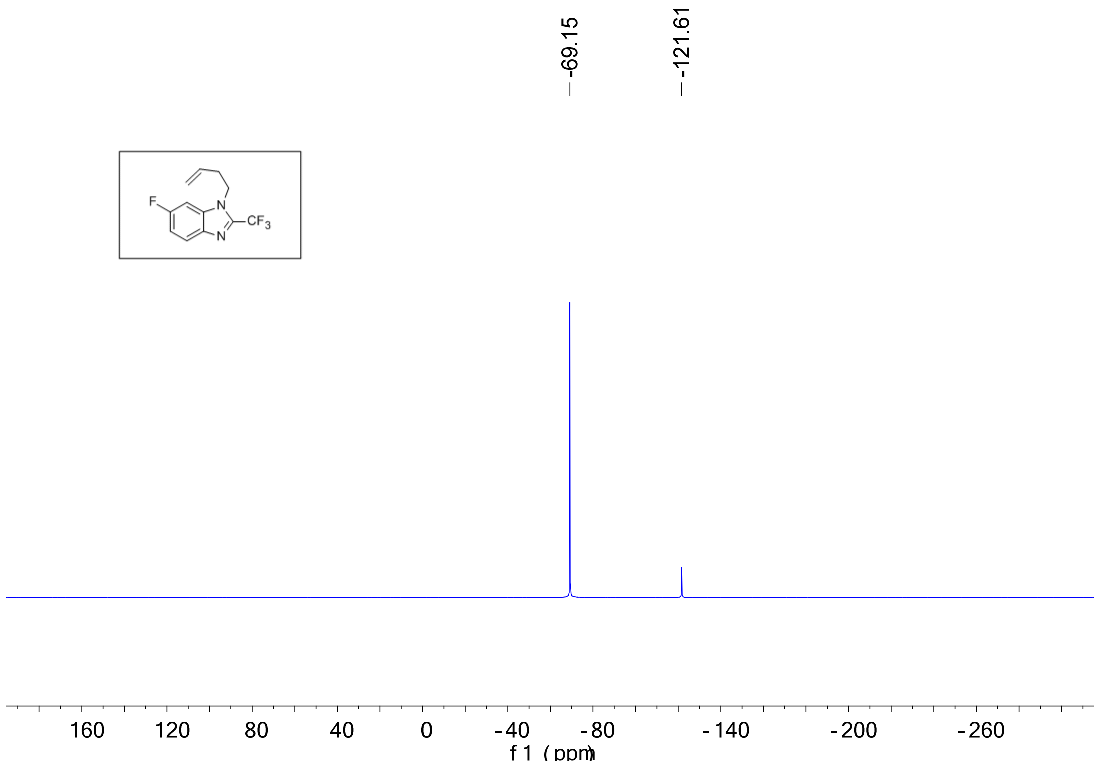

Supplementary Figure 22. ^{19}F NMR spectrum of compound **1g** in $CDCl_3$.

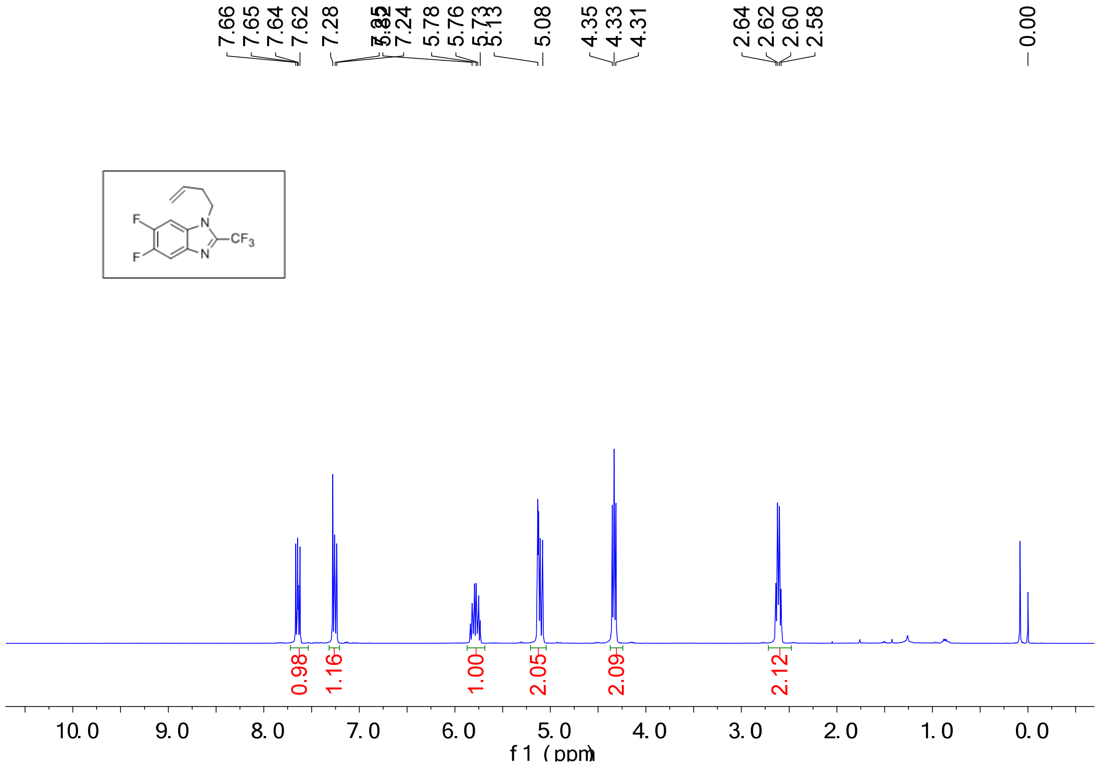

Supplementary Figure 23. ^{1}H NMR spectrum of compound **1h** in $CDCl_3$.

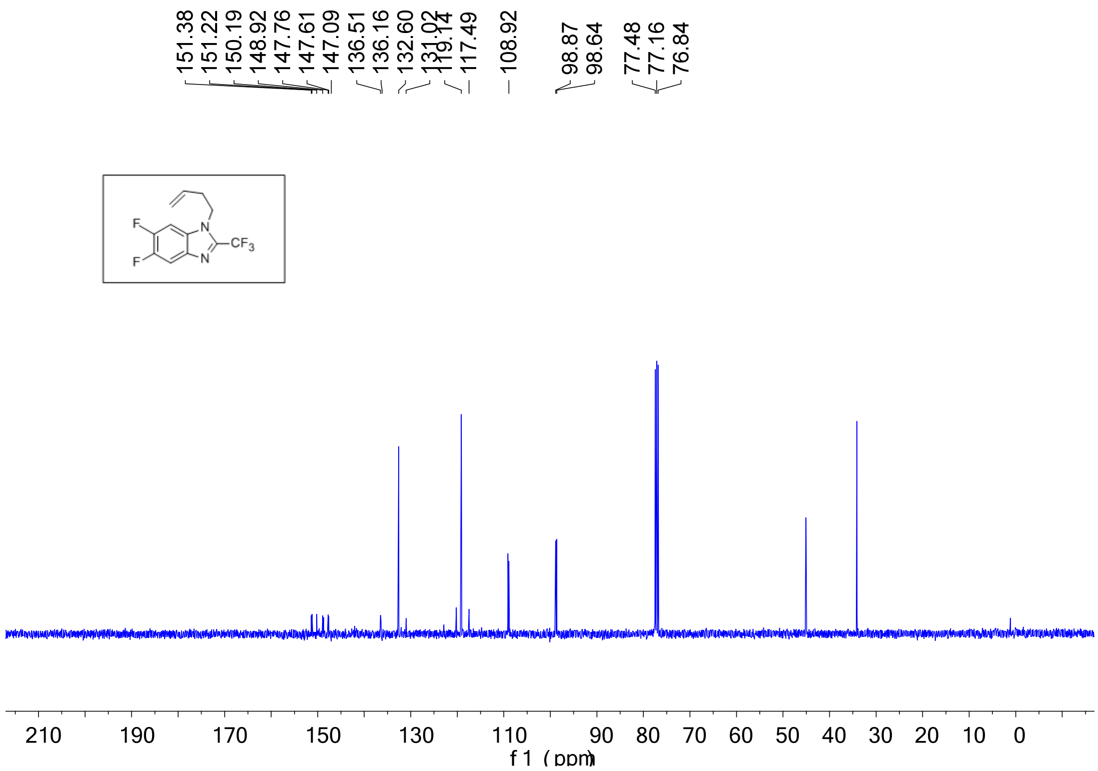

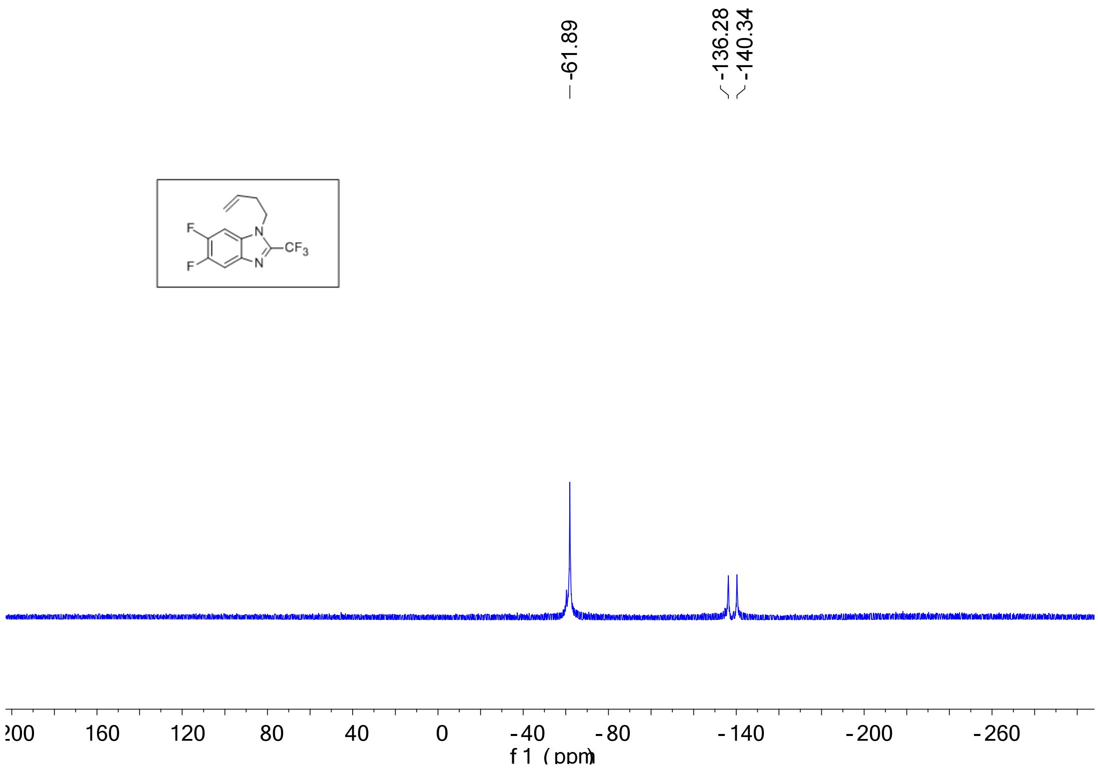

Supplementary Figure 24. ^{13}C and ^{19}F NMR spectra of compound **1h** in $CDCl_3$.

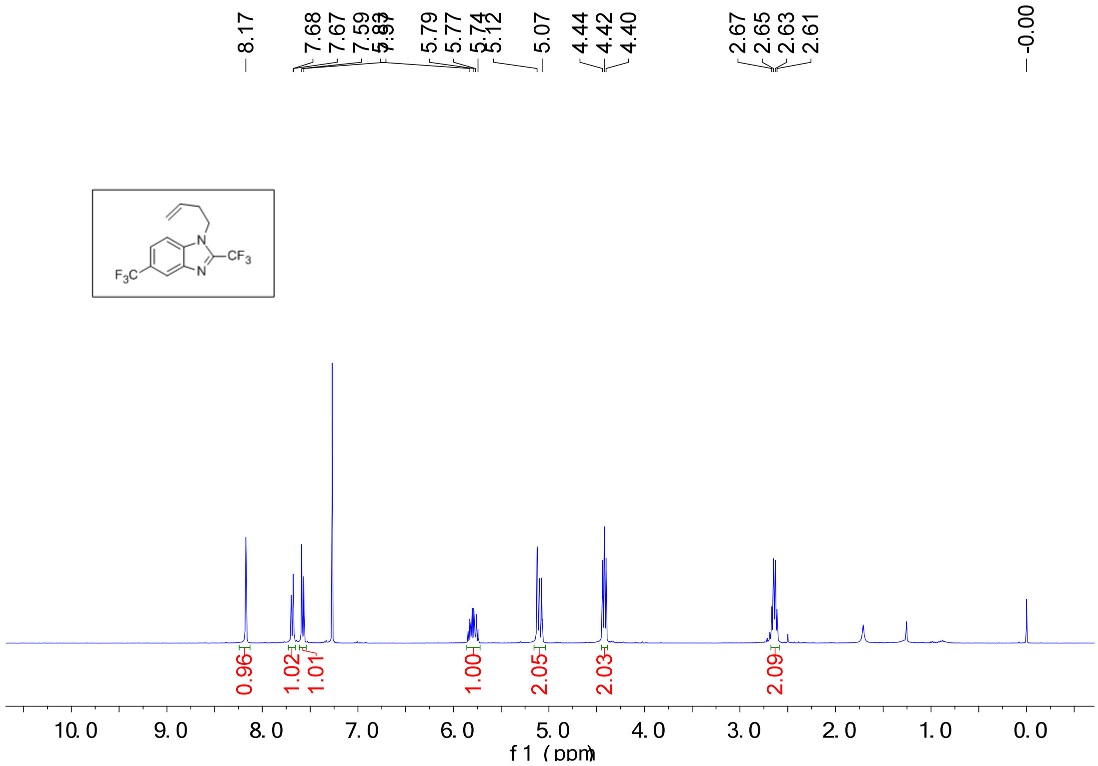

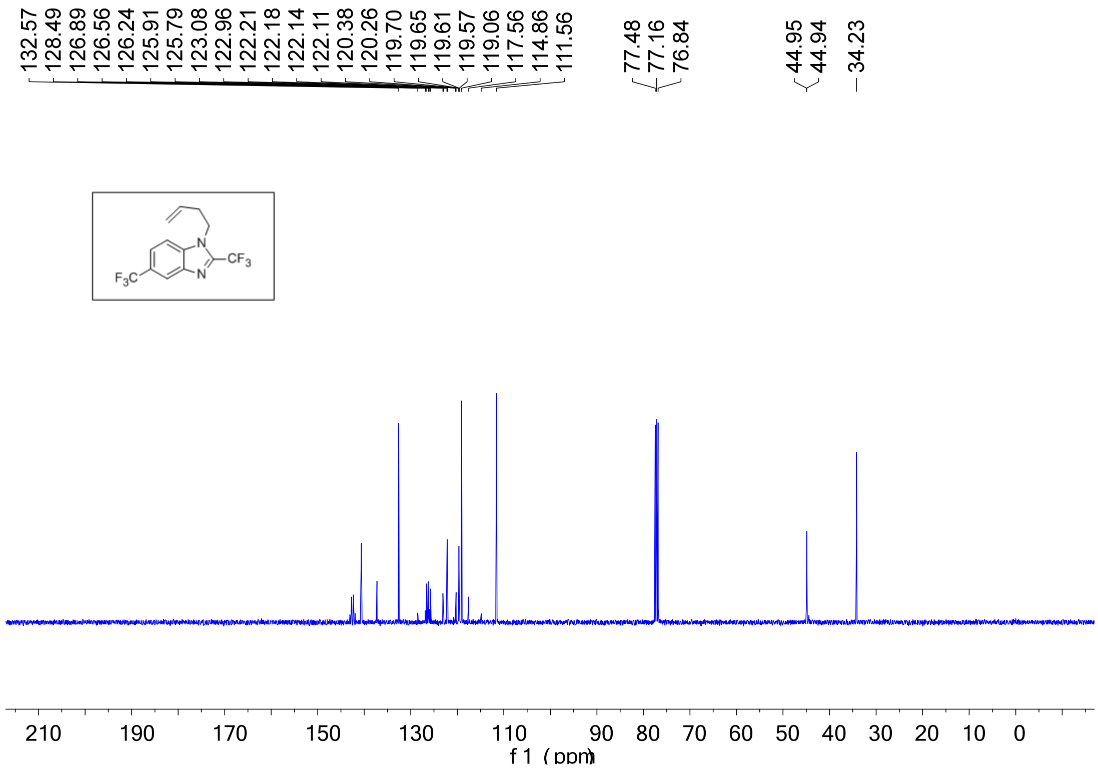

Supplementary Figure 25. ^{1}H and ^{13}C NMR spectra of compound **1i** in $CDCl_3$.

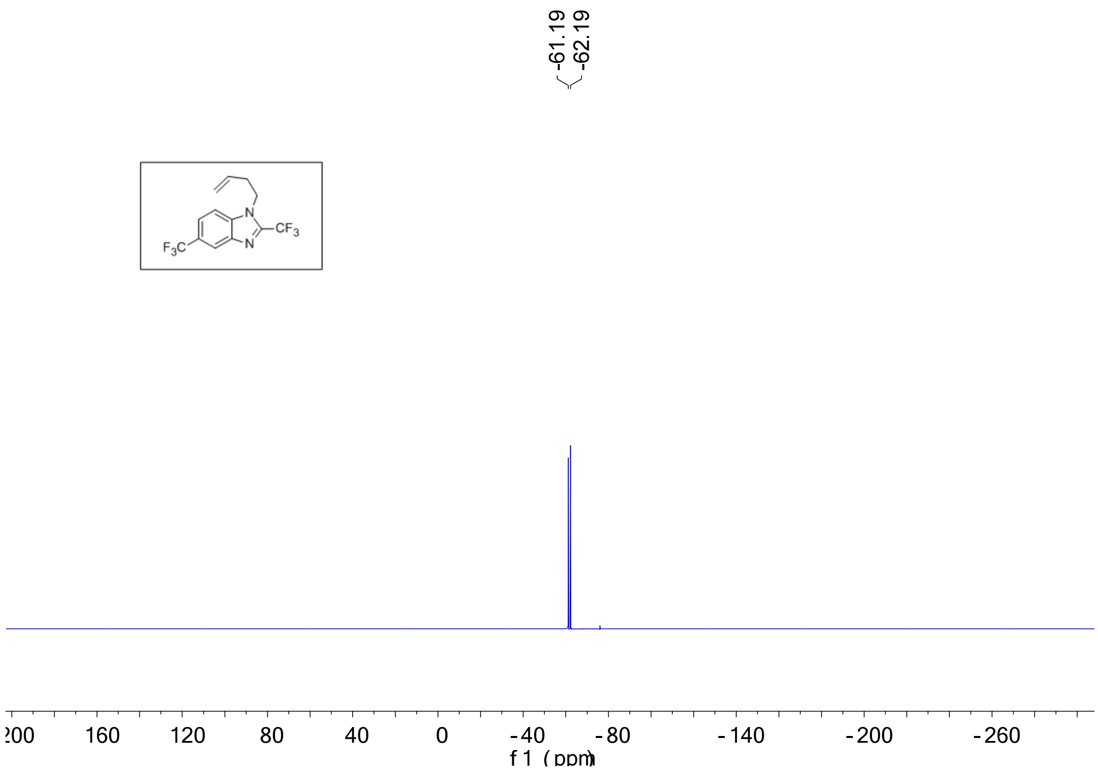

Supplementary Figure 26. ^{19}F NMR spectrum of compound **1i** in $CDCl_3$.

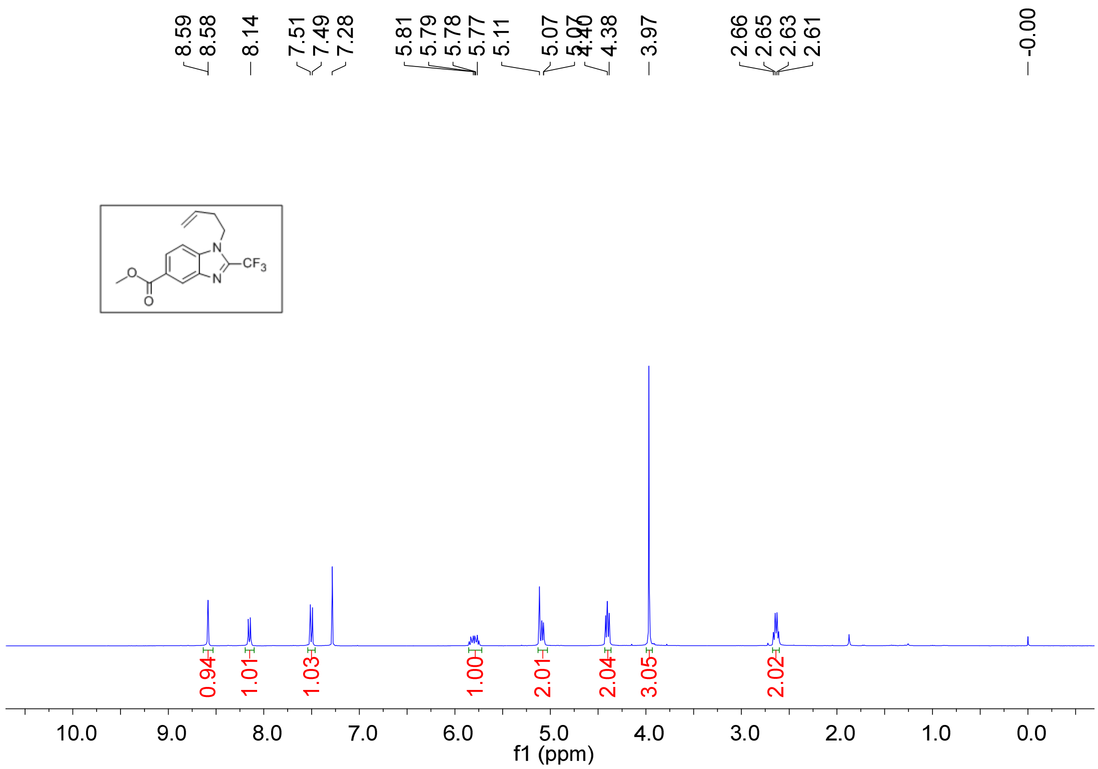

Supplementary Figure 27. ^{1}H NMR spectrum of compound **1j** in $CDCl_3$.

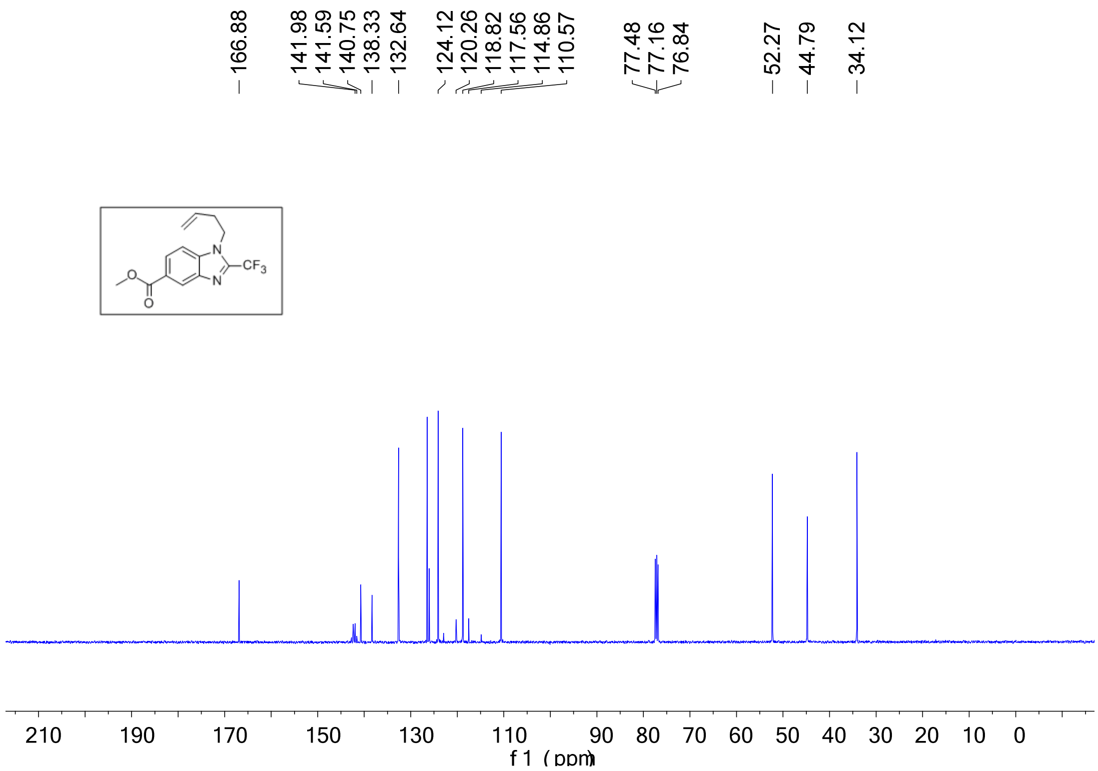

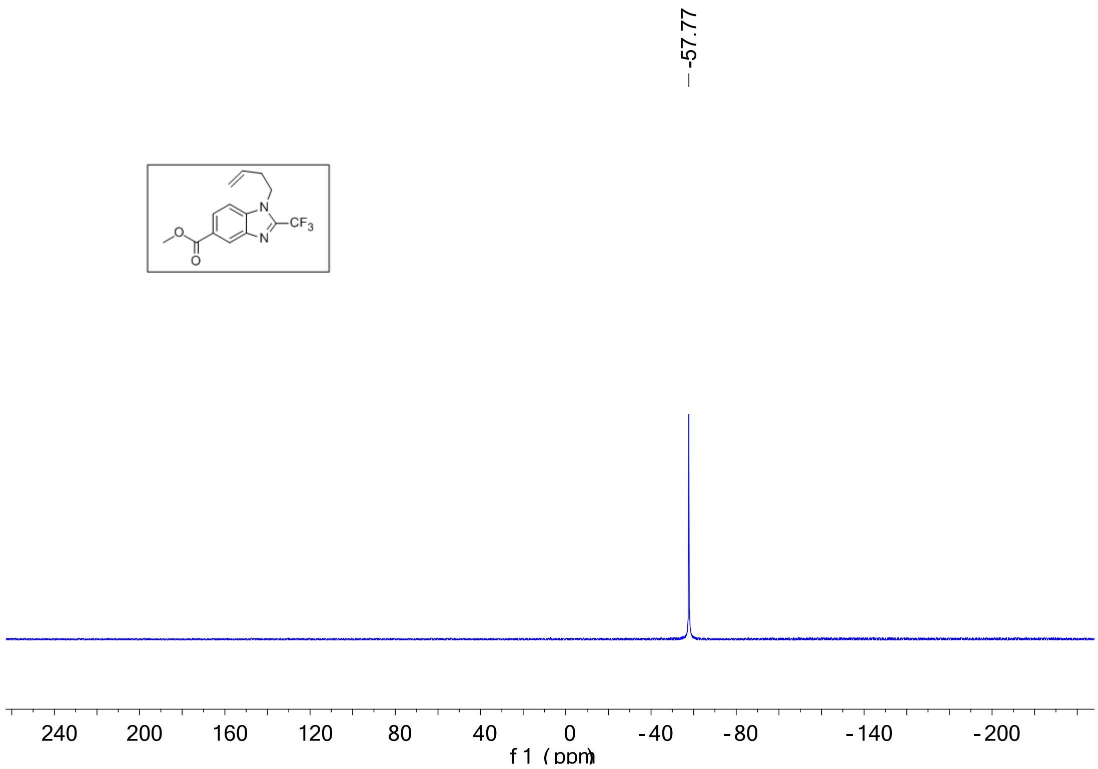

Supplementary Figure 28. ^{13}C and ^{19}F NMR spectra of compound **1j** in $CDCl_3$.

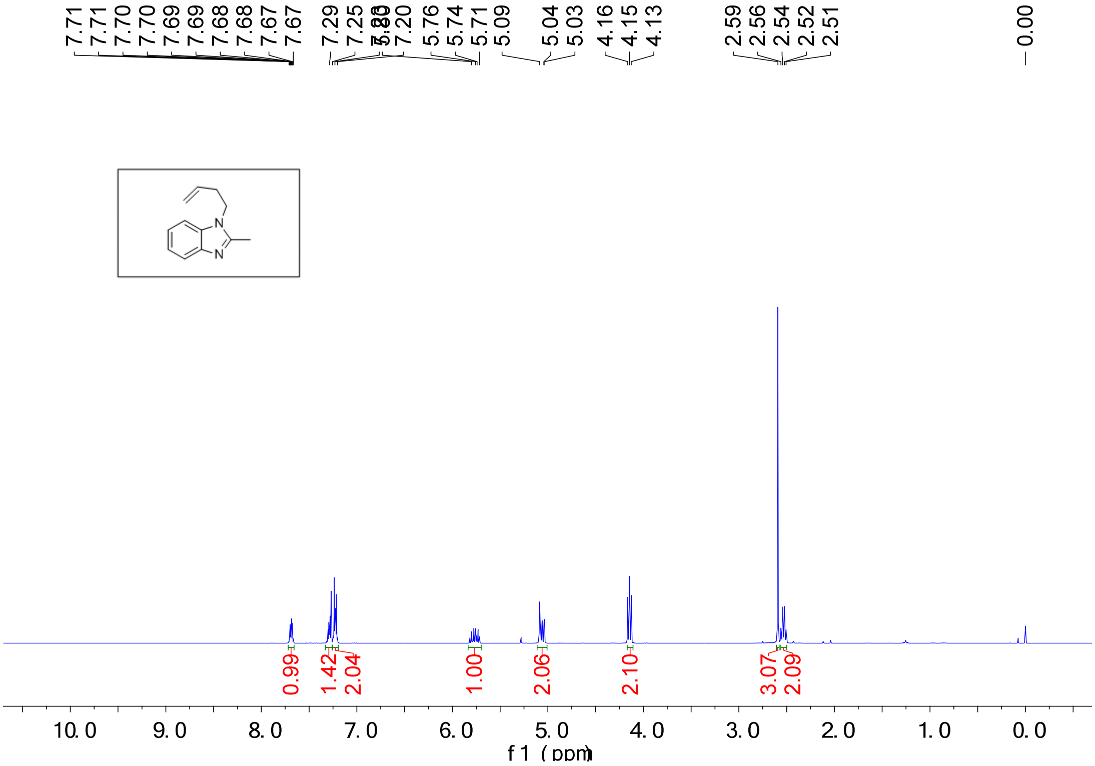

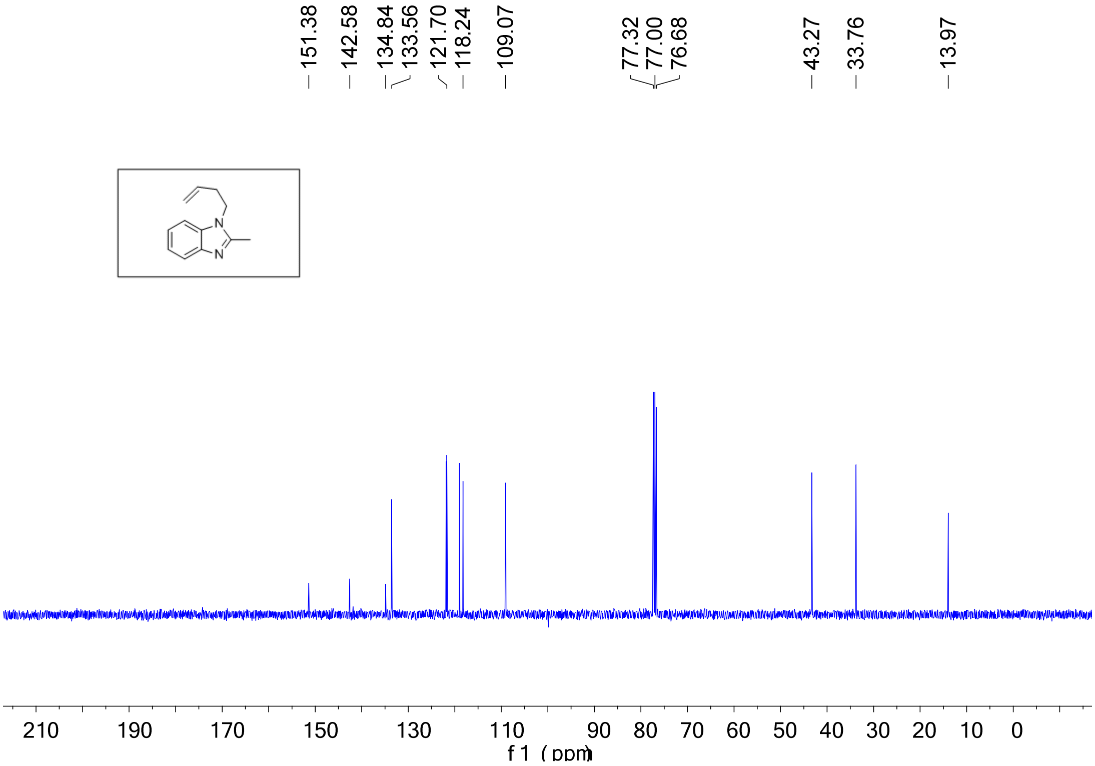

Supplementary Figure 29. ^{1}H and ^{13}C NMR spectra of compound **1k** in $CDCl_3$

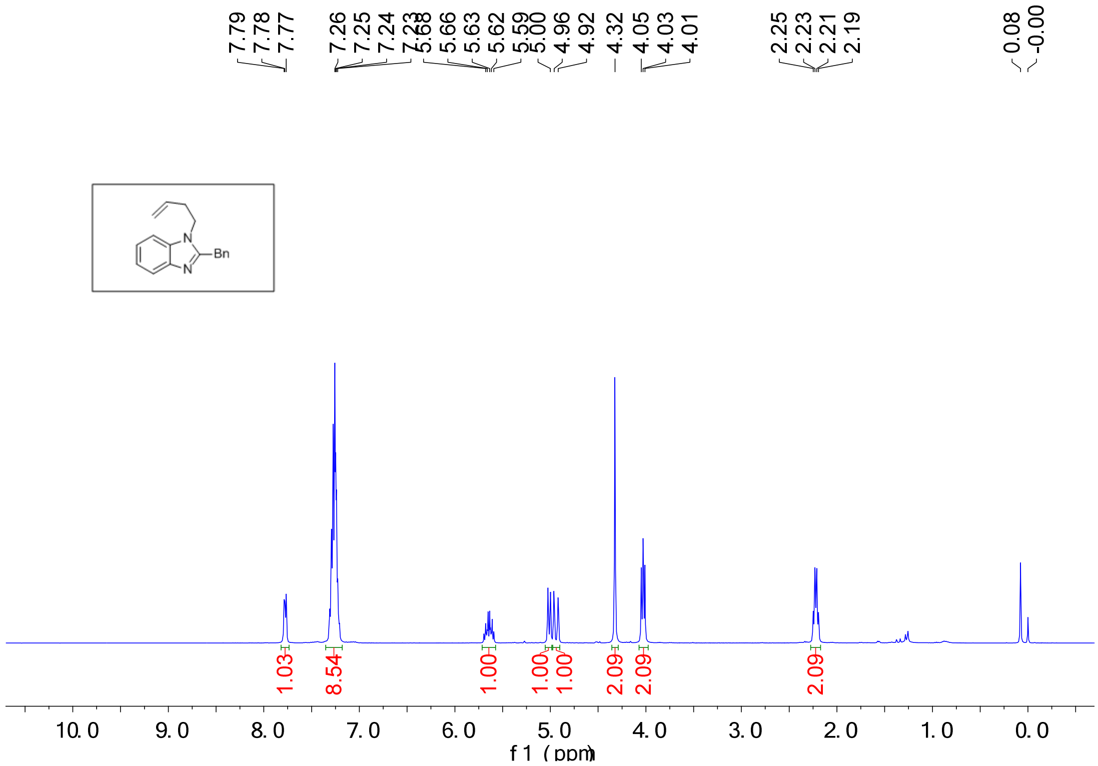

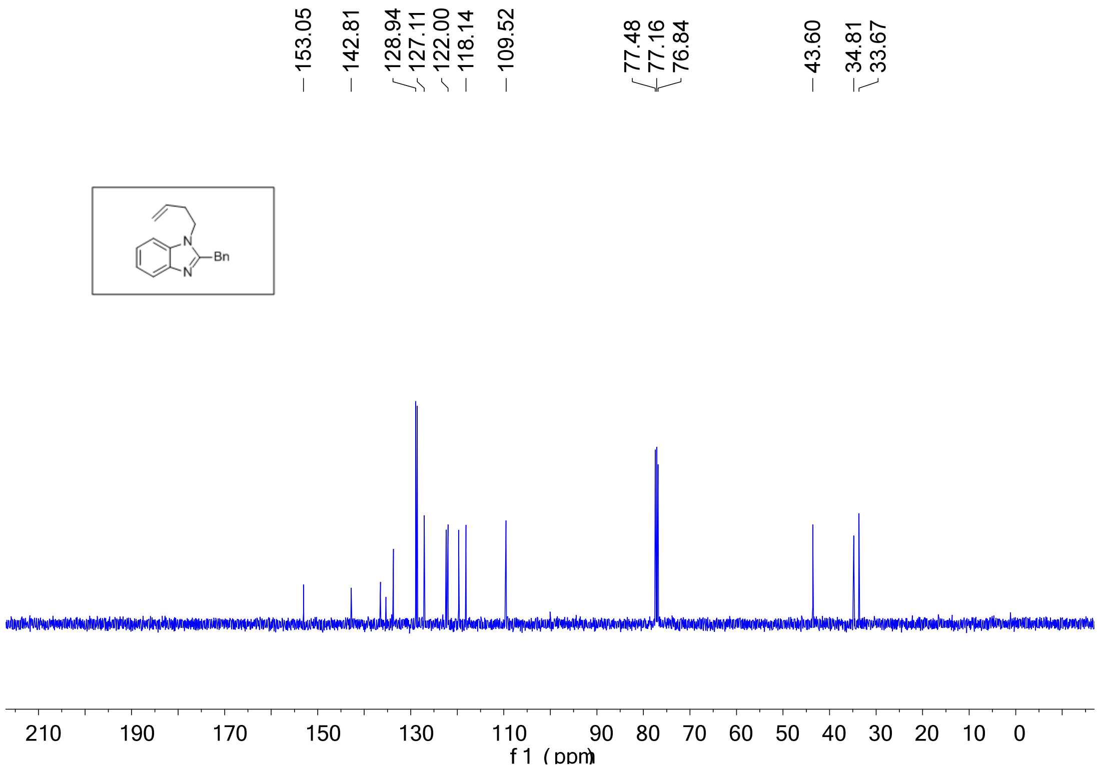

Supplementary Figure 30. ^{1}H and ^{13}C NMR spectra of compound **1l** in $CDCl_3$

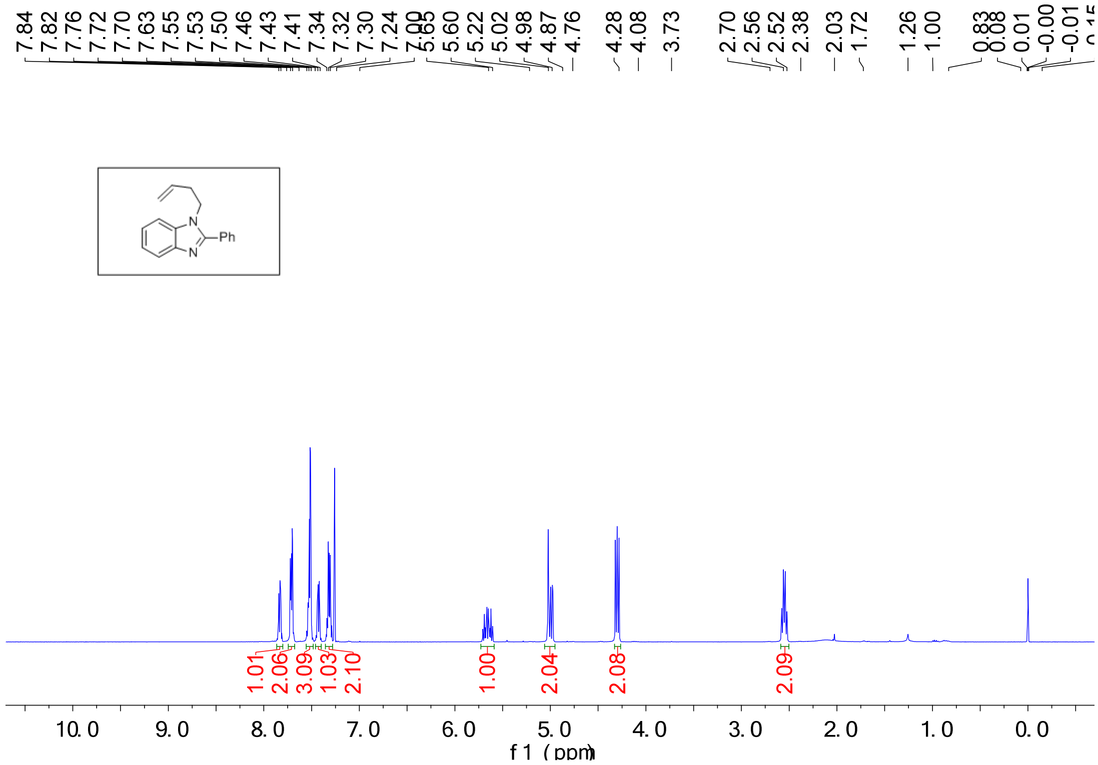

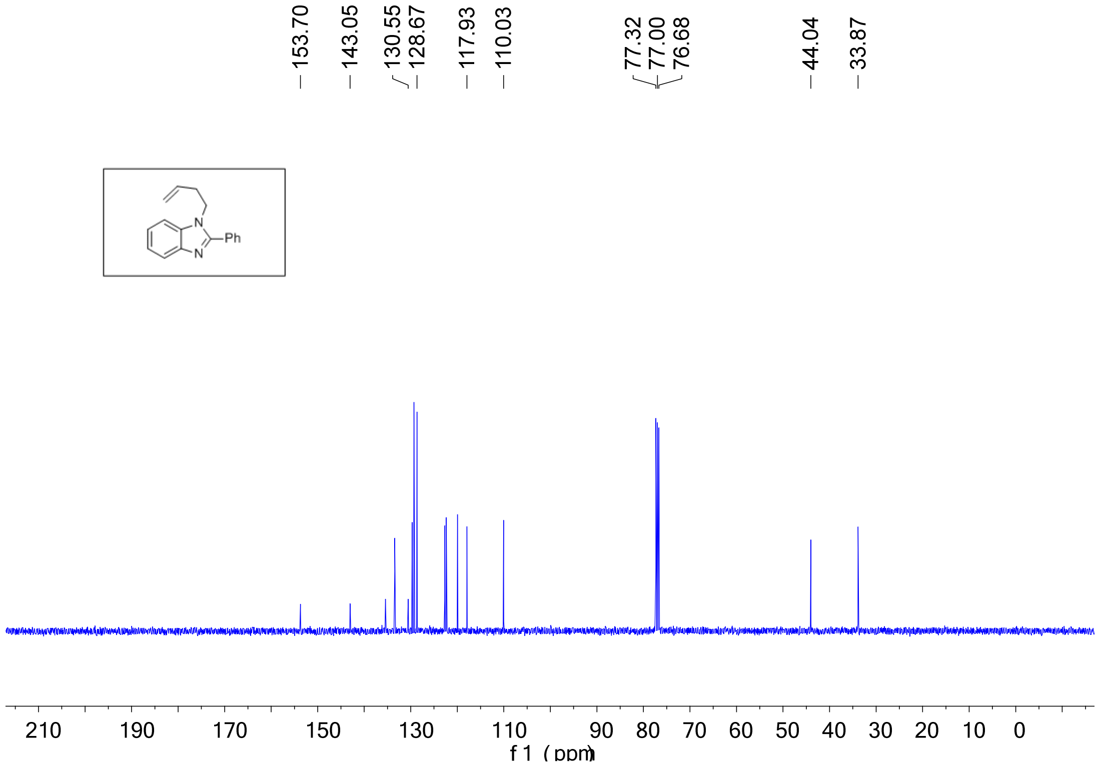

Supplementary Figure 31. ^{1}H and ^{13}C NMR spectra of compound **1m** in $CDCl_3$

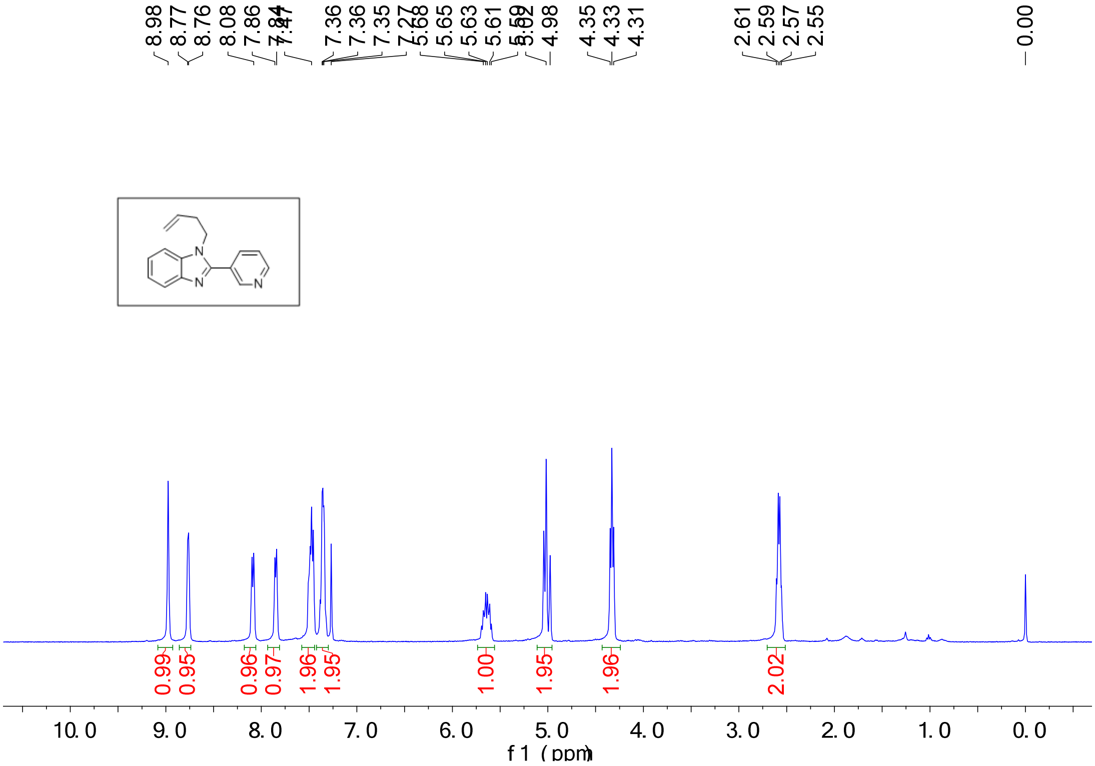

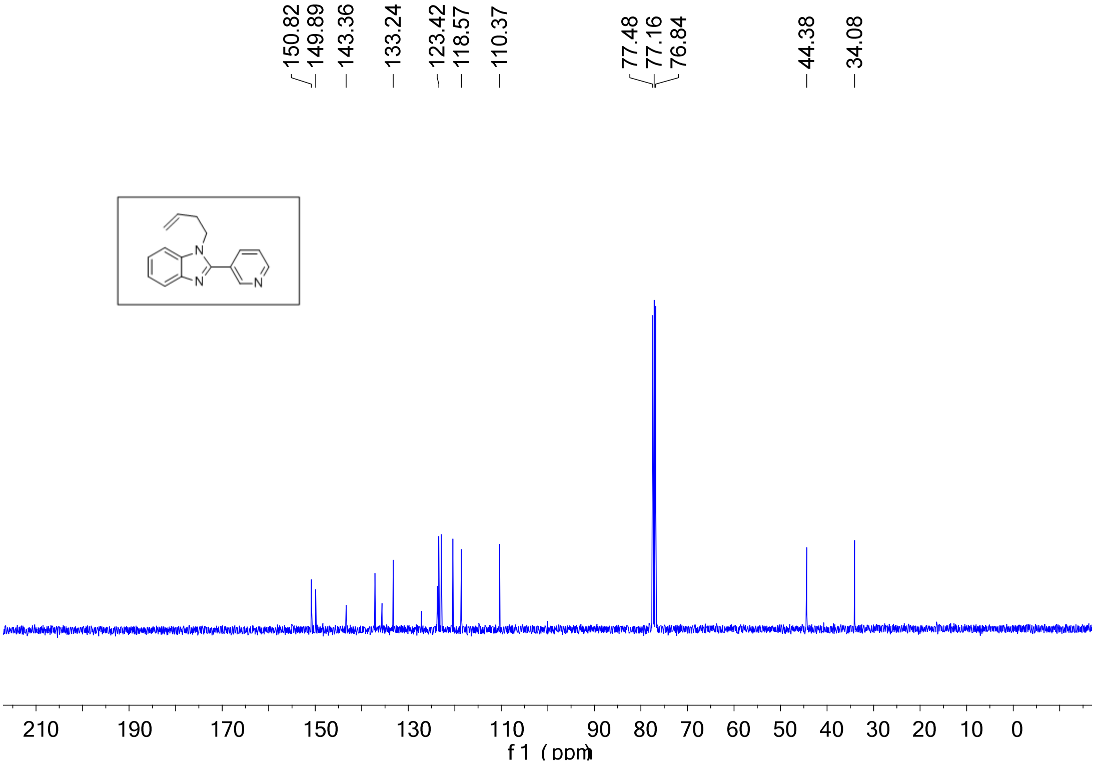

Supplementary Figure 32. ^{1}H and ^{13}C NMR spectra of compound **1n** in $CDCl_3$

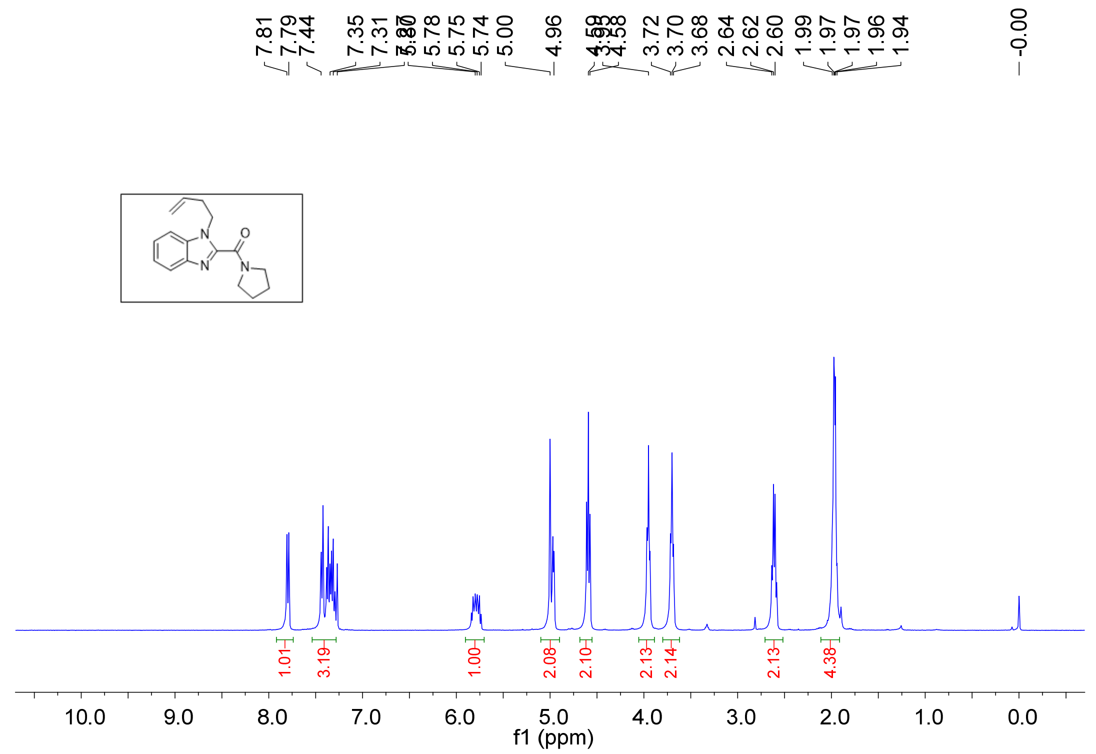

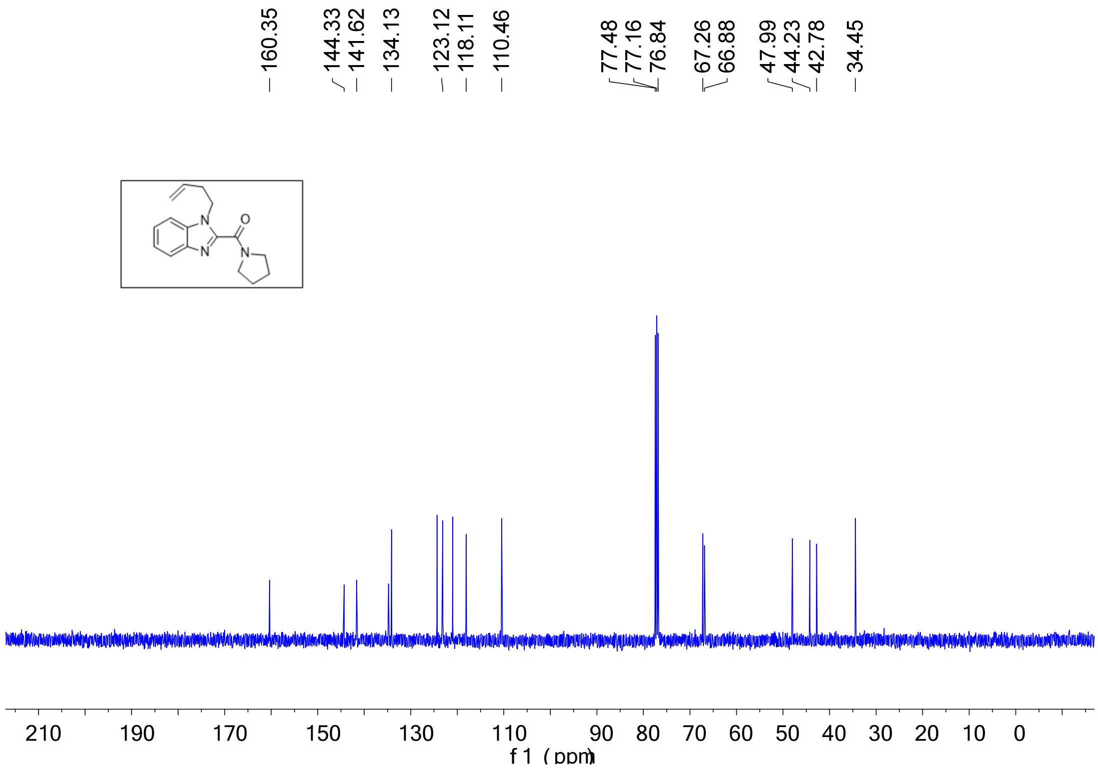

Supplementary Figure 33. ^{1}H and ^{13}C NMR spectra of compound **1o** in $CDCl_3$

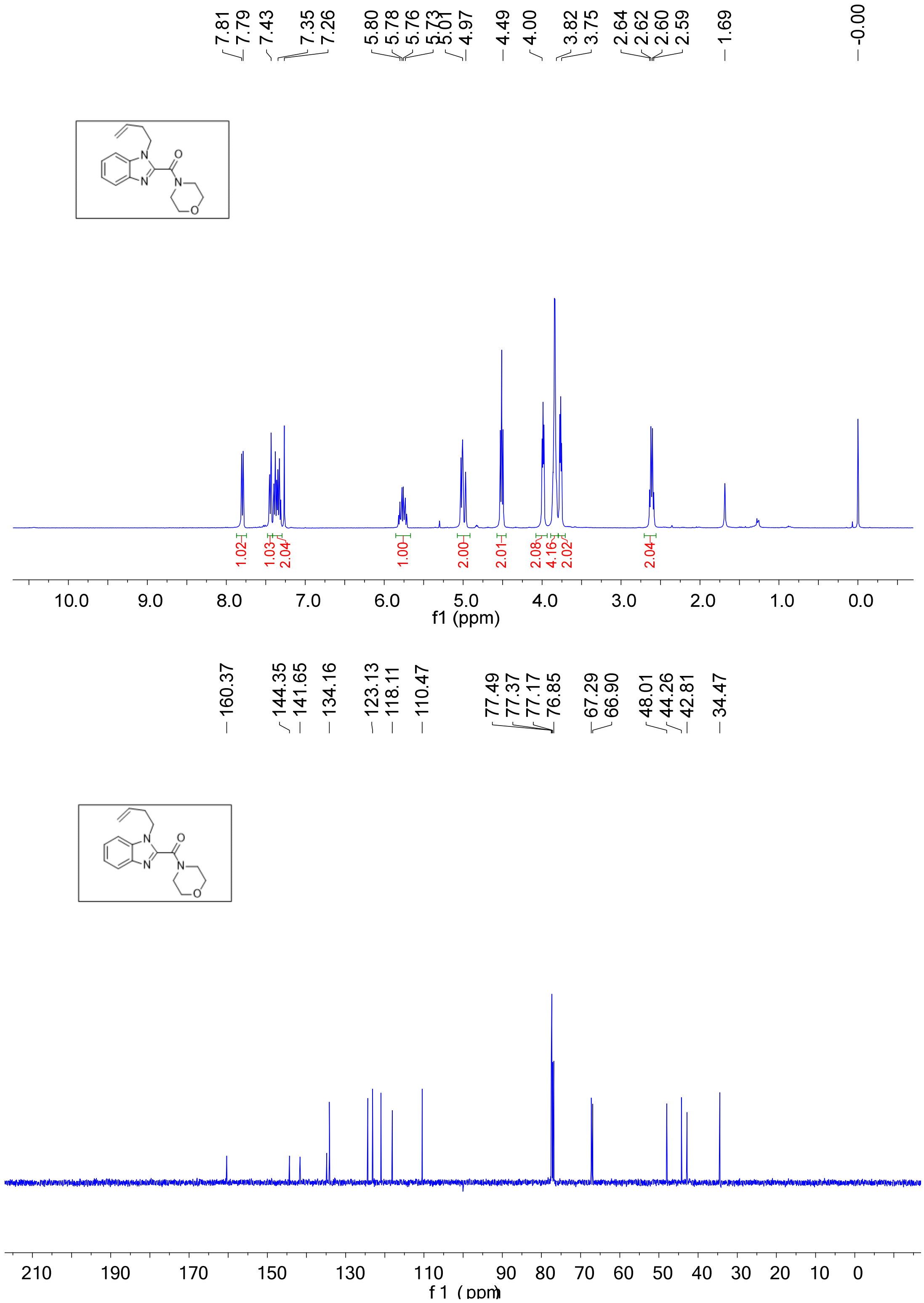

Supplementary Figure 34. ^{1}H and ^{13}C NMR spectra of compound **1p** in $CDCl_3$

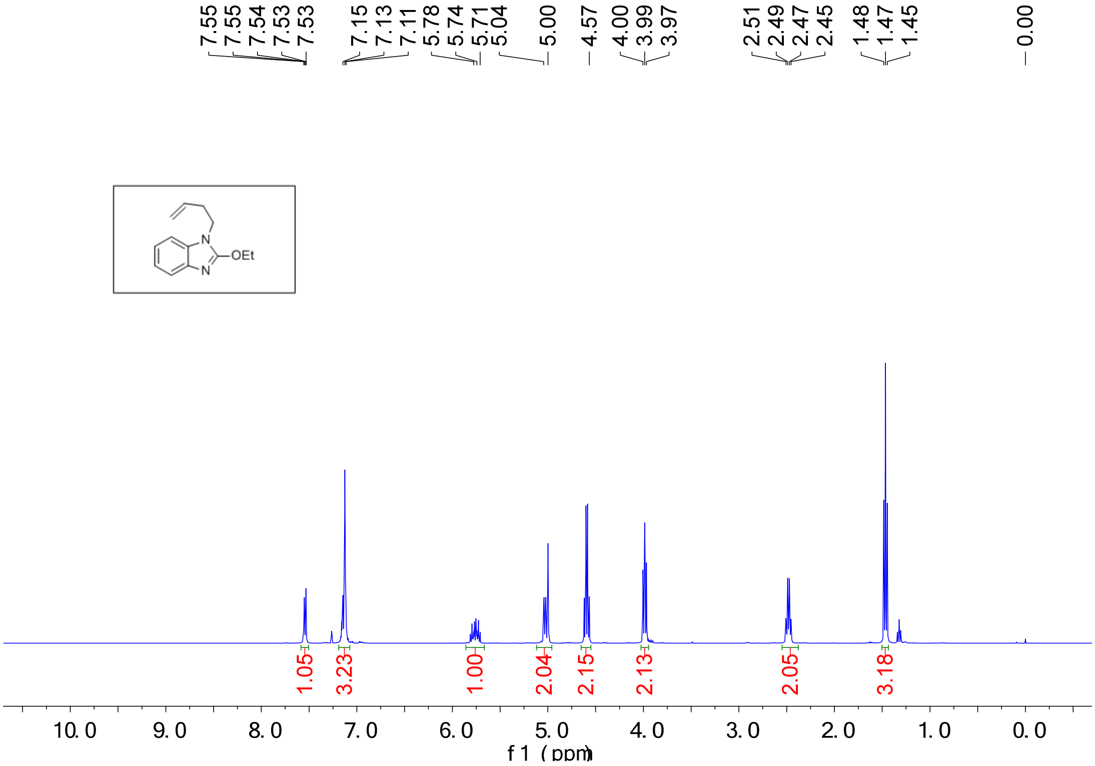

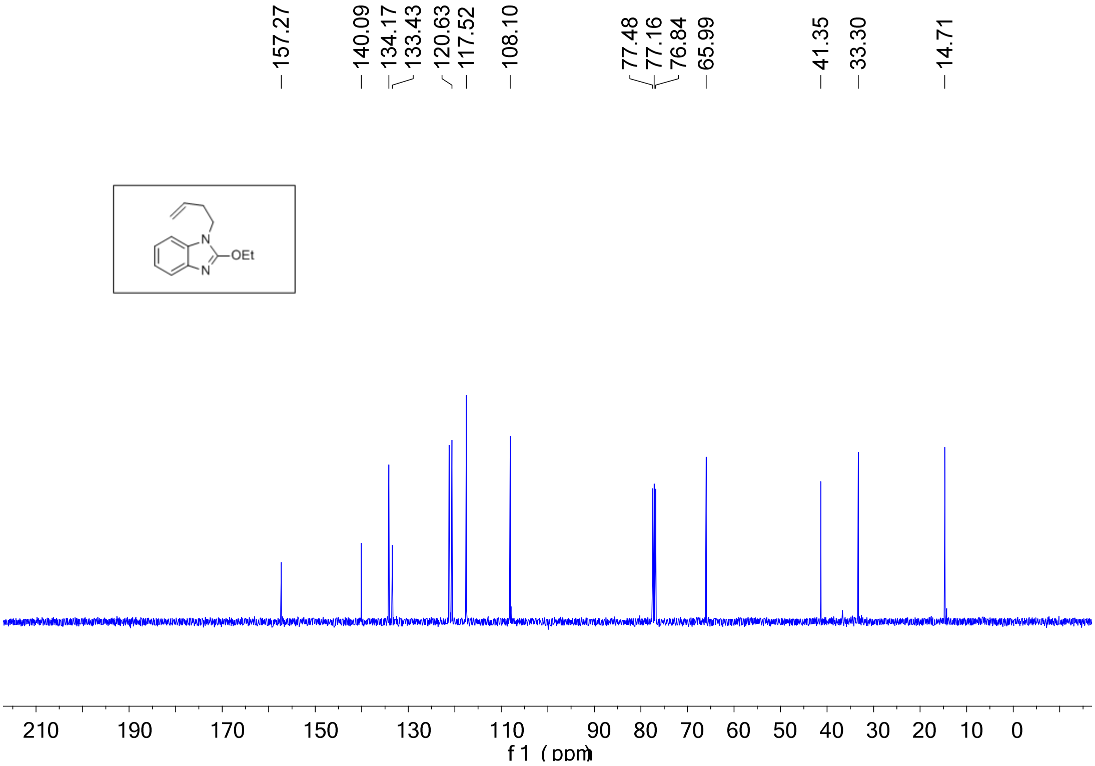

Supplementary Figure 35. ^{1}H and ^{13}C NMR spectra of compound **1q** in $CDCl_3$

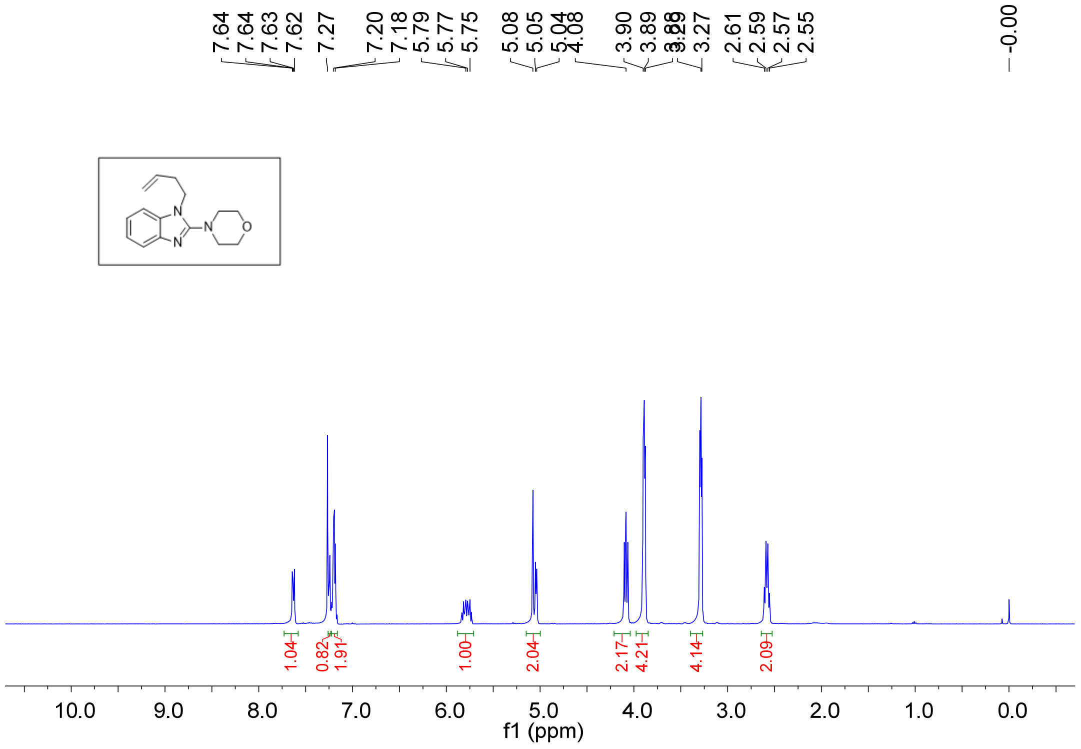

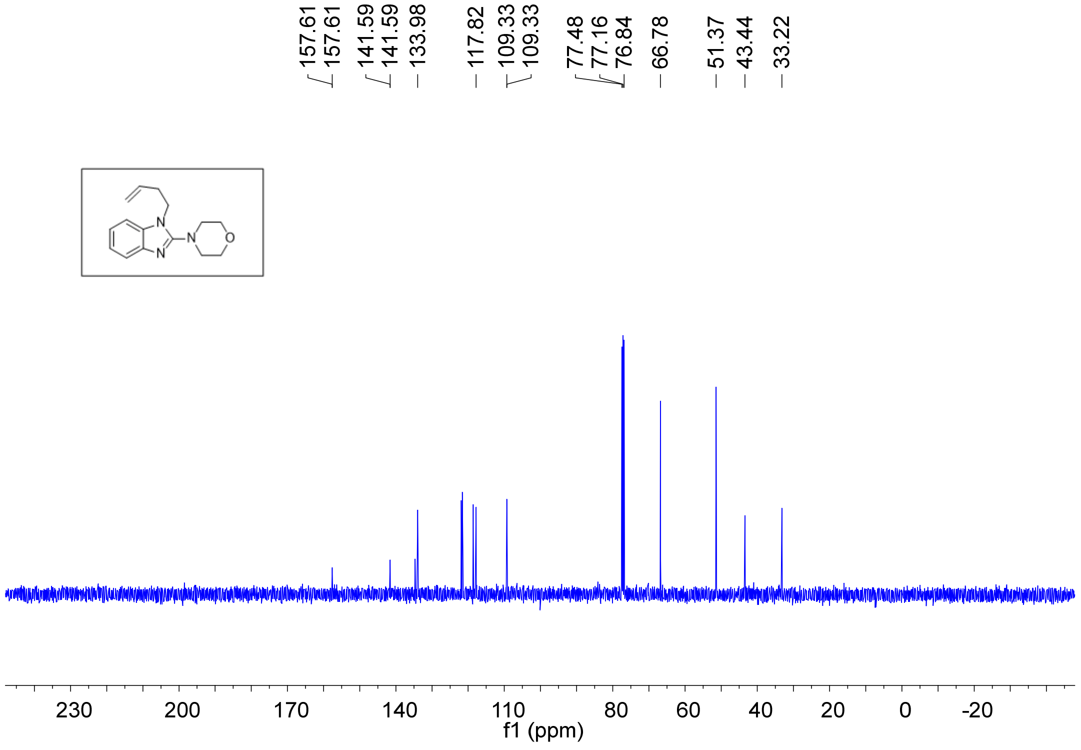

Supplementary Figure 36. ^{1}H and ^{13}C NMR spectra of compound **1r** in $CDCl_3$

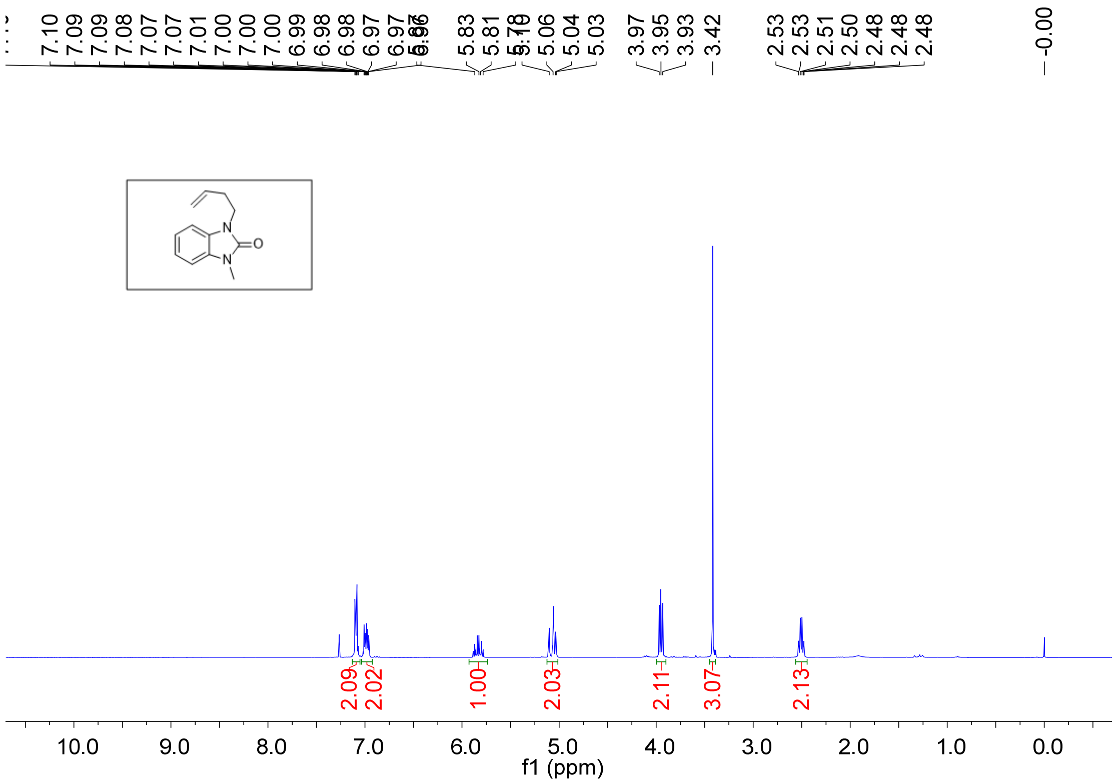

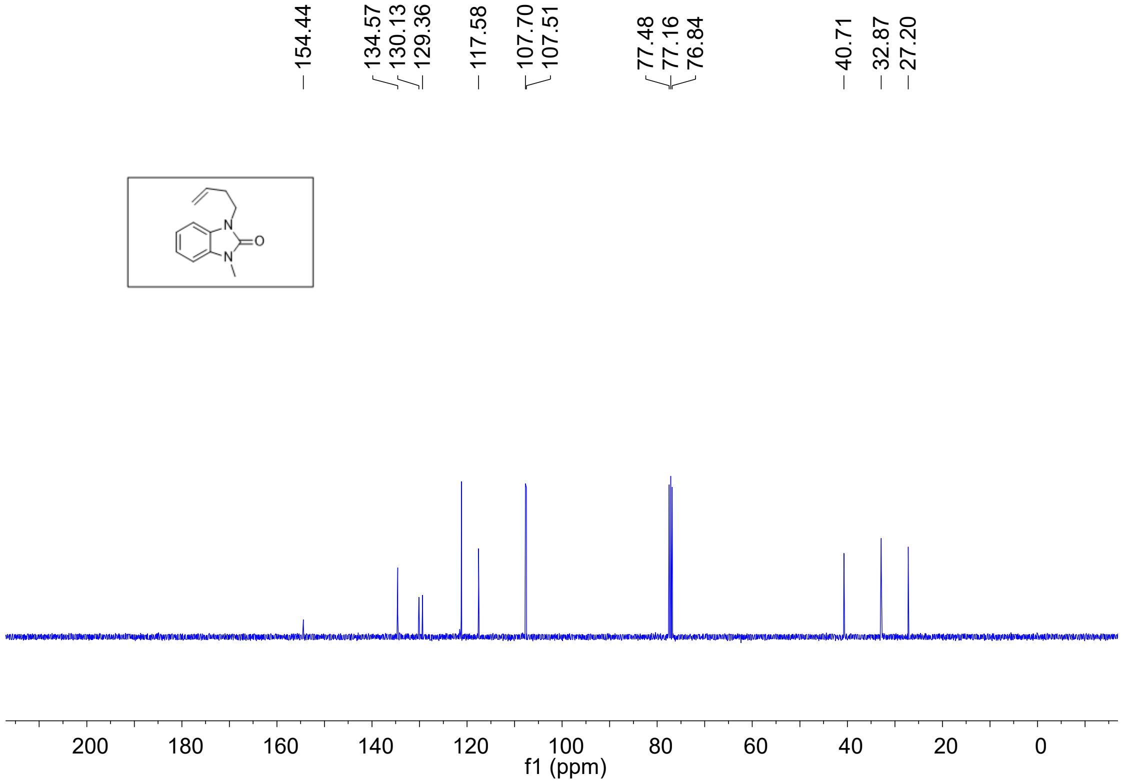

Supplementary Figure 37. ^{1}H and ^{13}C NMR spectra of compound **1s** in $CDCl_3$

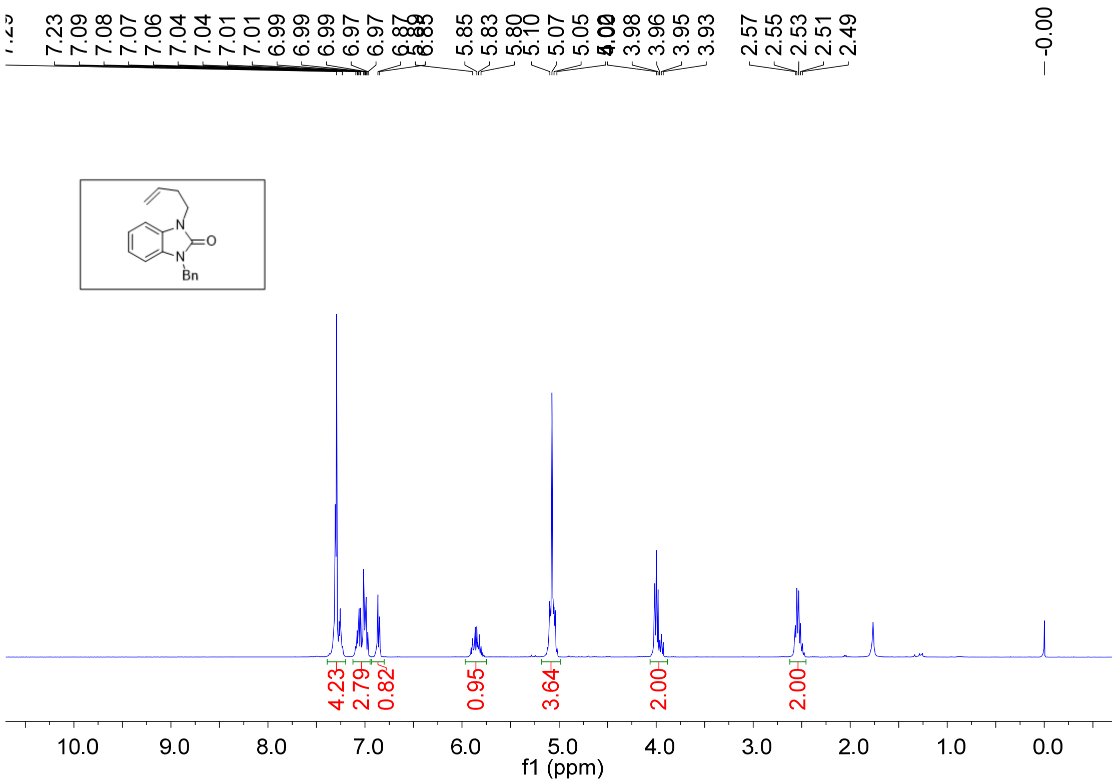

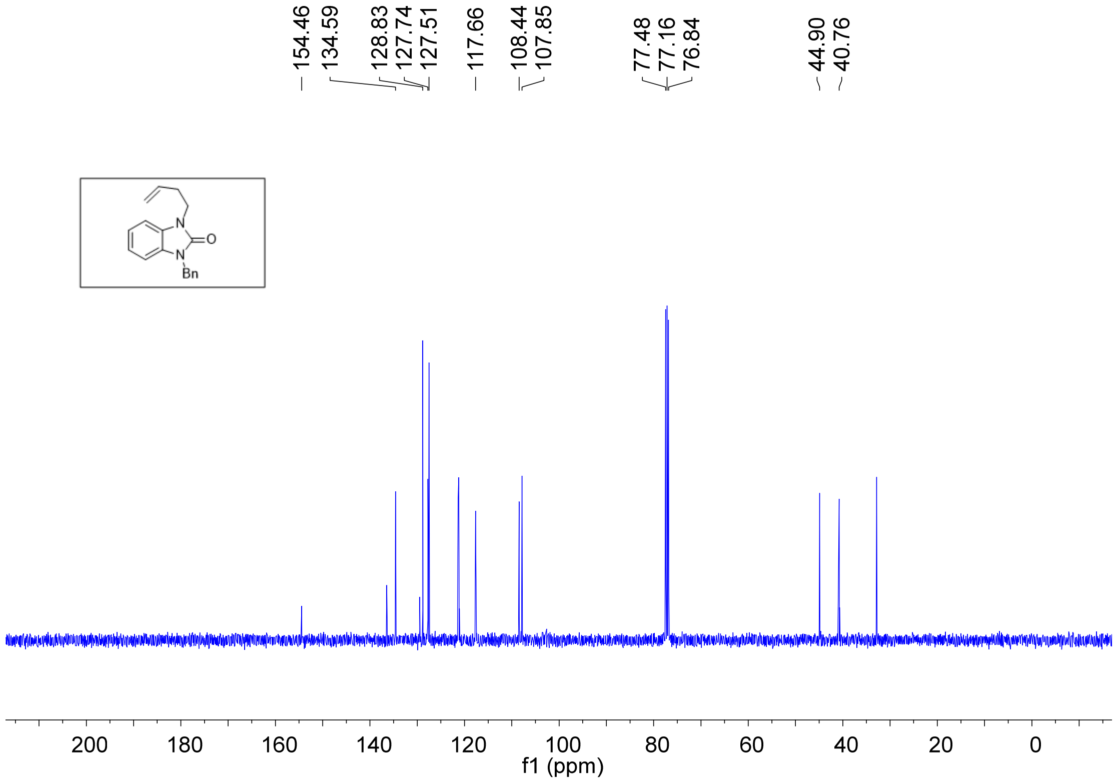

Supplementary Figure 38. ^{1}H and ^{13}C NMR spectra of compound **1t** in $CDCl_3$

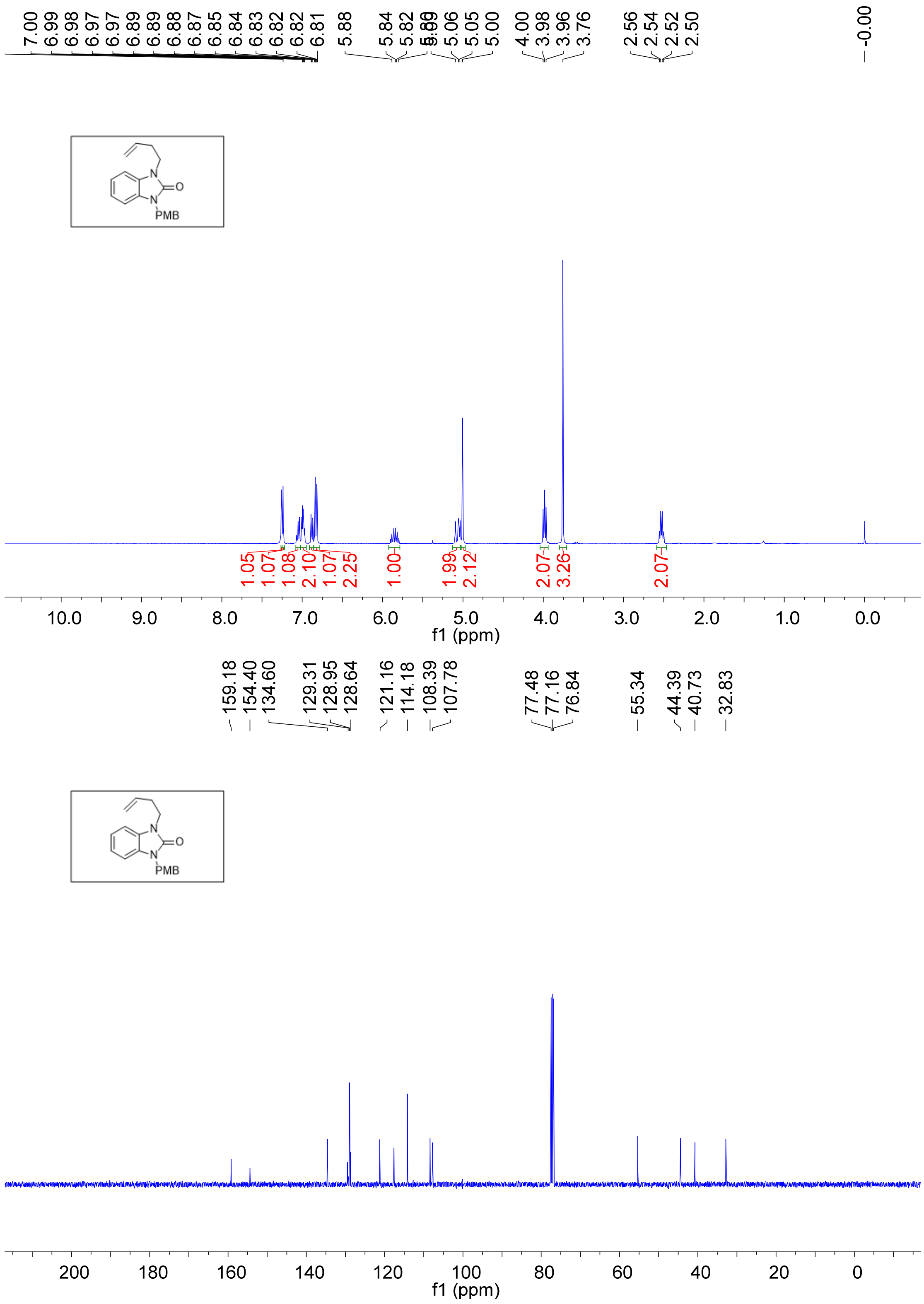

Supplementary Figure 39. ^{1}H and ^{13}C NMR spectra of compound **1u** in $CDCl_3$

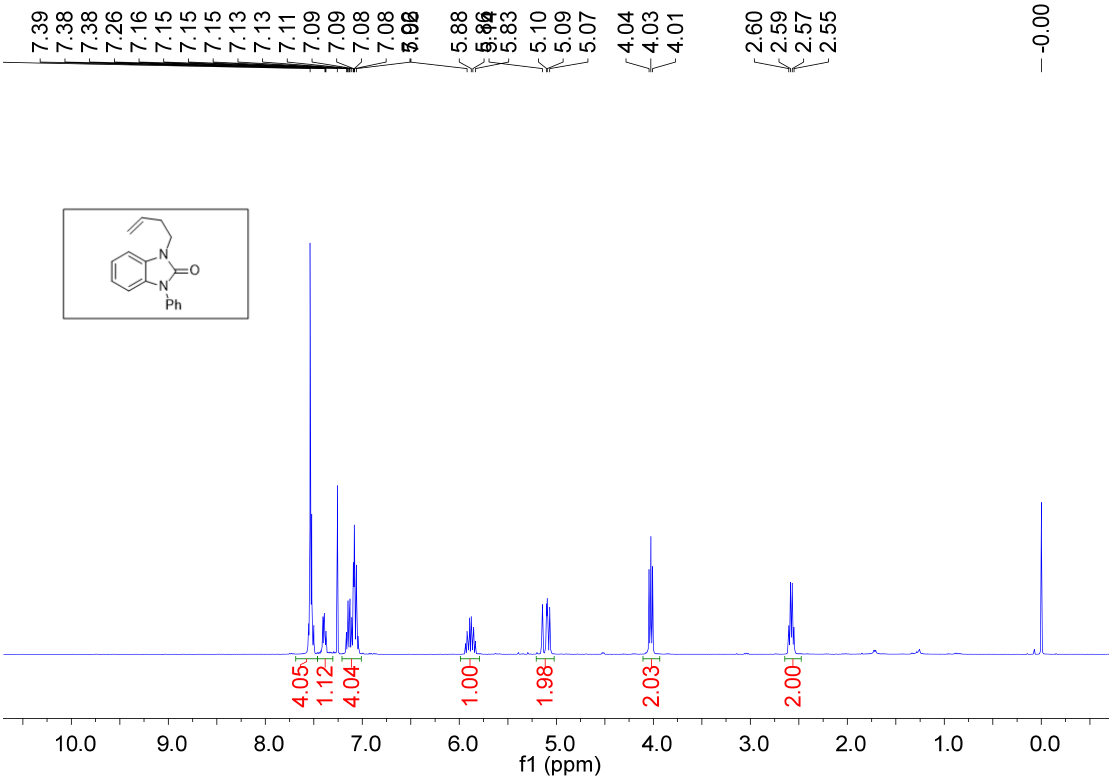

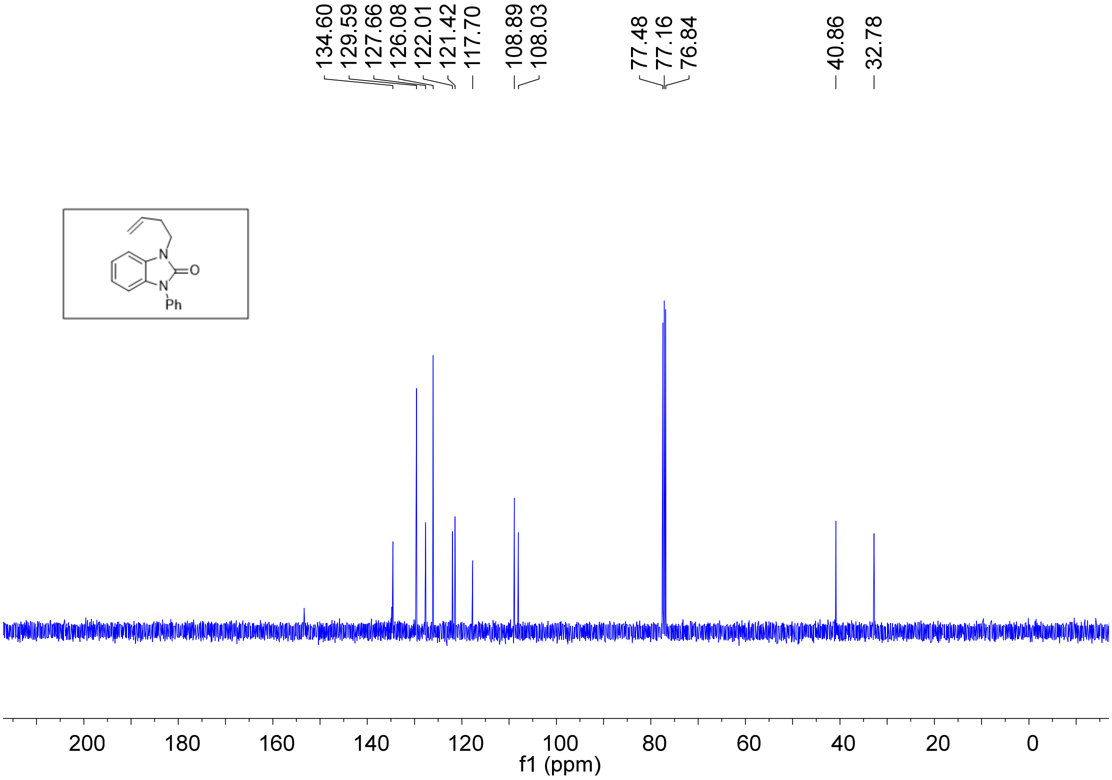

Supplementary Figure 40. ^{1}H and ^{13}C NMR spectra of compound **1v** in $CDCl_3$

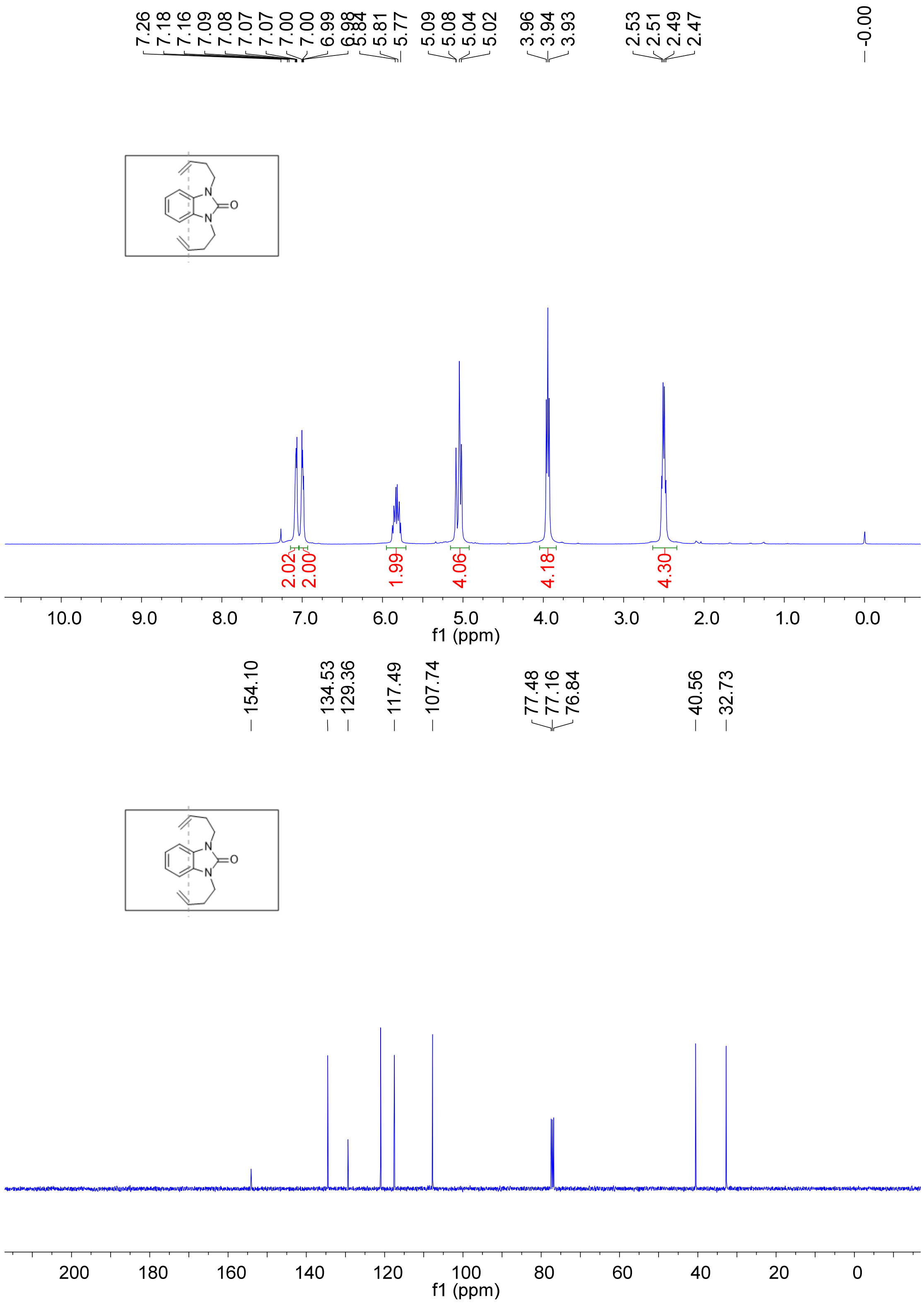

Supplementary Figure 41. ^{1}H and ^{13}C NMR spectra of compound **1w** in $CDCl_3$

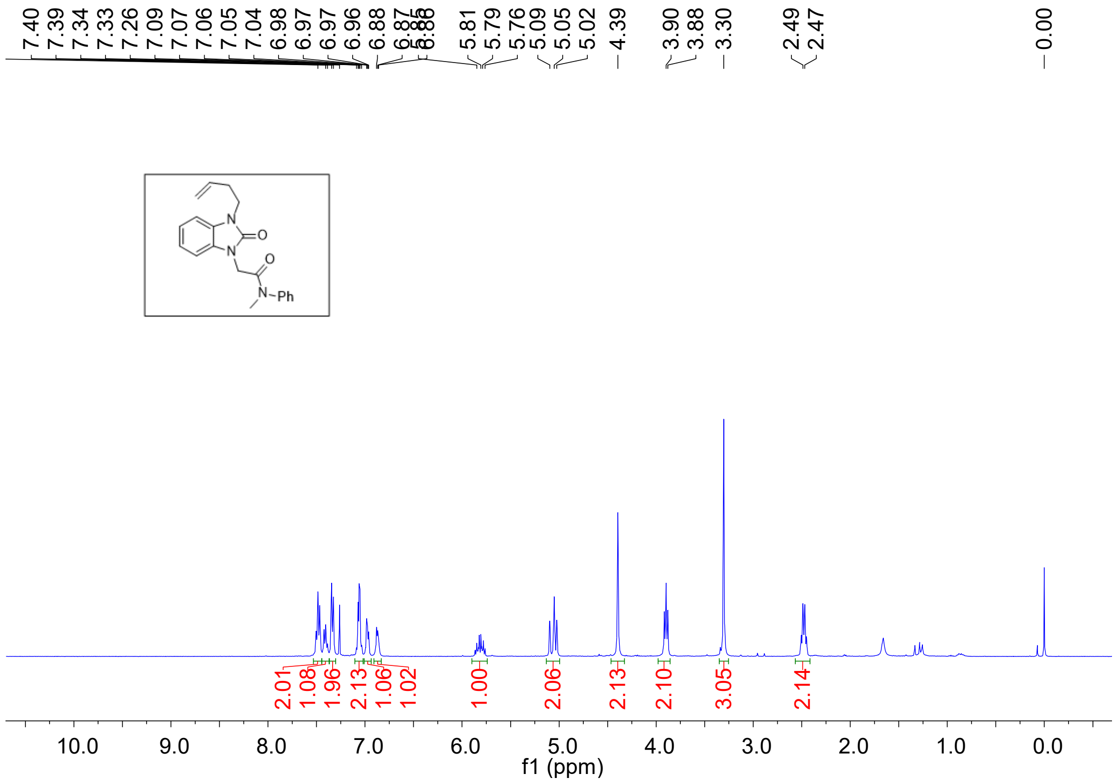

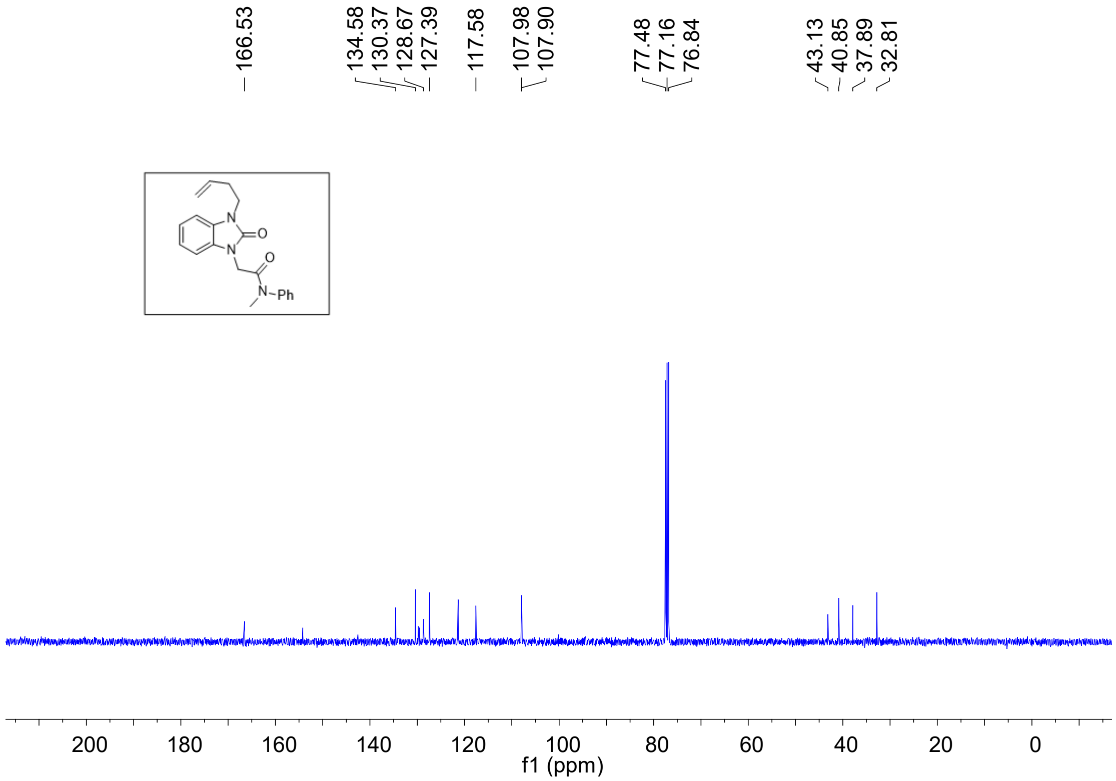

Supplementary Figure 42. ^{1}H and ^{13}C NMR spectra of compound **1x** in $CDCl_3$

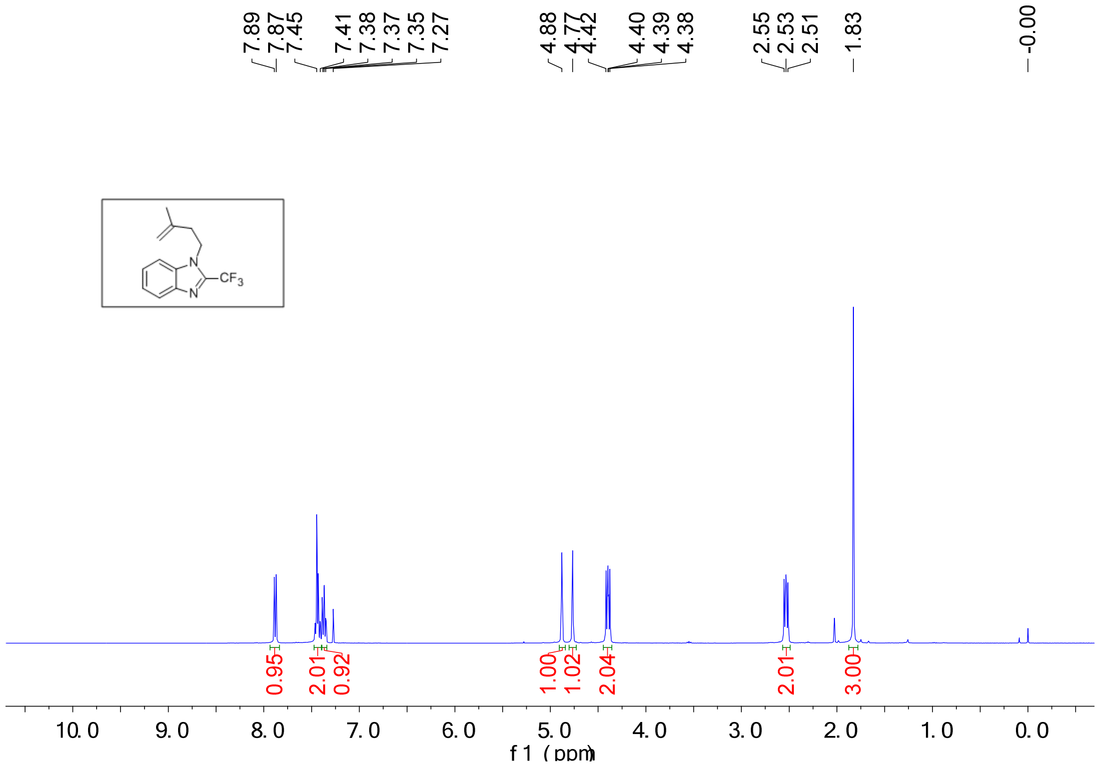

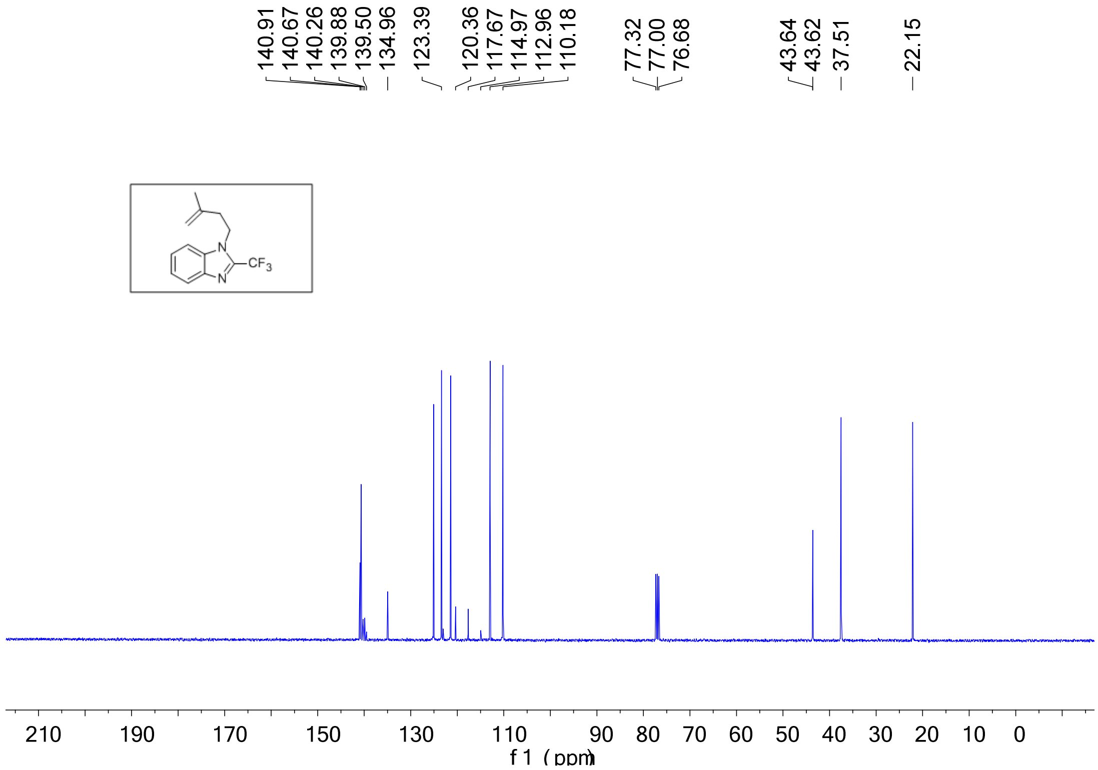

Supplementary Figure 43. ^{1}H and ^{13}C NMR spectra of compound **1a'** in $CDCl_3$.

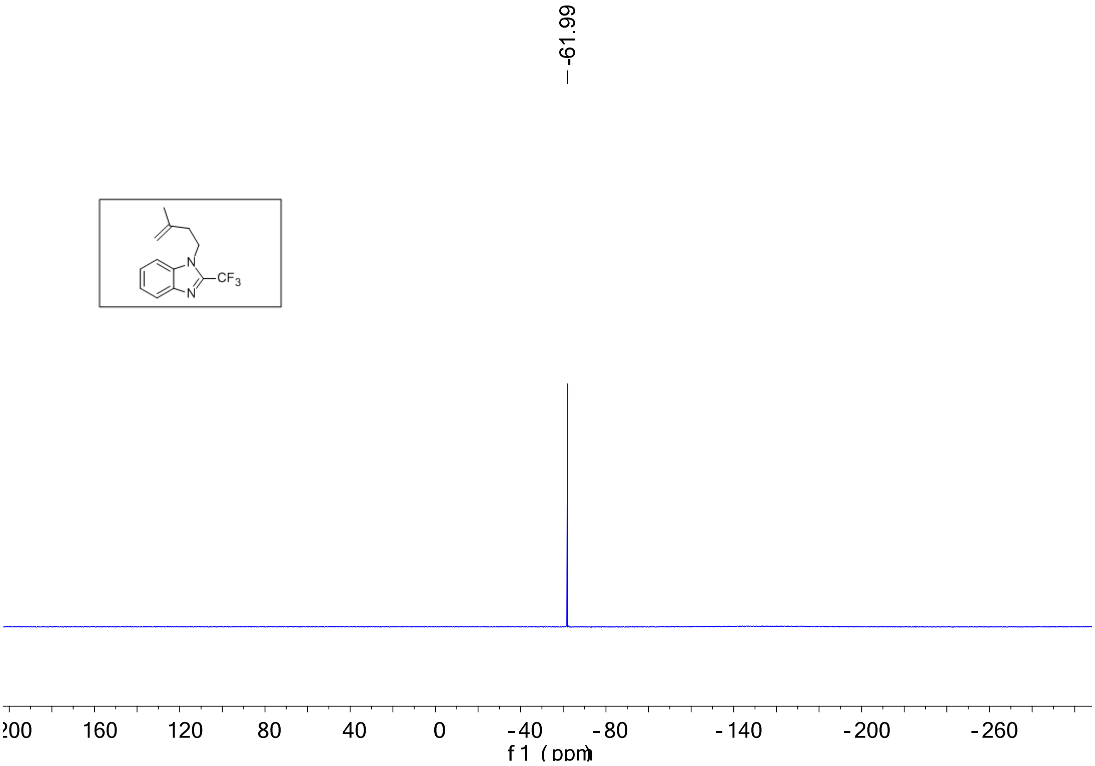

Supplementary Figure 44. ^{19}F NMR spectra of compound **1a'** in $CDCl_3$.

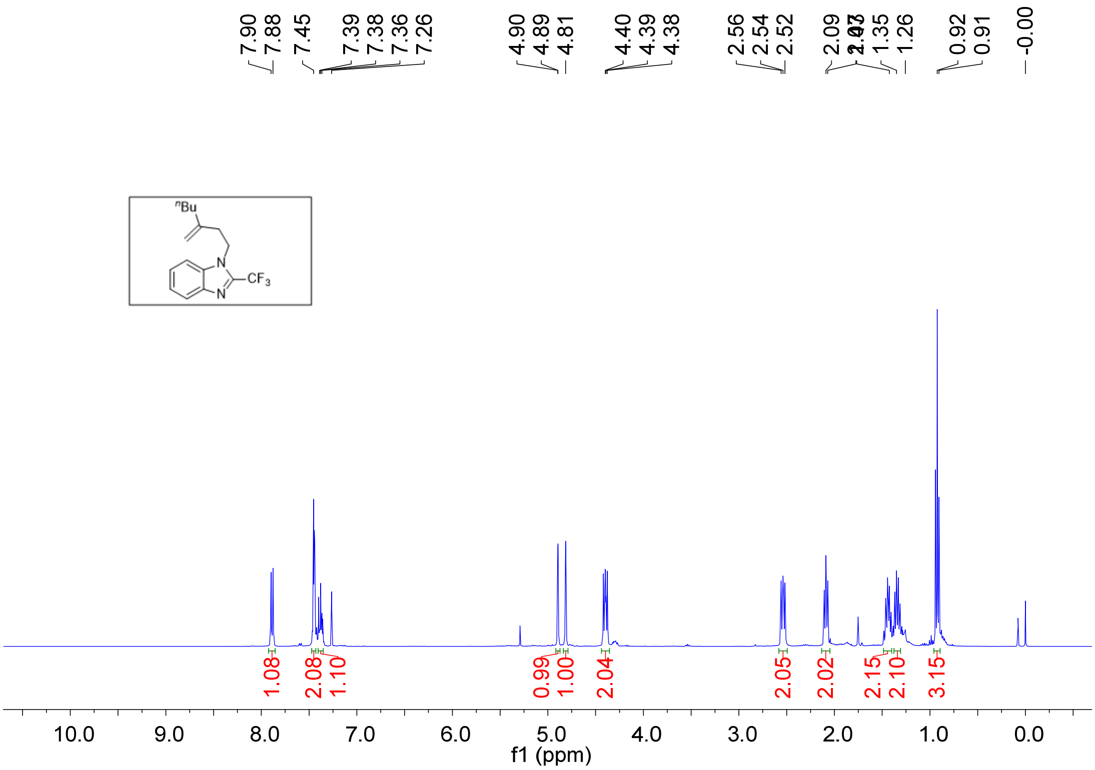

Supplementary Figure 45. ^{1}H NMR spectrum of compound **1b'** in $CDCl_3$.

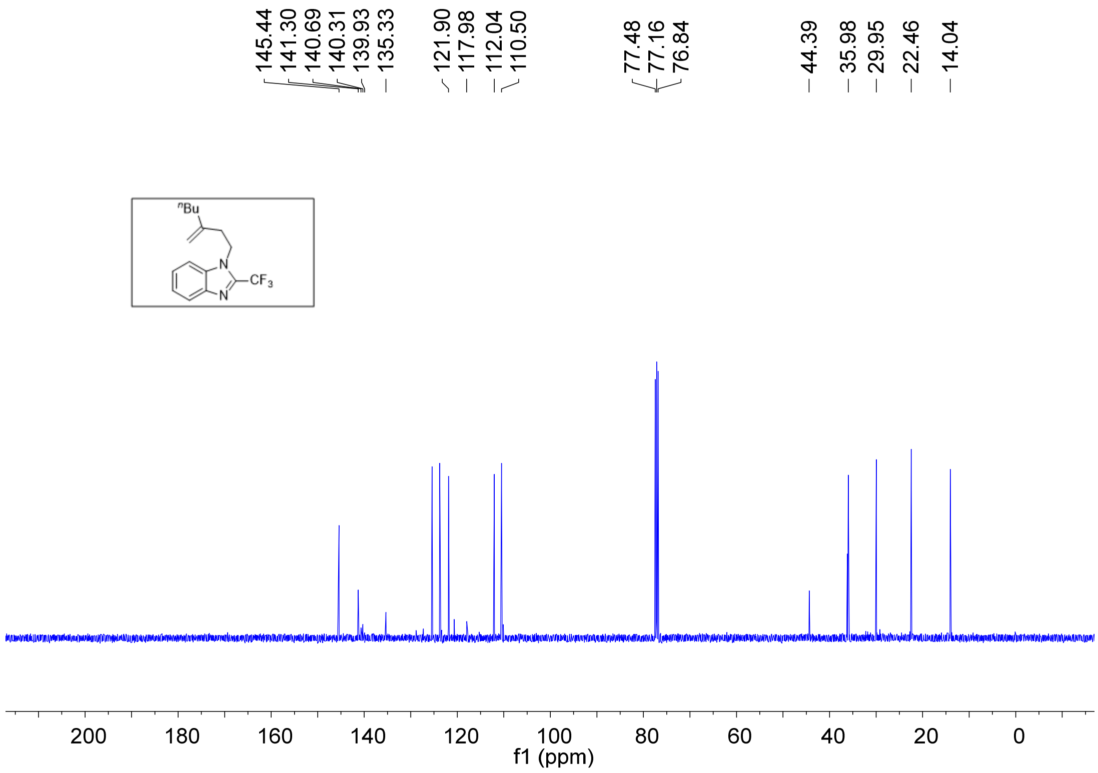

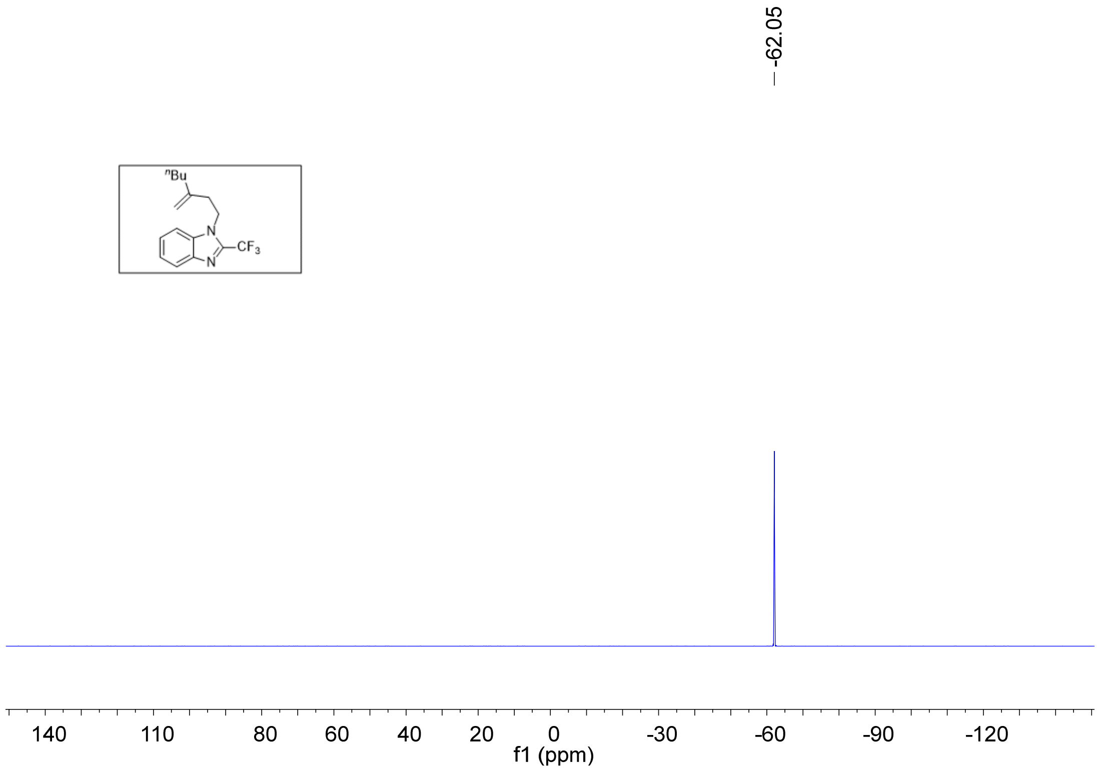

Supplementary Figure 46. ^{13}C and ^{19}F NMR spectra of compound **1b’** in $CDCl_3$.

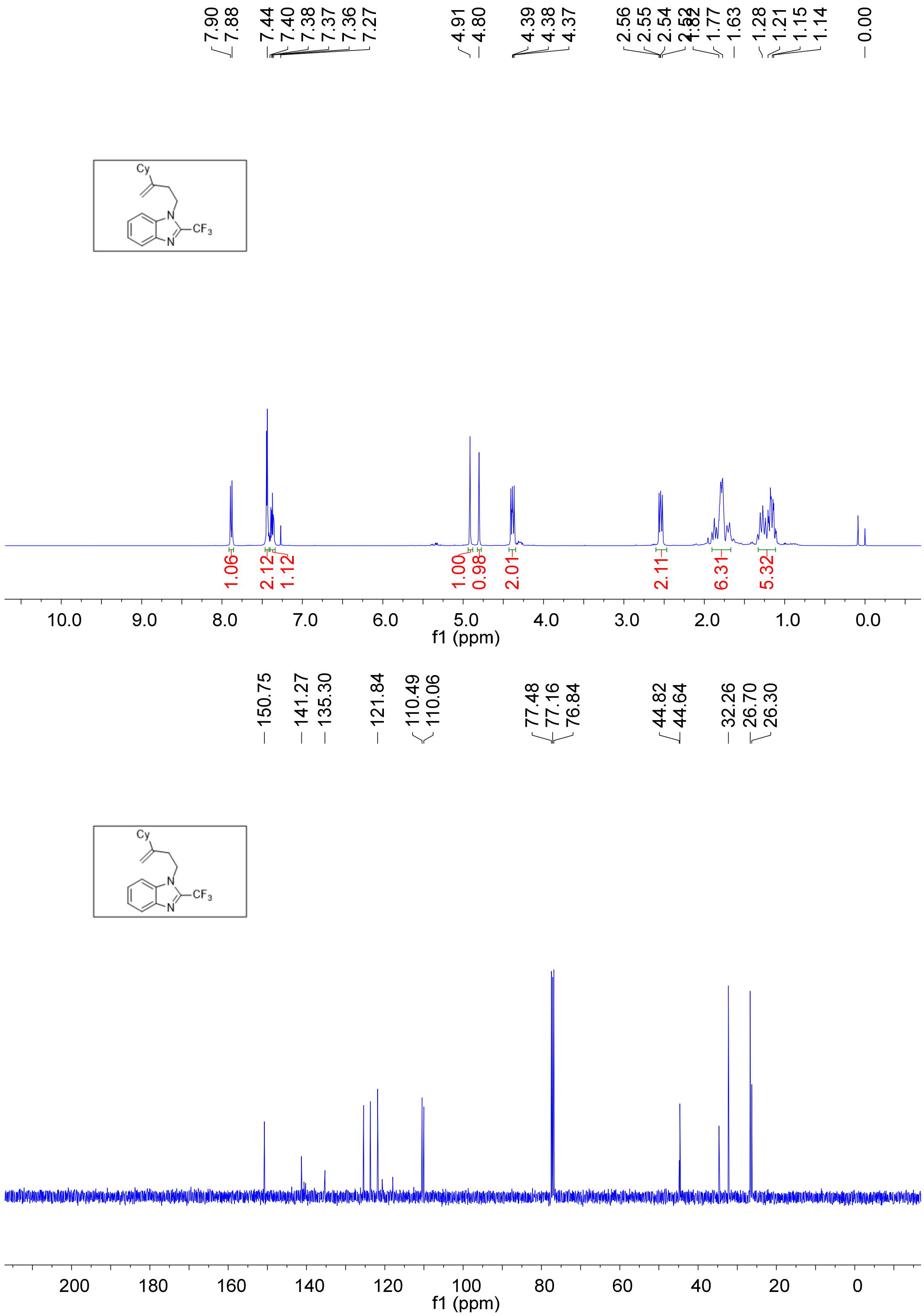

Supplementary Figure 47. ^{1}H and ^{13}C NMR spectra of compound **1c'** in $CDCl_3$.

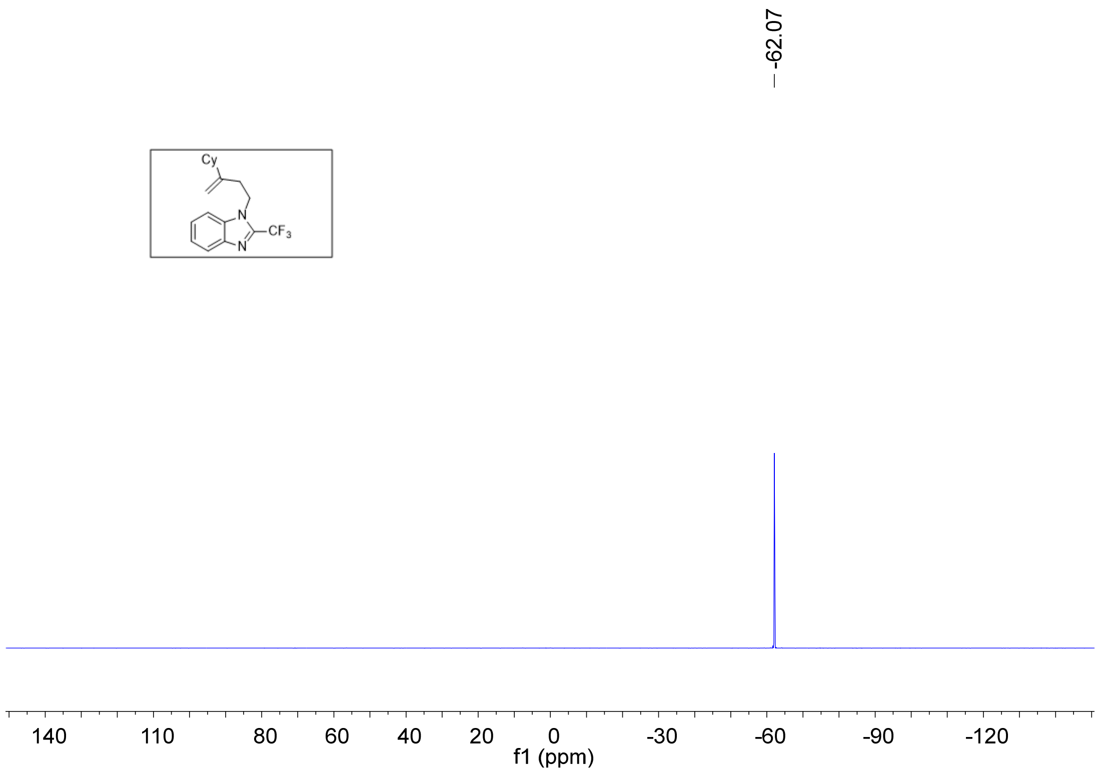

Supplementary Figure 48. ^{19}F NMR spectrum of compound **1c'** in $CDCl_3$.

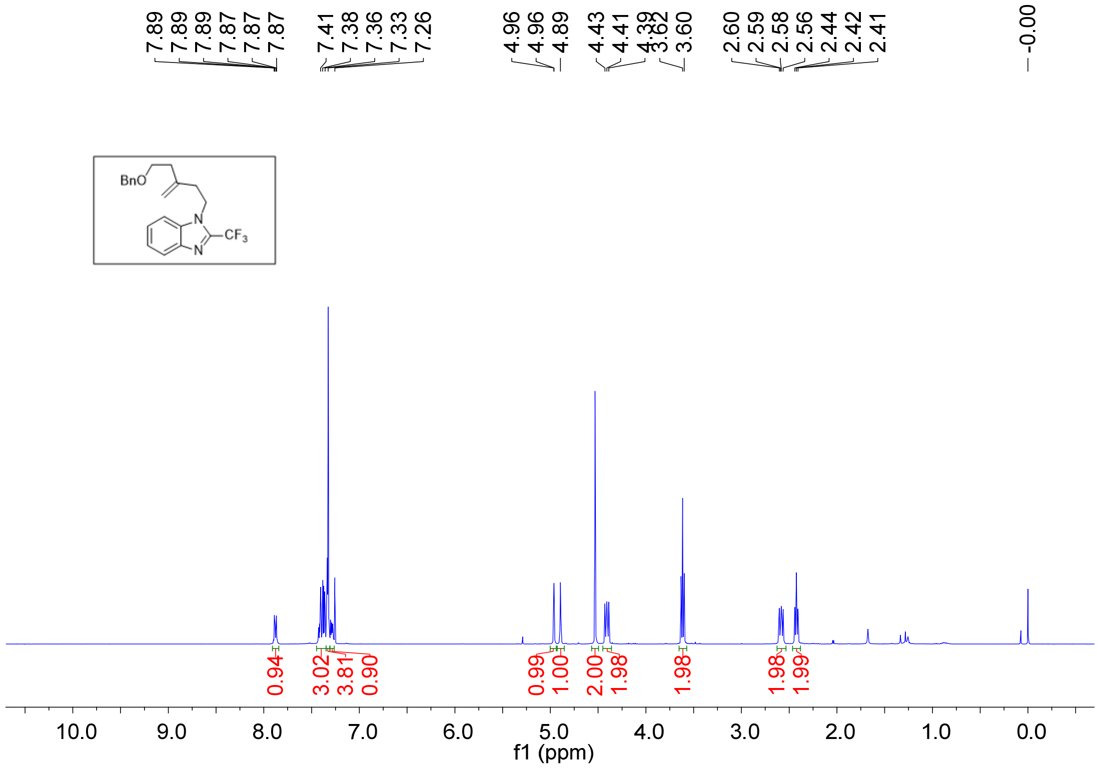

Supplementary Figure 49. ^{1}H NMR spectrum of compound **1d'** in $CDCl_3$.

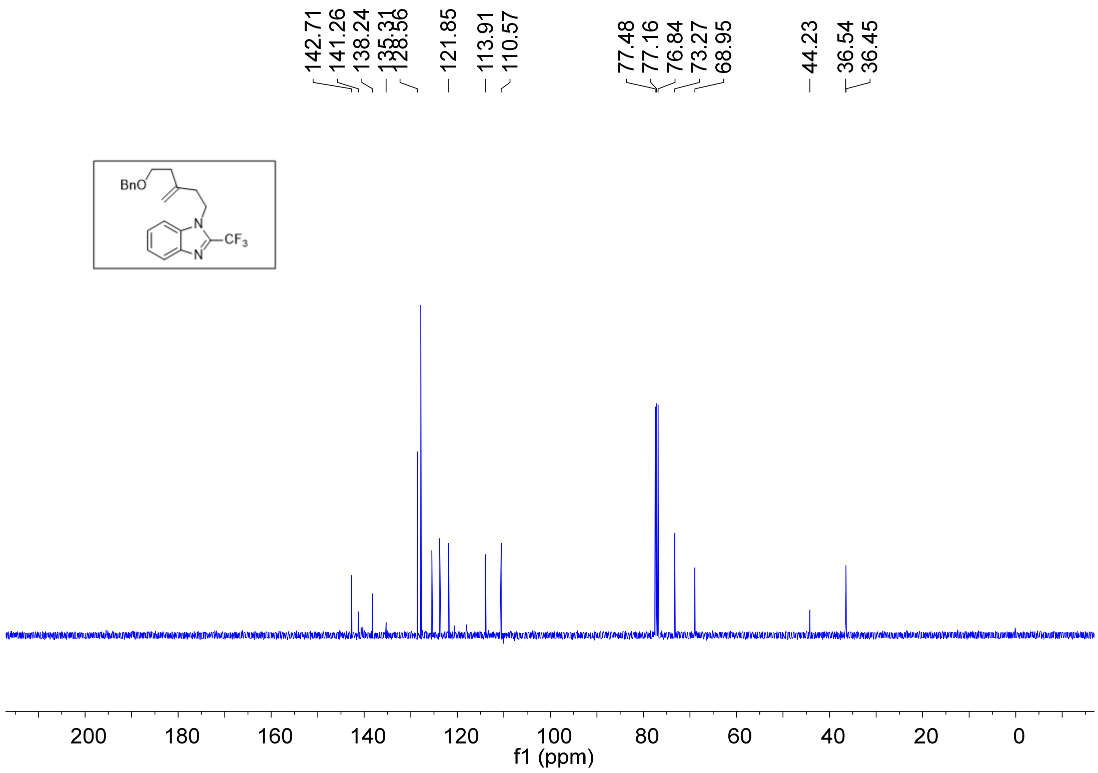

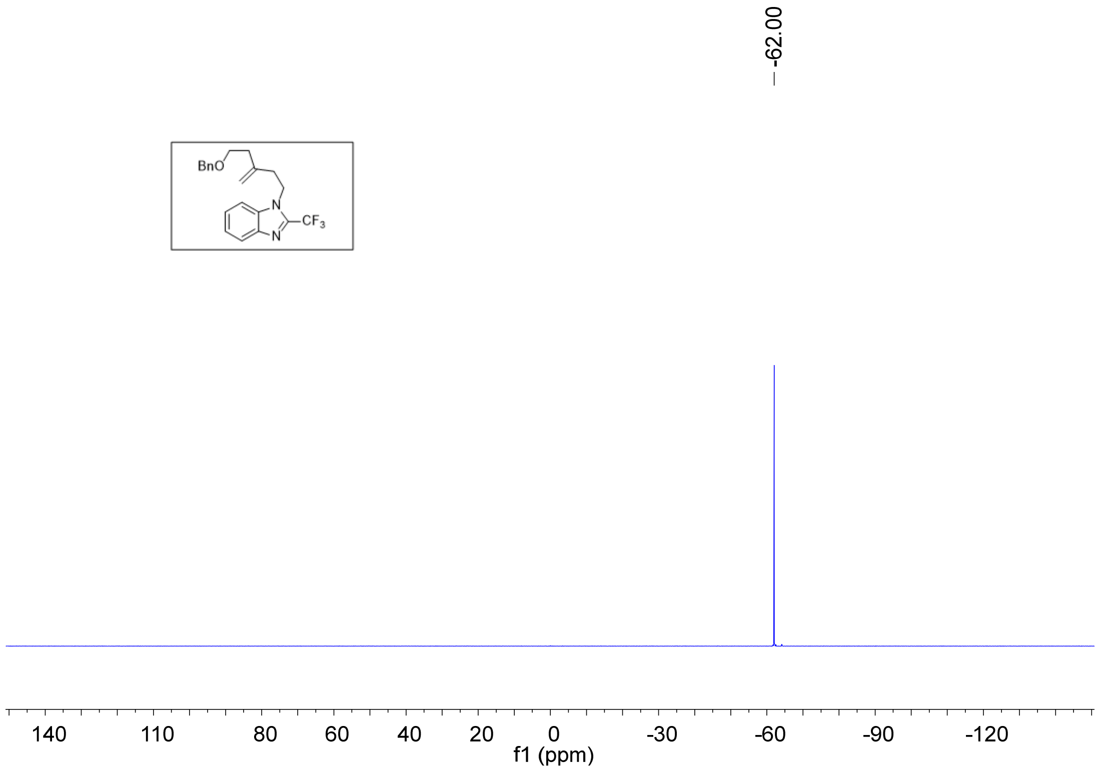

Supplementary Figure 50. ^{13}C and ^{19}F NMR spectra of compound **1d'** in $CDCl_3$.

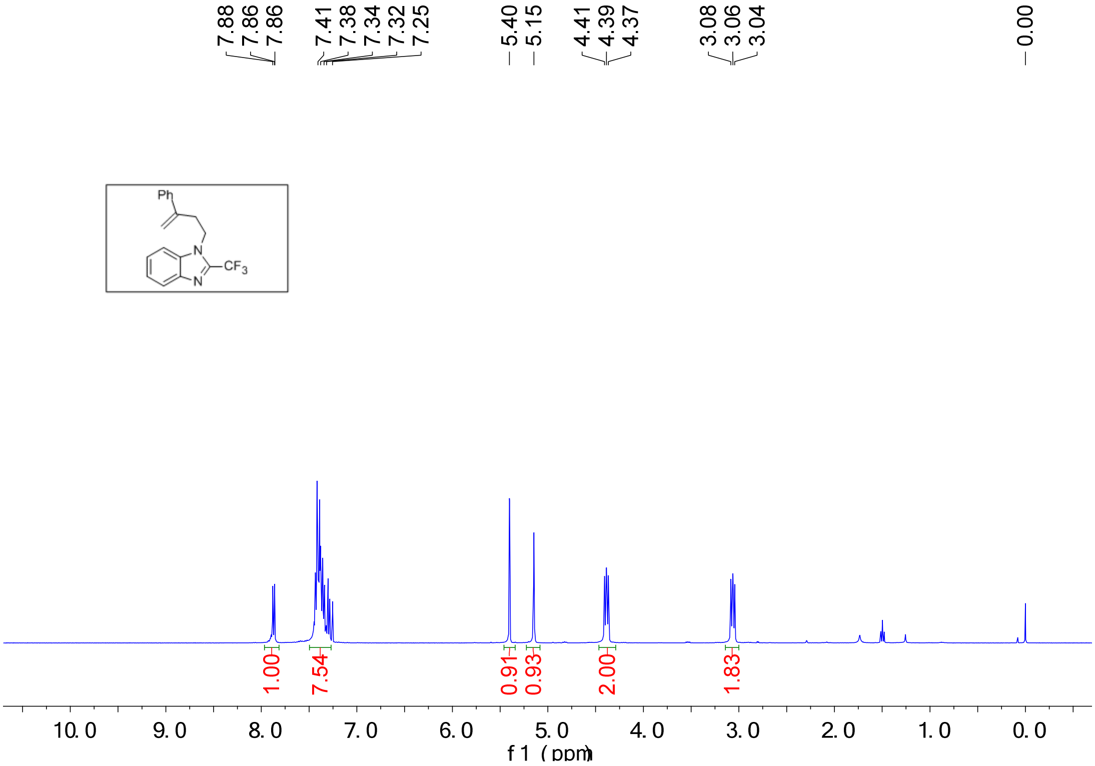

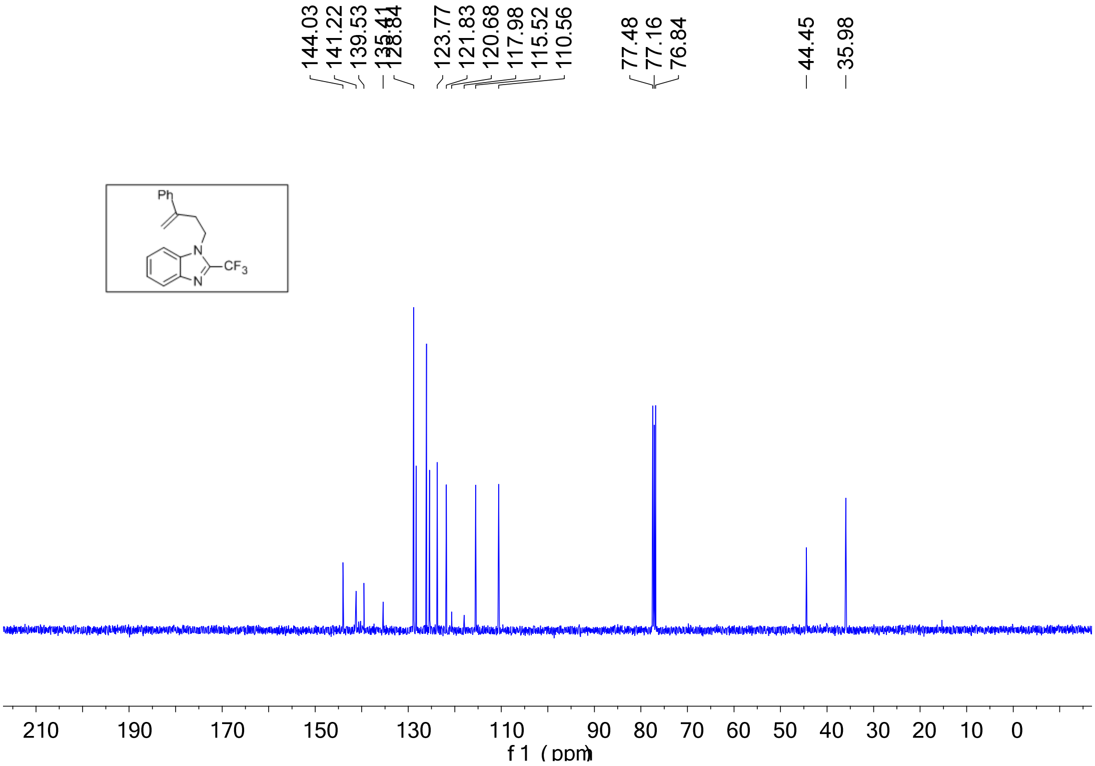

Supplementary Figure 51. ^{1}H and ^{13}C NMR spectra of compound **1e'** in $CDCl_3$.

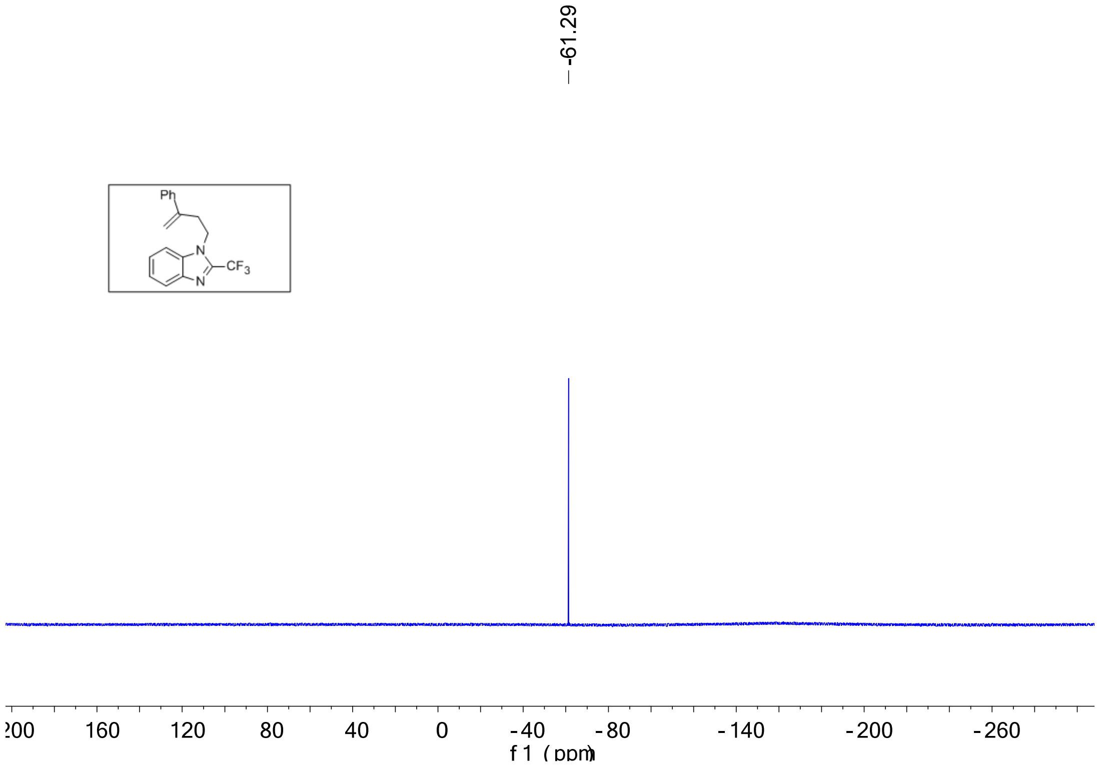

Supplementary Figure 52. ^{19}F NMR spectrum of compound **1e'** in $CDCl_3$.

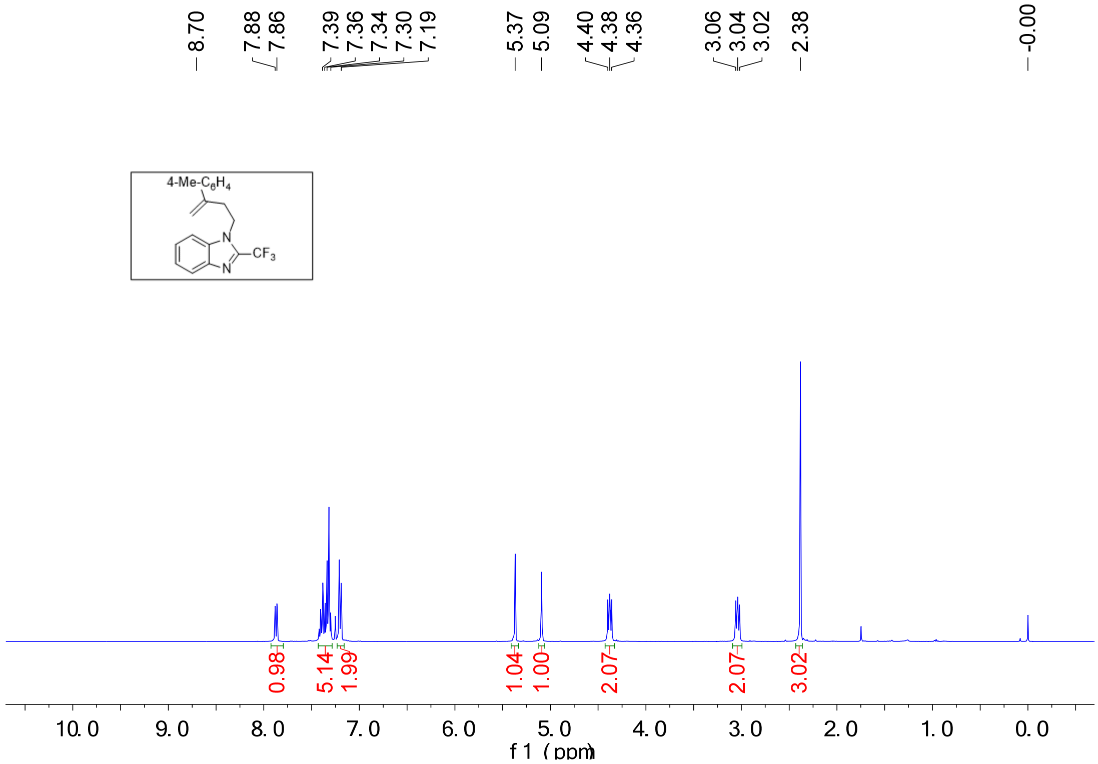

Supplementary Figure 53. ^{1}H NMR spectrum of compound **1f'** in $CDCl_3$.

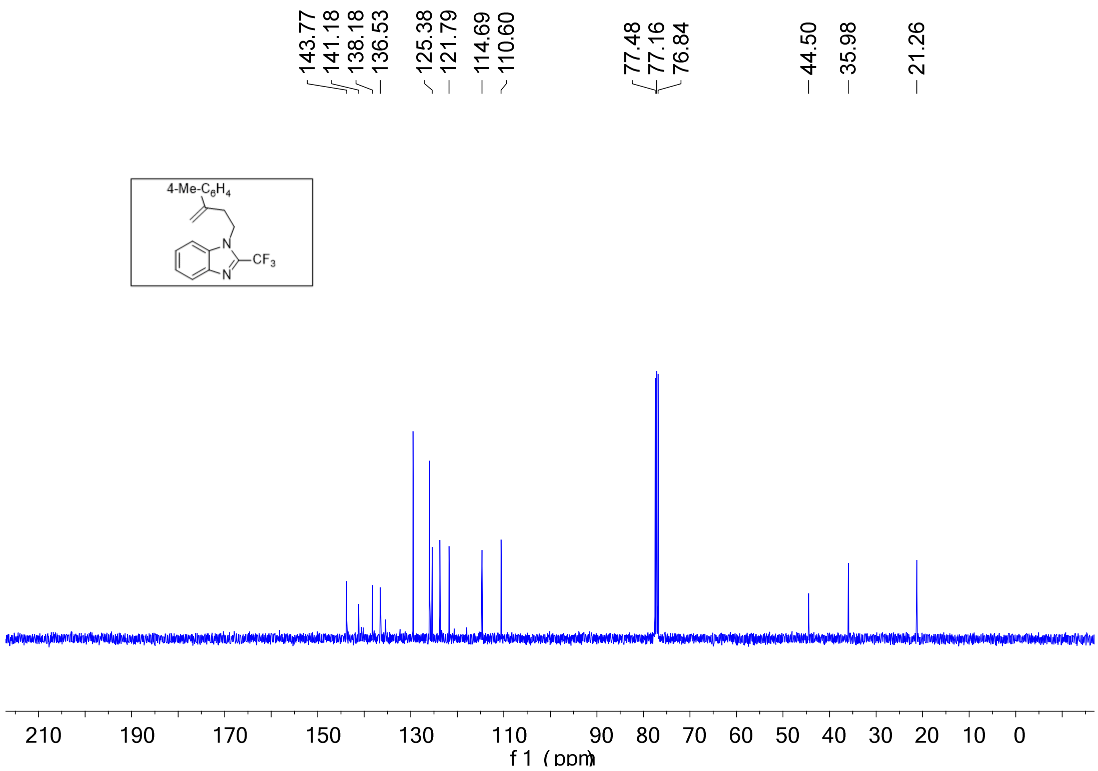

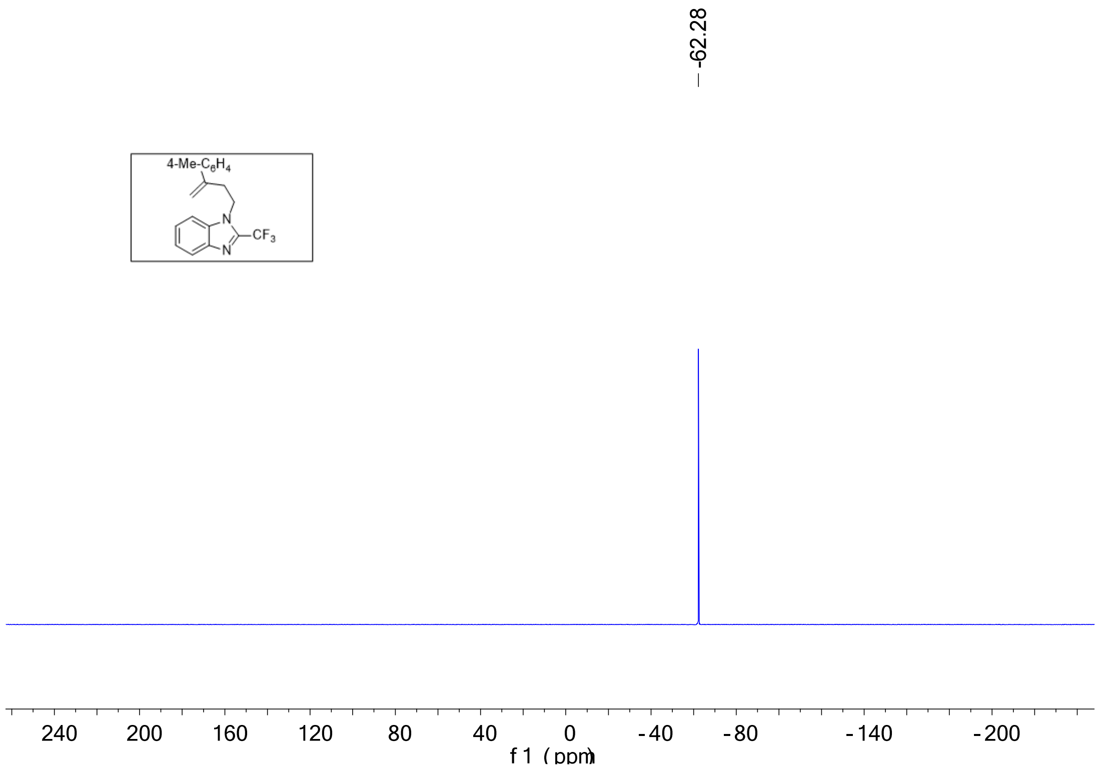

Supplementary Figure 54. ^{13}C and ^{19}F NMR spectra of compound **1f'** in $CDCl_3$.

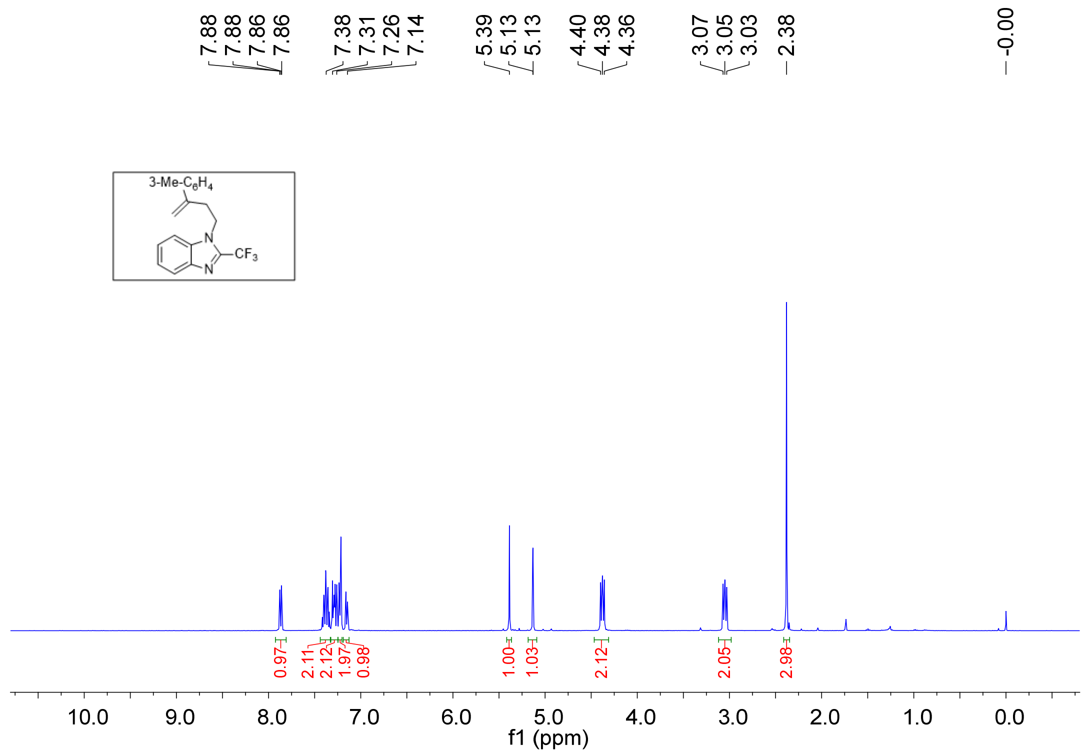

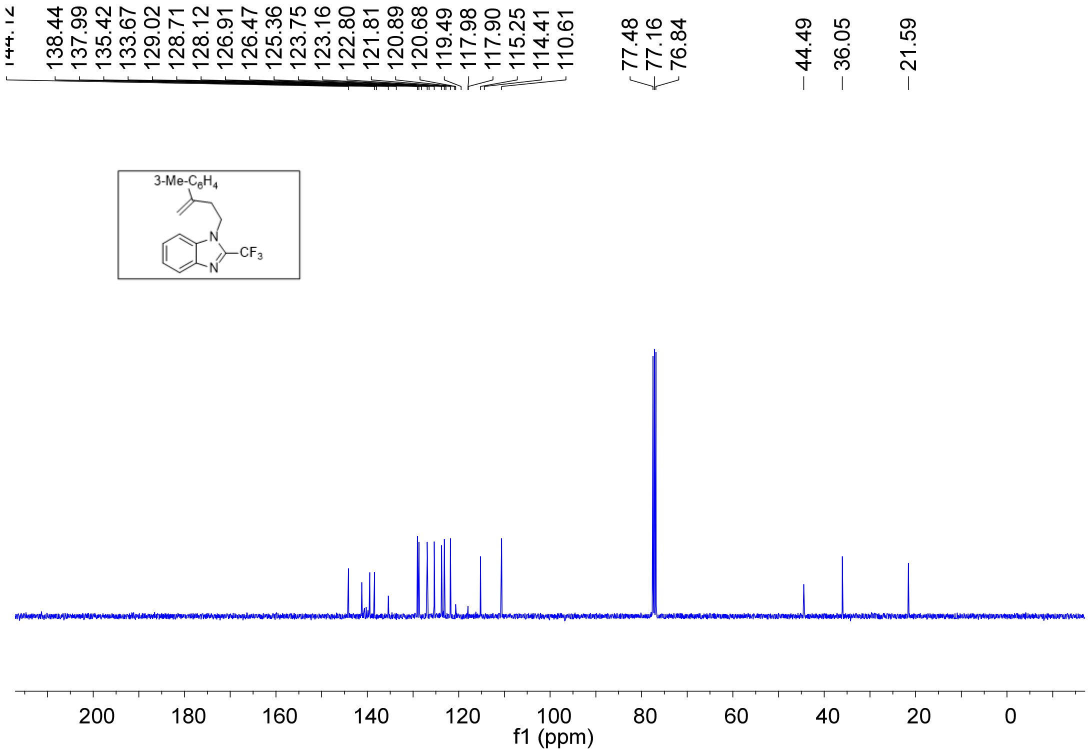

Supplementary Figure 55. ^{1}H and ^{13}C NMR spectra of compound **1g'** in $CDCl_3$.

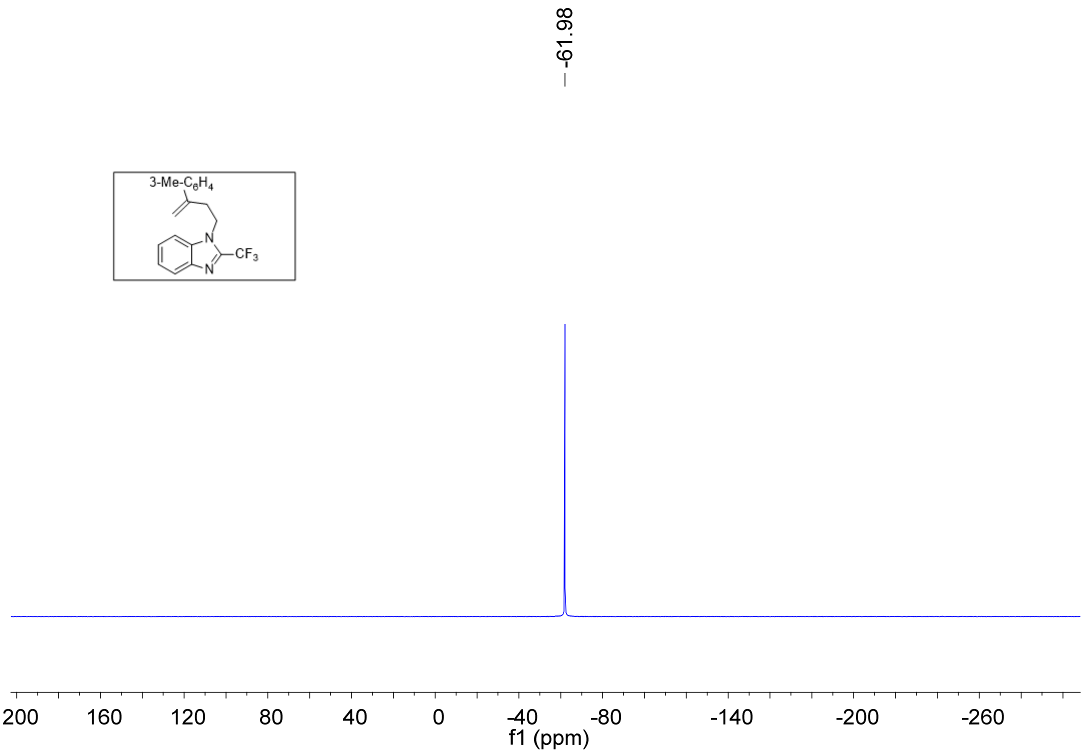

Supplementary Figure 56.^{19}F NMR spectrum of compound **1g'** in $CDCl_3$.

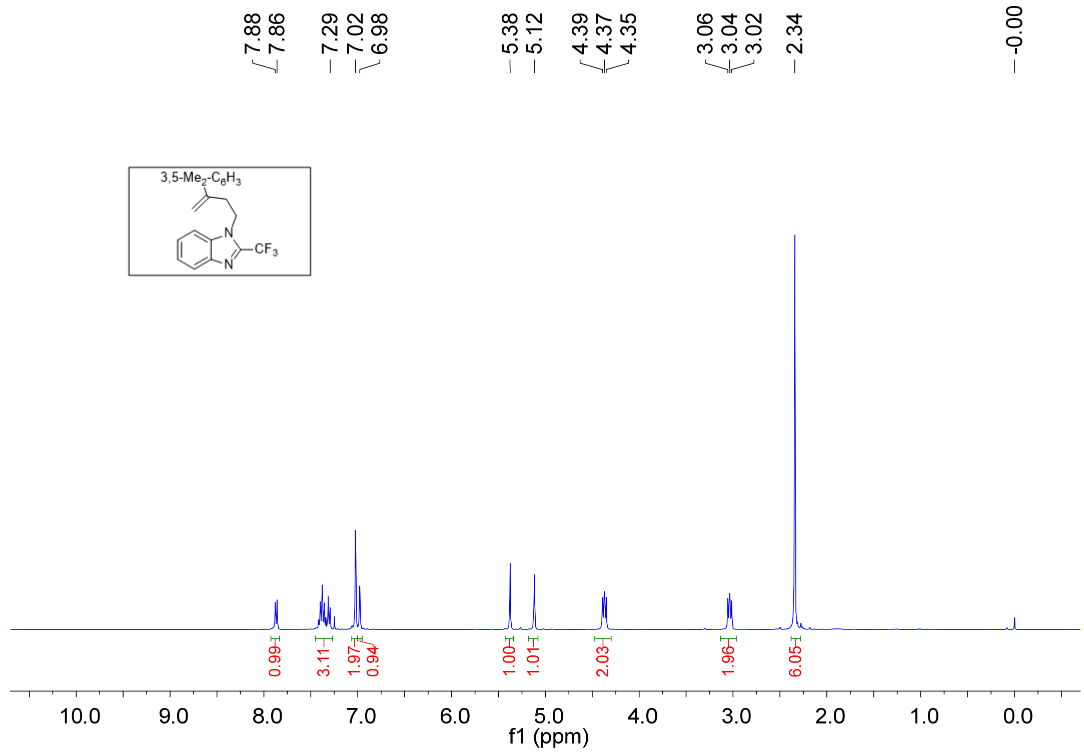

Supplementary Figure 57. ^{1}H NMR spectrum of compound **1h'** in $CDCl_3$

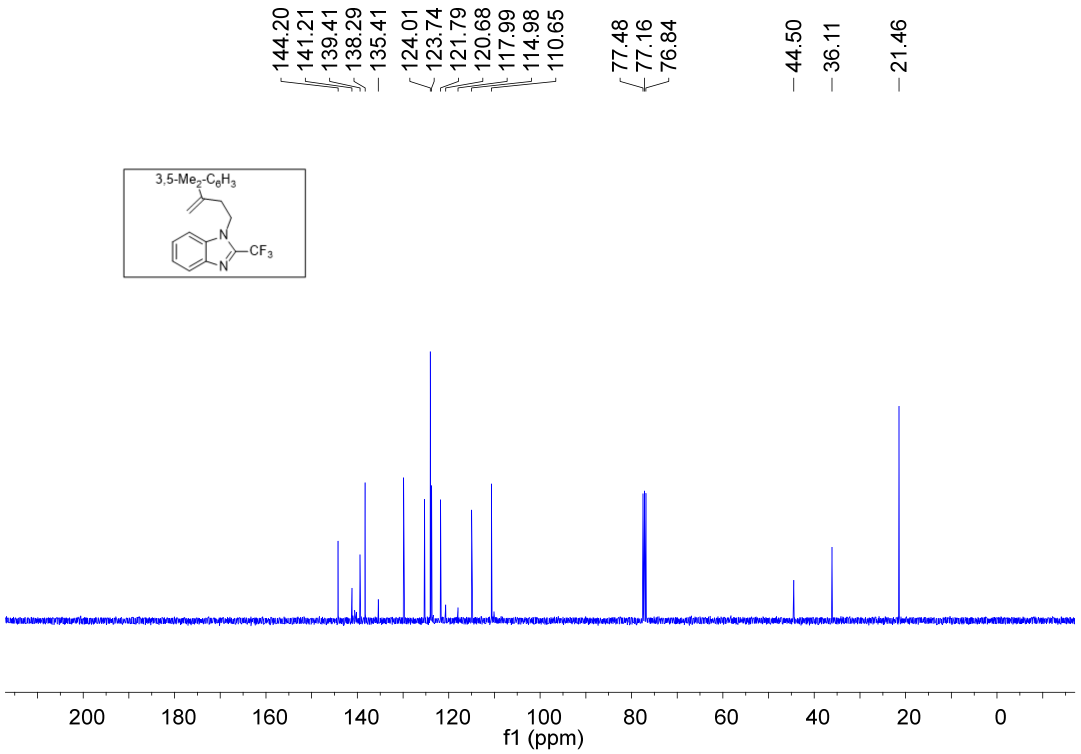

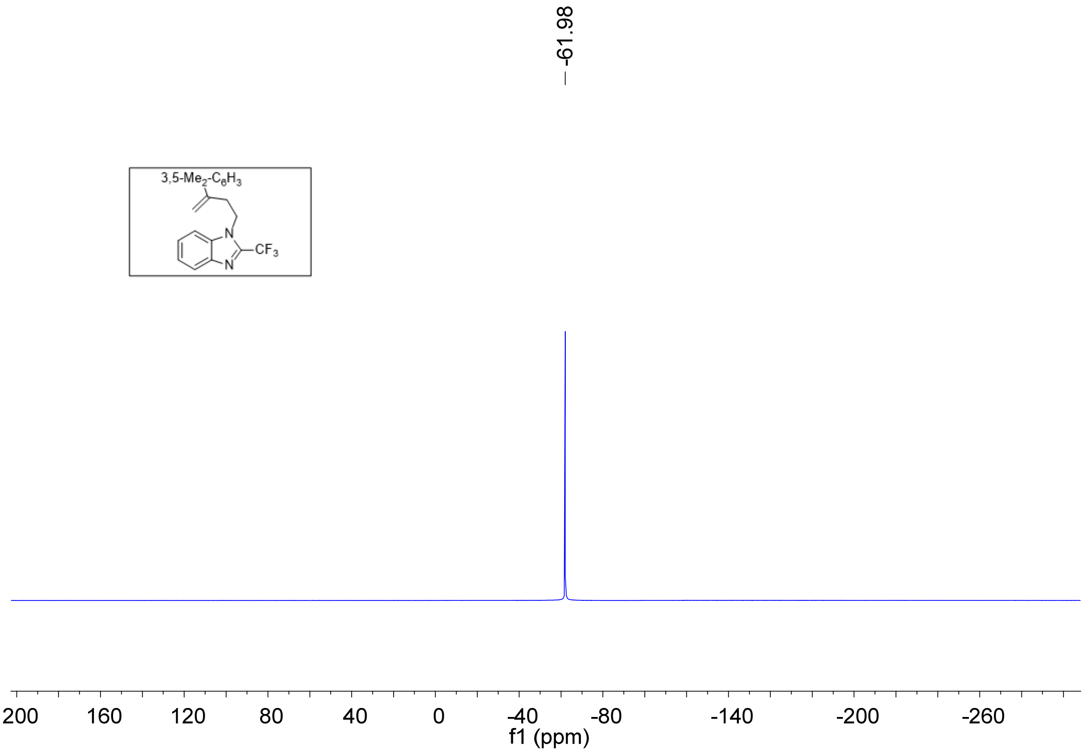

Supplementary Figure 58. ^{13}C and ^{19}F NMR spectrum of compound **1h'** in $CDCl_3$

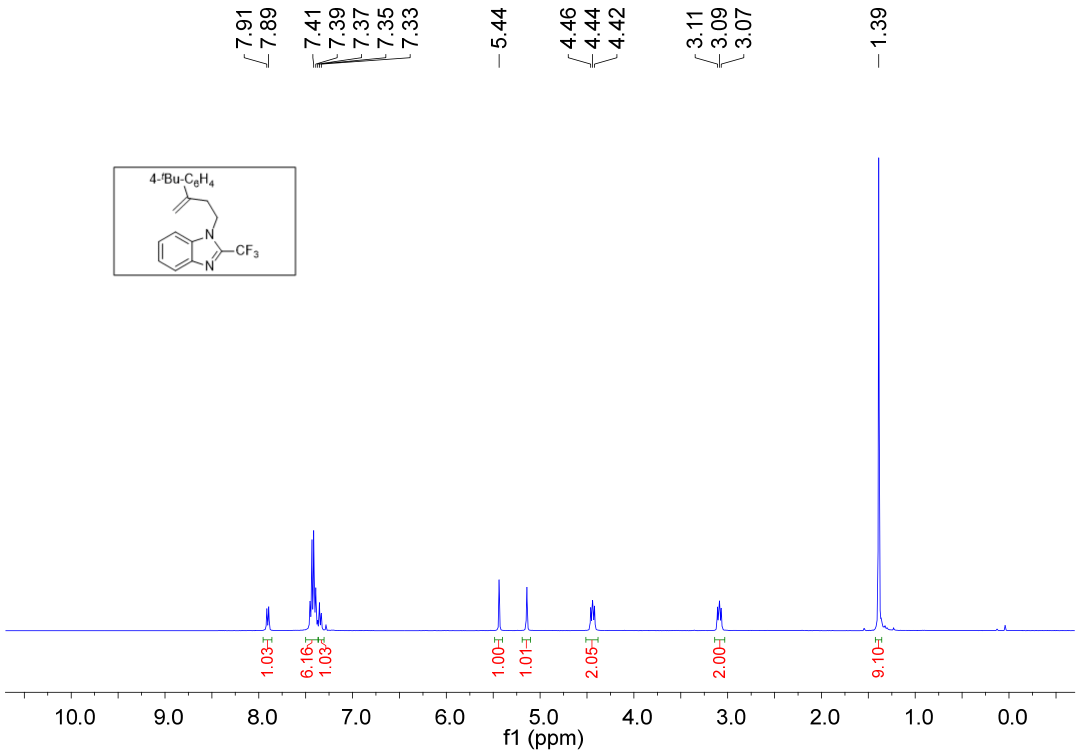

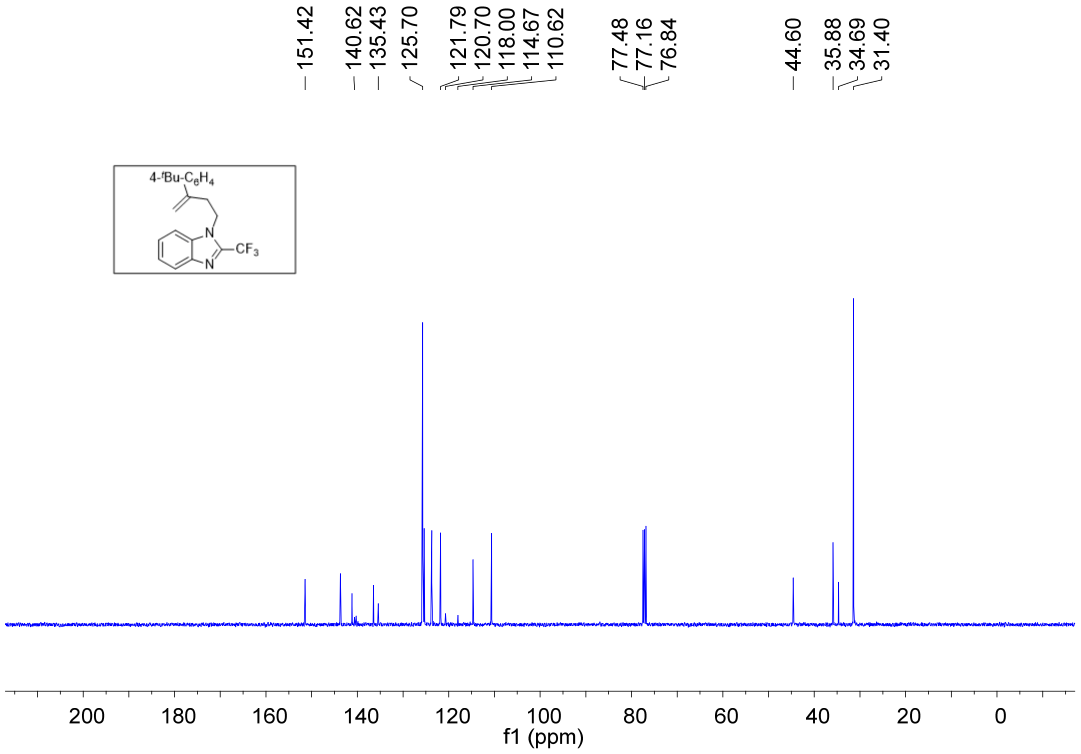

Supplementary Figure 59. ^{1}H and ^{13}C NMR spectra of compound **1i'** in $CDCl_3$.

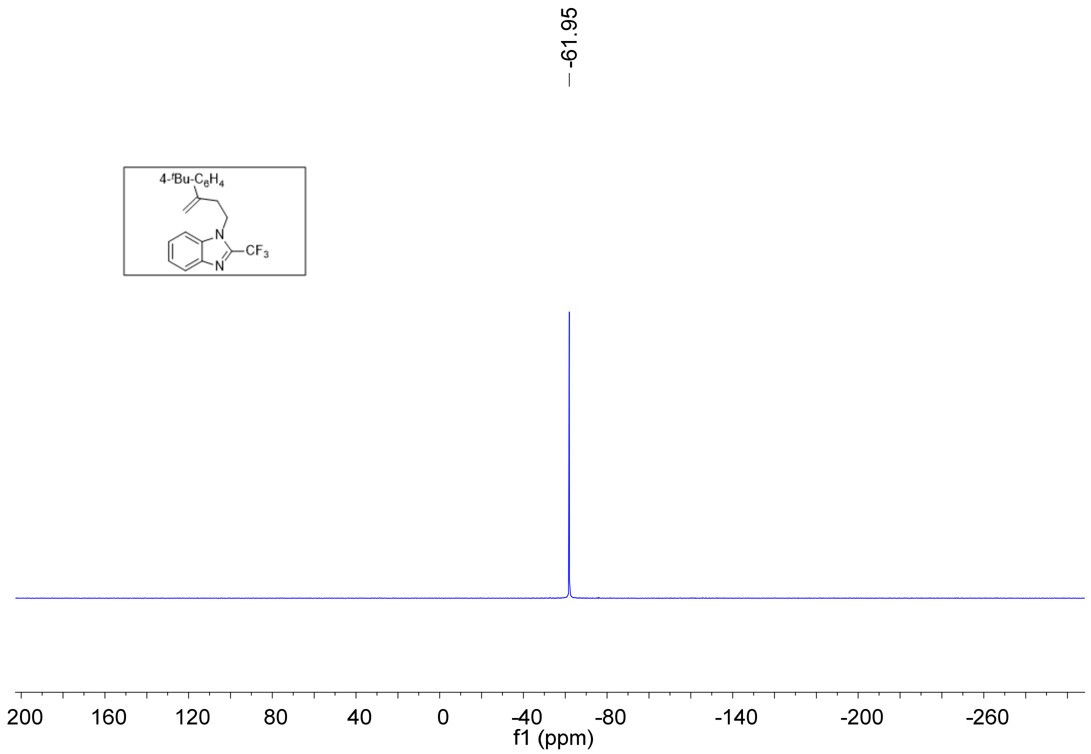

Supplementary Figure 60. ^{19}F NMR spectrum of compound **1i'** in $CDCl_3$.

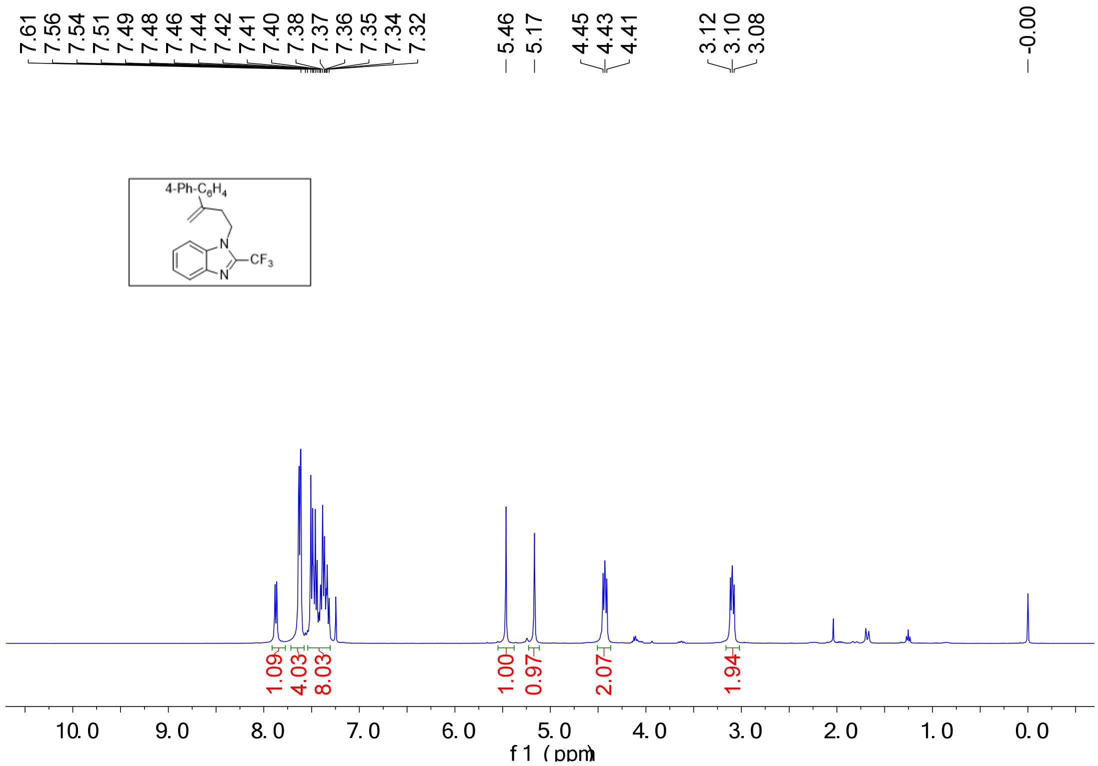

Supplementary Figure 61. ^{1}H NMR spectrum of compound **1j'** in $CDCl_3$.

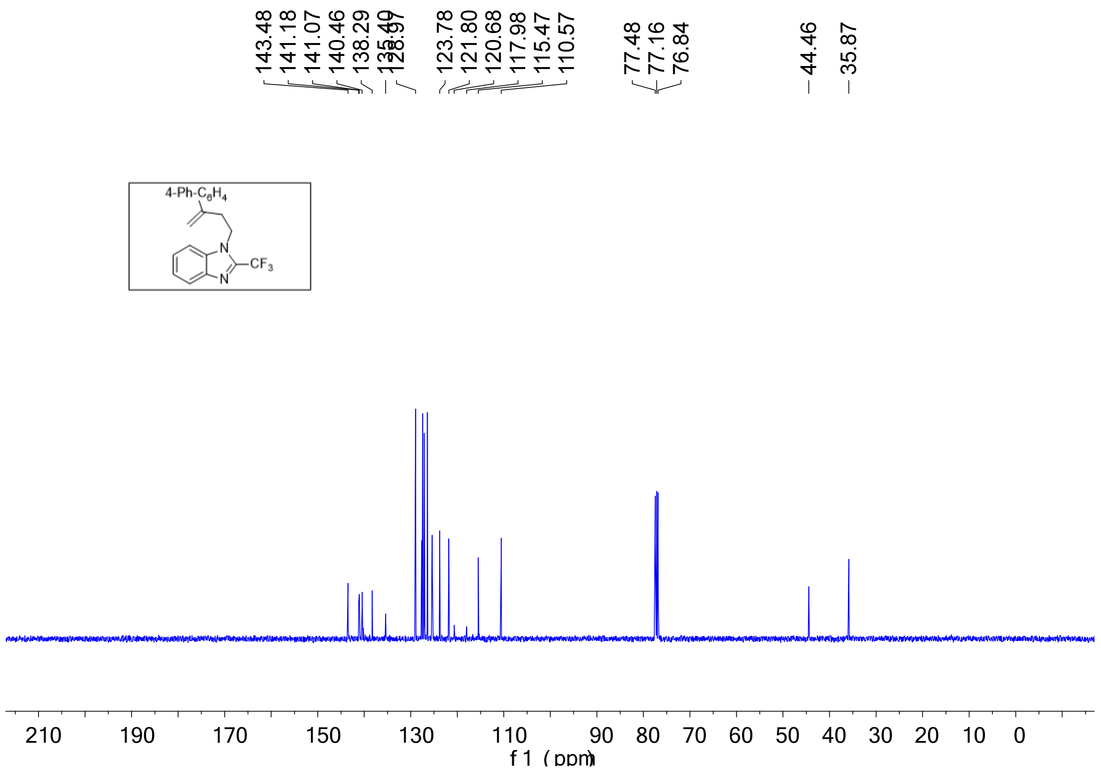

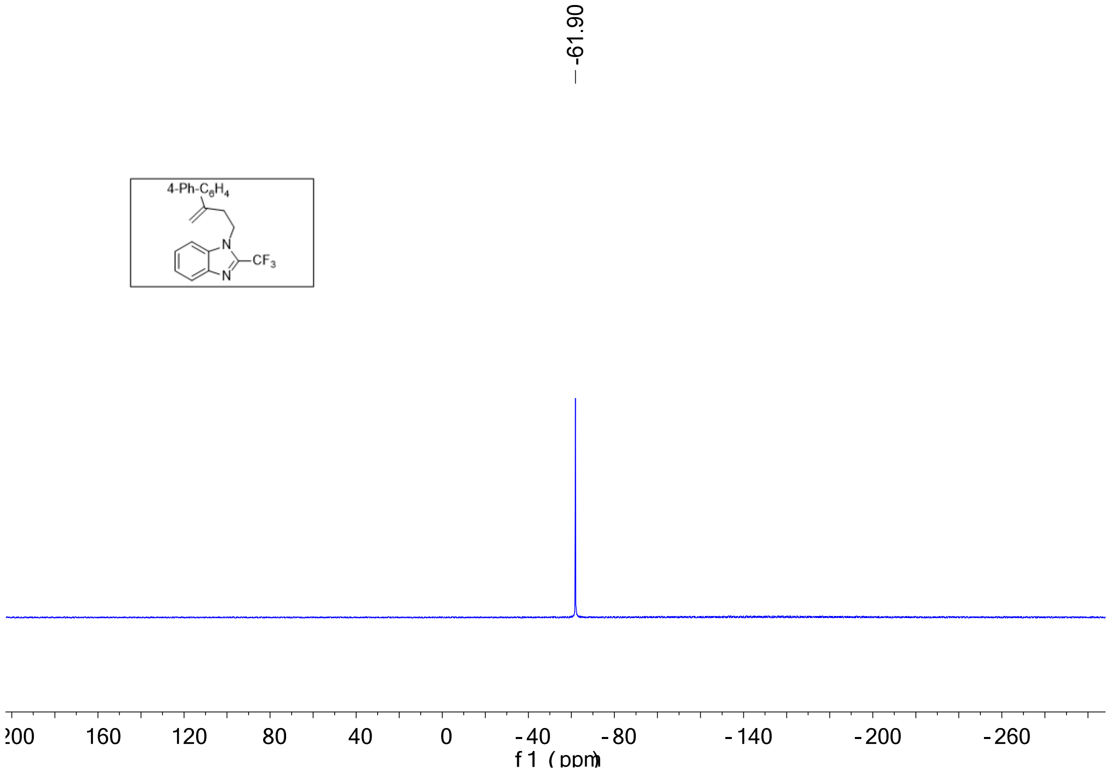

Supplementary Figure 62. ^{13}C and ^{19}F NMR spectra of compound **1j'** in $CDCl_3$.

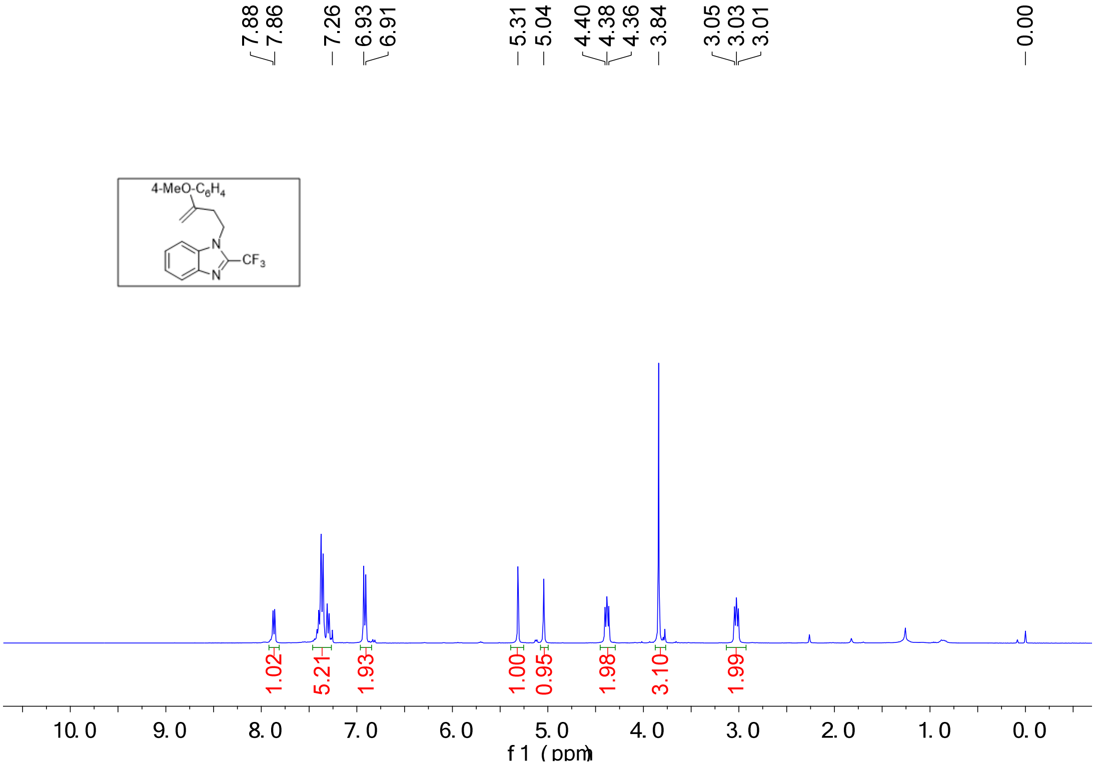

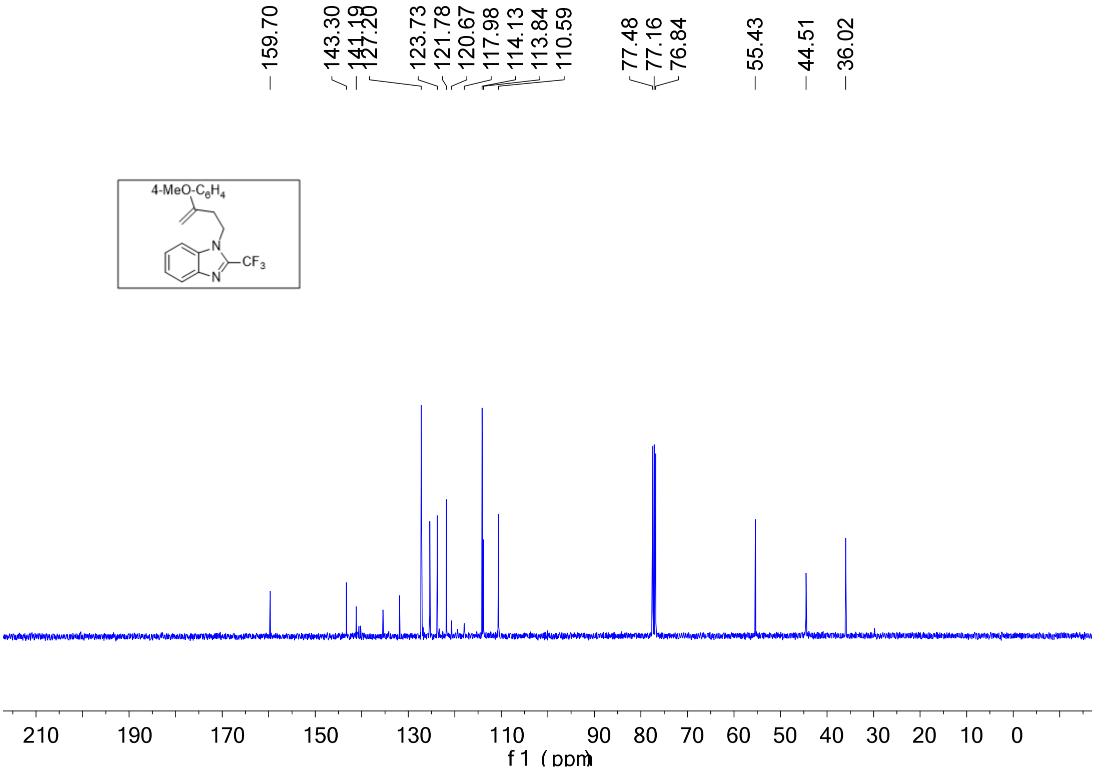

Supplementary Figure 63. ^{1}H and ^{13}C NMR spectra of compound **1k′** in $CDCl_3$.

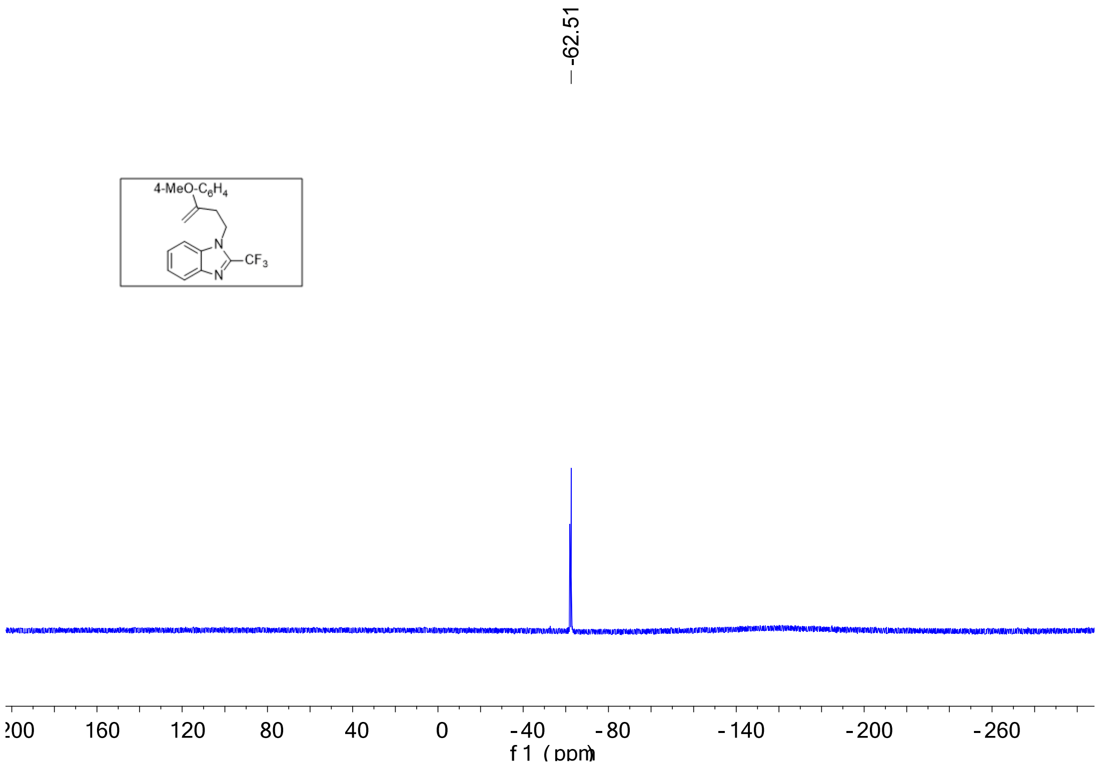

Supplementary Figure 64. ^{19}F NMR spectrum of compound **1k'** in $CDCl_3$.

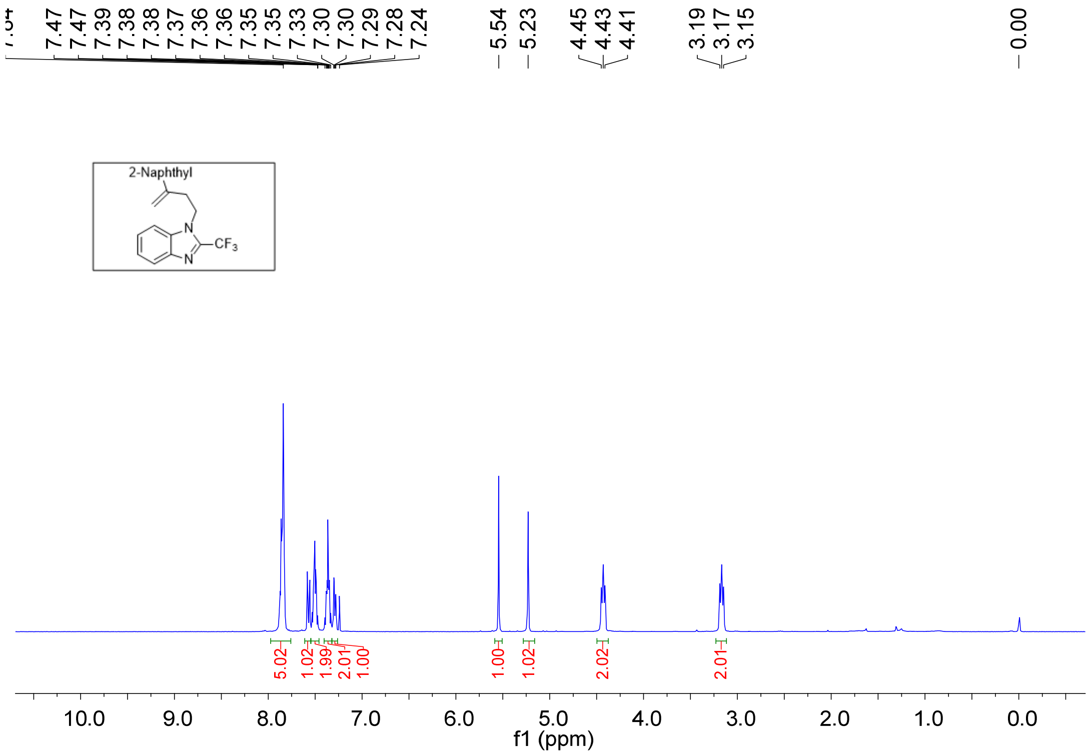

Supplementary Figure 65. ^{1}H NMR spectrum of compound **1l'** in $CDCl_3$

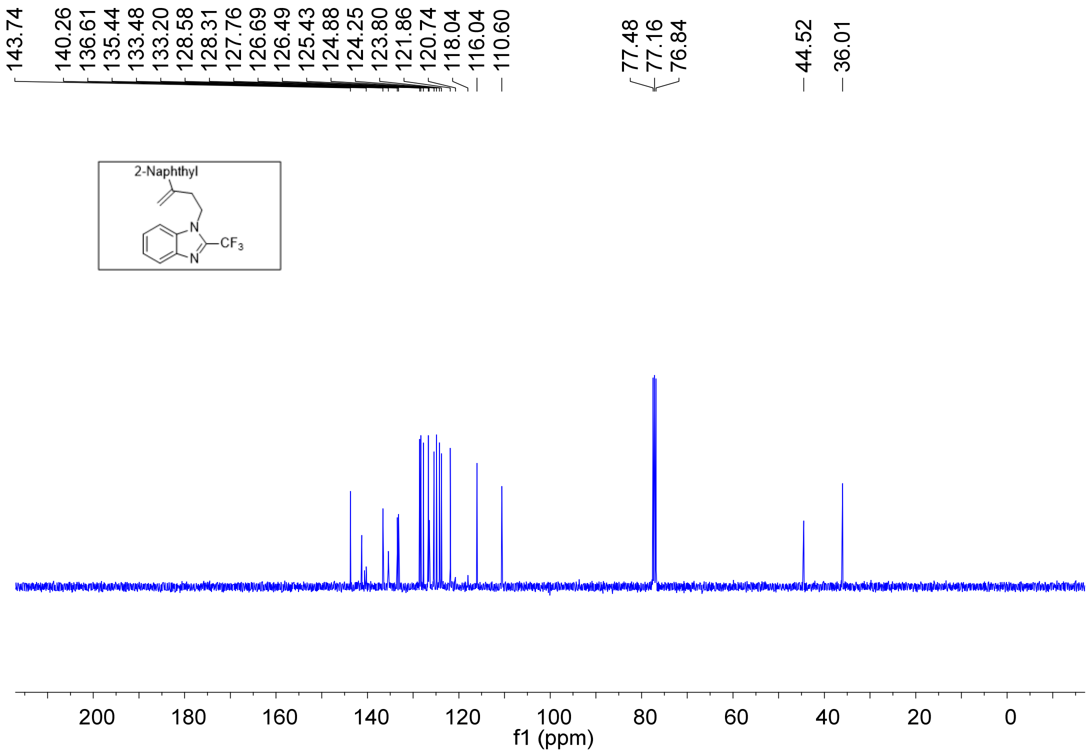

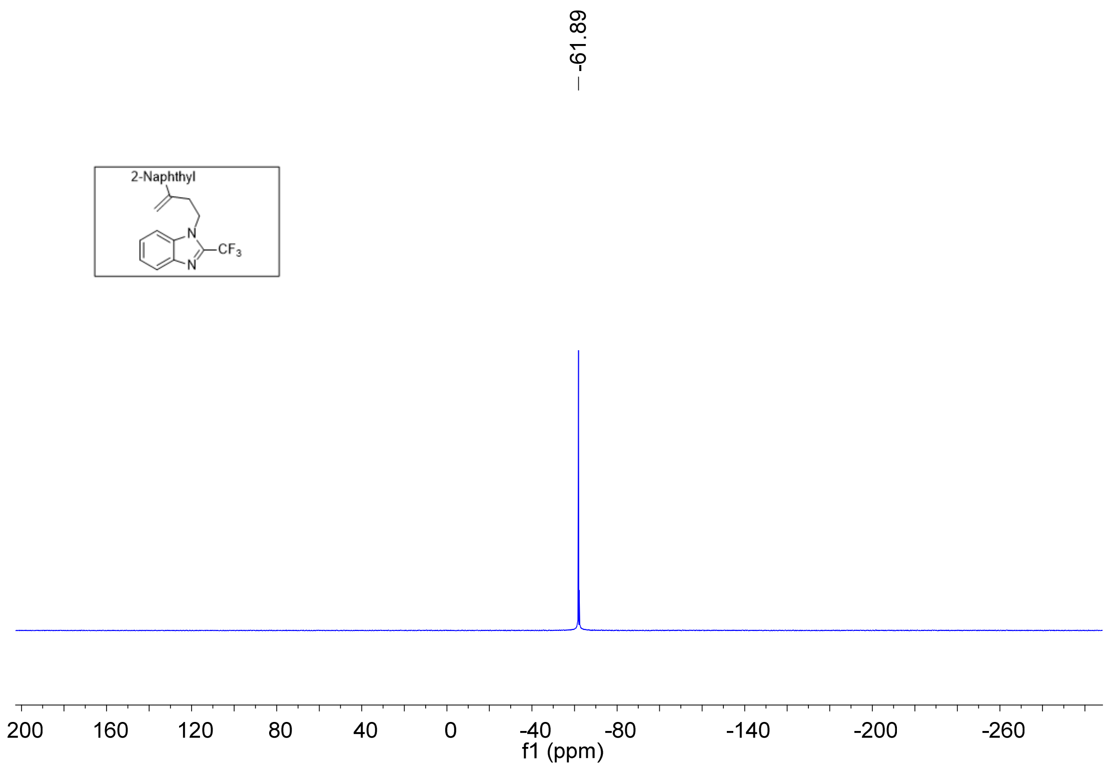

Supplementary Figure 66. ^{13}C and ^{19}F NMR spectra of compound **1l'** in $CDCl_3$

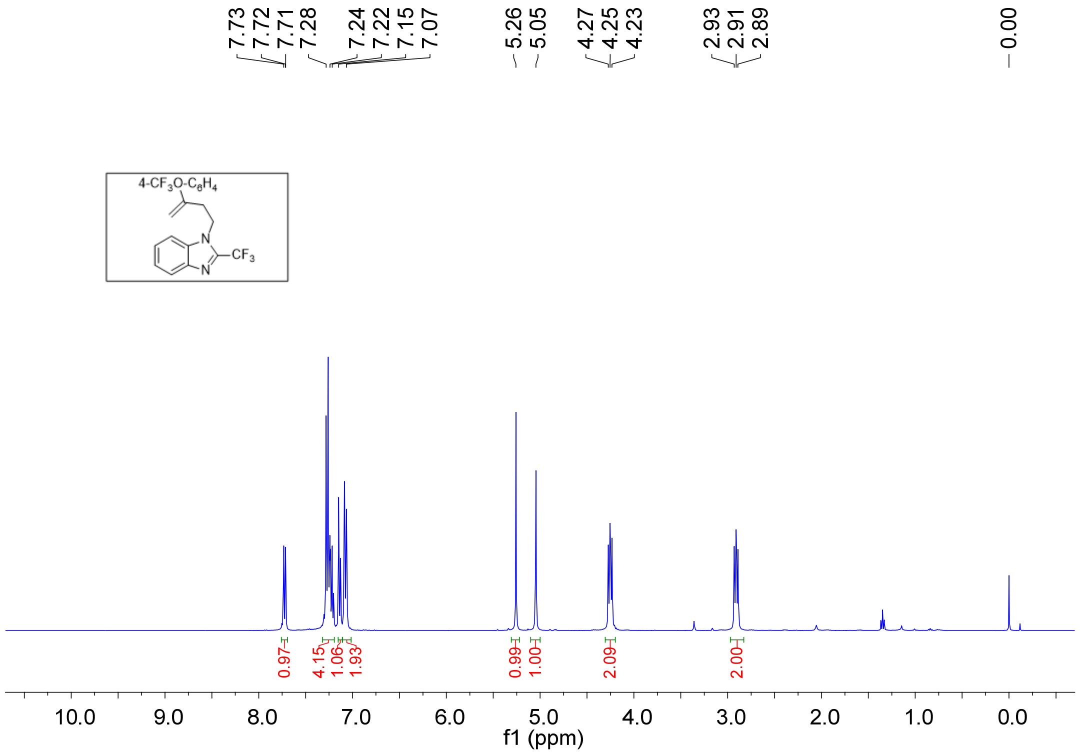

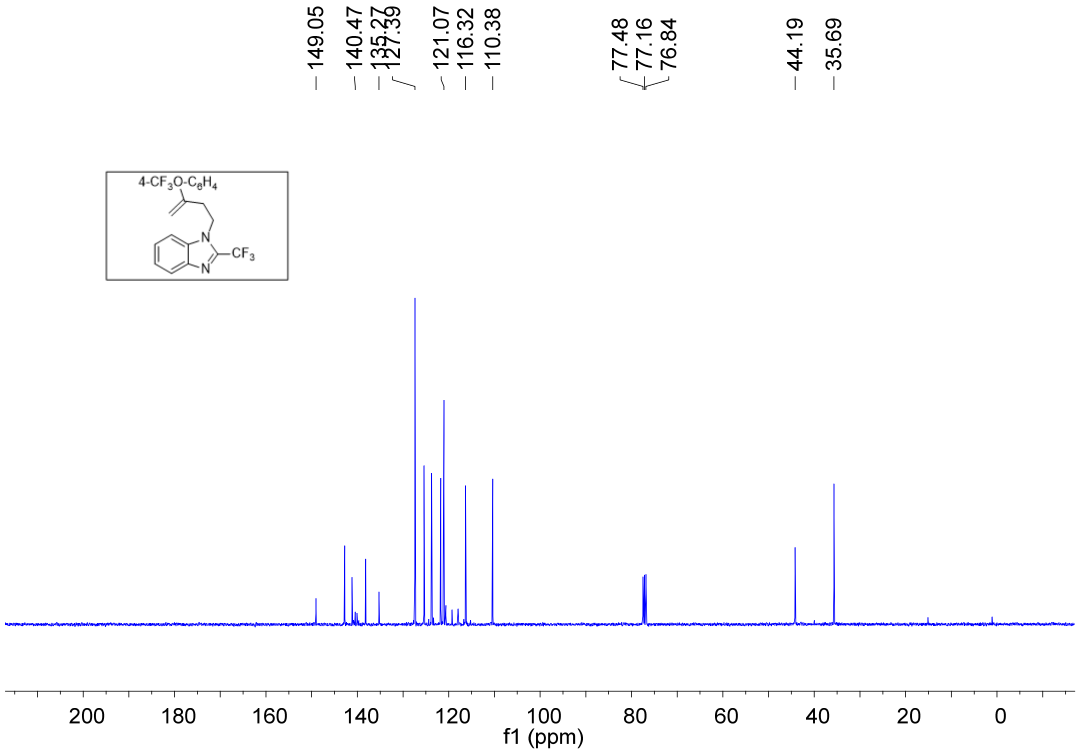

Supplementary Figure 67. ^{1}H and ^{13}C NMR spectra of compound **1m’** in $CDCl_3$.

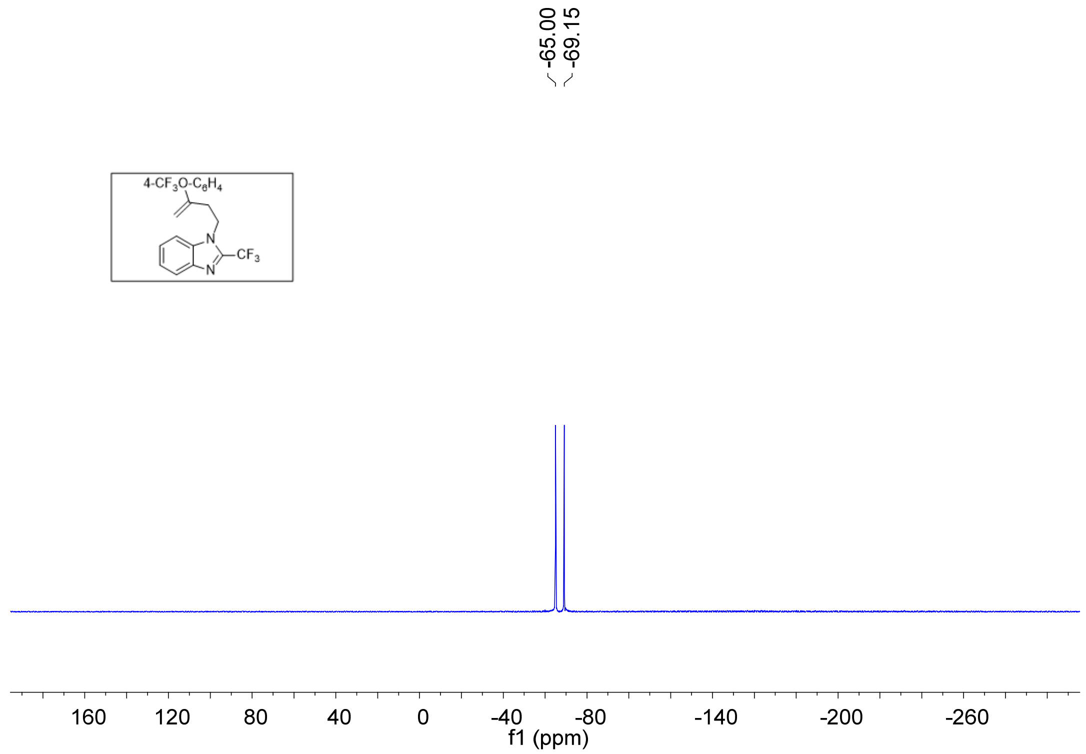

Supplementary Figure 68. ^{19}F NMR spectrum of compound **1m'** in $CDCl_3$.

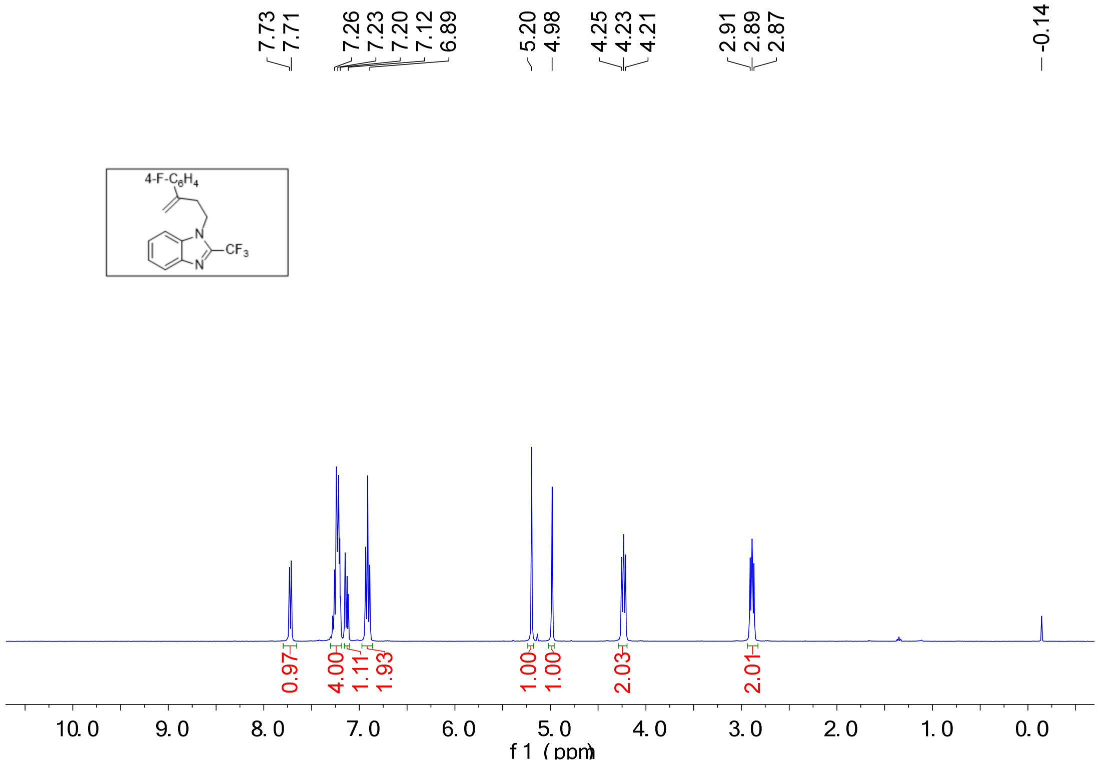

Supplementary Figure 69. ^{1}H spectrum of compound **1n'** in $CDCl_3$.

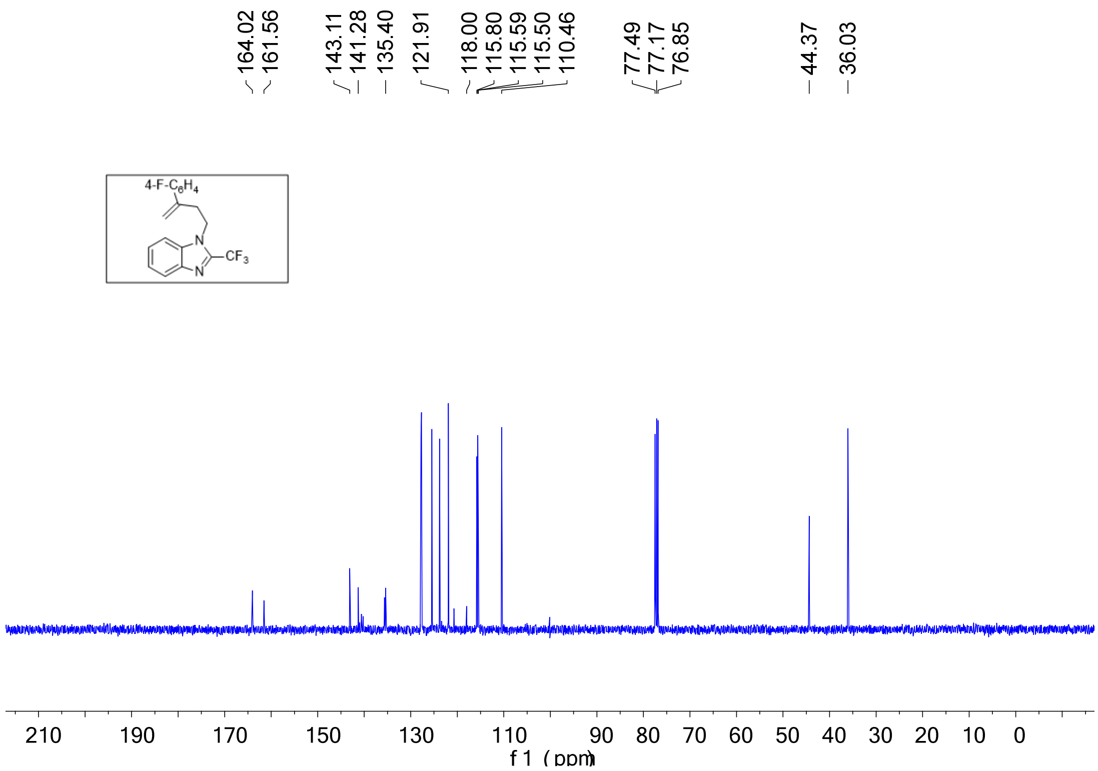

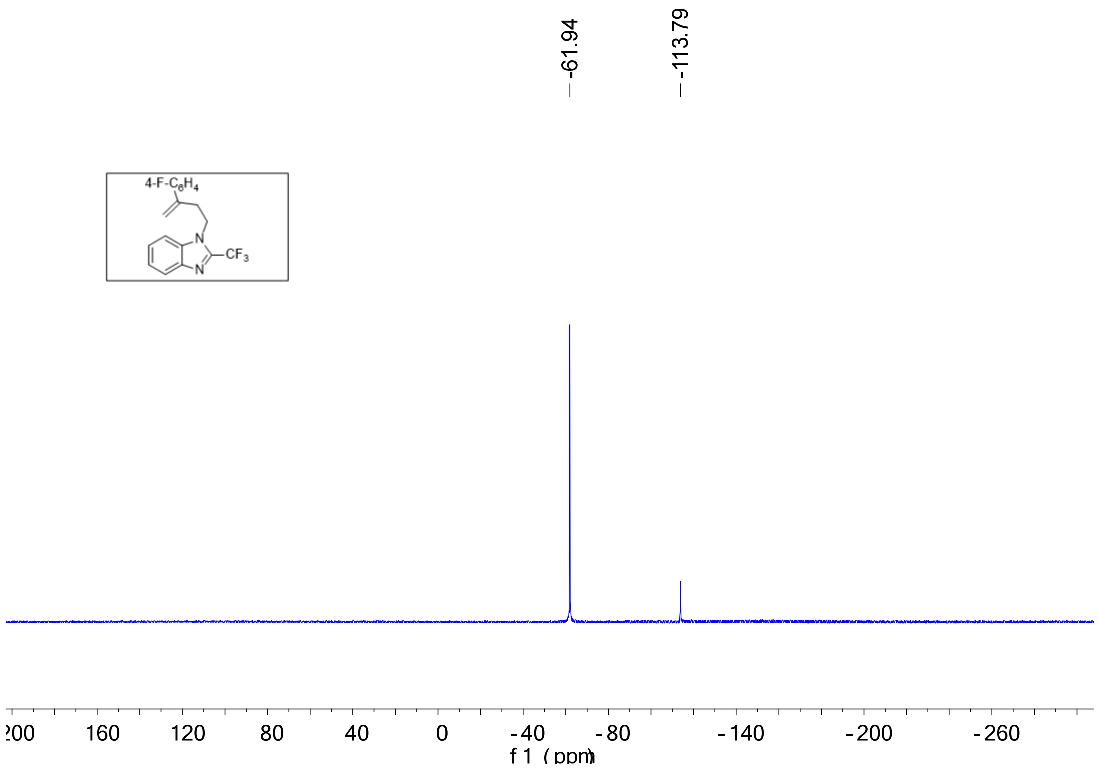

Supplementary Figure 70. ^{13}C and ^{19}F NMR spectra of compound **1n'** in $CDCl_3$.

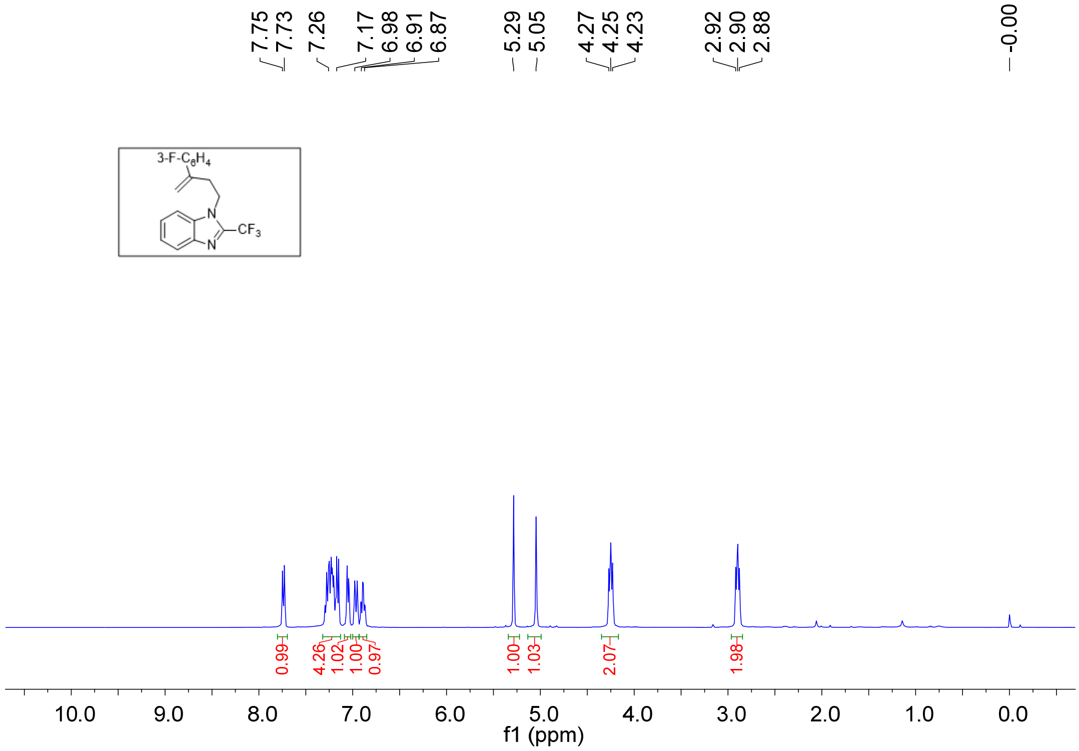

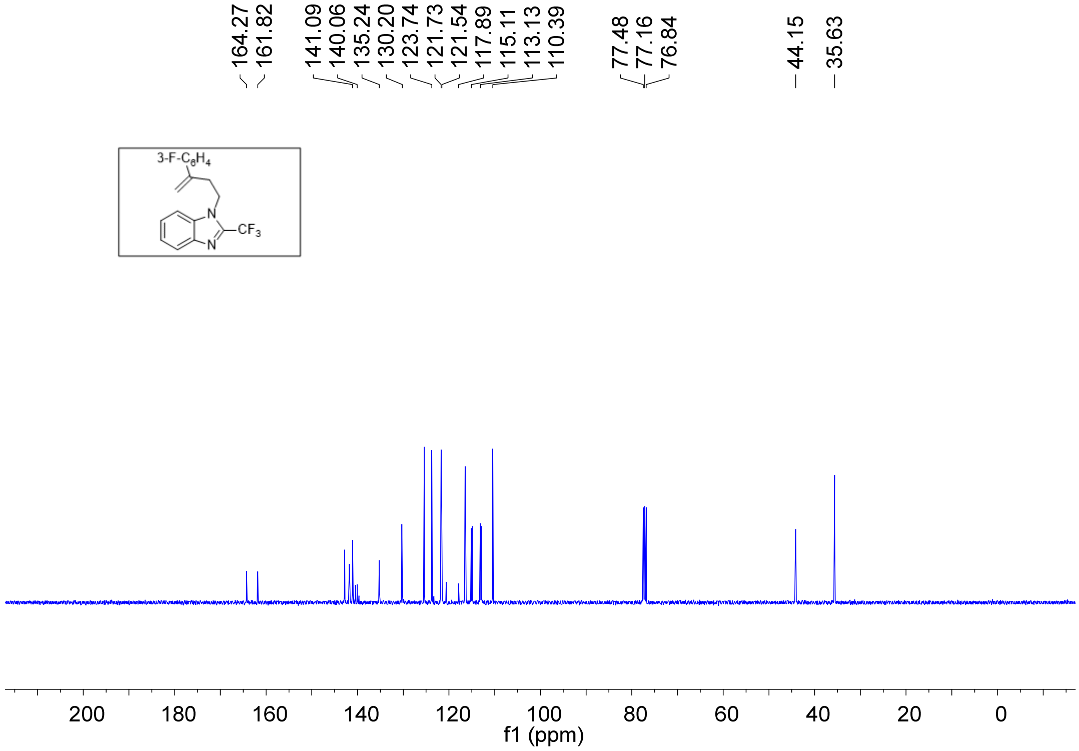

Supplementary Figure 71. ^{1}H and ^{13}C NMR spectra of compound **1o'** in $CDCl_3$.

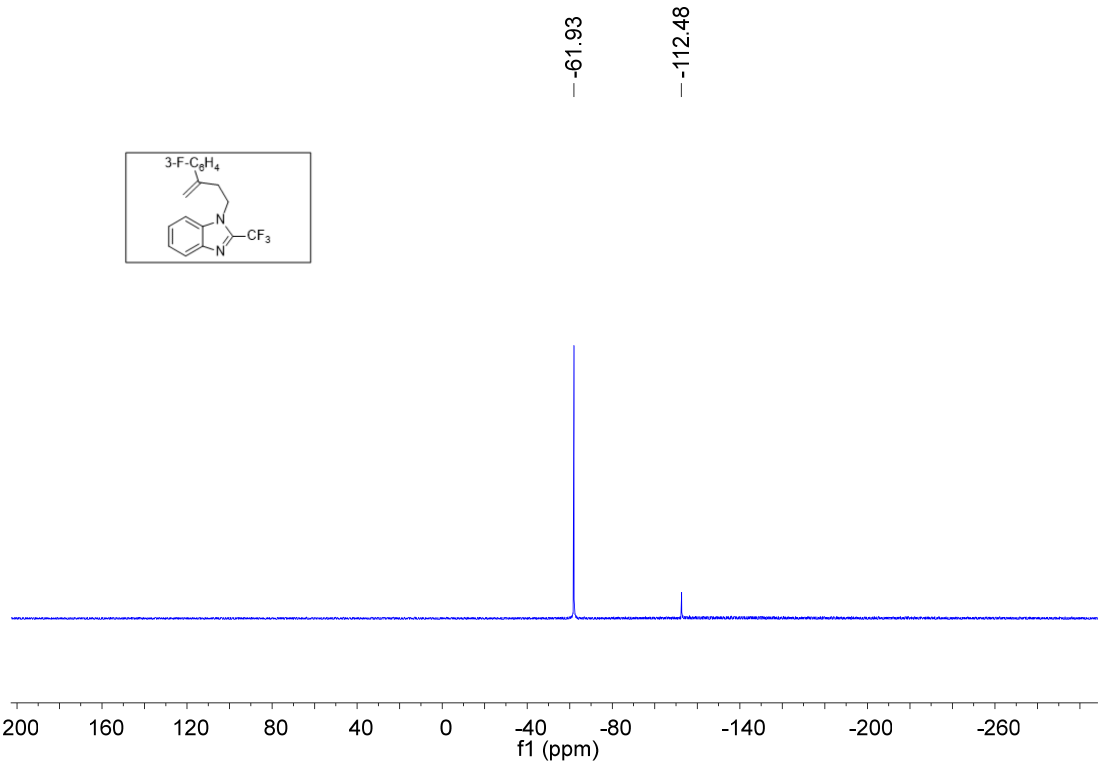

Supplementary Figure 72. ^{19}F NMR spectrum of compound **1o'** in $CDCl_3$

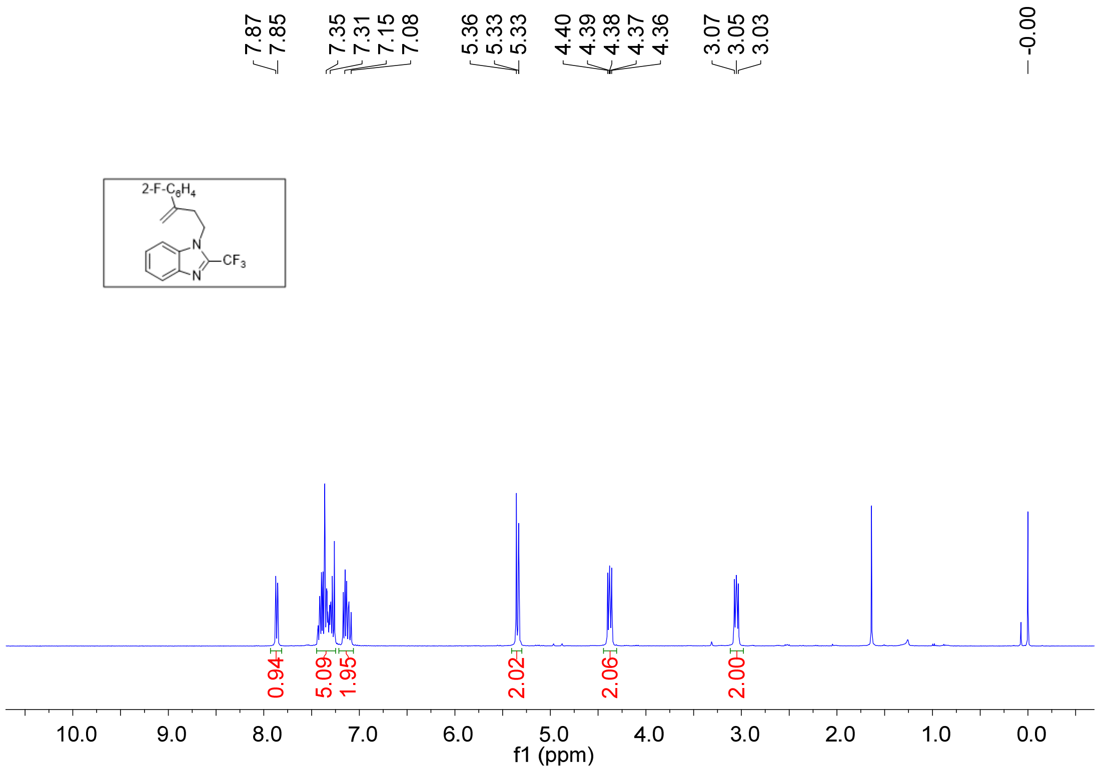

Supplementary Figure 73. ^{1}H NMR spectrum of compound **1p'** in $CDCl_3$.

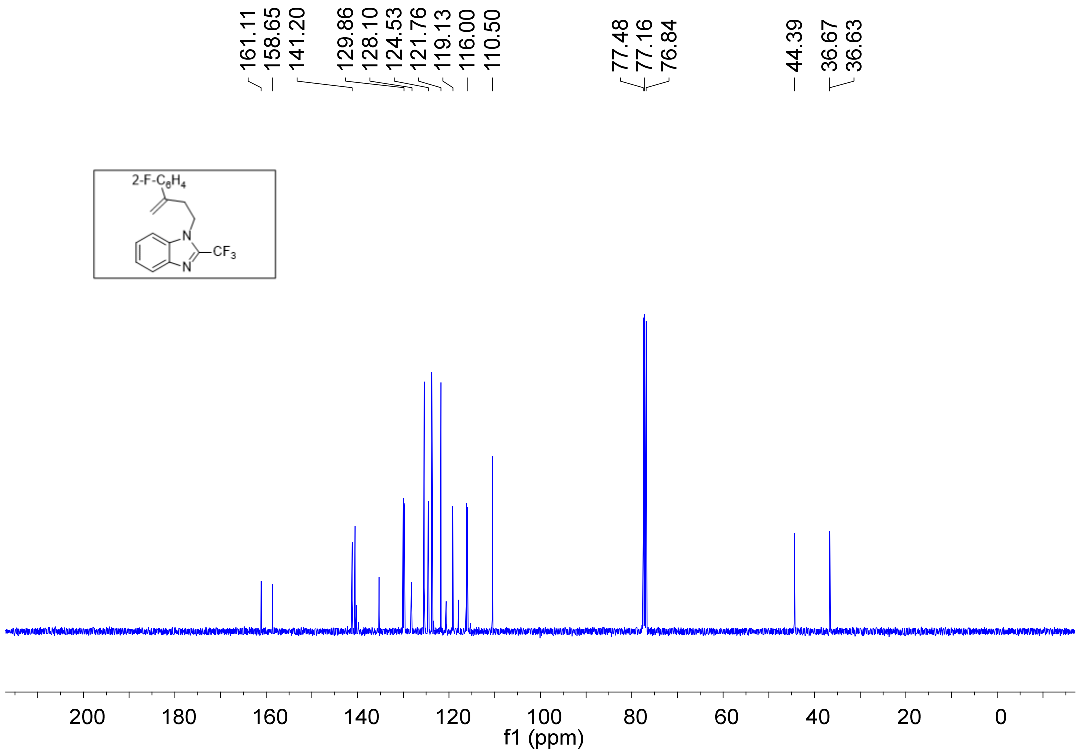

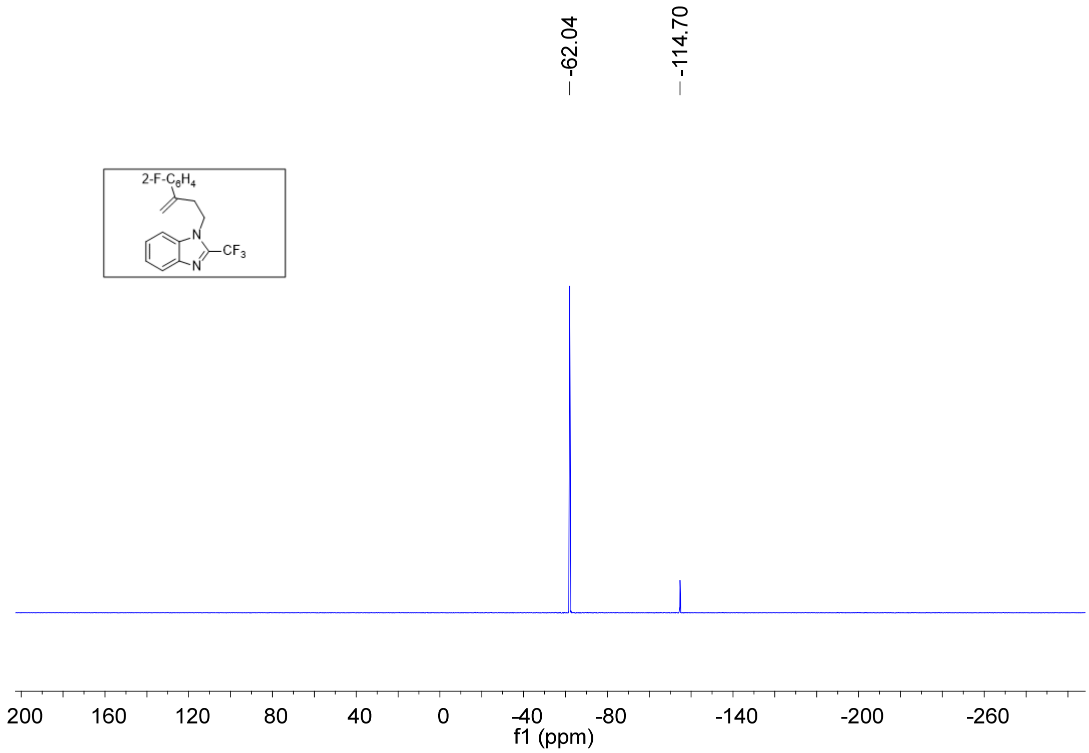

Supplementary Figure 74. ^{13}C and ^{19}F NMR spectra of compound **1p'** in $CDCl_3$.

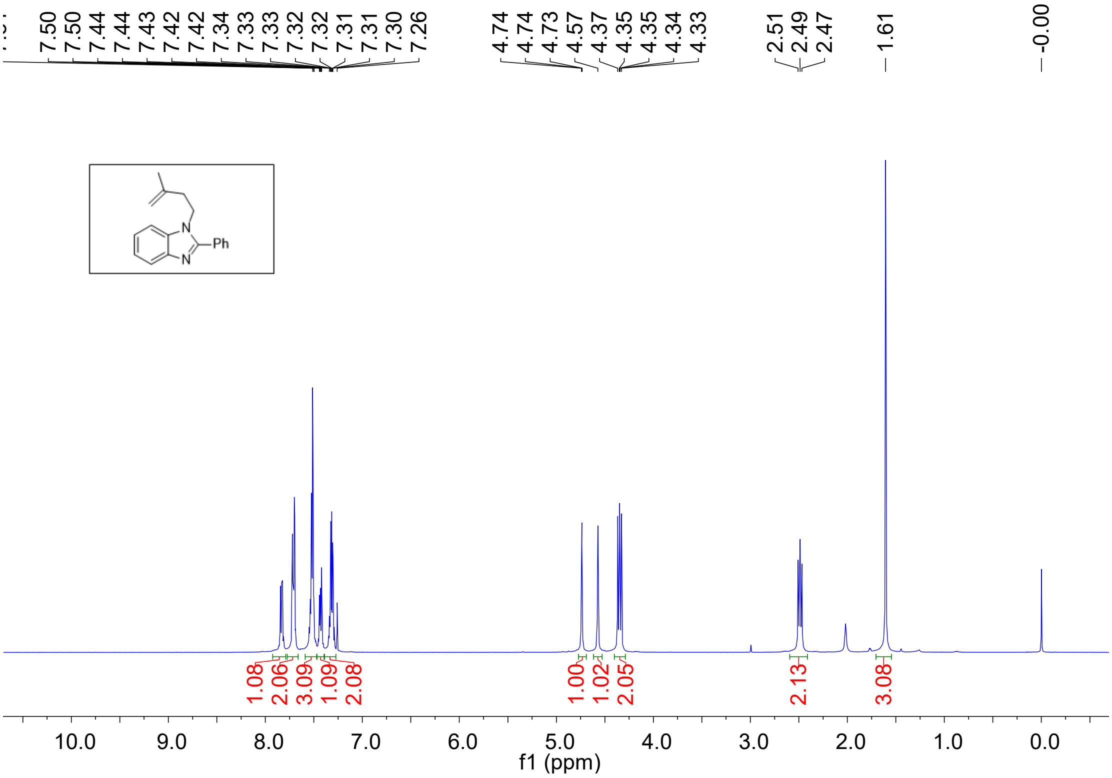

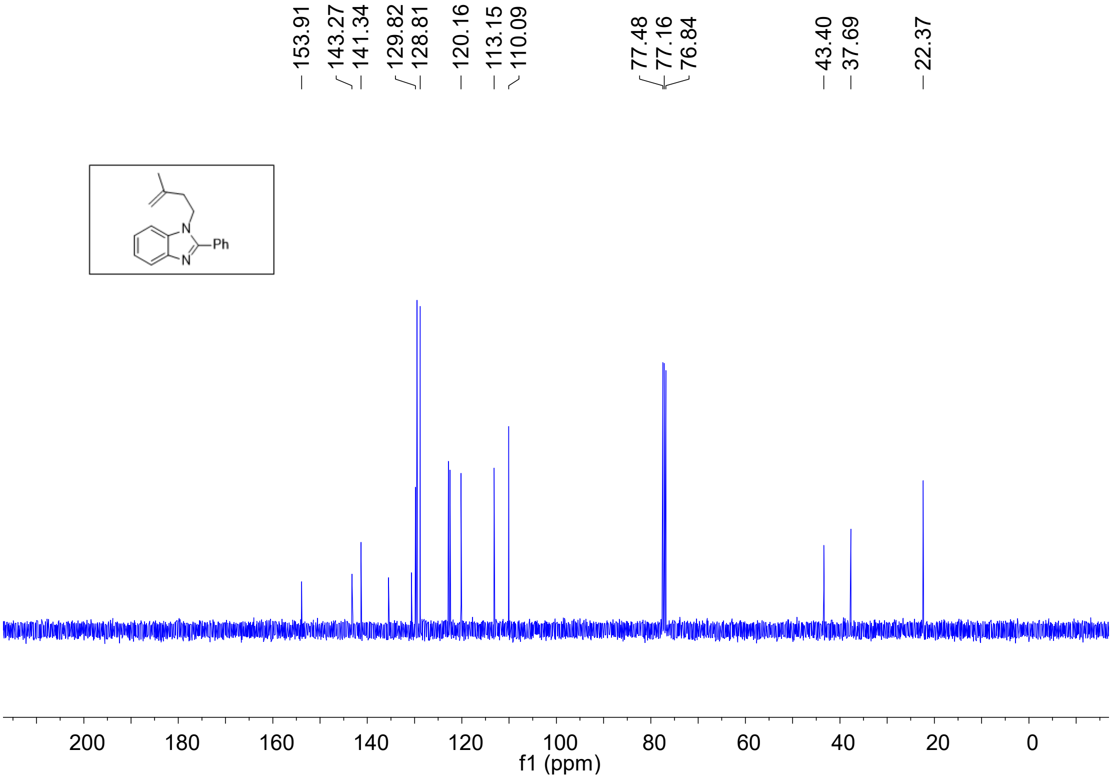

Supplementary Figure 75. ^{1}H and ^{13}C NMR spectra of compound **1r’** in $CDCl_3$.

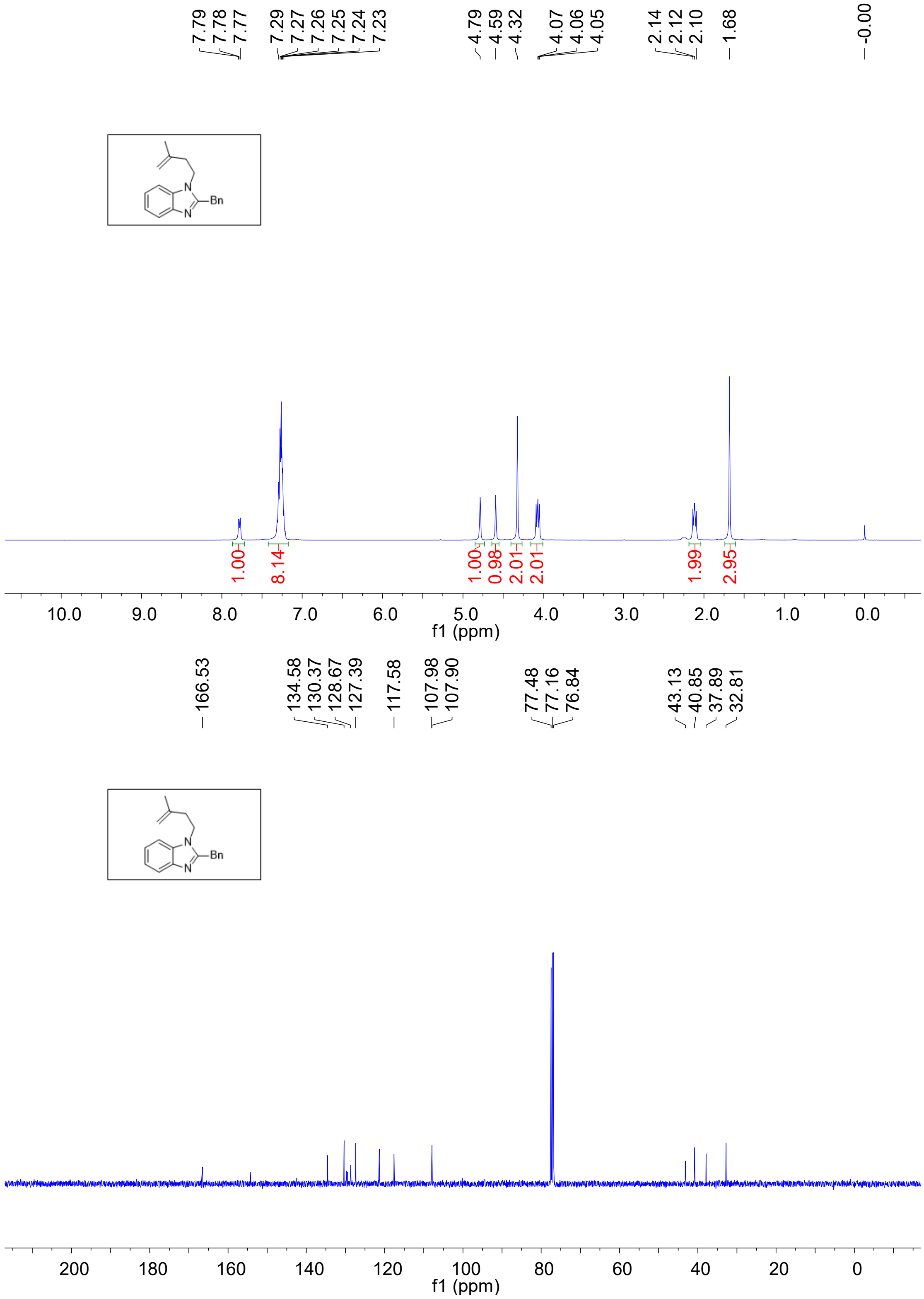

Supplementary Figure 76. ^{1}H and ^{13}C NMR spectra of compound **1s'** in $CDCl_3$

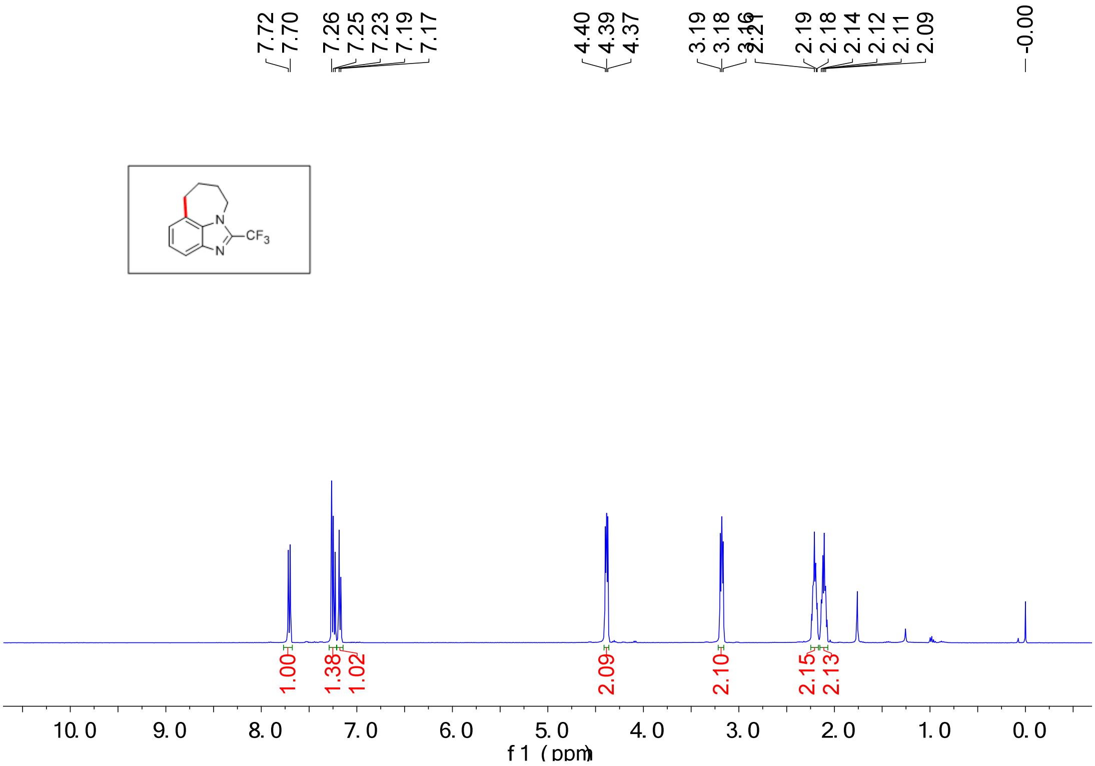

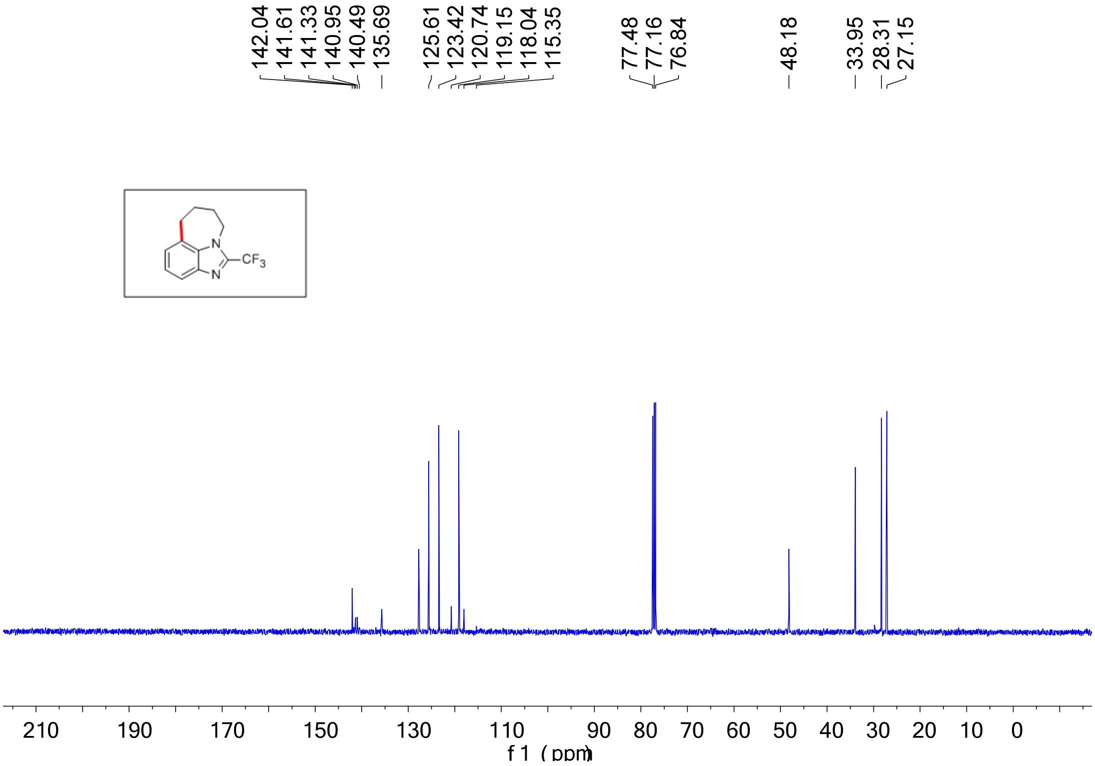

Supplementary Figure 77. ^{1}H and ^{13}C NMR spectra of compound **2a** in $CDCl_3$.

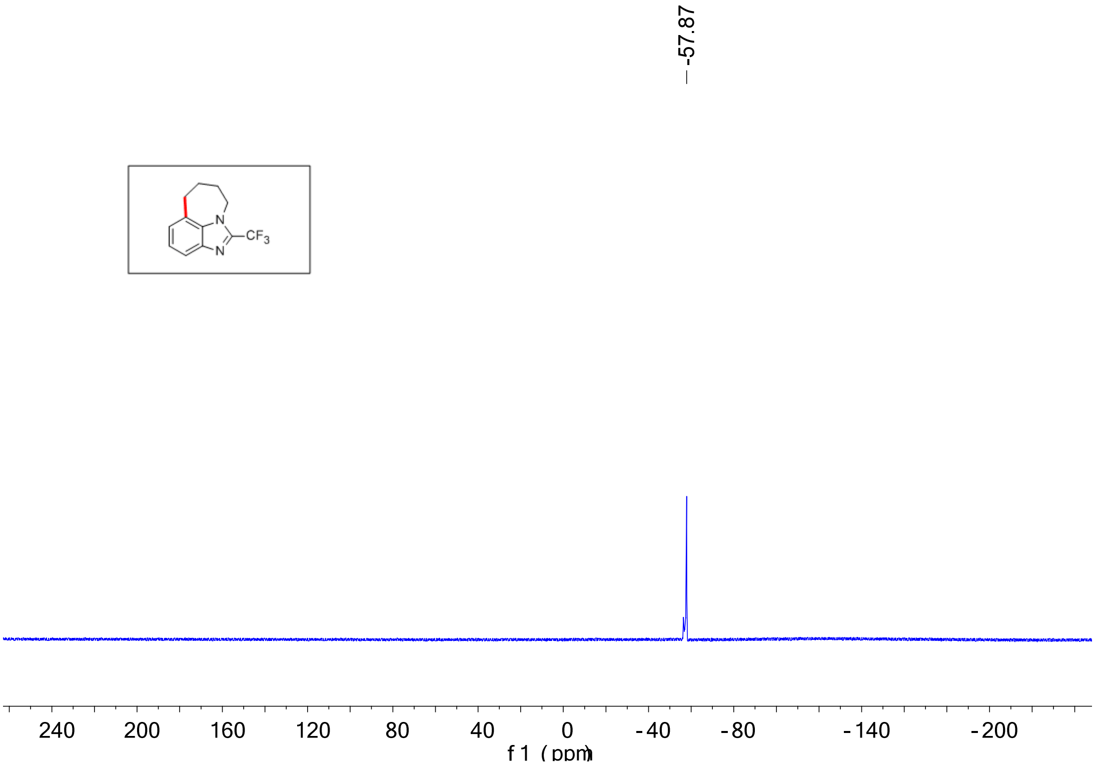

Supplementary Figure 78. ^{19}F NMR spectrum of compound **2a** in $CDCl_3$.

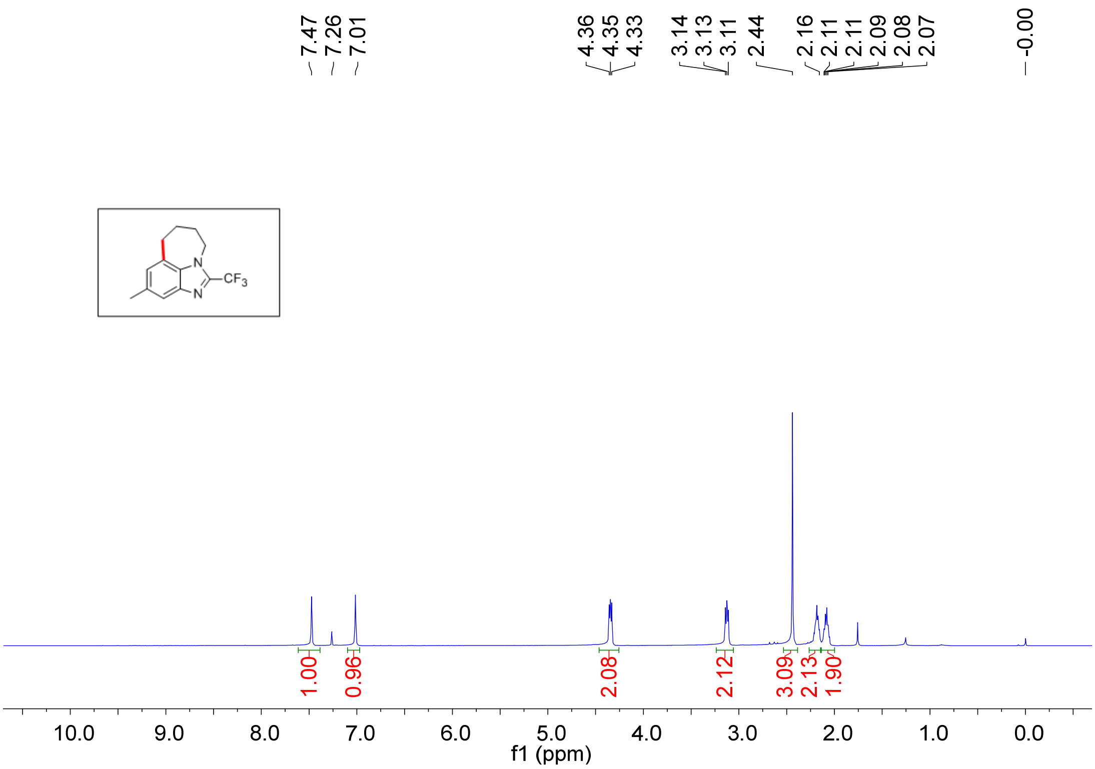

Supplementary Figure 79. ^{1}H NMR spectrum of compound **2b** in $CDCl_3$.

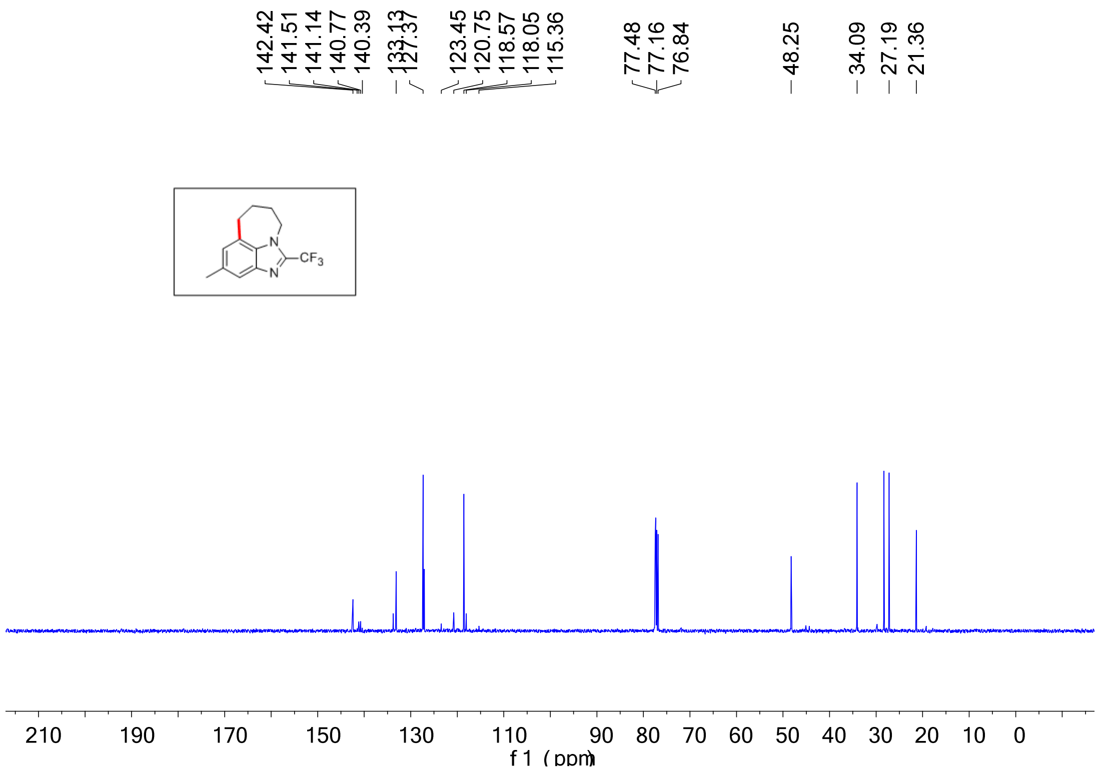

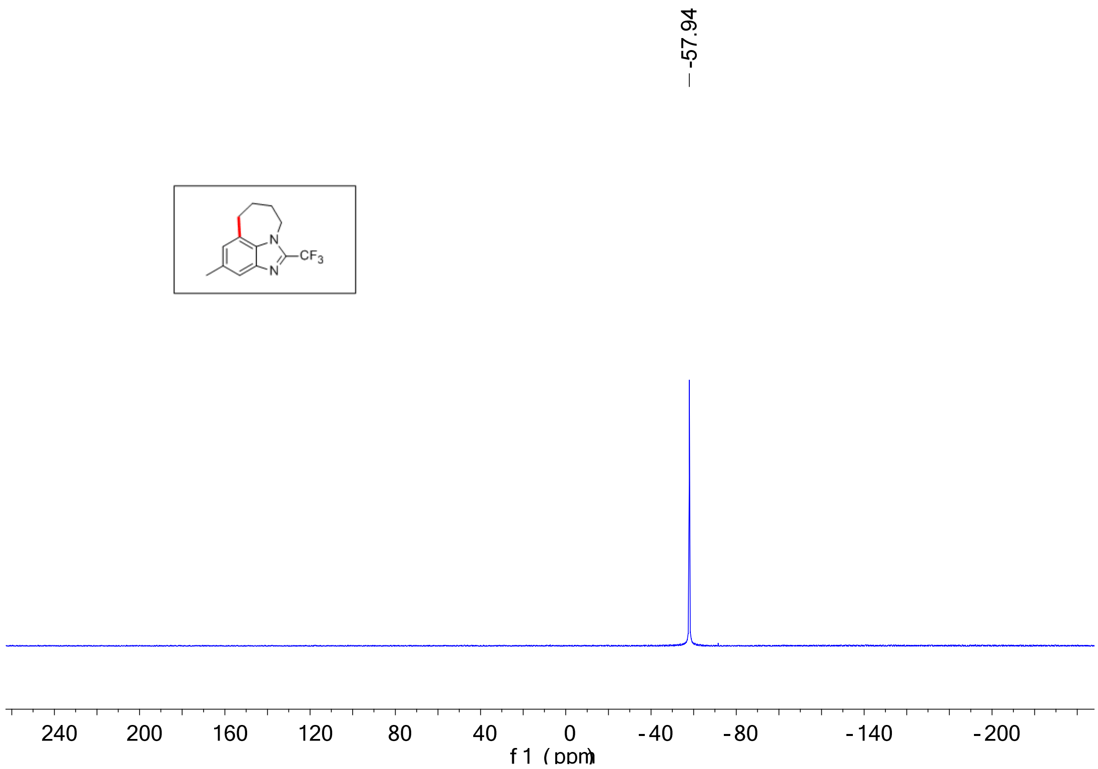

Supplementary Figure 80. ^{13}C and ^{19}F NMR spectra of compound **2b** in $CDCl_3$.

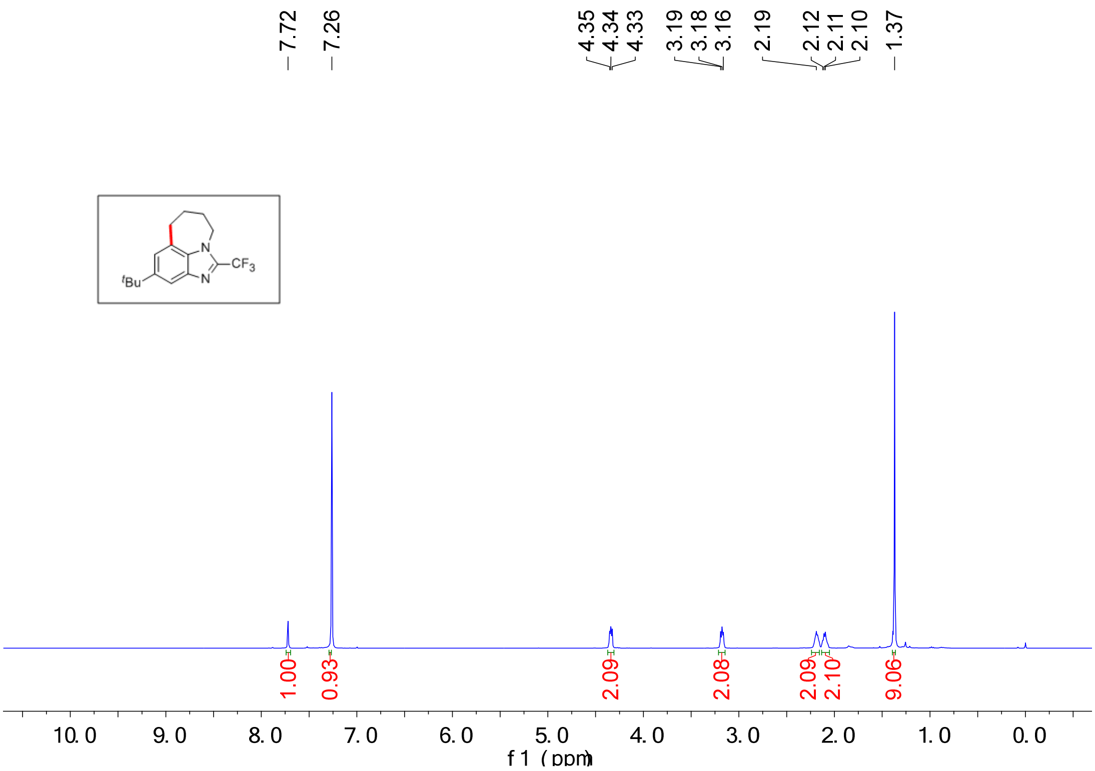

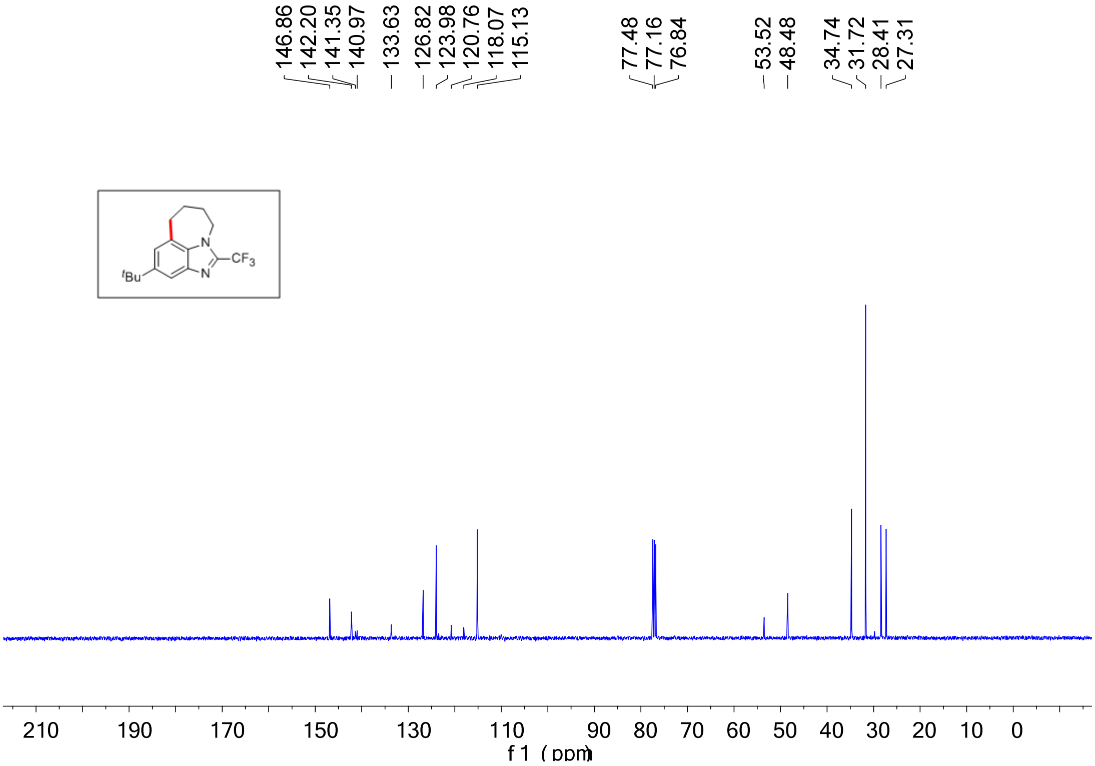

Supplementary Figure 81. ^{1}H and ^{13}C NMR spectra of compound **2c** in $CDCl_3$.

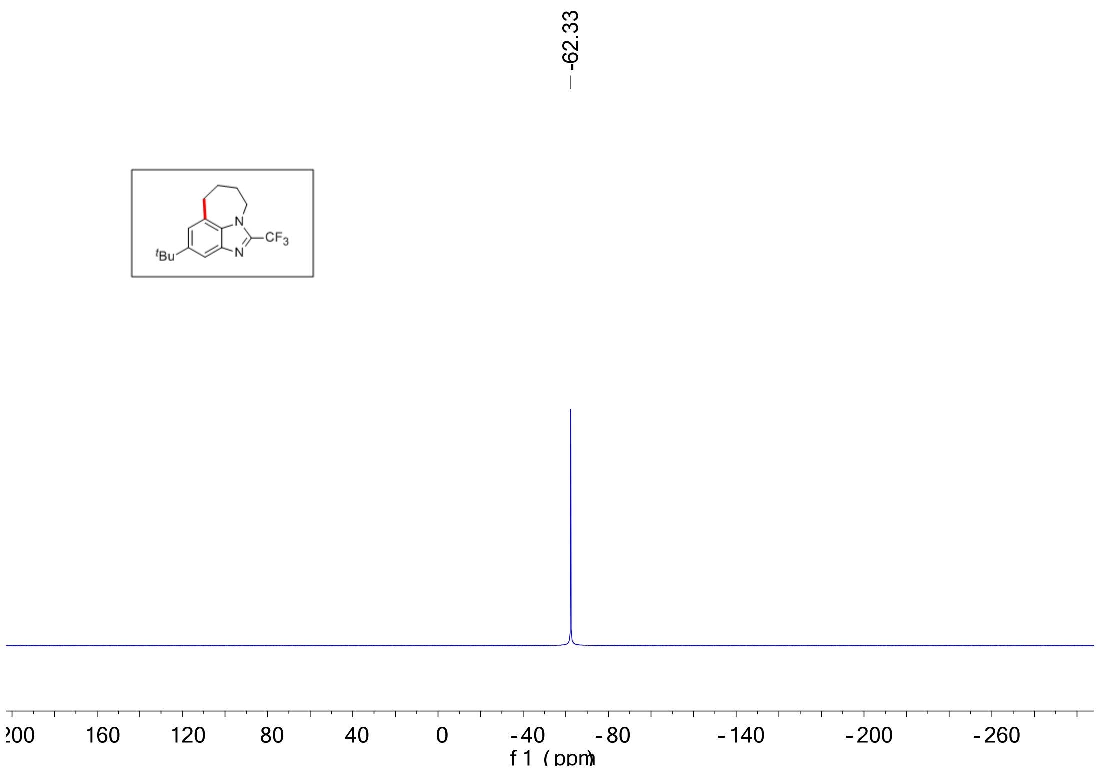

Supplementary Figure 82. ^{19}F NMR spectrum of compound **2c** in $CDCl_3$.

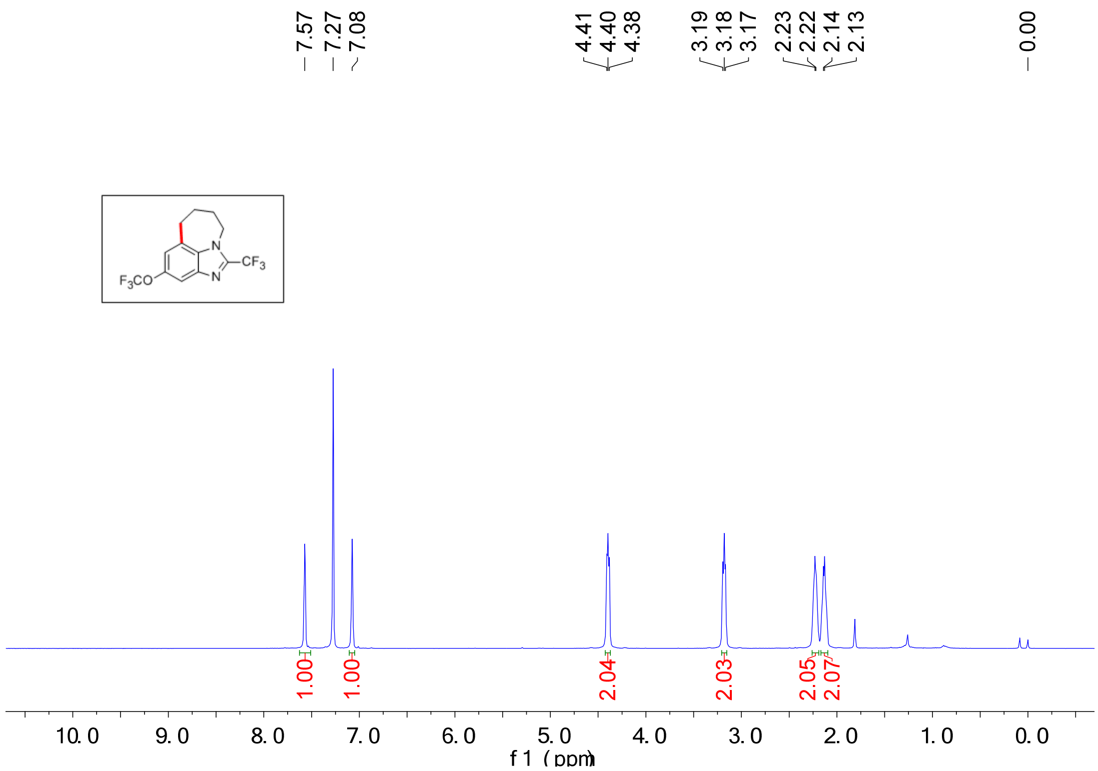

Supplementary Figure 83. ^{1}H NMR spectrum of compound **2d** in $CDCl_3$.

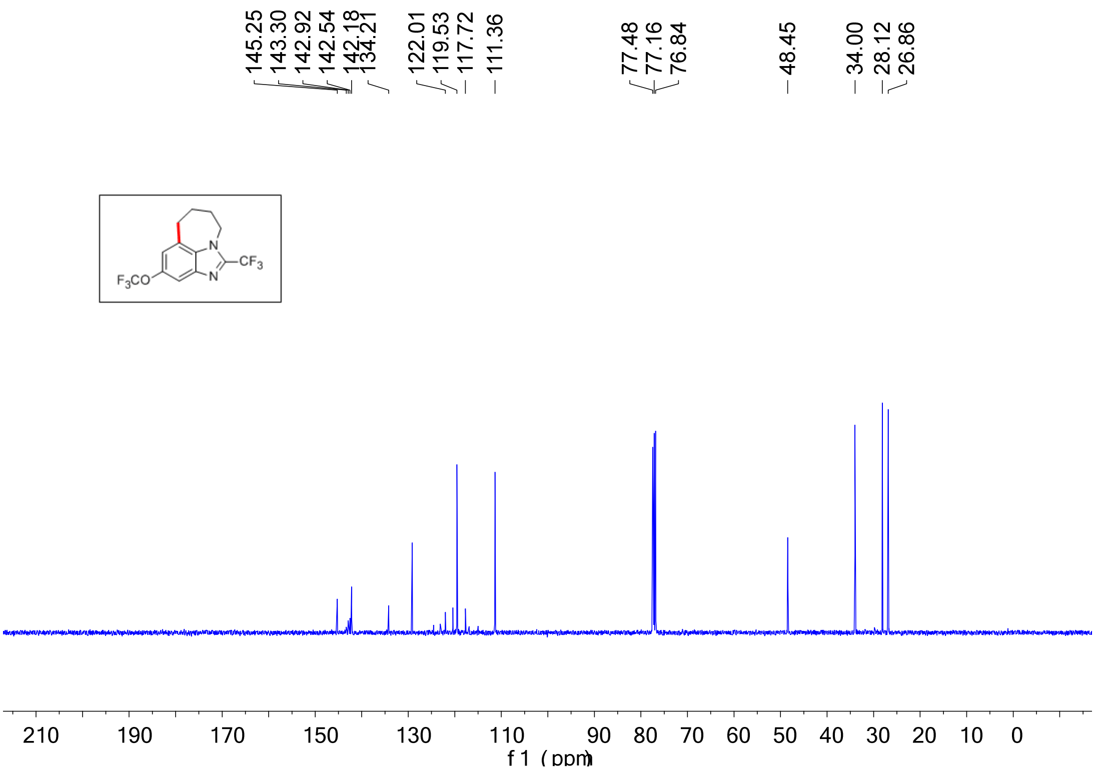

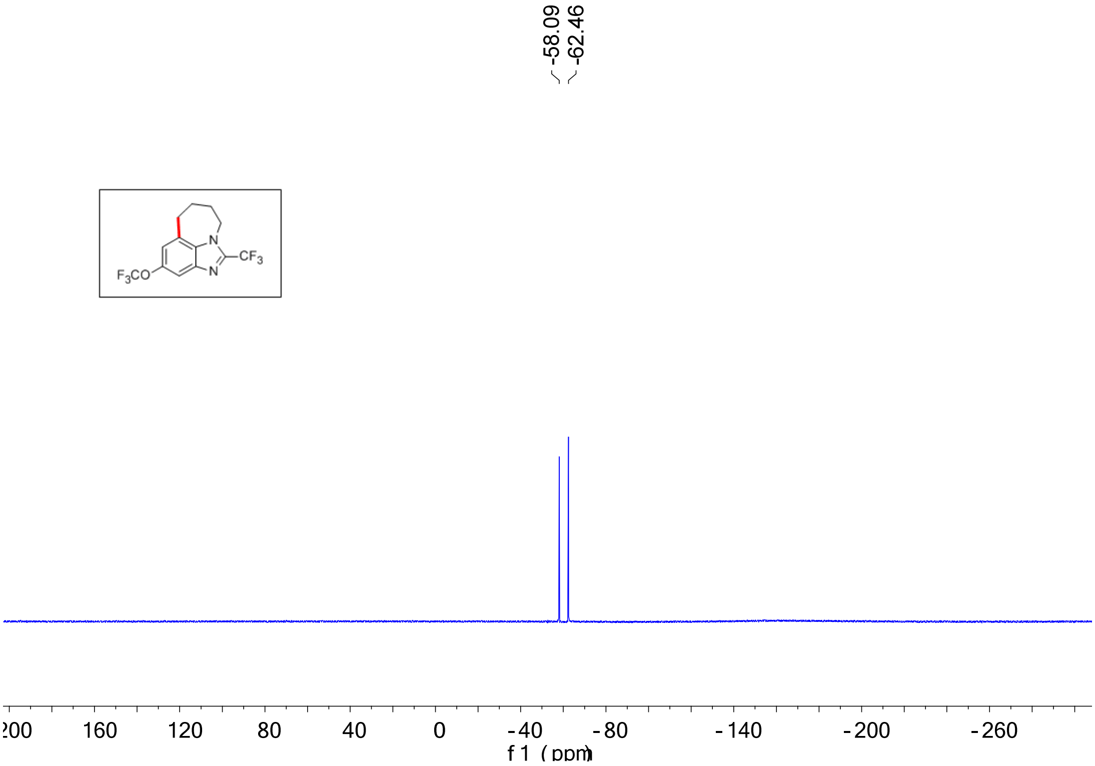

Supplementary Figure 84. ^{13}C and ^{19}F NMR spectra of compound **2d** in $CDCl_3$.

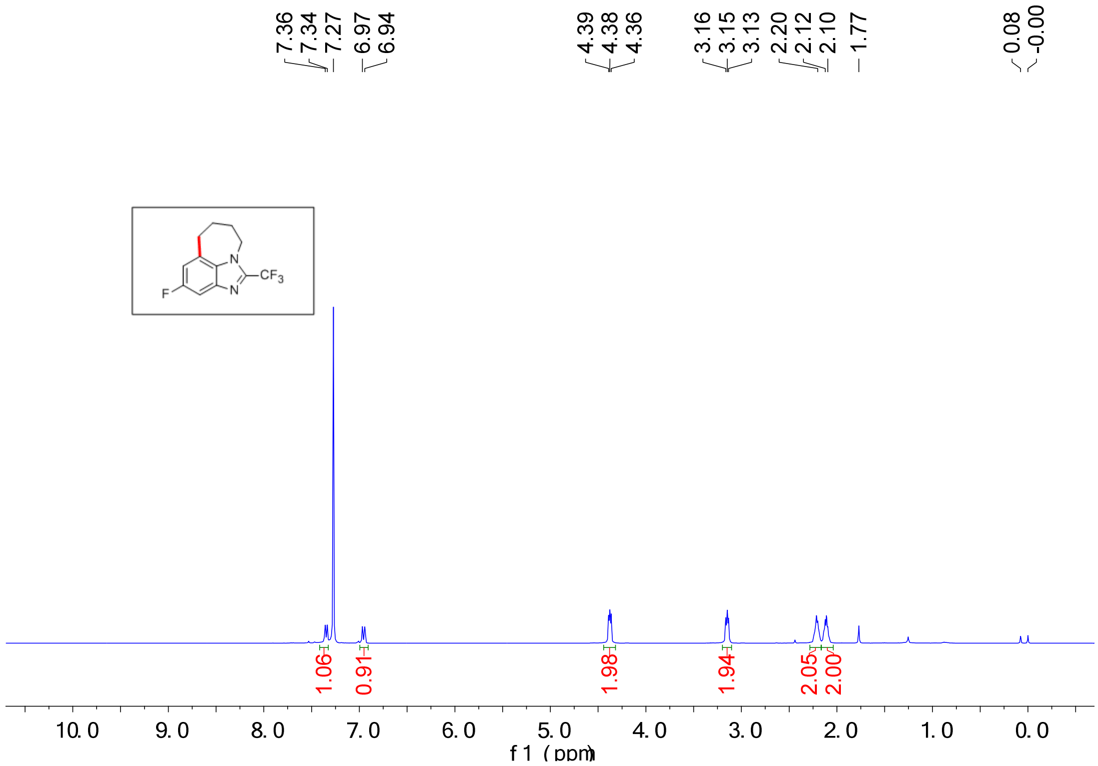

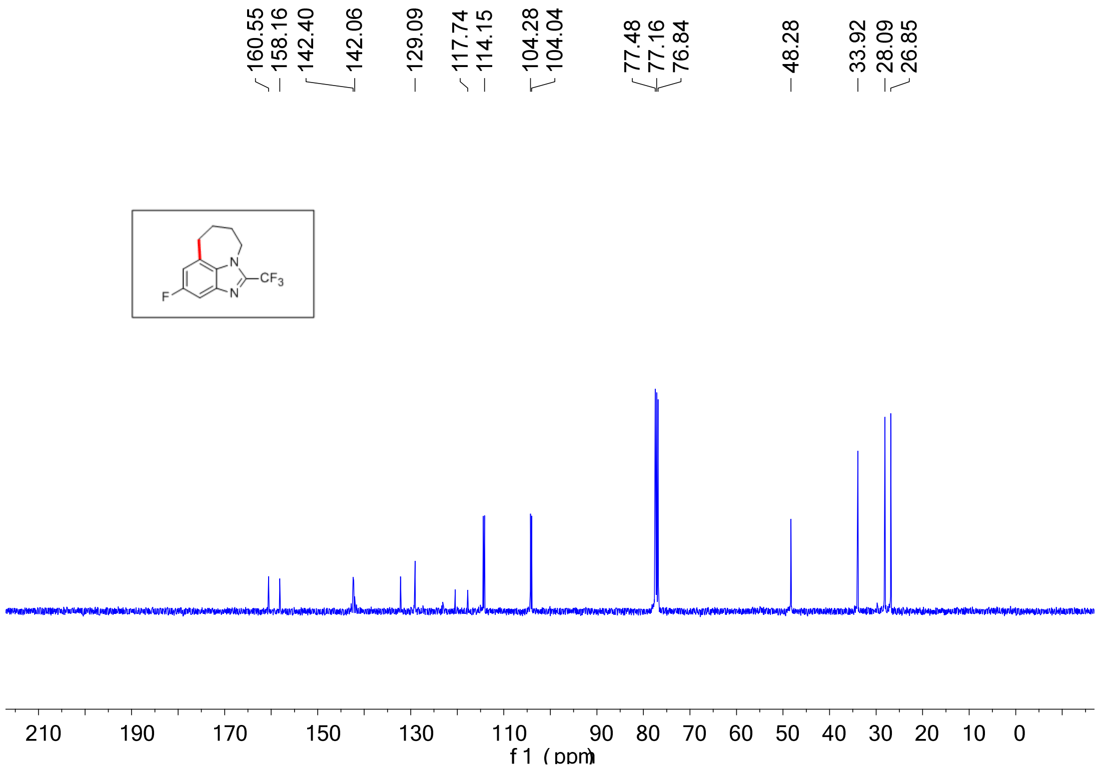

Supplementary Figure 85. ^{1}H and ^{13}C NMR spectra of compound **2e** in $CDCl_3$.

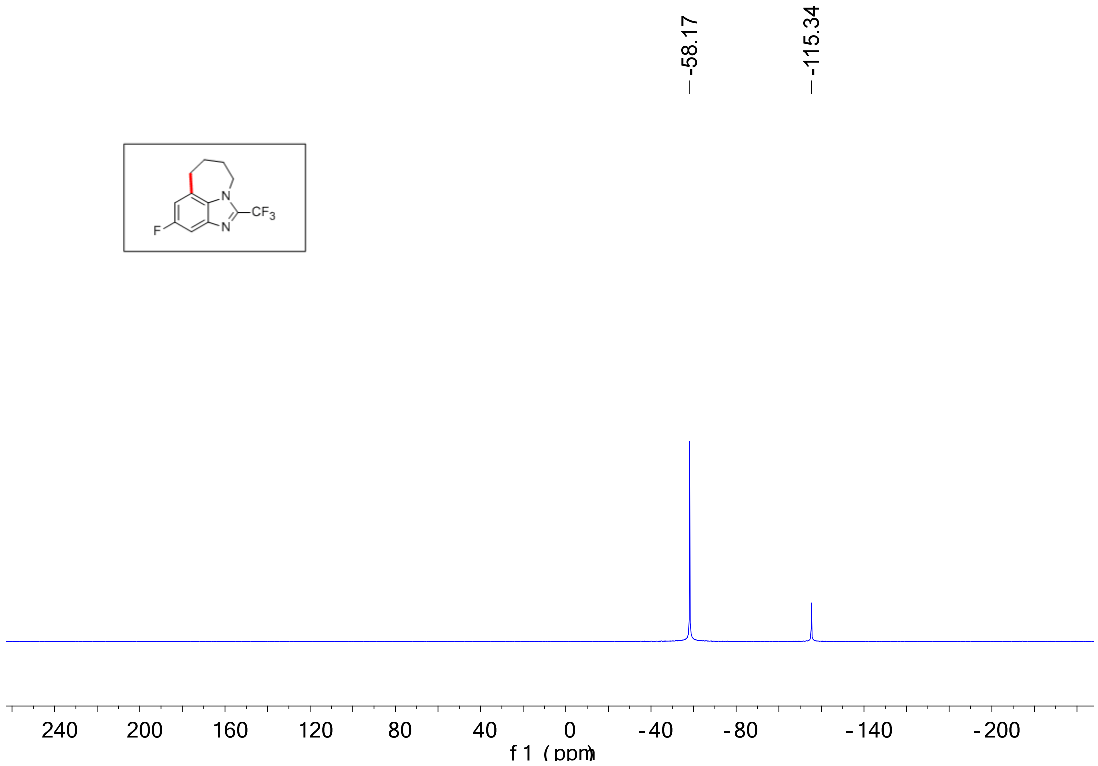

Supplementary Figure 86. ^{19}F NMR spectrum of compound **2e** in $CDCl_3$.

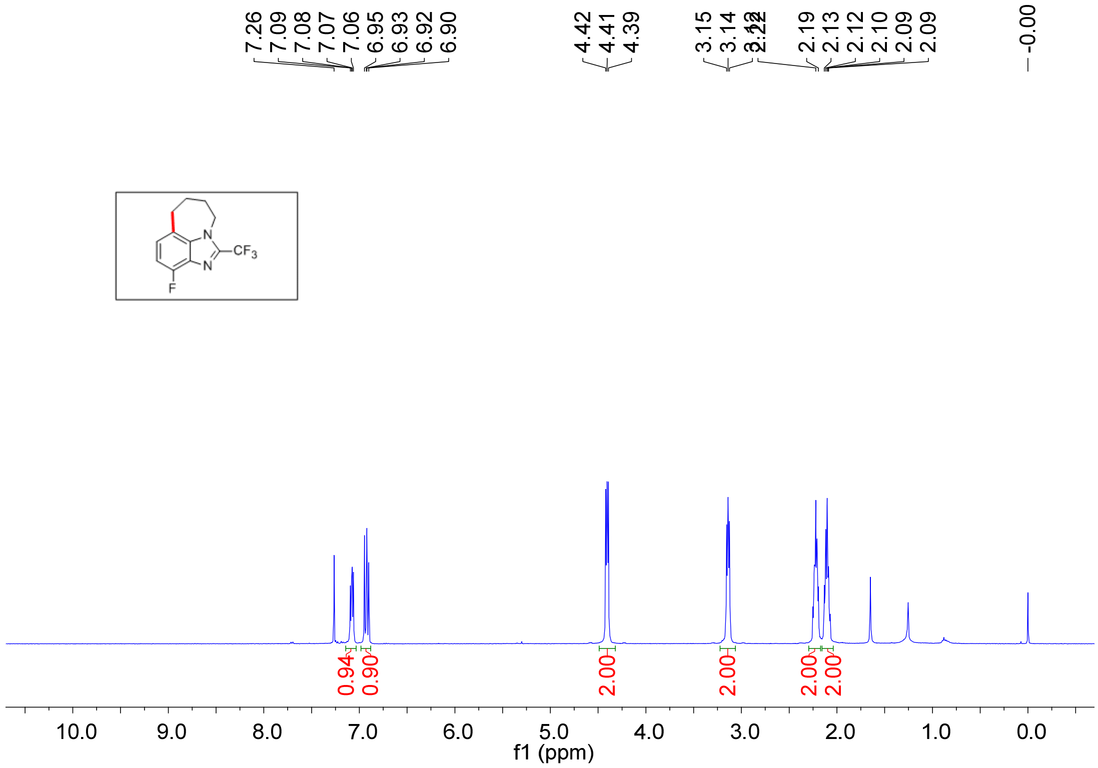

Supplementary Figure 87. ^{1}H NMR spectrum of compound **2f** in $CDCl_3$.

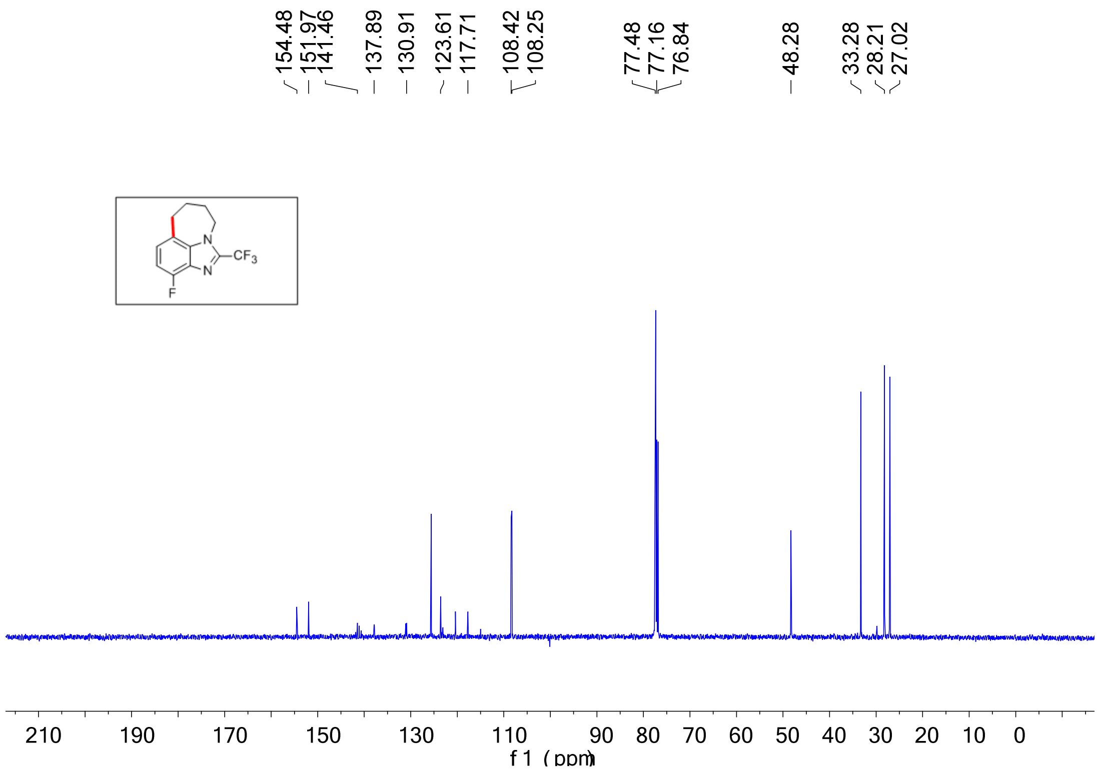

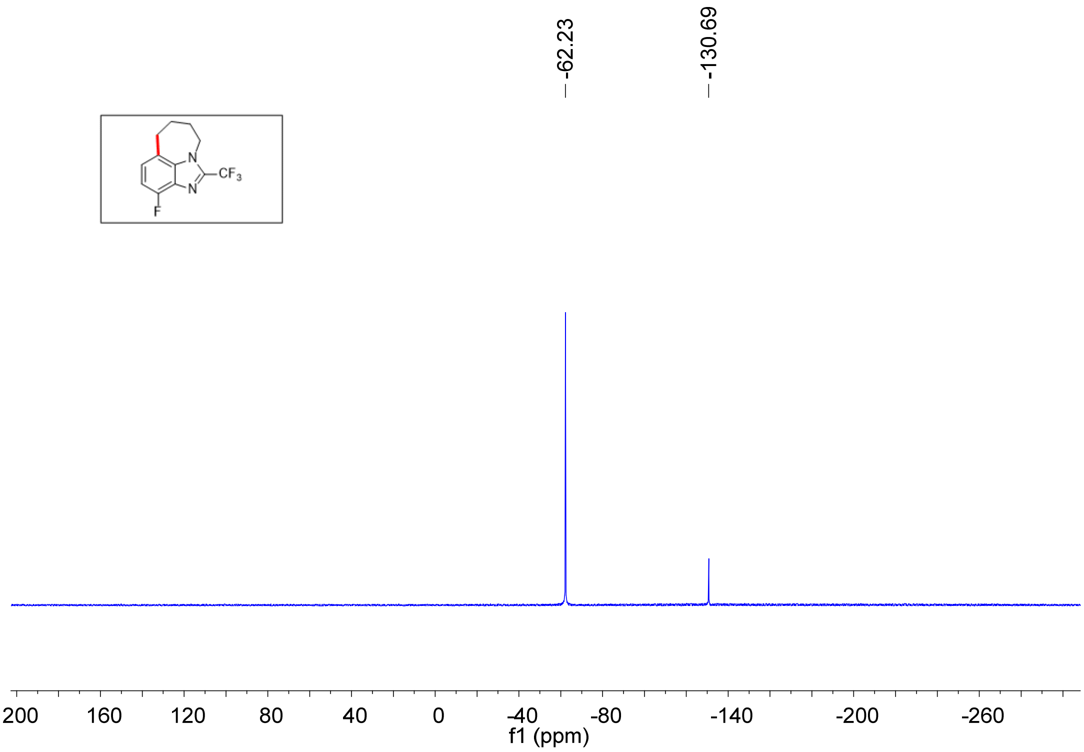

Supplementary Figure 88. ^{13}C and ^{19}F NMR spectra of compound **2f** in $CDCl_3$.

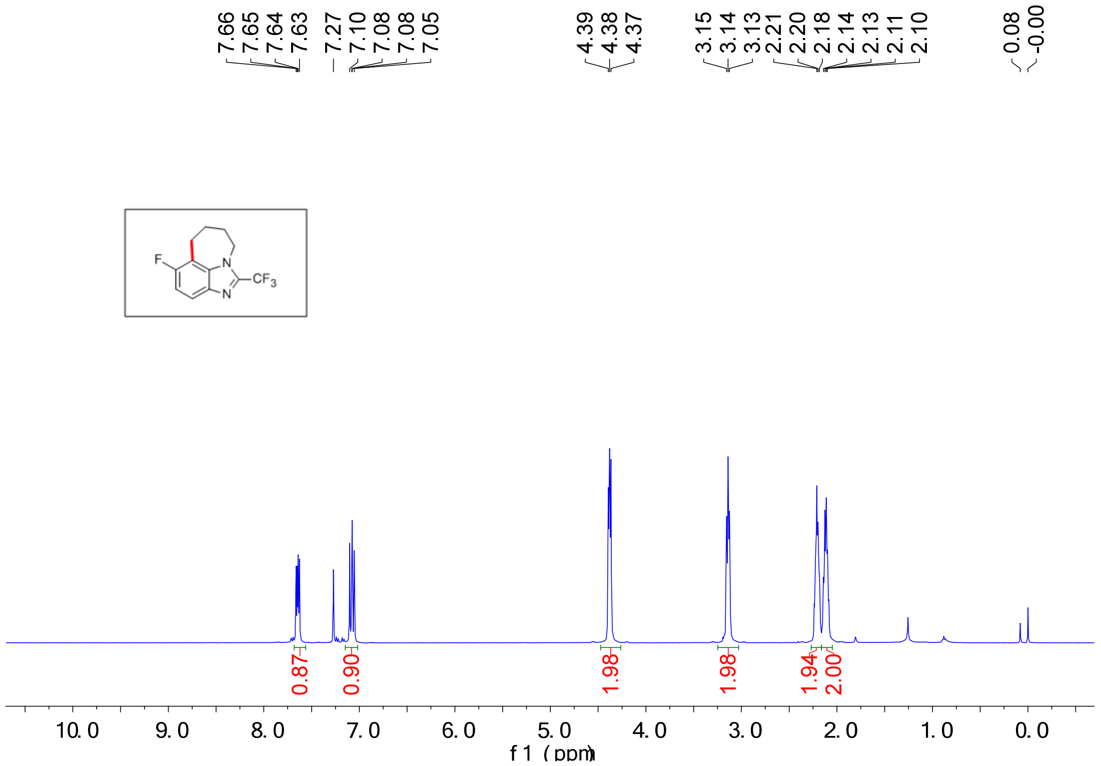

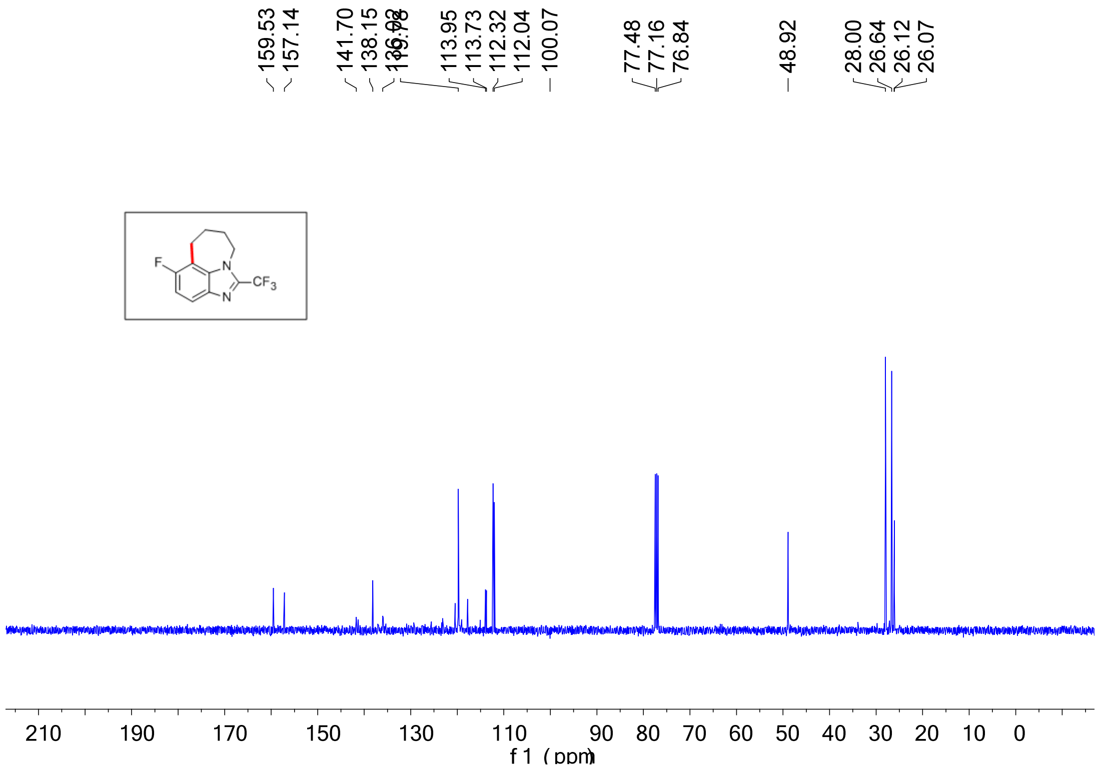

Supplementary Figure 89. ^{1}H and ^{13}C NMR spectra of compound **2g** in $CDCl_3$.

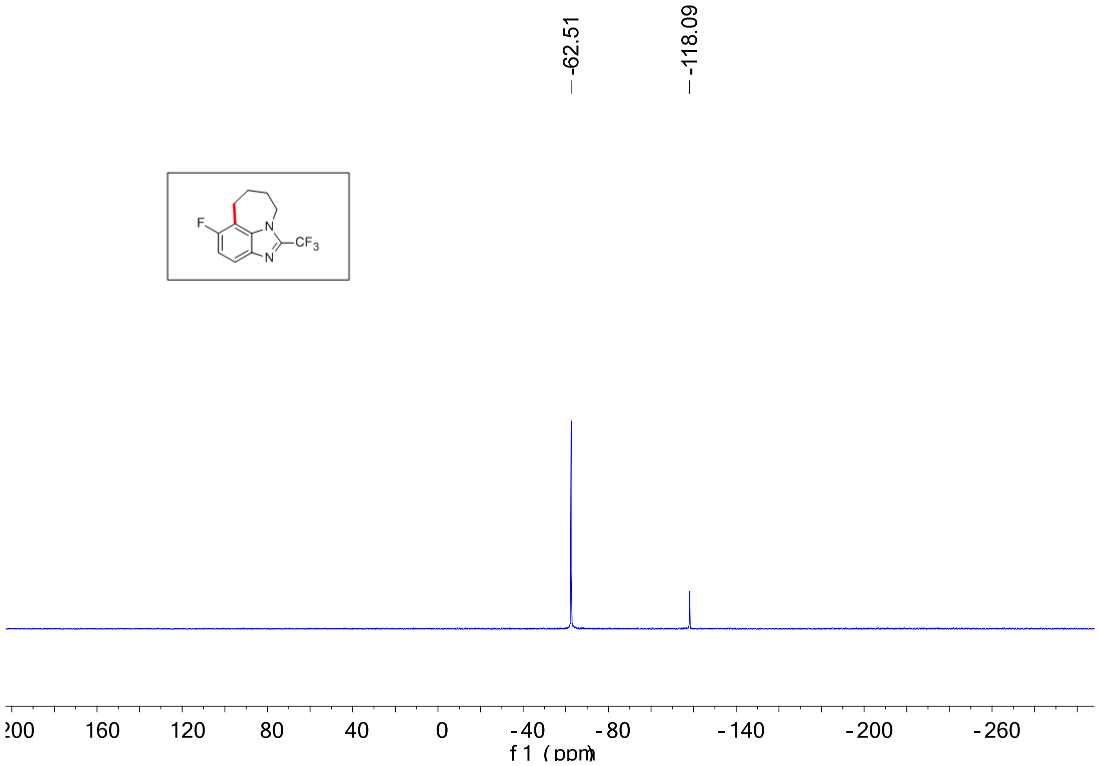

Supplementary Figure 90. ^{19}F NMR spectrum of compound **2g** in $CDCl_3$.

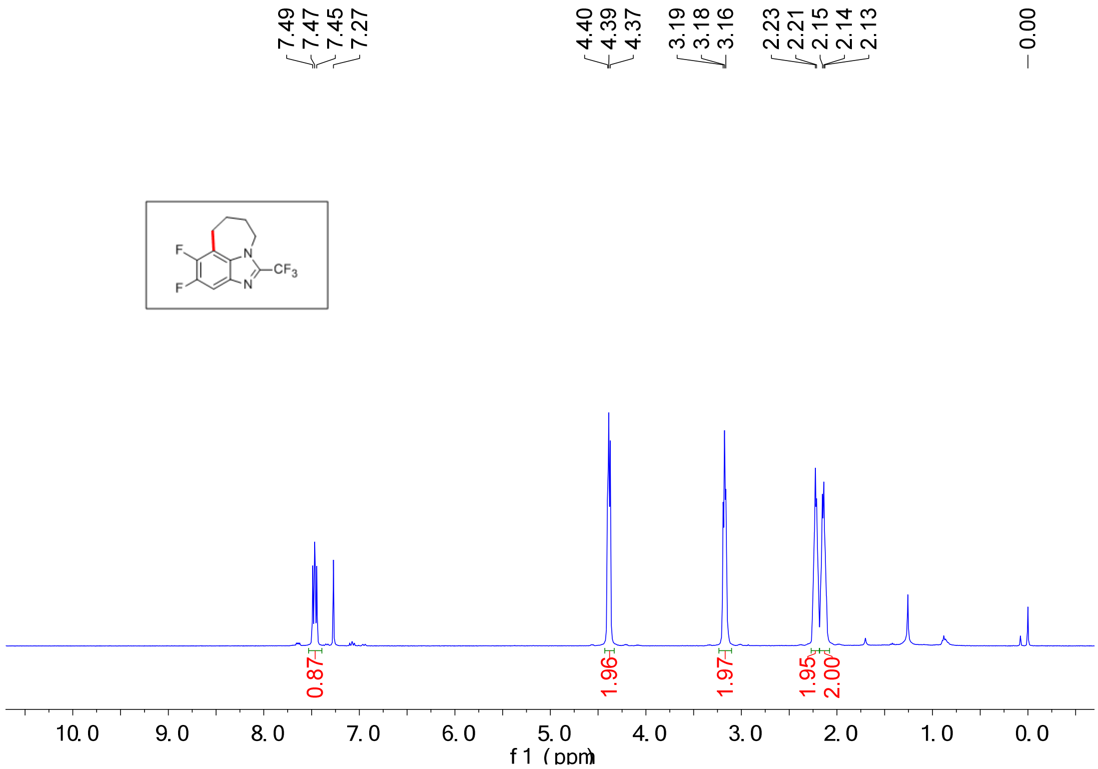

Supplementary Figure 91. ^{1}H NMR spectrum of compound **2h** in $CDCl_3$.

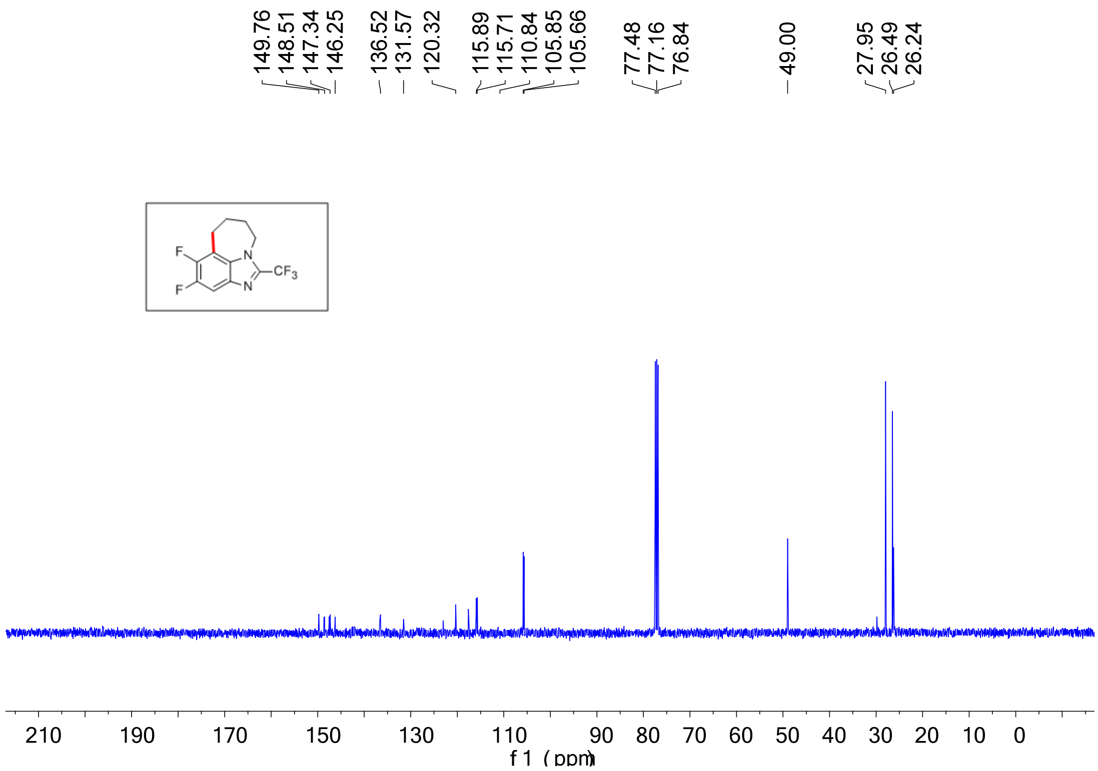

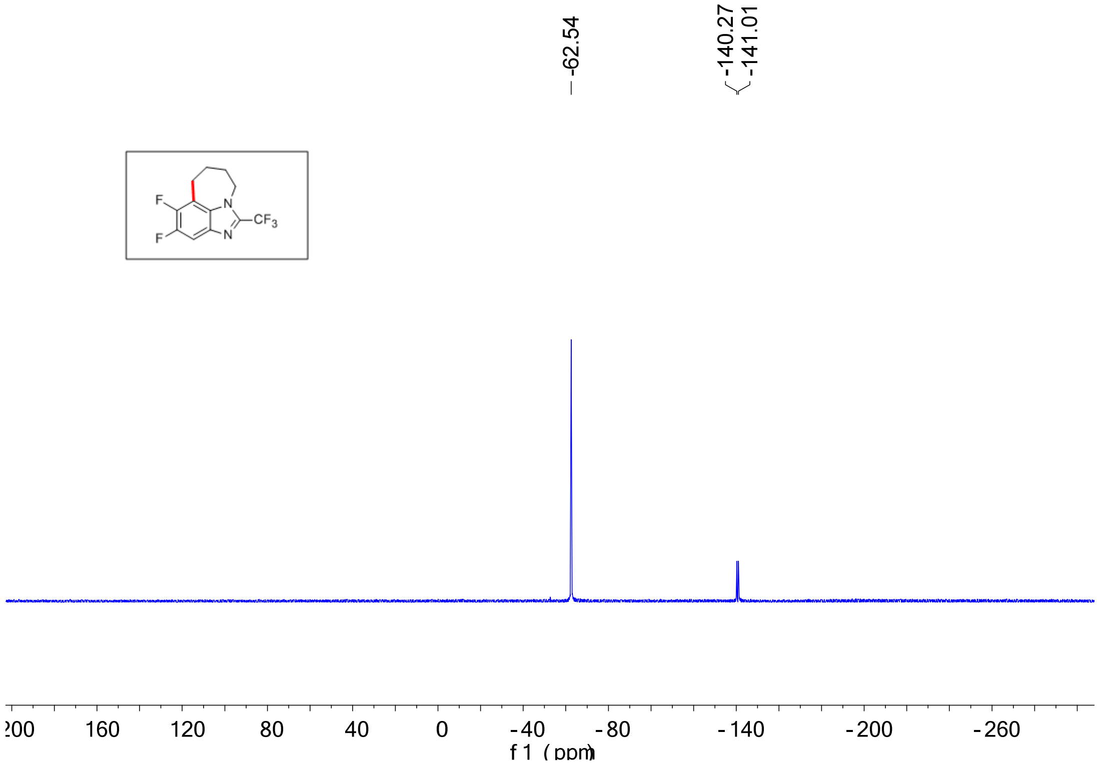

Supplementary Figure 92. ^{13}C and ^{19}F NMR spectra of compound **2h** in $CDCl_3$

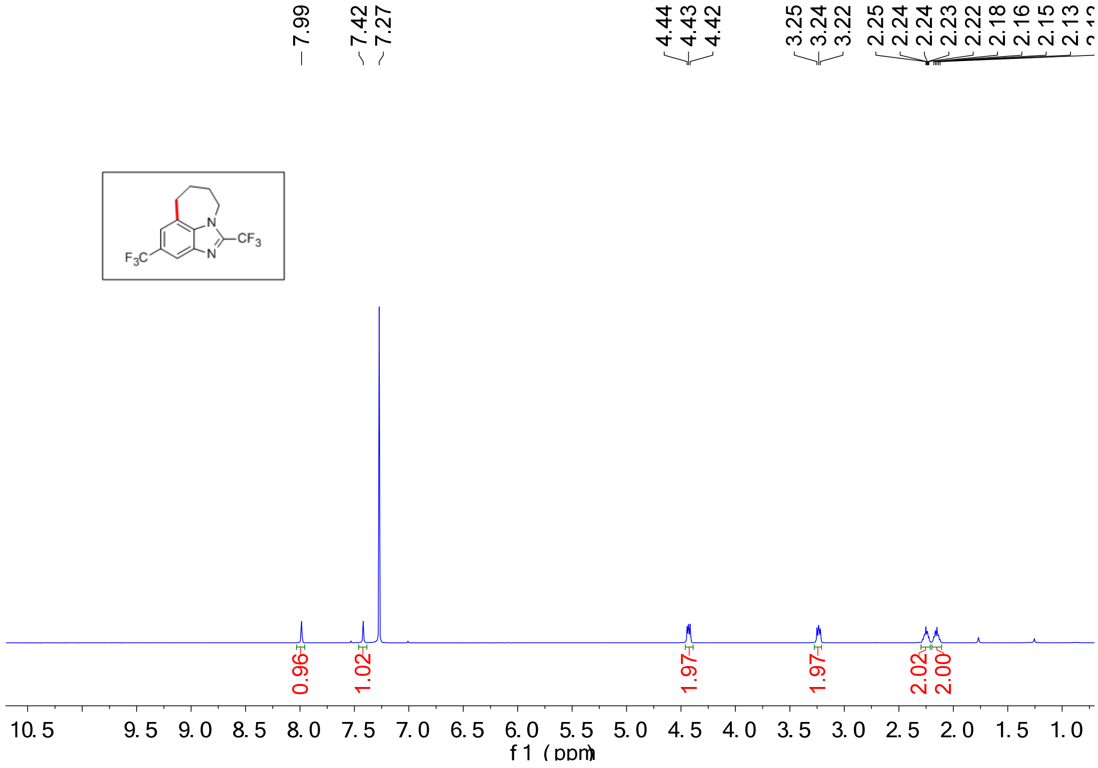

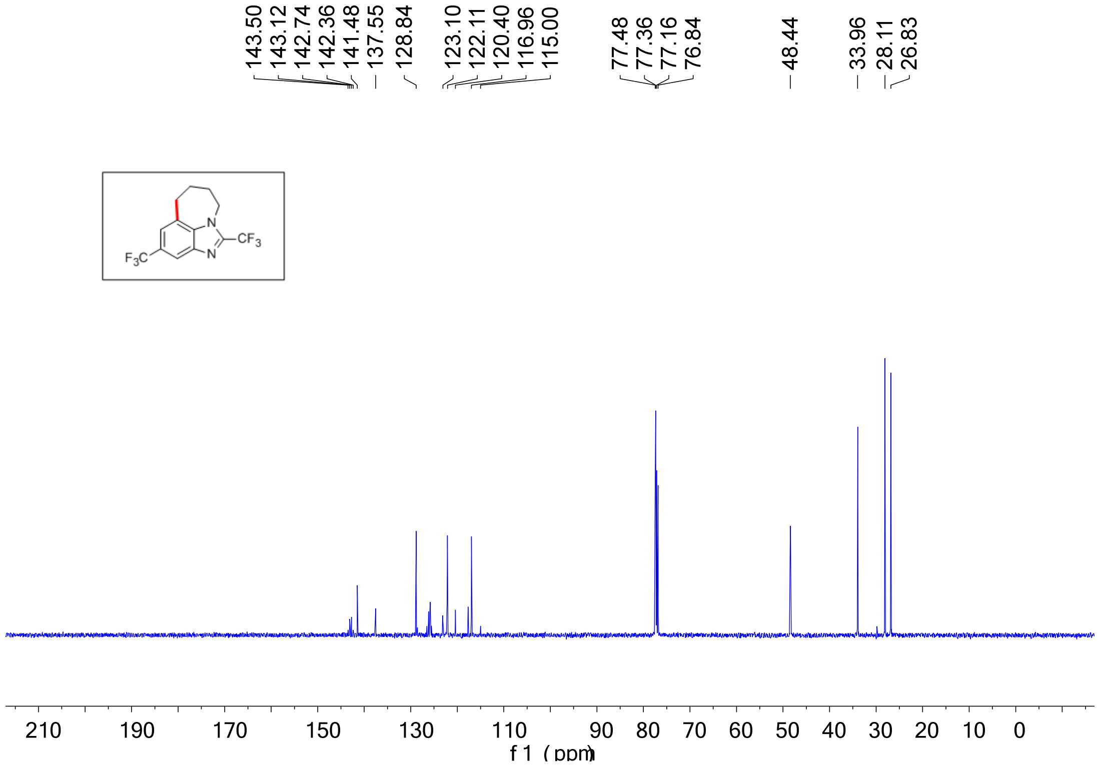

Supplementary Figure 93. ^{1}H and ^{13}C NMR spectra of compound **2i** in $CDCl_3$.

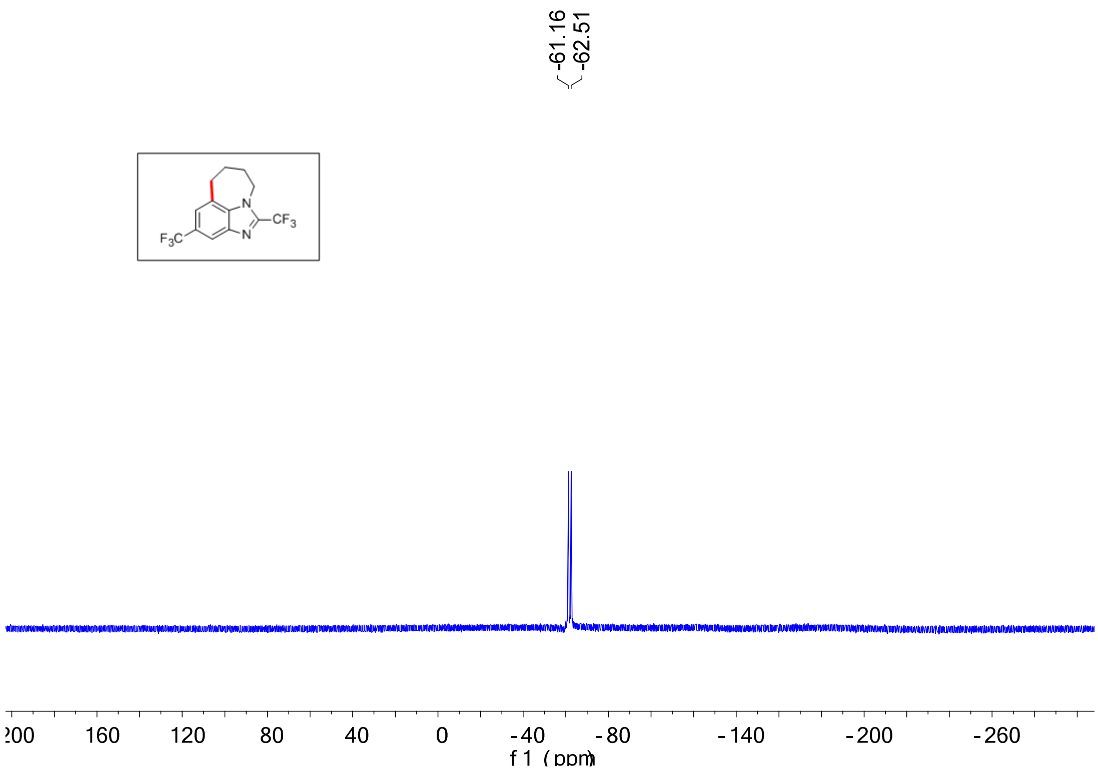

Supplementary Figure 94. ^{19}F NMR spectrum of compound **2i** in $CDCl_3$.

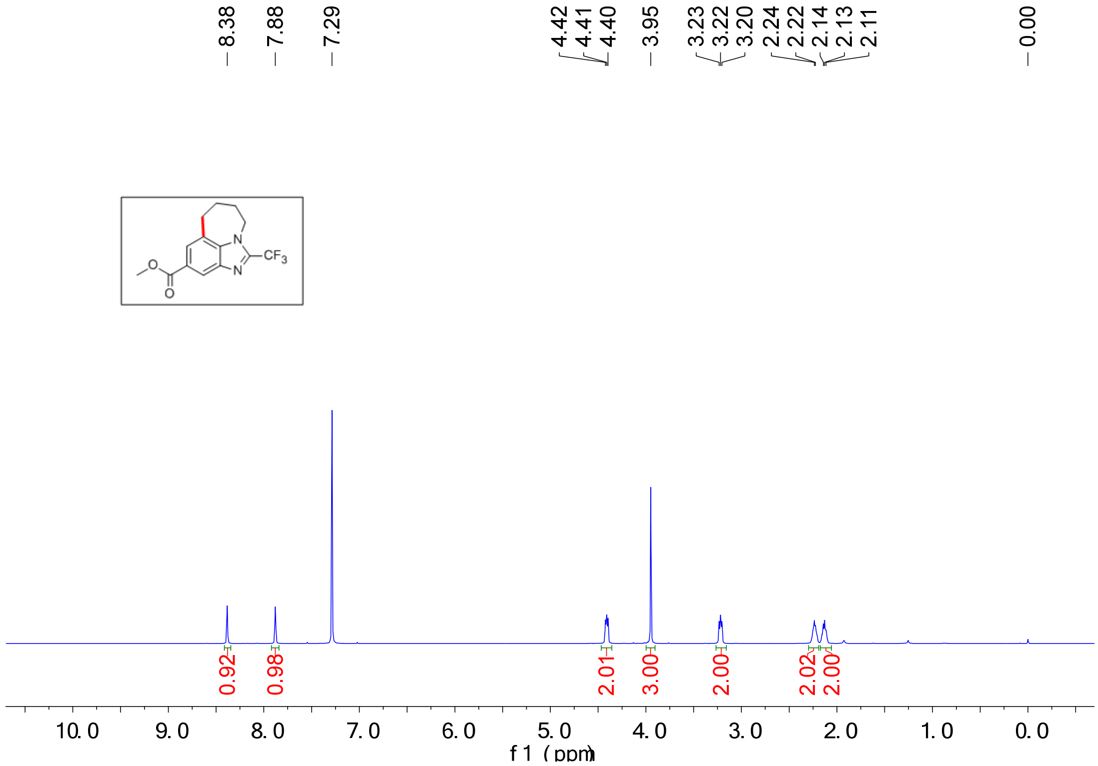

Supplementary Figure 95. ^{1}H NMR spectrum of compound **2j** in $CDCl_3$.

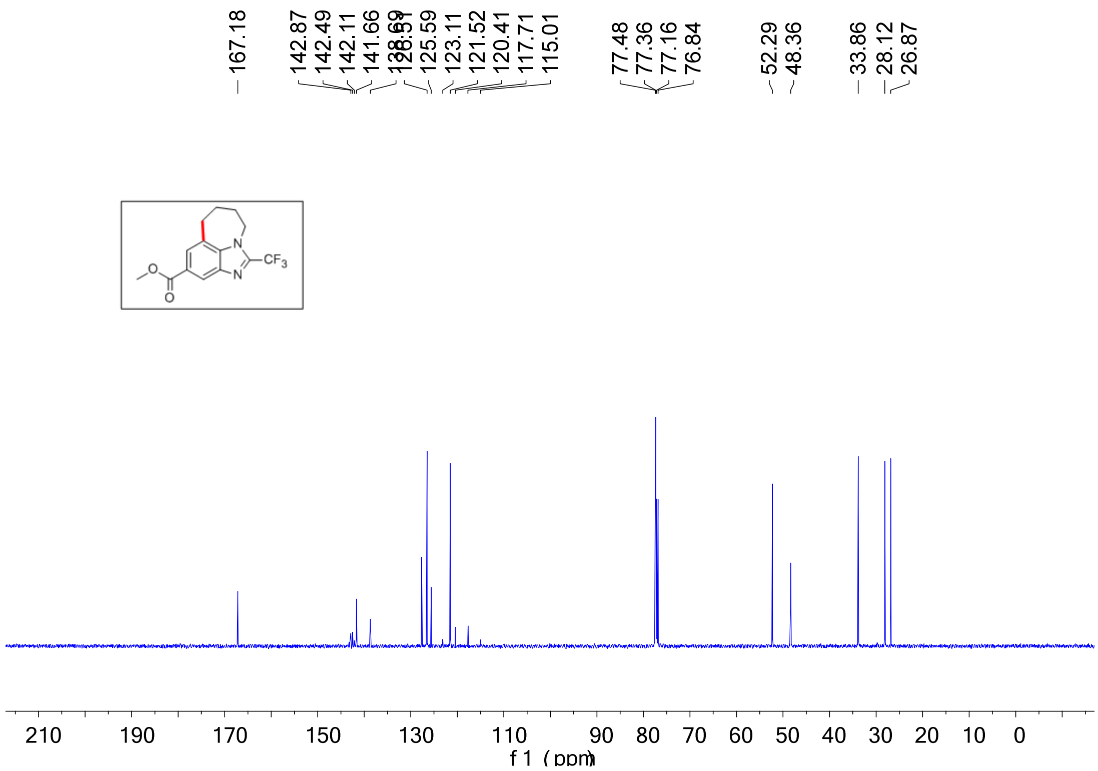

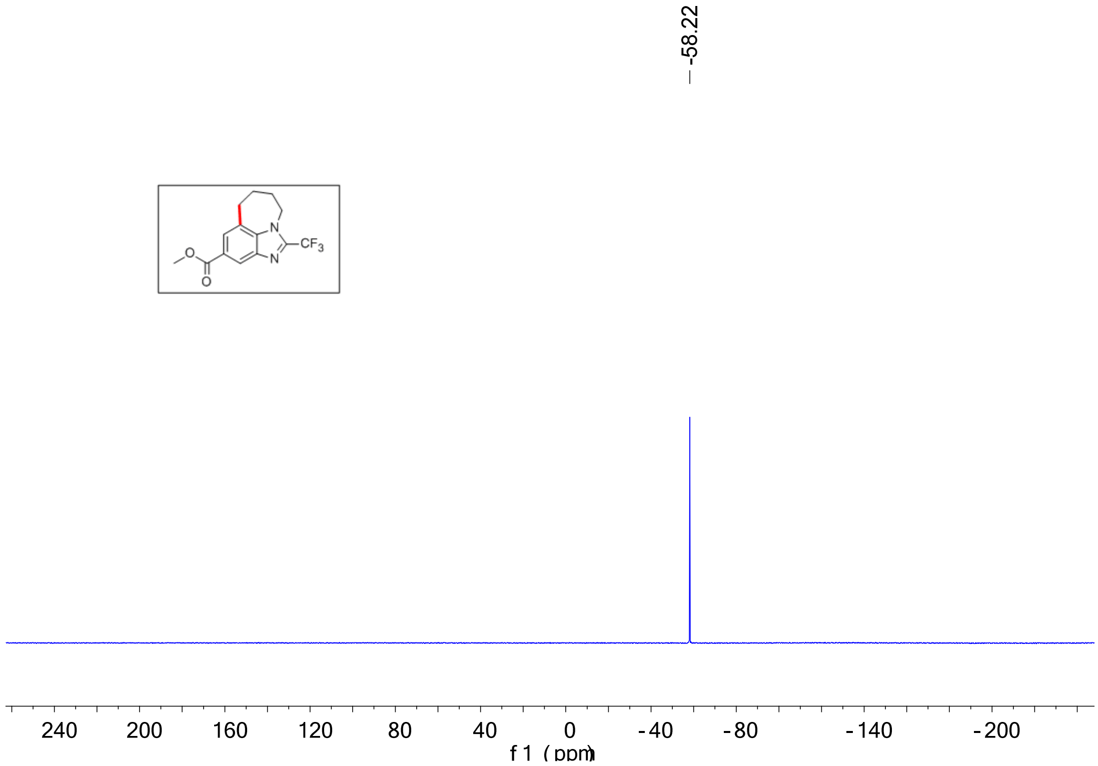

Supplementary Figure 96. ^{13}C and ^{19}F NMR spectra of compound **2j** in $CDCl_3$.

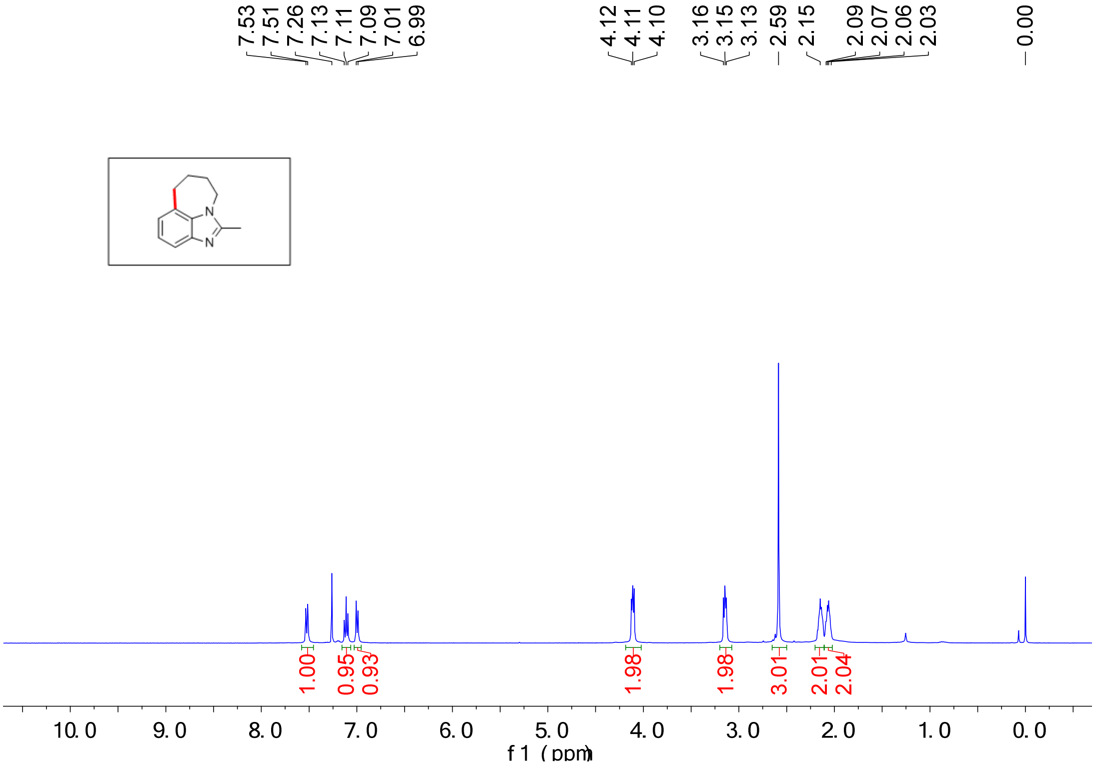

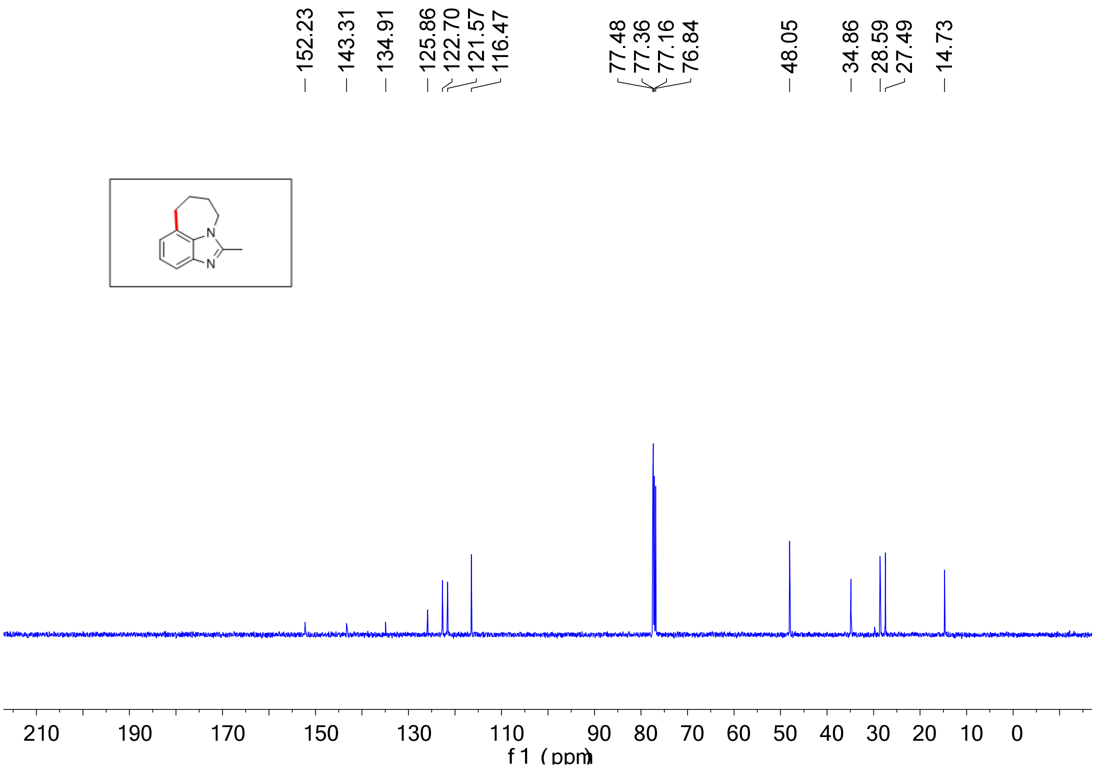

Supplementary Figure 97. ^{1}H and ^{13}C NMR spectra of compound **2k** in $CDCl_3$.

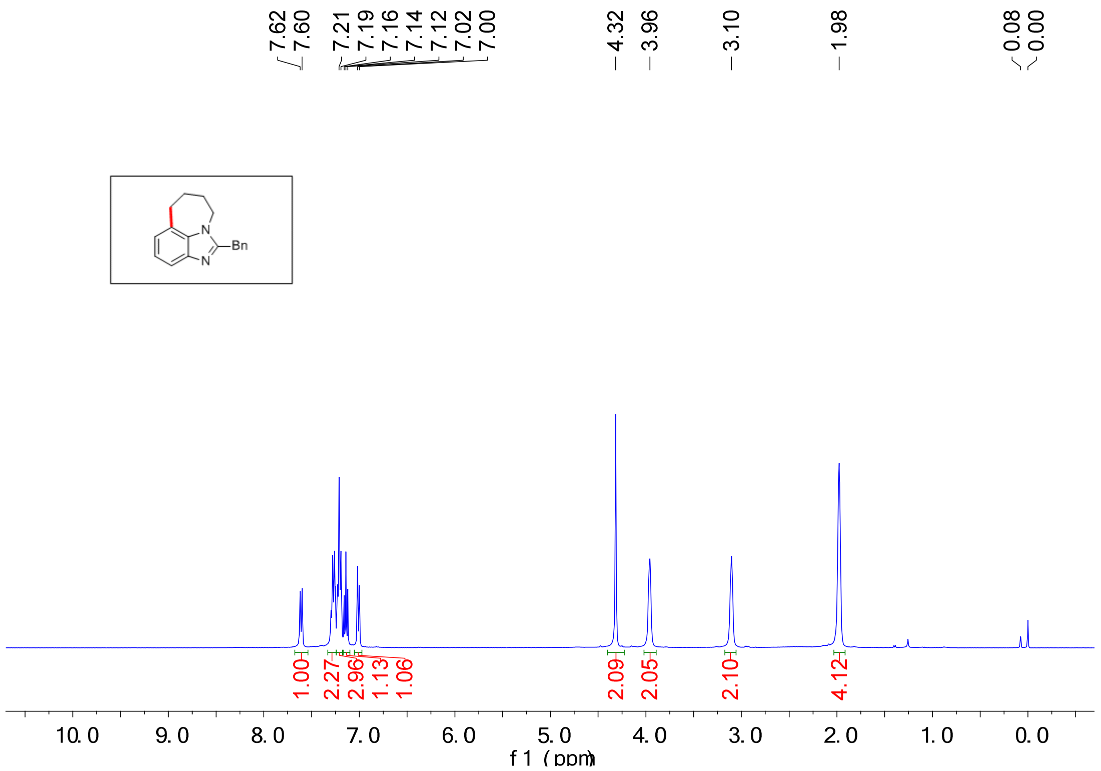

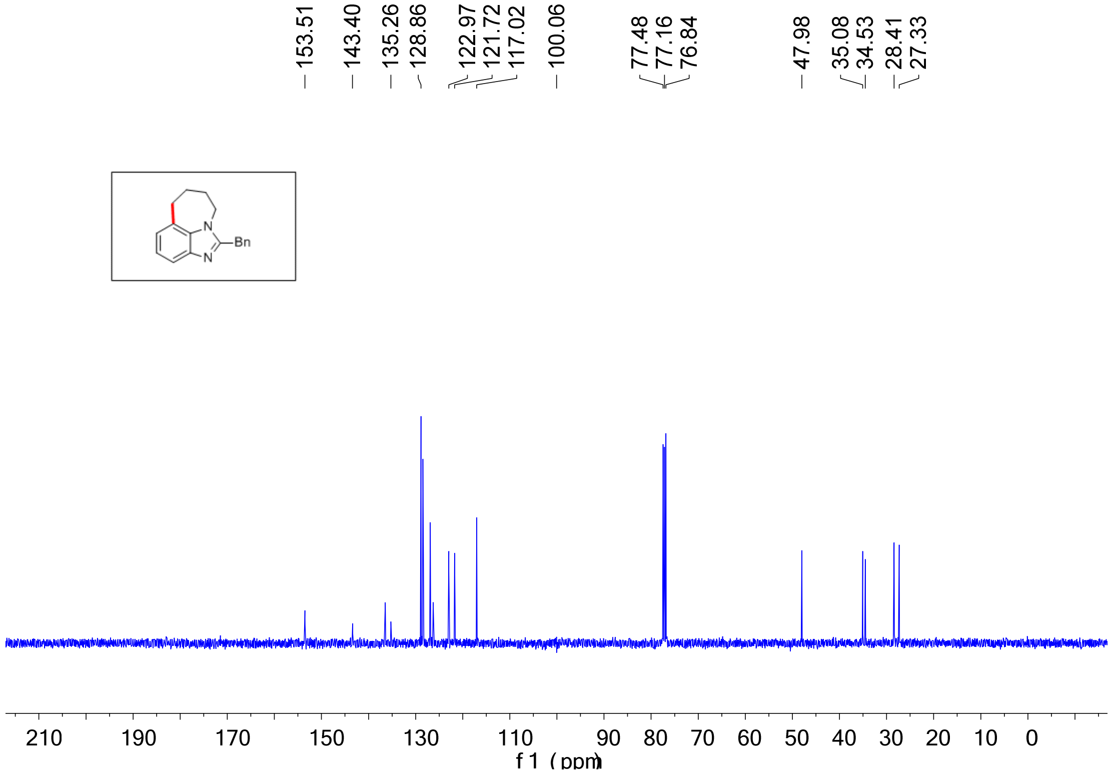

Supplementary Figure 98. ^{1}H and ^{13}C NMR spectra of compound **2l** in $CDCl_3$.

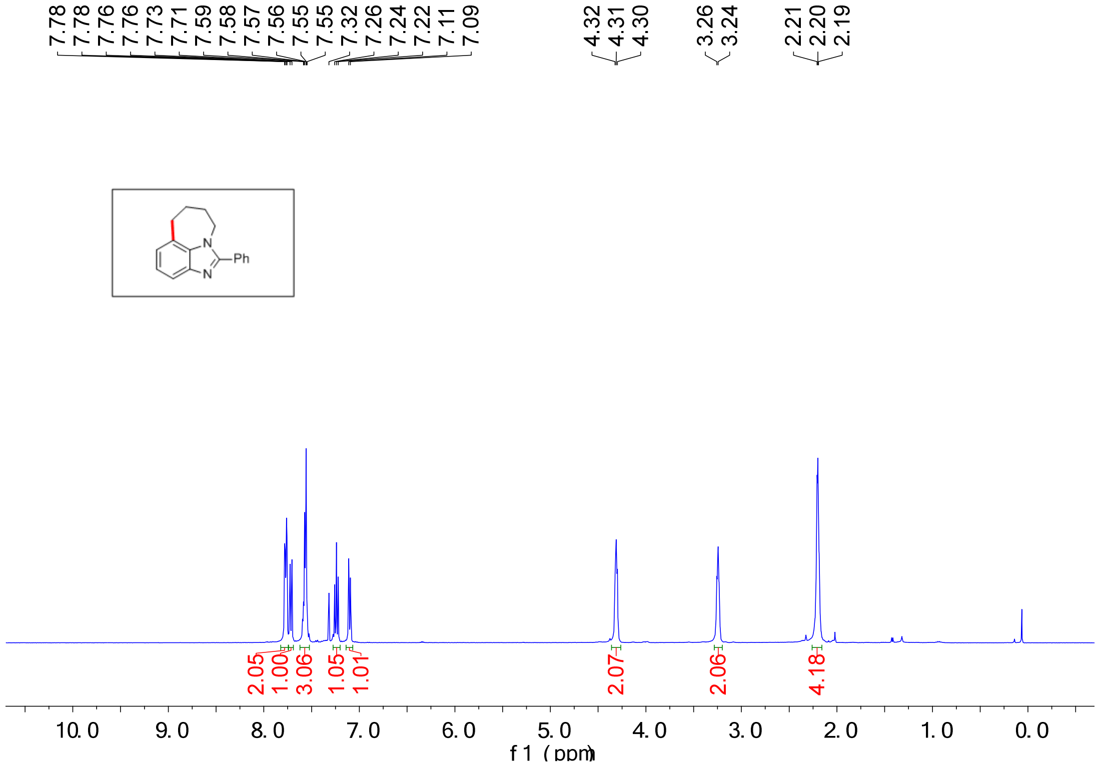

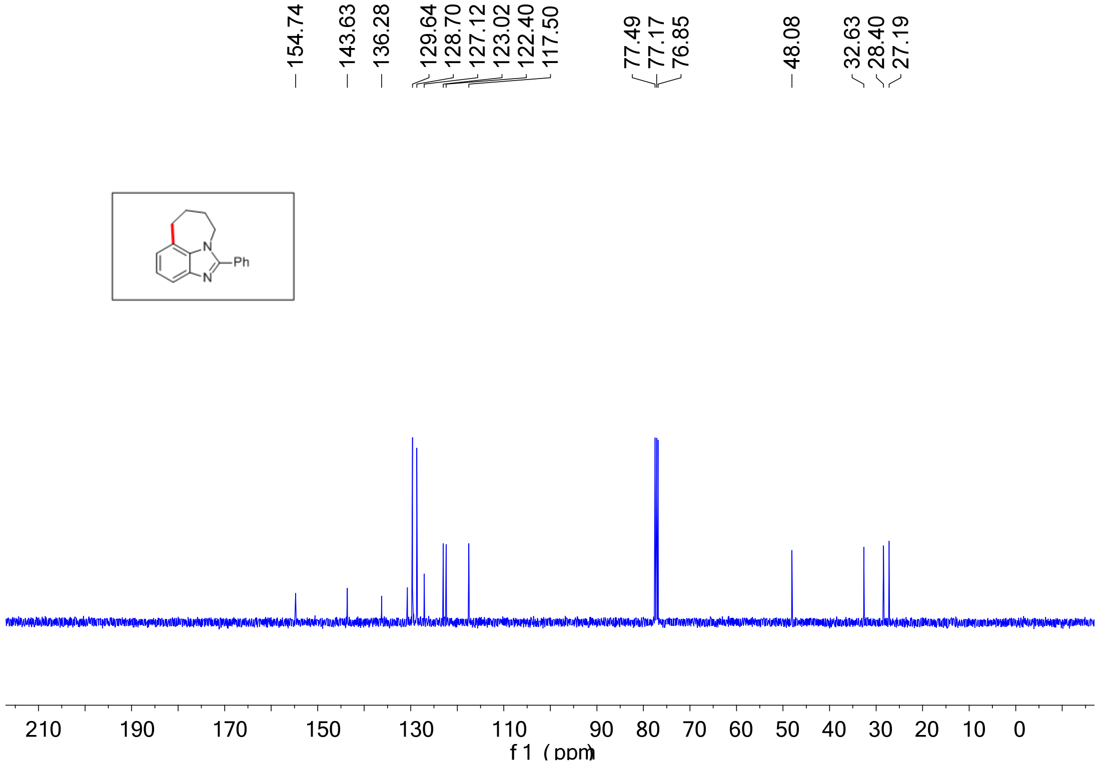

Supplementary Figure 99. ^{1}H and ^{13}C NMR spectra of compound **2m** in $CDCl_3$

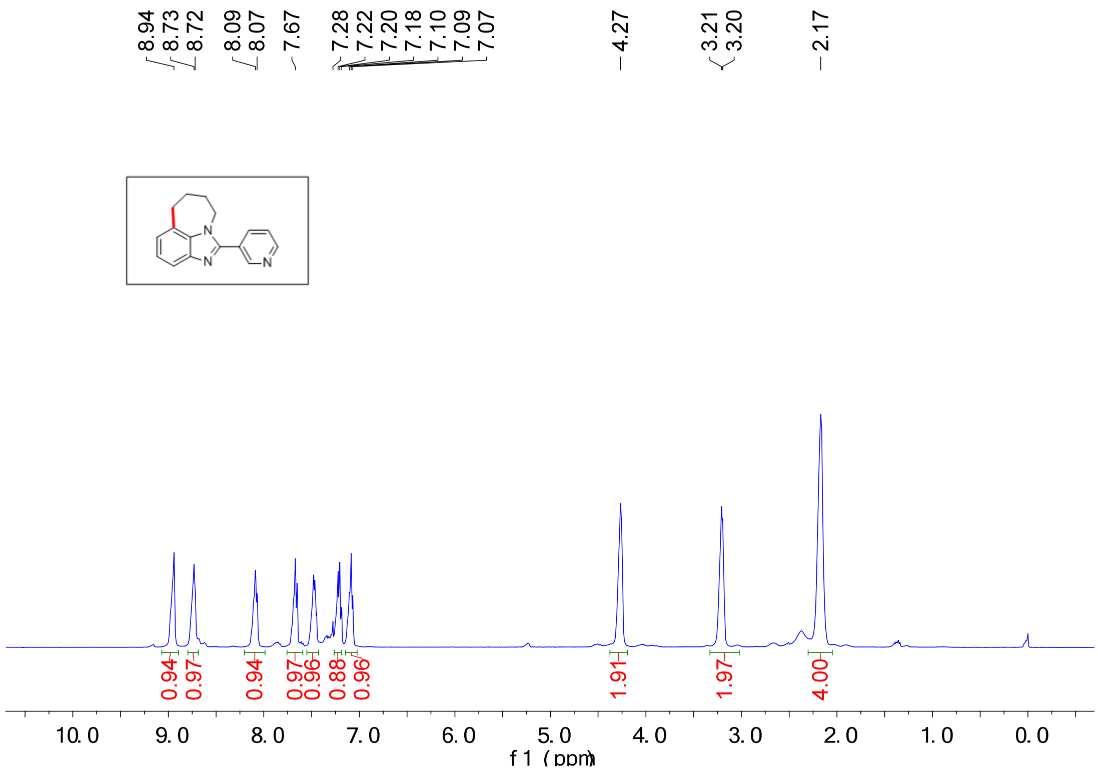

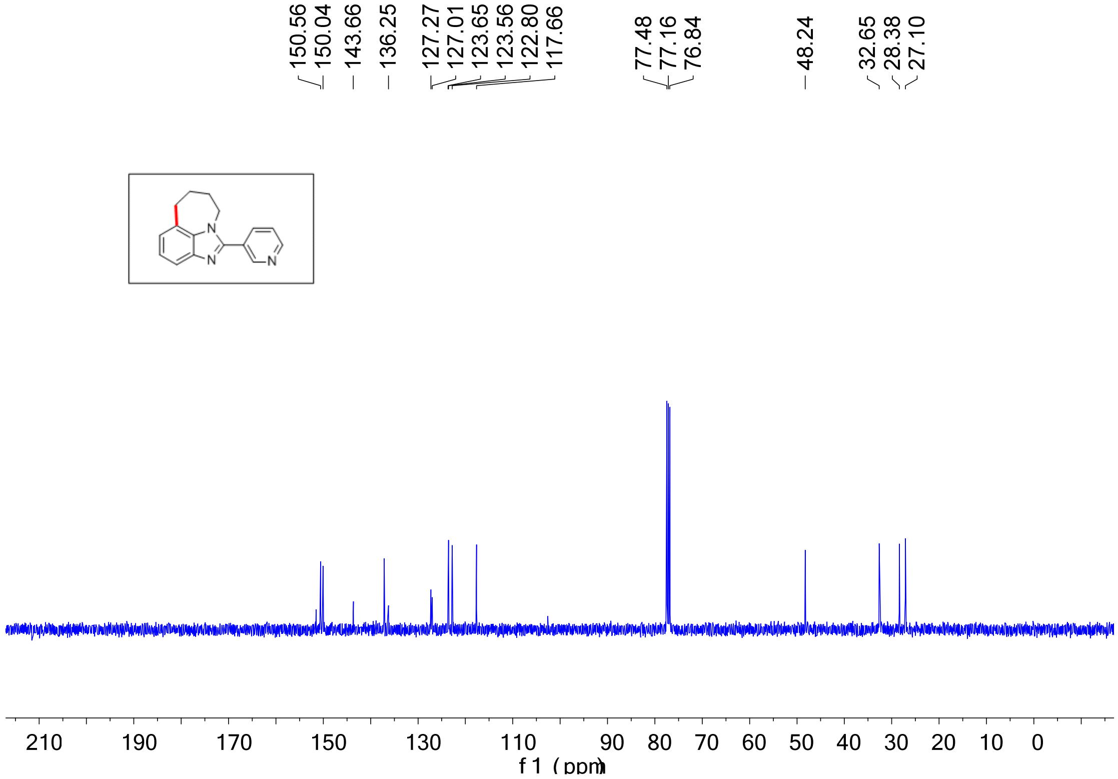

Supplementary Figure 100. ^{1}H and ^{13}C NMR spectra of compound **2n** in $CDCl_3$.

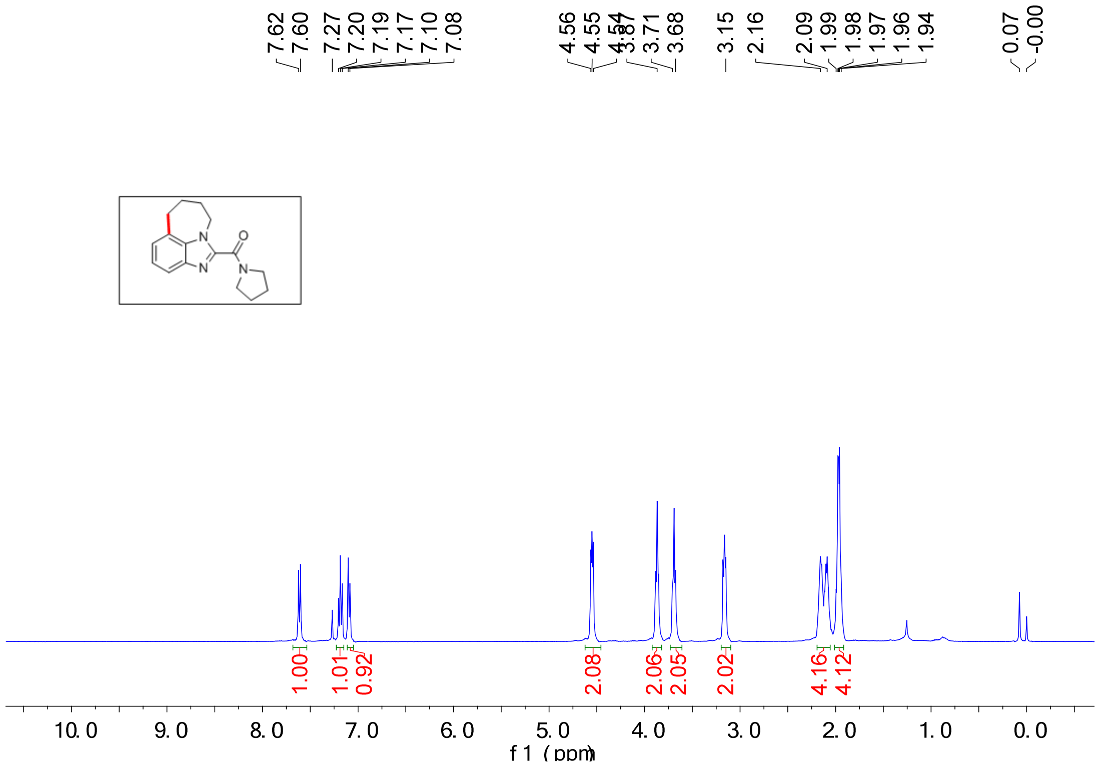

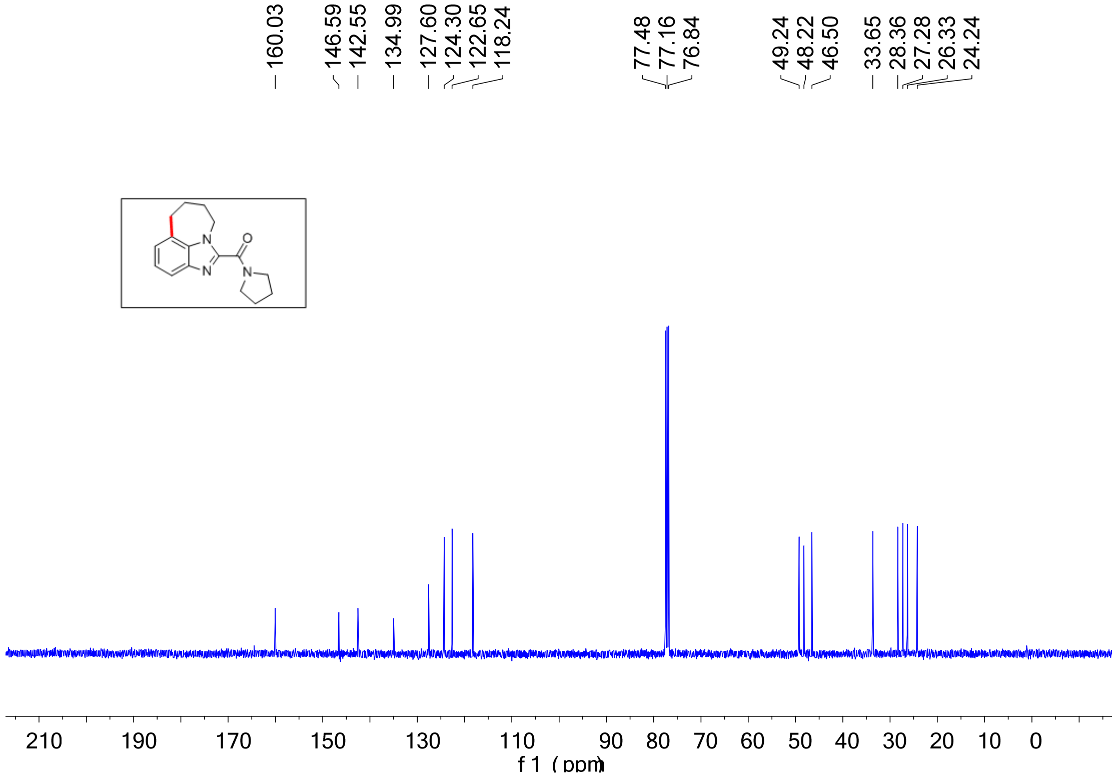

Supplementary Figure 101. ^{1}H and ^{13}C NMR spectra of compound **2o** in $CDCl_3$.

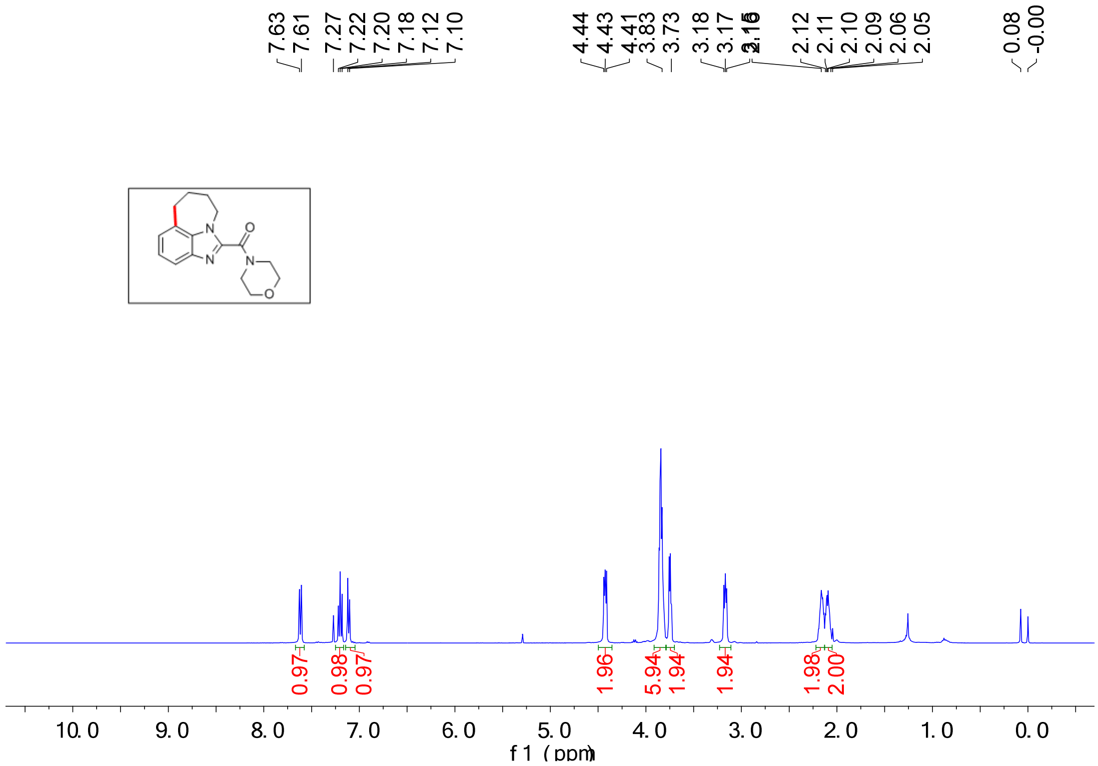

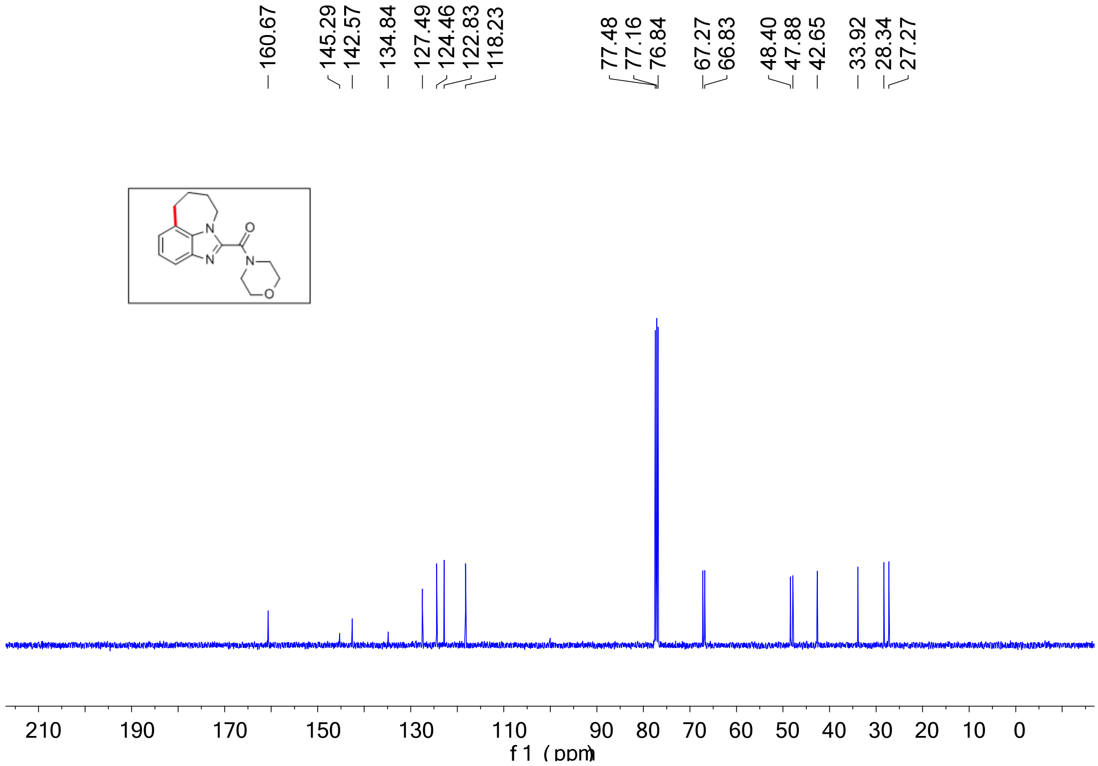

Supplementary Figure 102. ^{1}H and ^{13}C NMR spectra of compound **2p** in $CDCl_3$.

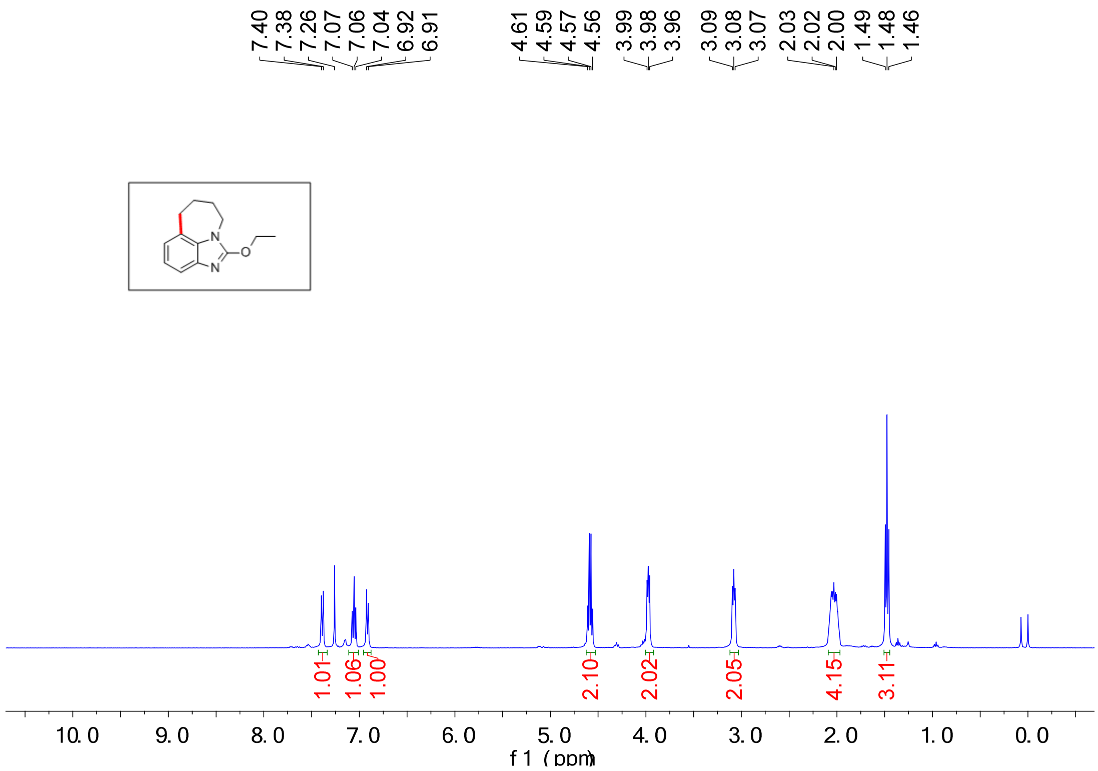

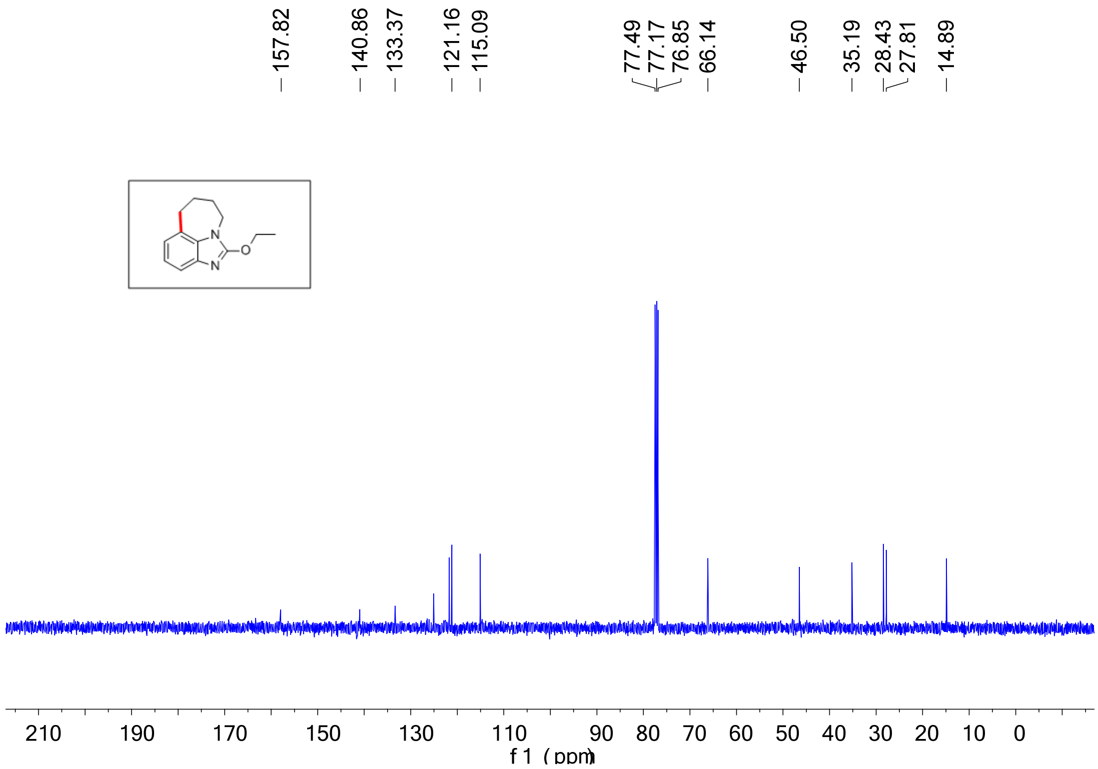

Supplementary Figure 103. ^{1}H and ^{13}C NMR spectra of compound **2q** in $CDCl_3$

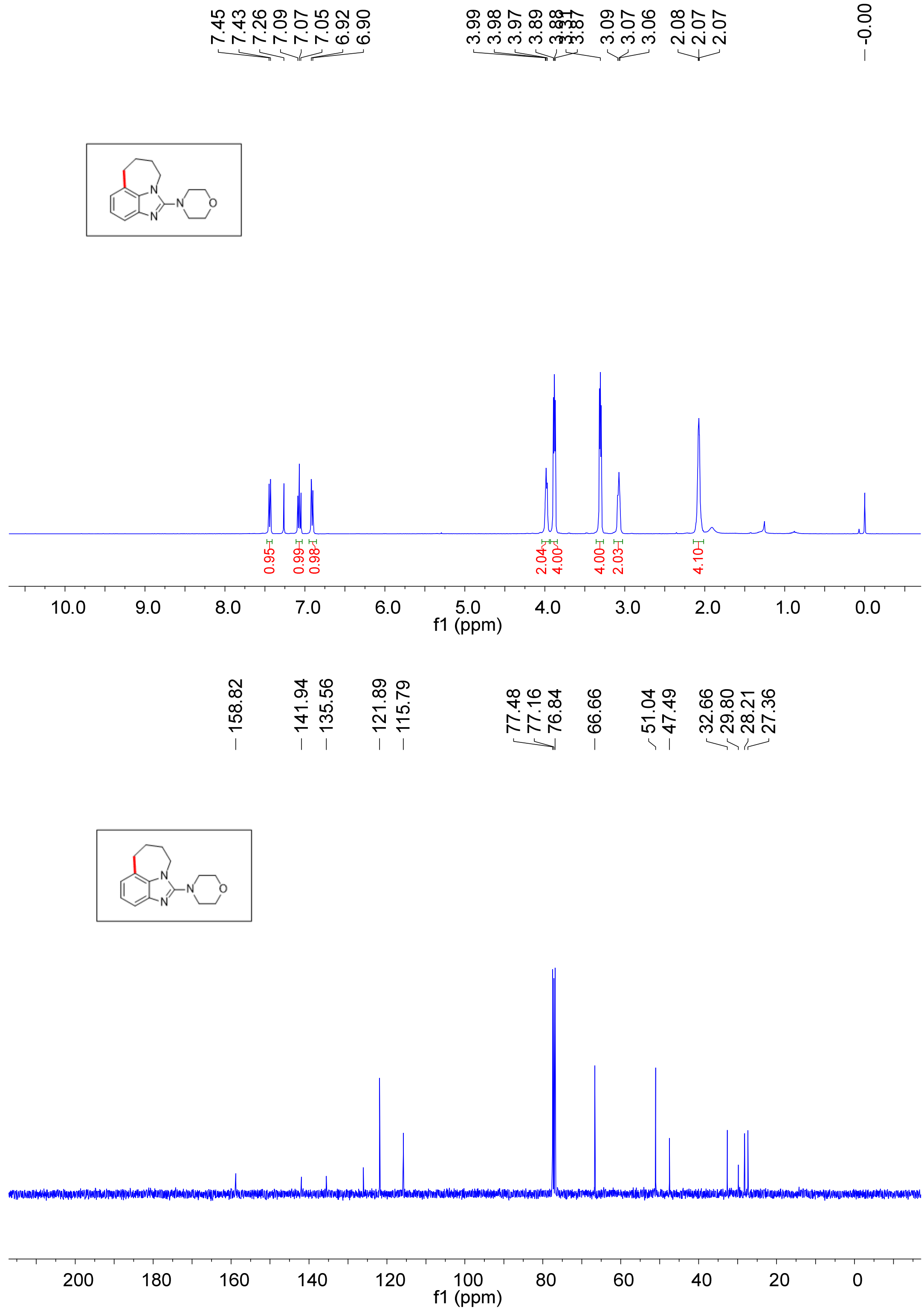

Supplementary Figure 104. ^{1}H and ^{13}C NMR spectra of compound **2r** in $CDCl_3$.

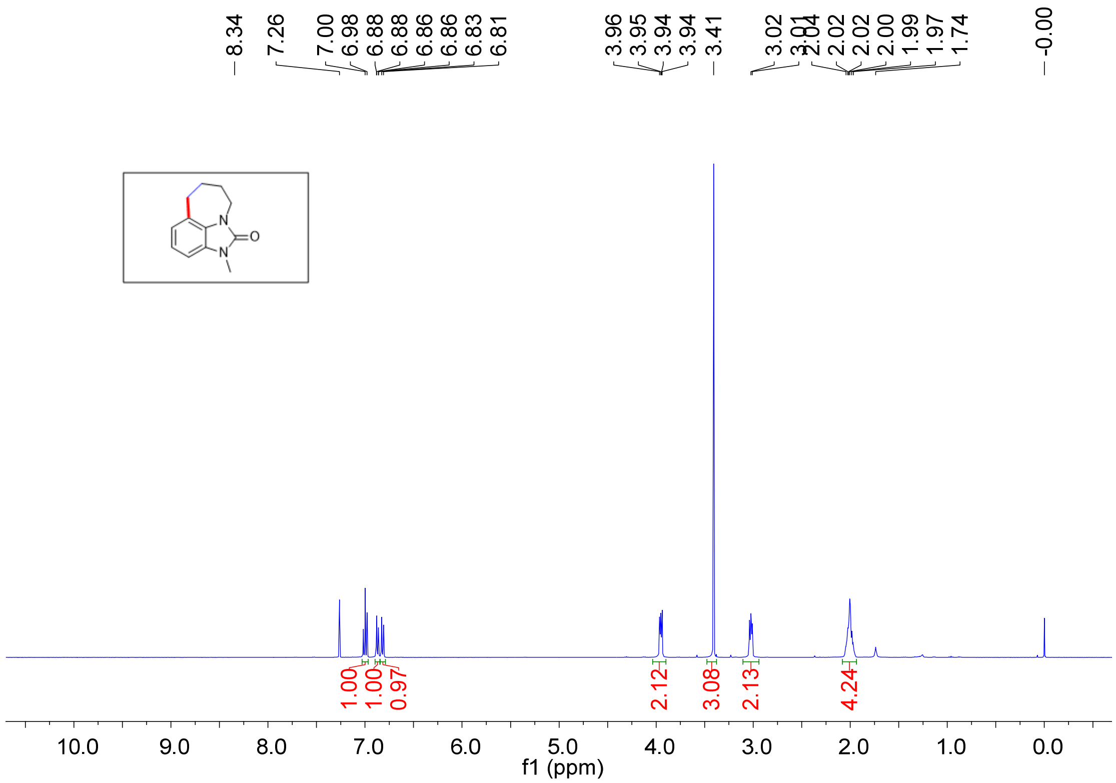

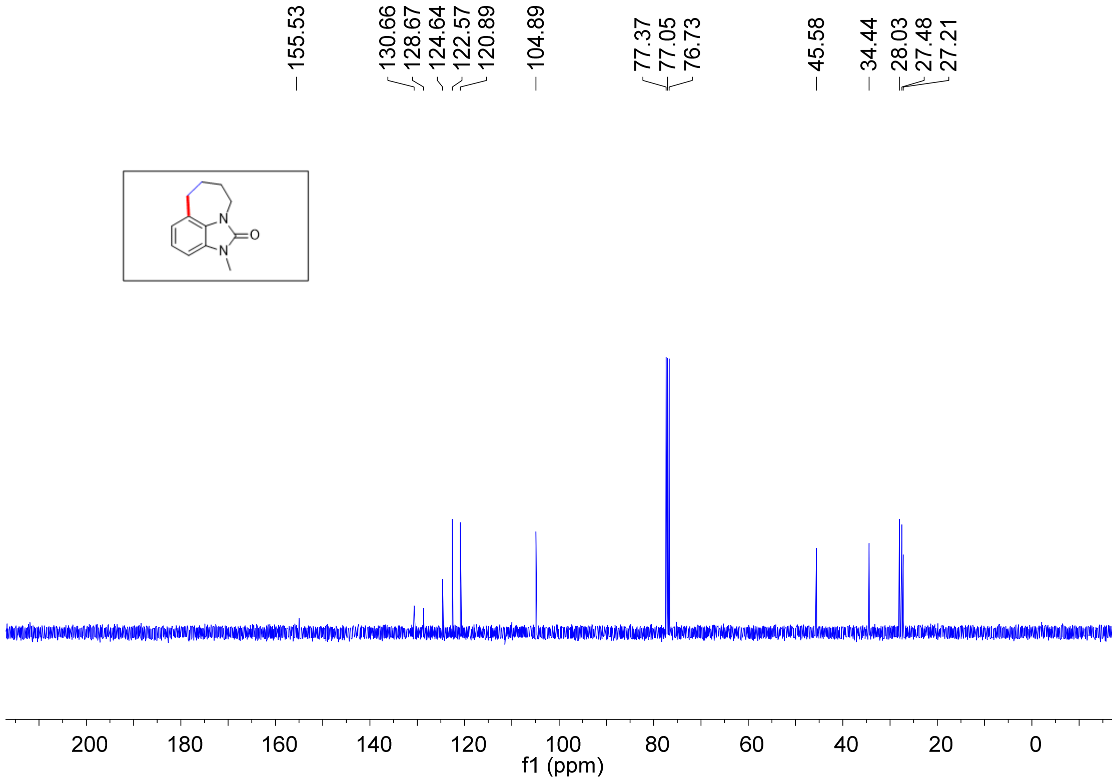

Supplementary Figure 105. ^{1}H and ^{13}C NMR spectra of compound **2s** in $CDCl_3$.

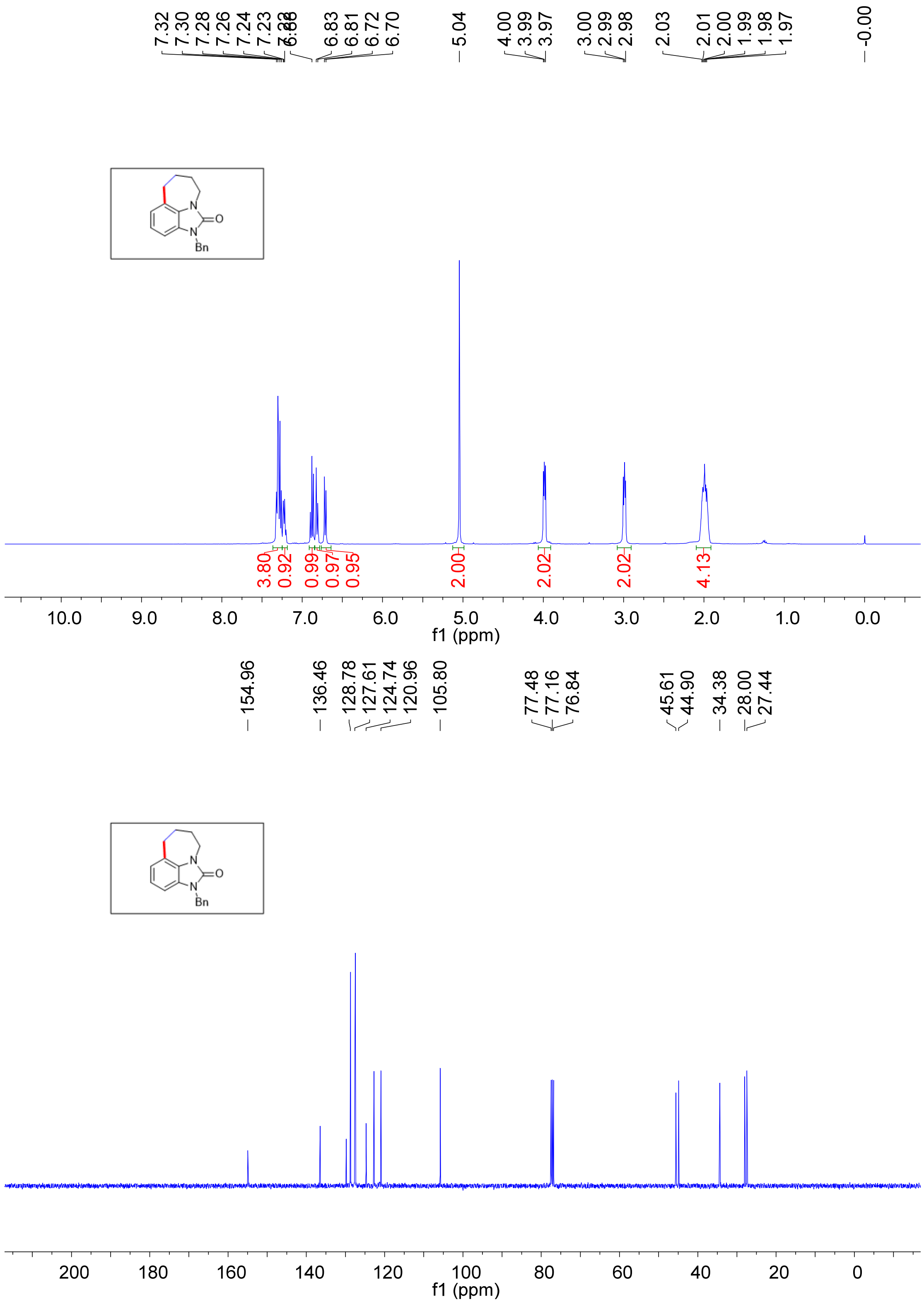

Supplementary Figure 106. ^{1}H and ^{13}C NMR spectra of compound **2t** in $CDCl_3$.

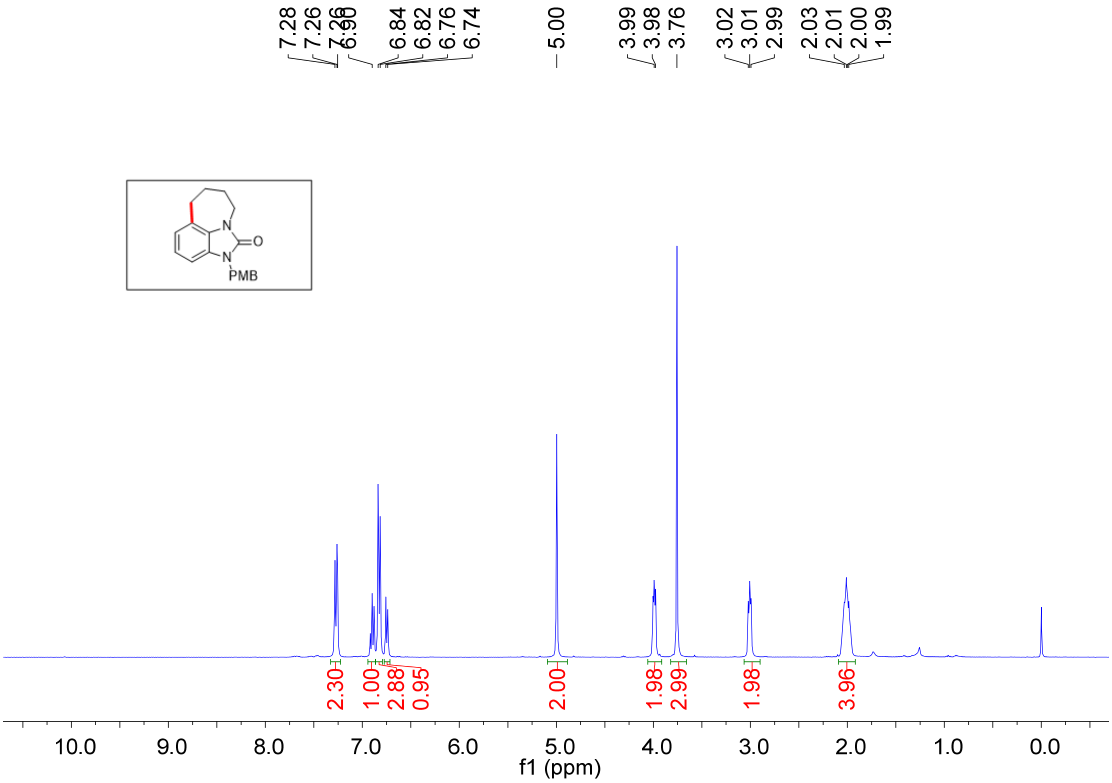

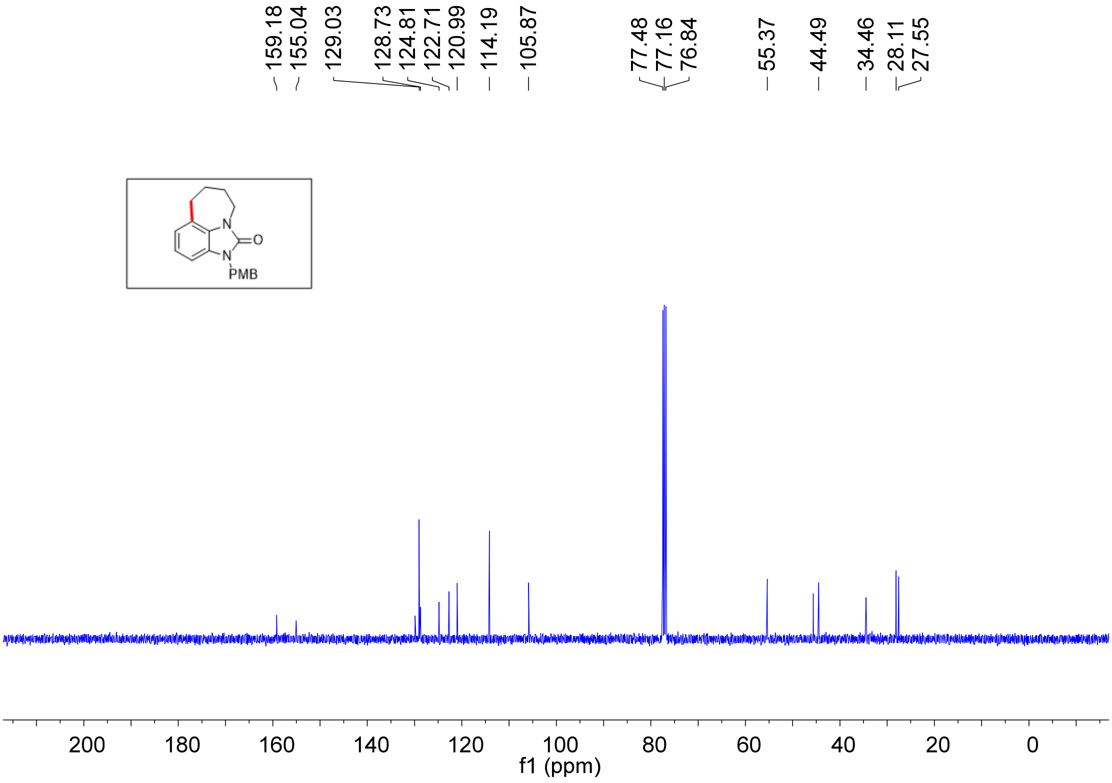

Supplementary Figure 107. ^1H and ^{13}C NMR spectra of compound **2u** in $CDCl_3$.

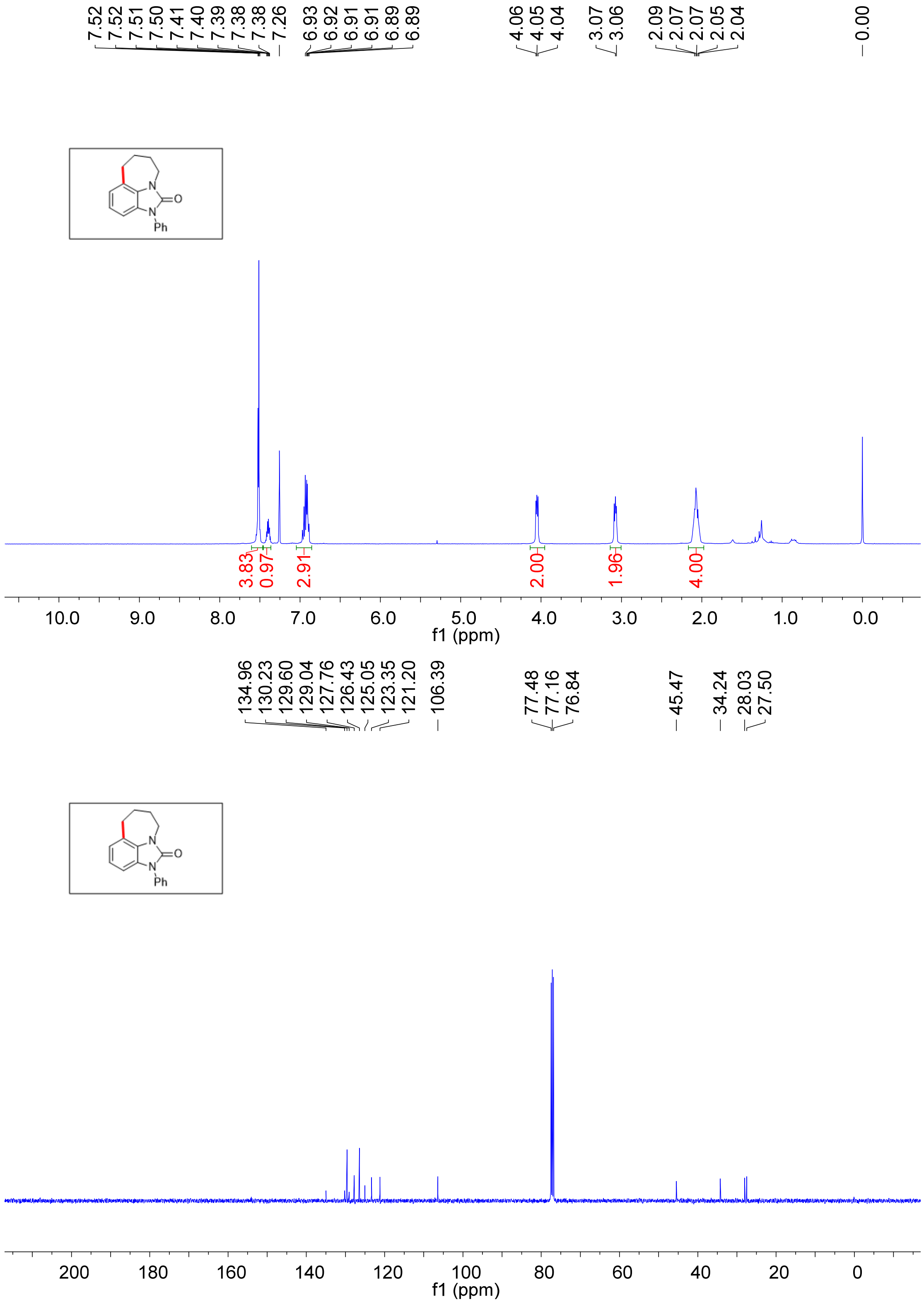

Supplementary Figure 108. ^{1}H and ^{13}C NMR spectra of compound **2v** in $CDCl_3$.

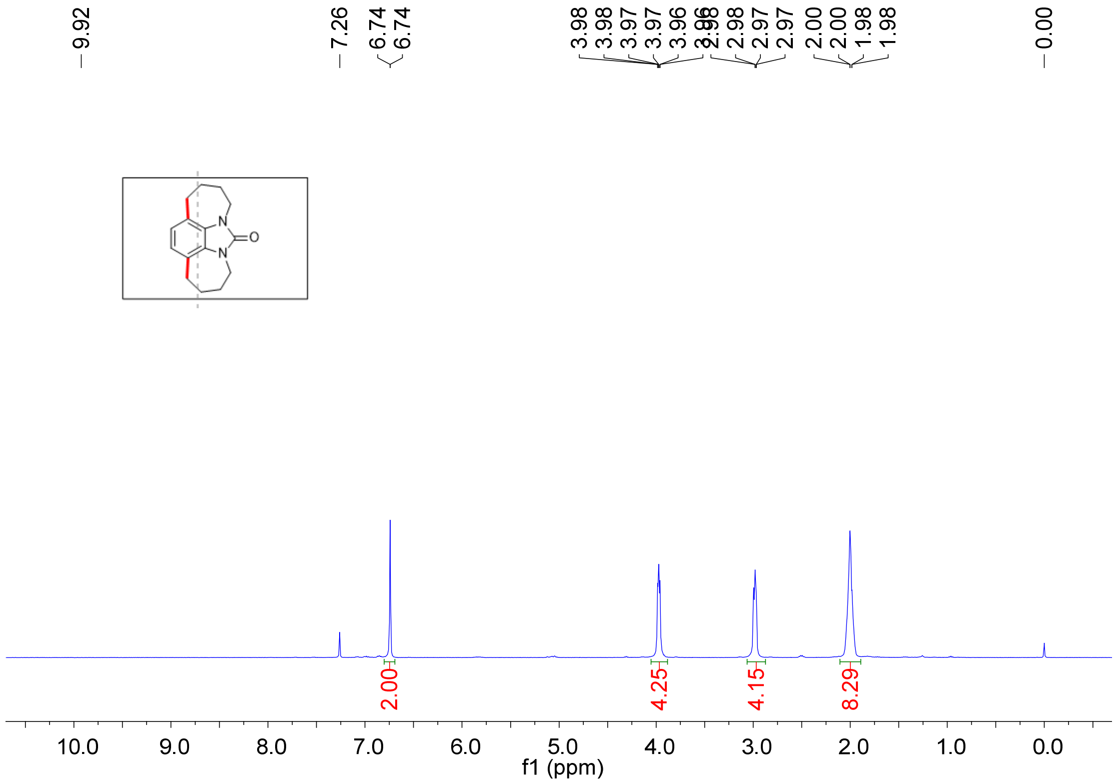

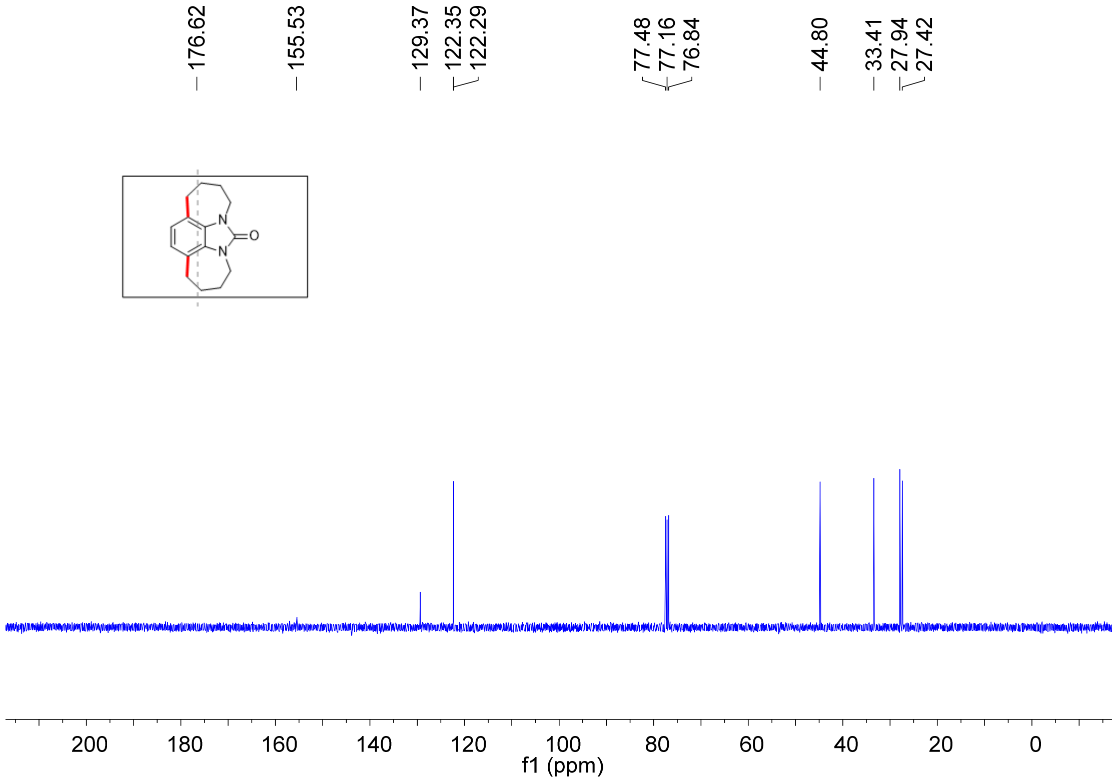

Supplementary Figure 109. ^{1}H and ^{13}C NMR spectra of compound **2w** in $CDCl_3$.

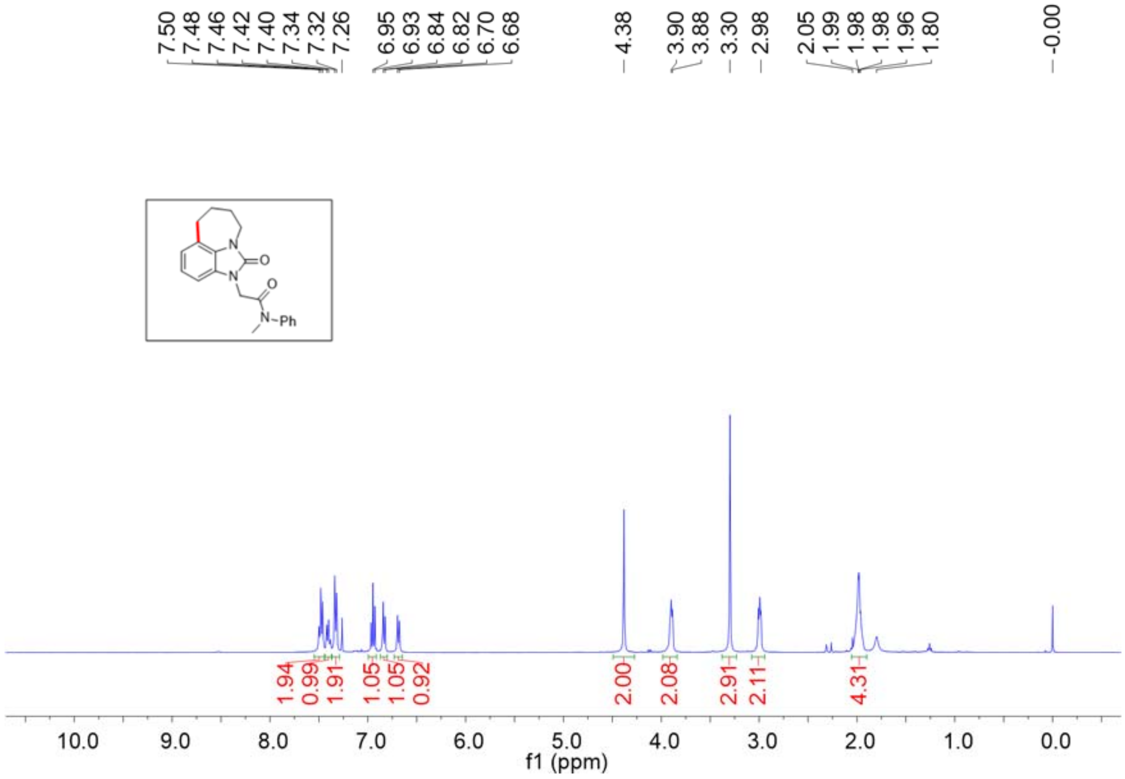

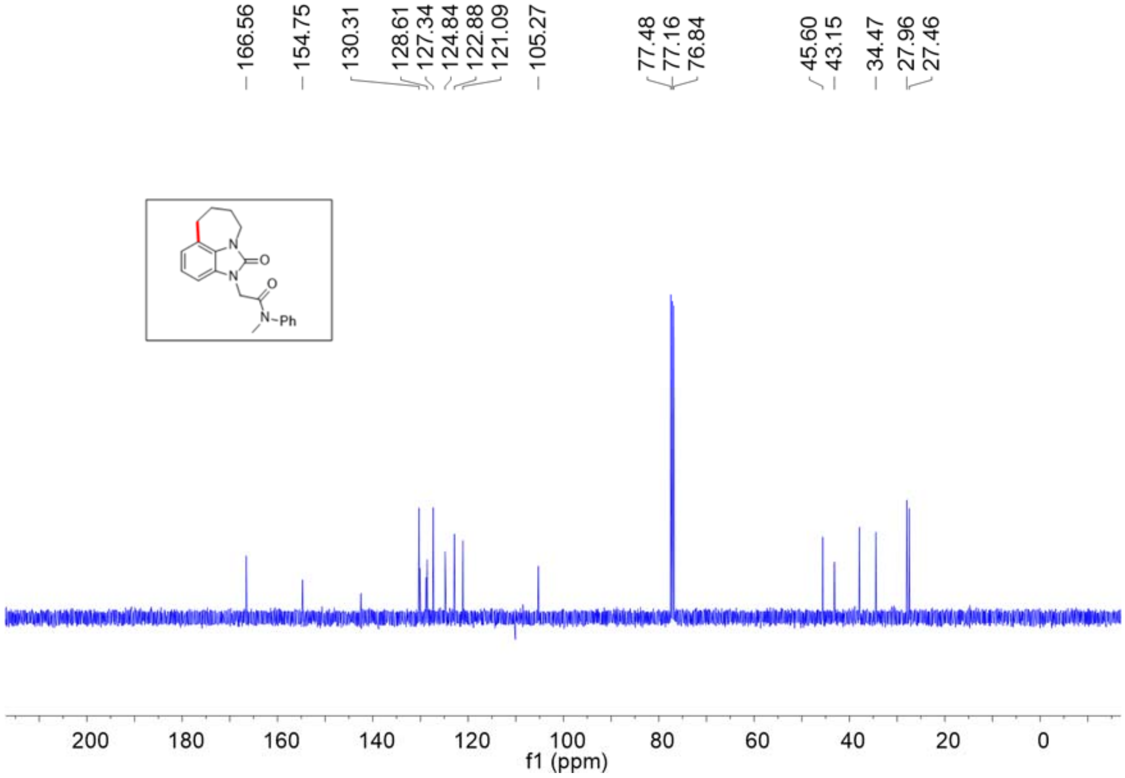

Supplementary Figure 110. ^{1}H and ^{13}C NMR spectra of compound **2x** in $CDCl_3$.

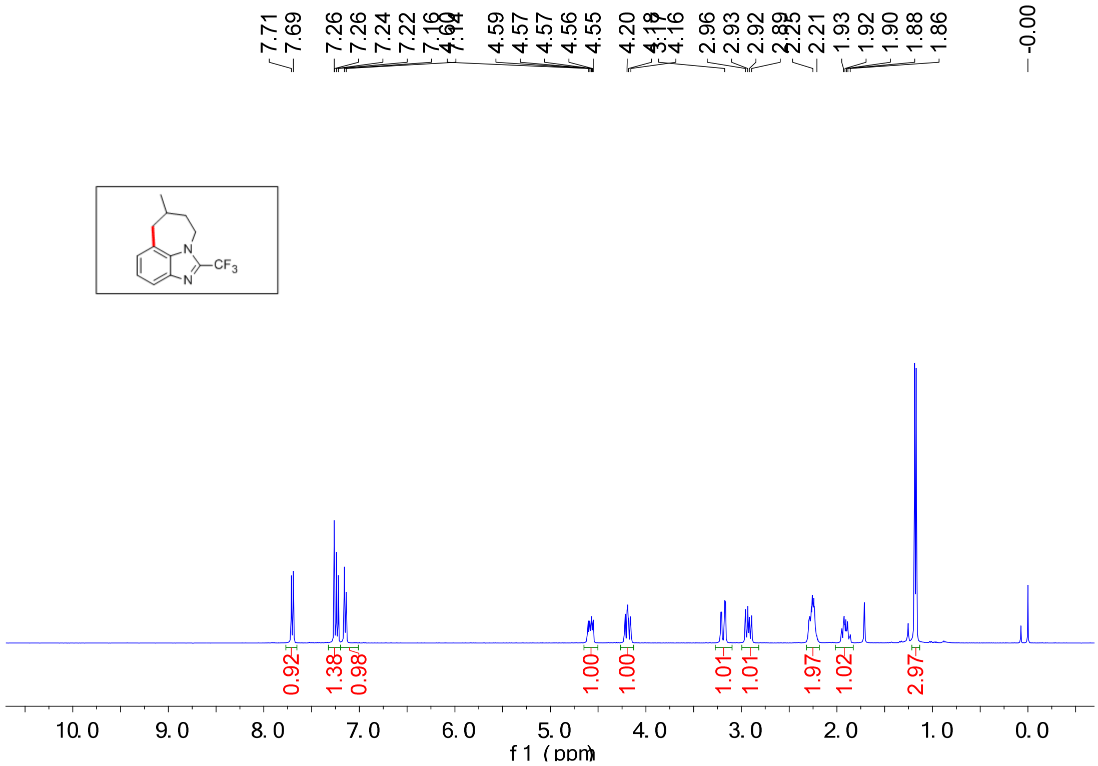

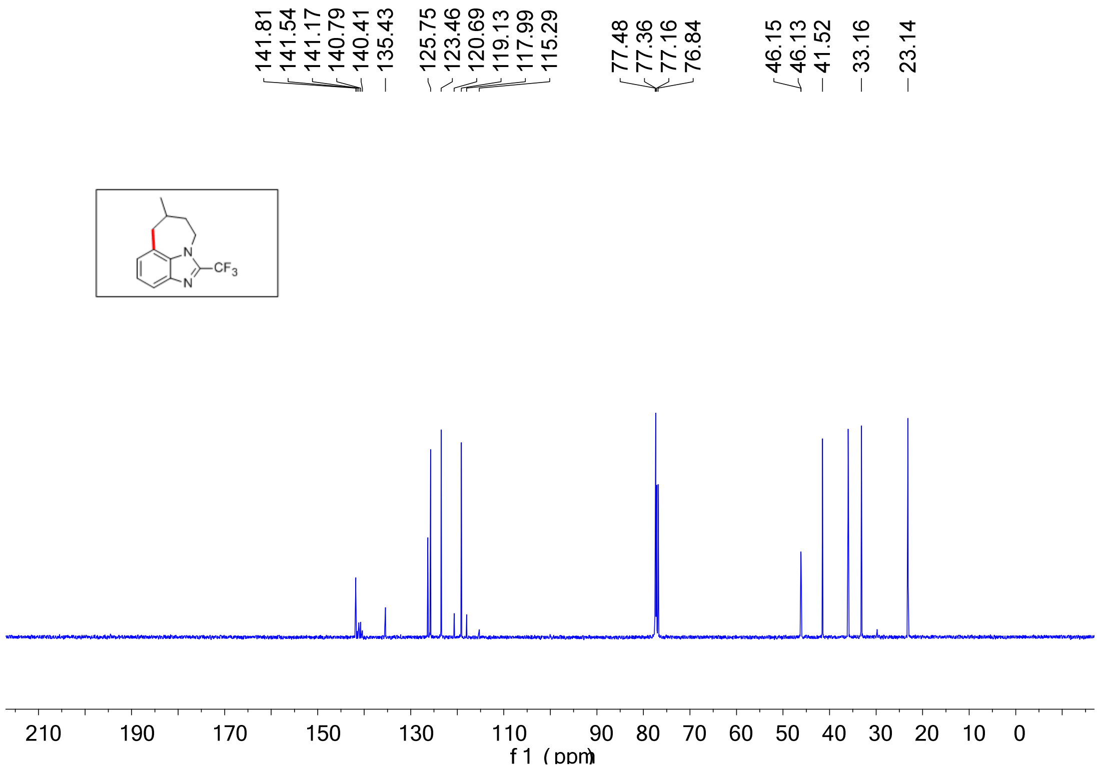

Supplementary Figure 111. ^{1}H and ^{13}C NMR spectra of compound **3a** in $CDCl_3$.

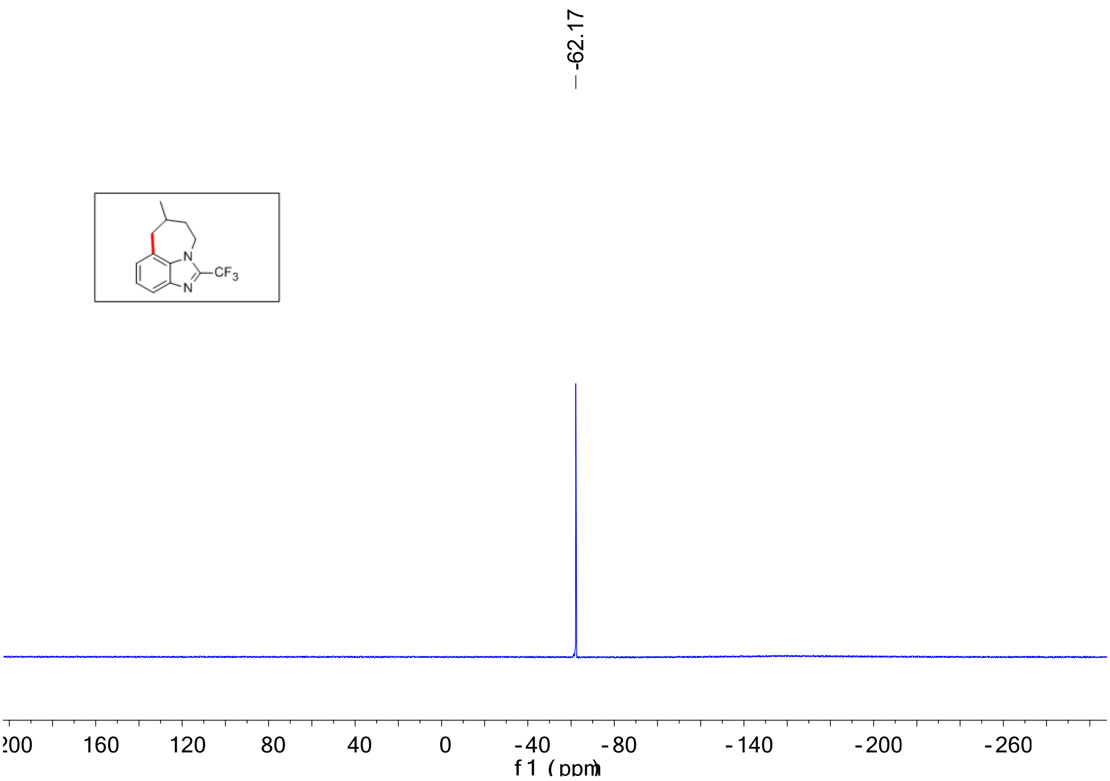

Supplementary Figure 112. ^{19}F NMR spectrum of compound **3a** in $CDCl_3$.

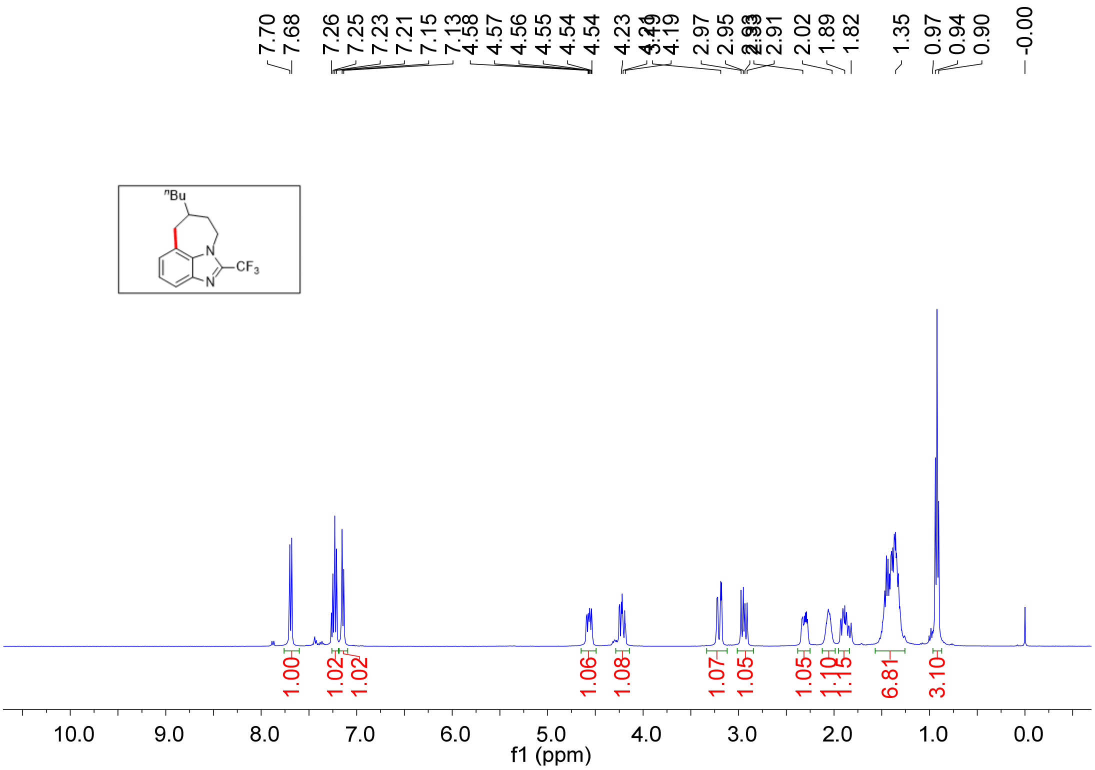

Supplementary Figure 113. ^{1}H NMR spectrum of compound **3b** in $CDCl_3$.

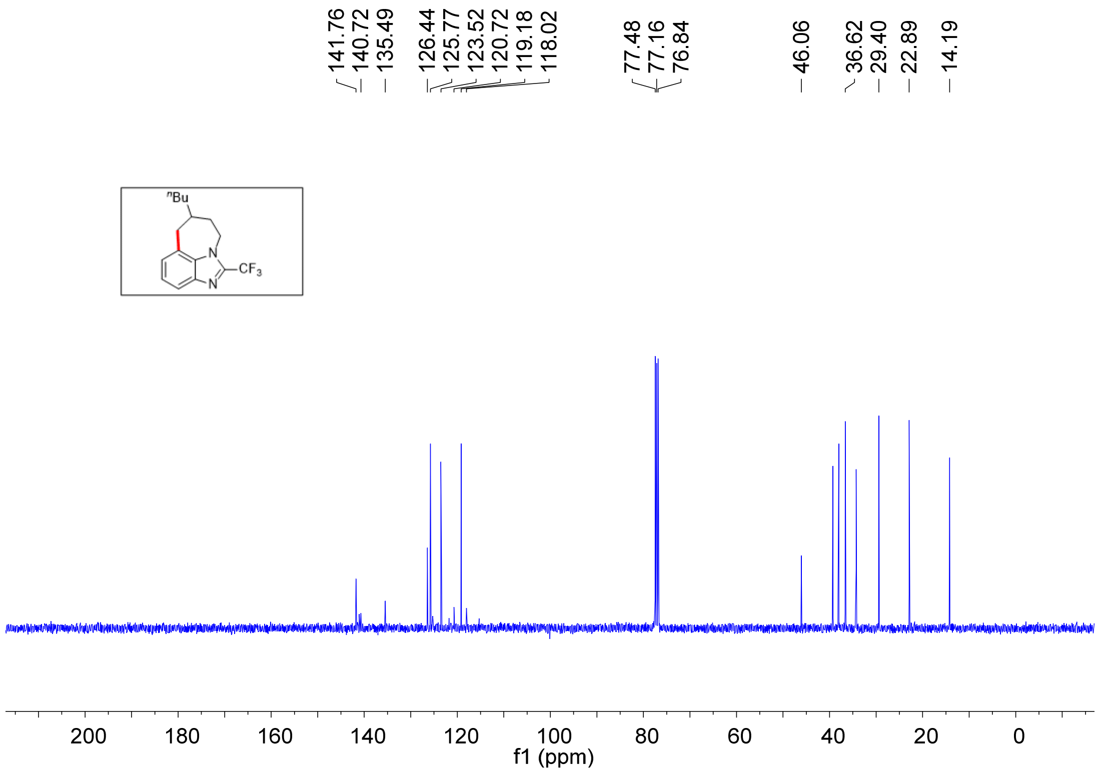

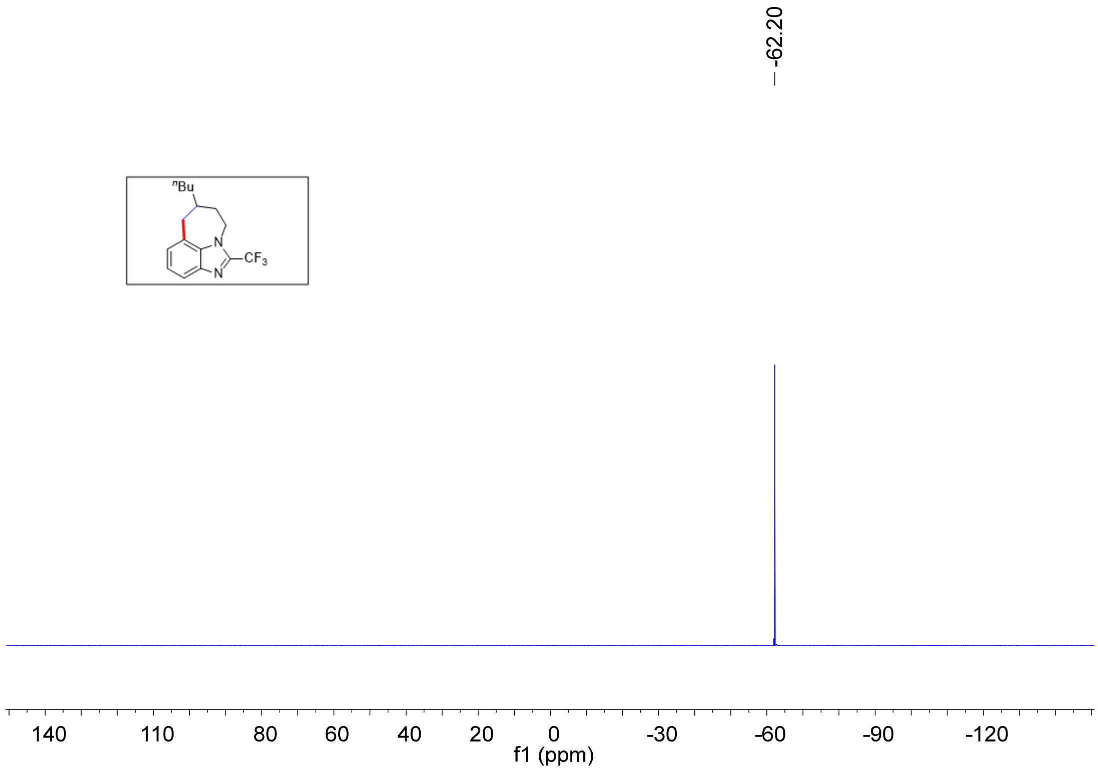

Supplementary Figure 114. ^{13}C and ^{19}F NMR spectra of compound **3b** in $CDCl_3$.

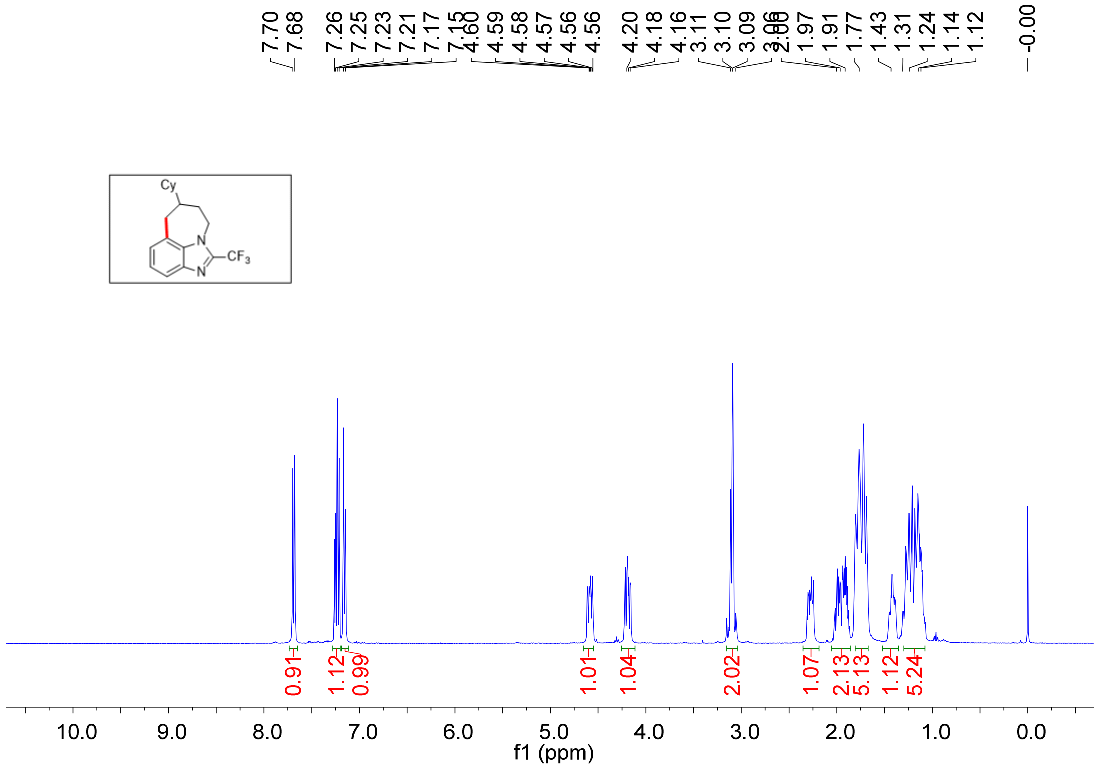

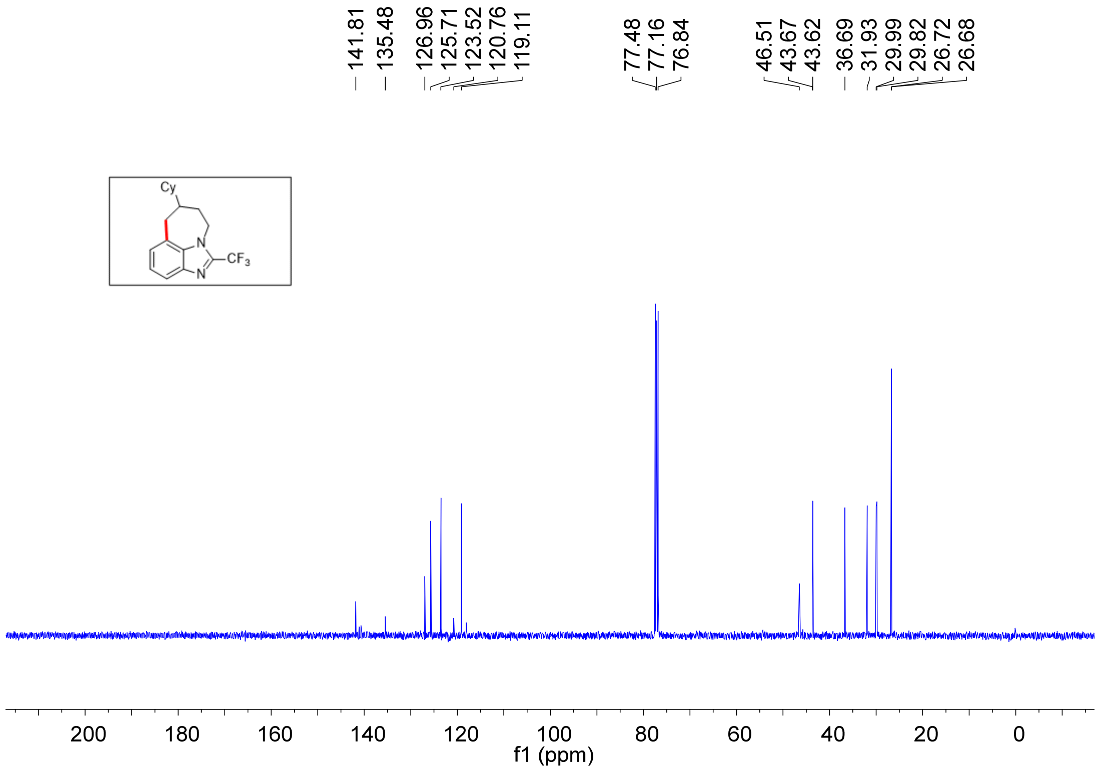

Supplementary Figure 115. ^{1}H and ^{13}C NMR spectra of compound **3c** in $CDCl_3$.

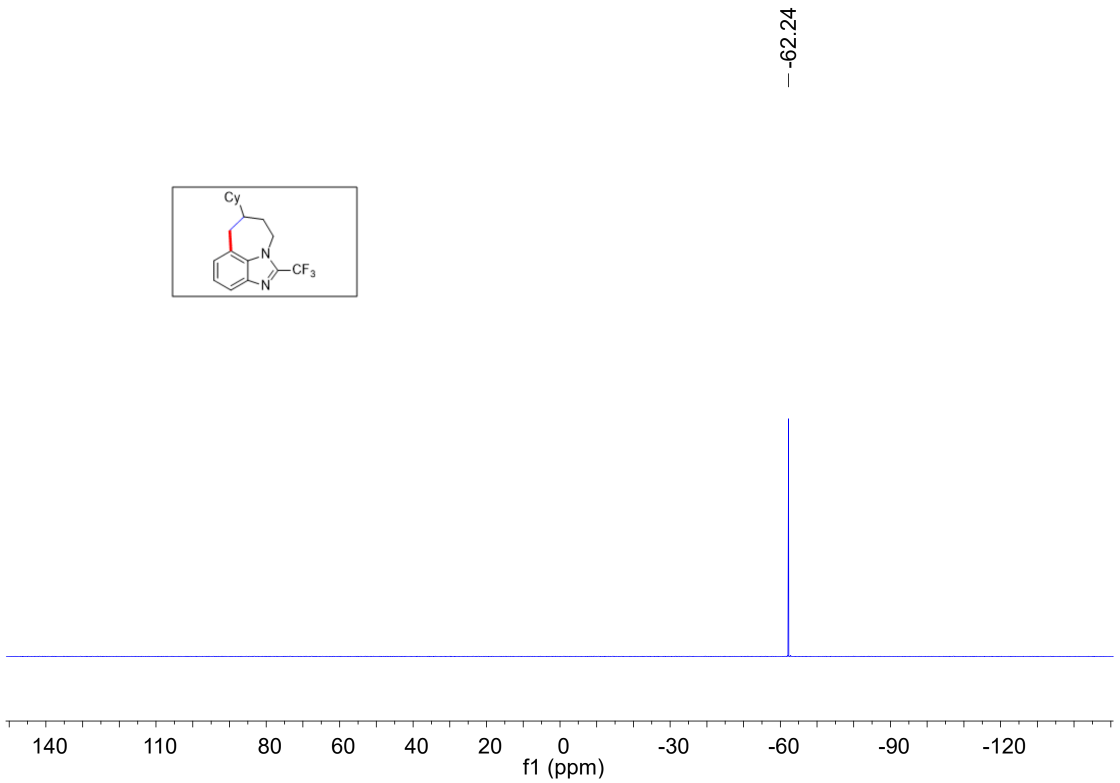

Supplementary Figure 116. ^{19}F NMR spectrum of compound **3c** in $CDCl_3$.

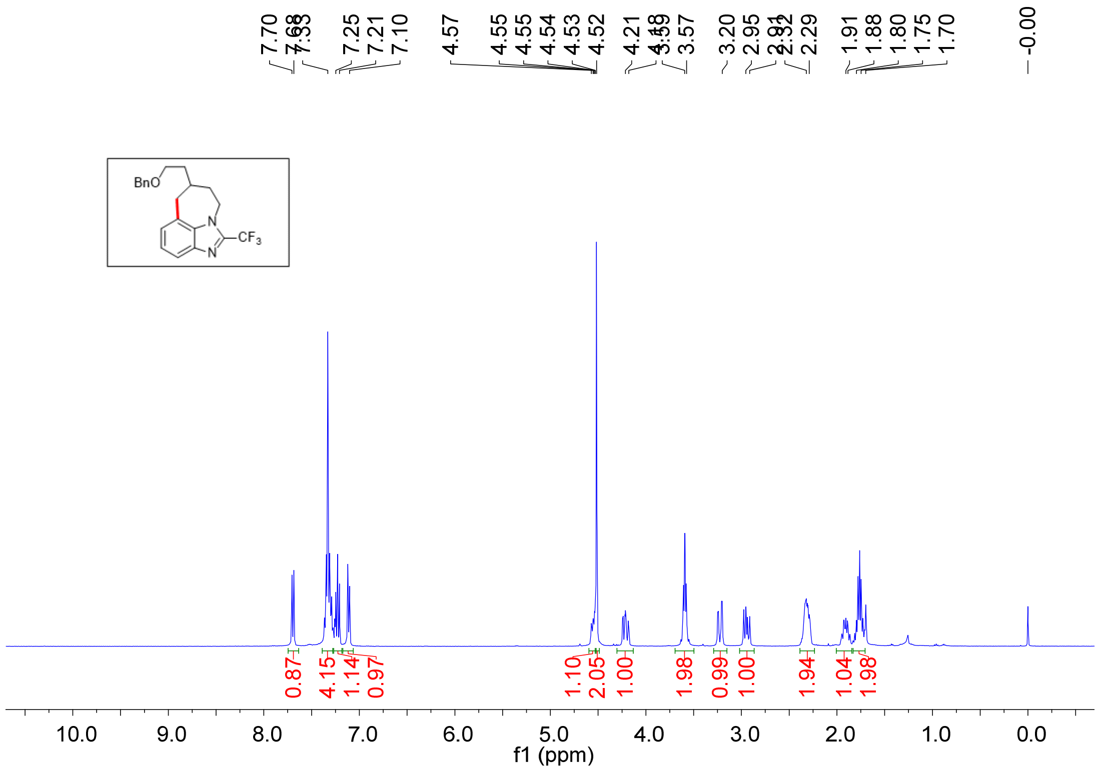

Supplementary Figure 117. ^{1}H NMR spectrum of compound **3d** in $CDCl_3$.

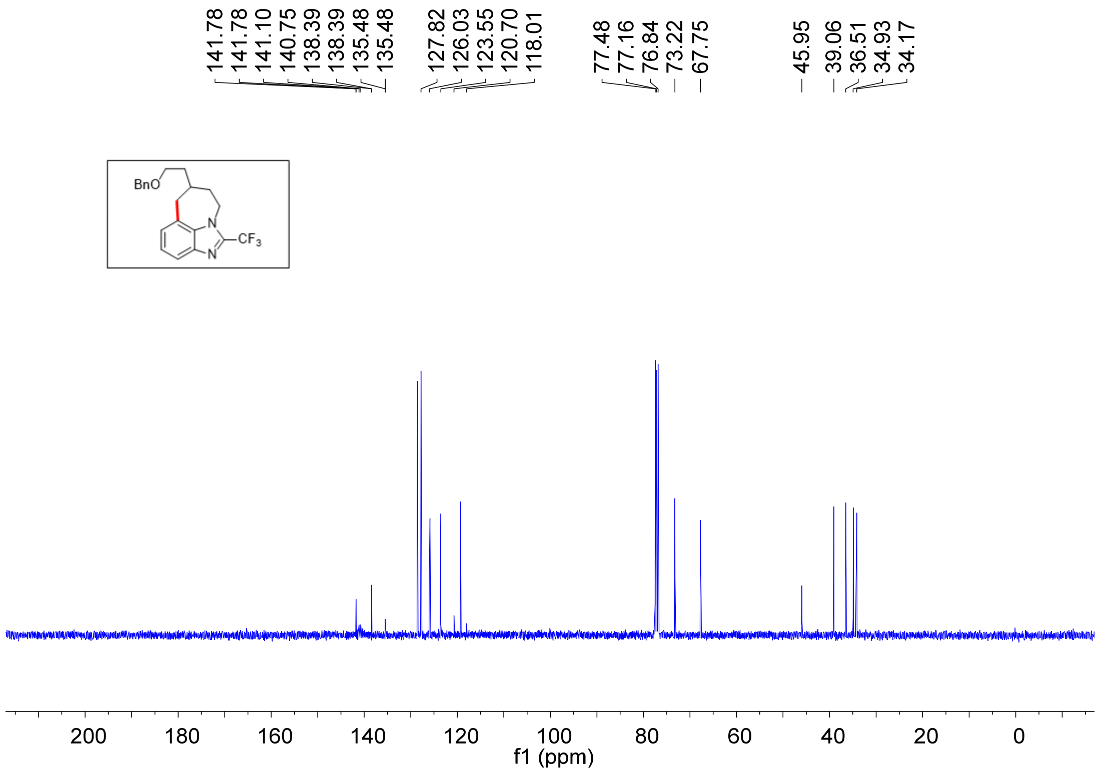

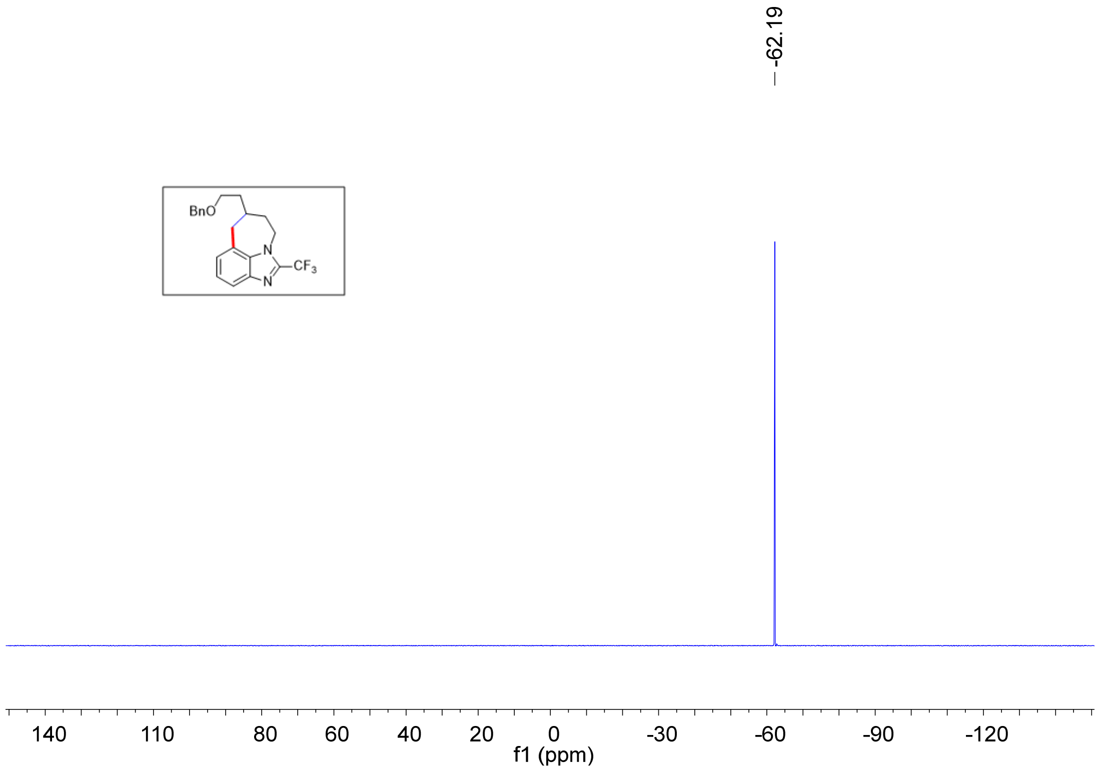

Supplementary Figure 118. ^{13}C and ^{19}F NMR spectra of compound **3d** in $CDCl_3$.

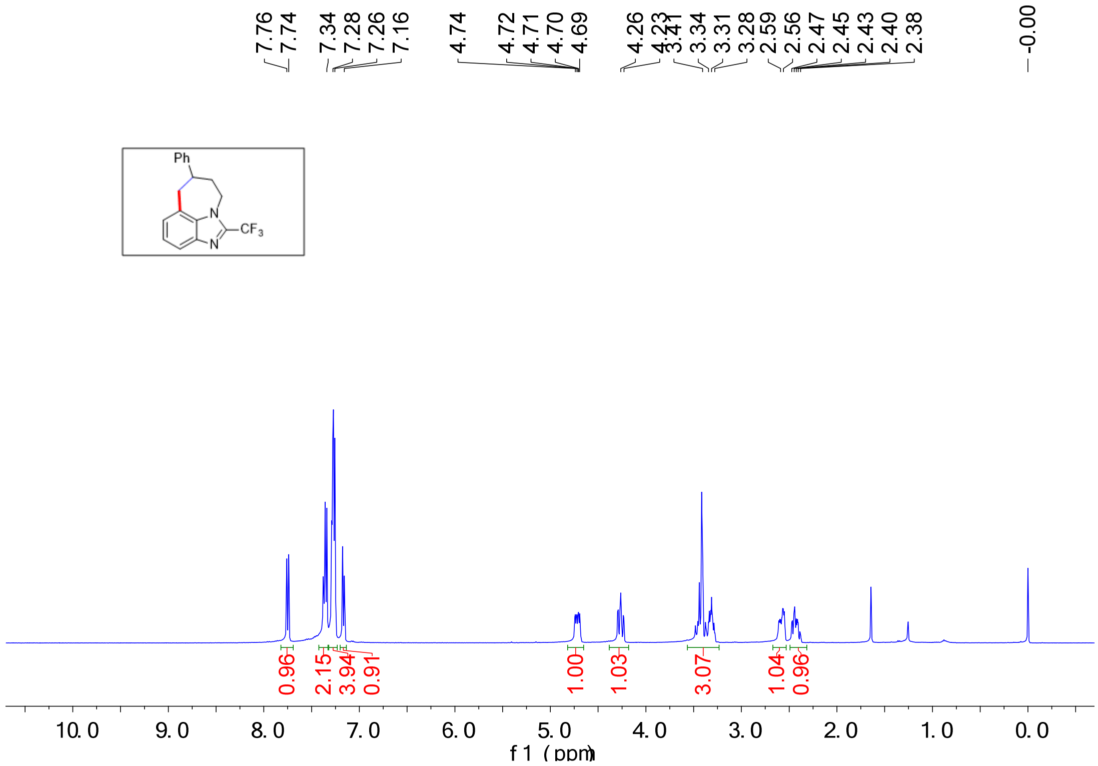

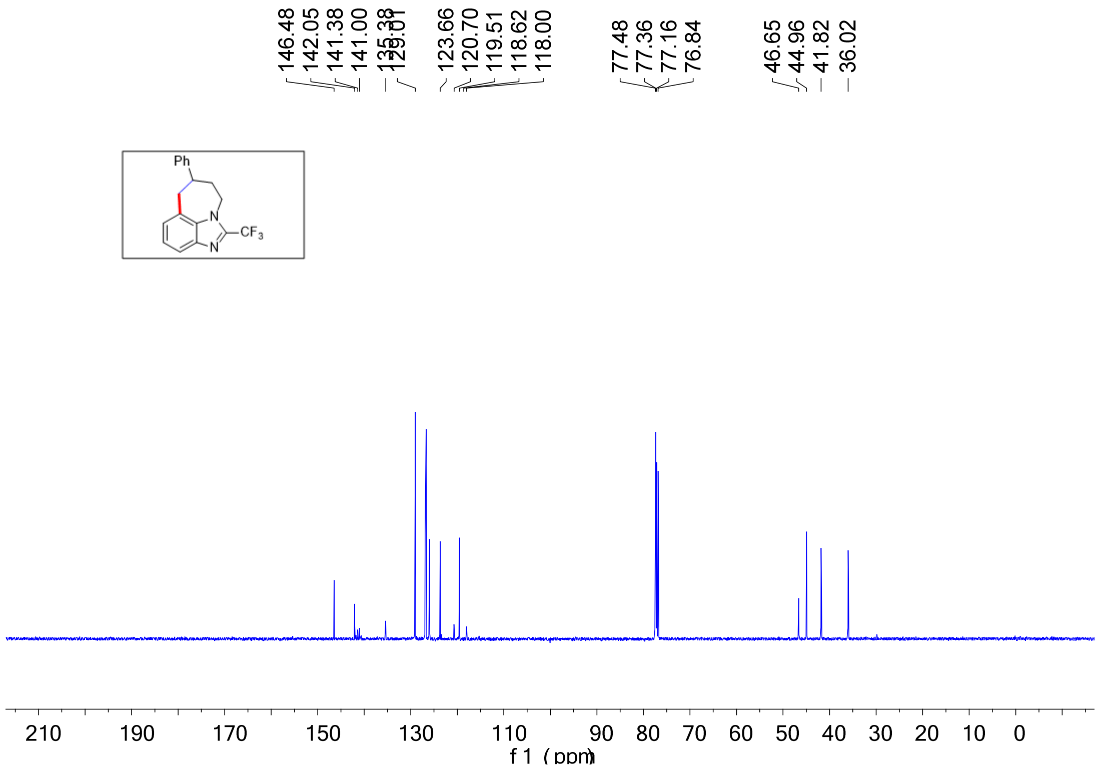

Supplementary Figure 119. ^{1}H and ^{13}C NMR spectra of compound **3e** in $CDCl_3$.

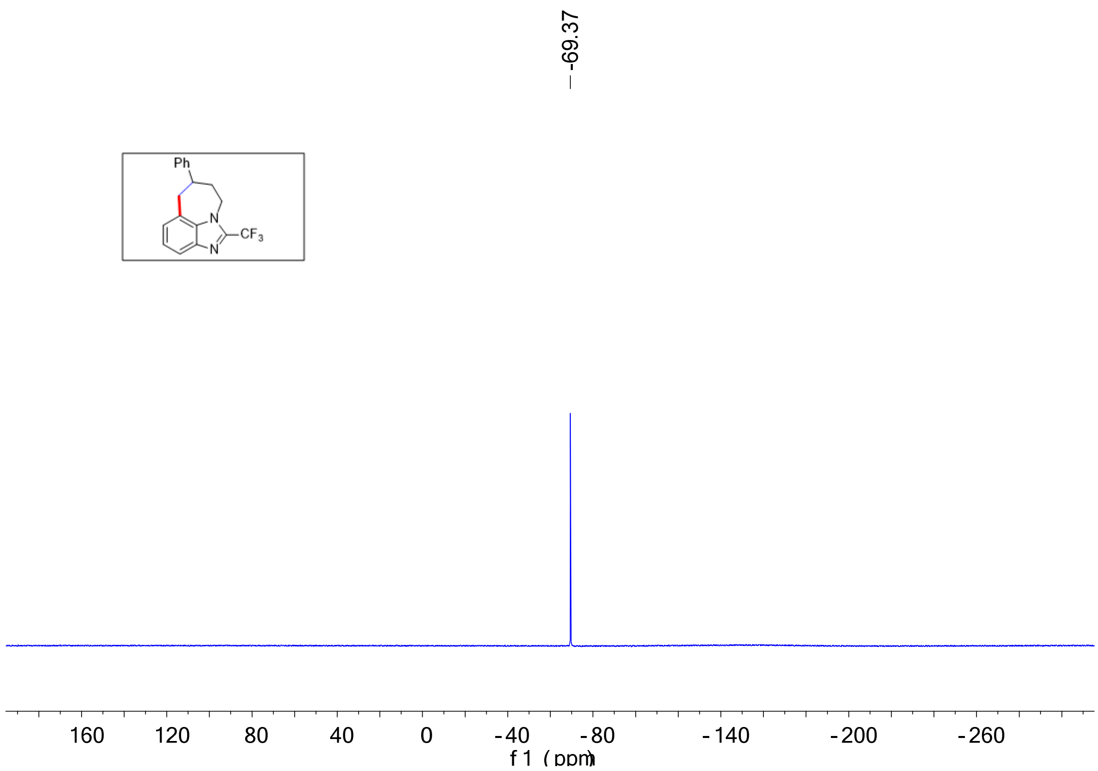

Supplementary Figure 120. ^{19}F NMR spectrum of compound **3e** in $CDCl_3$.

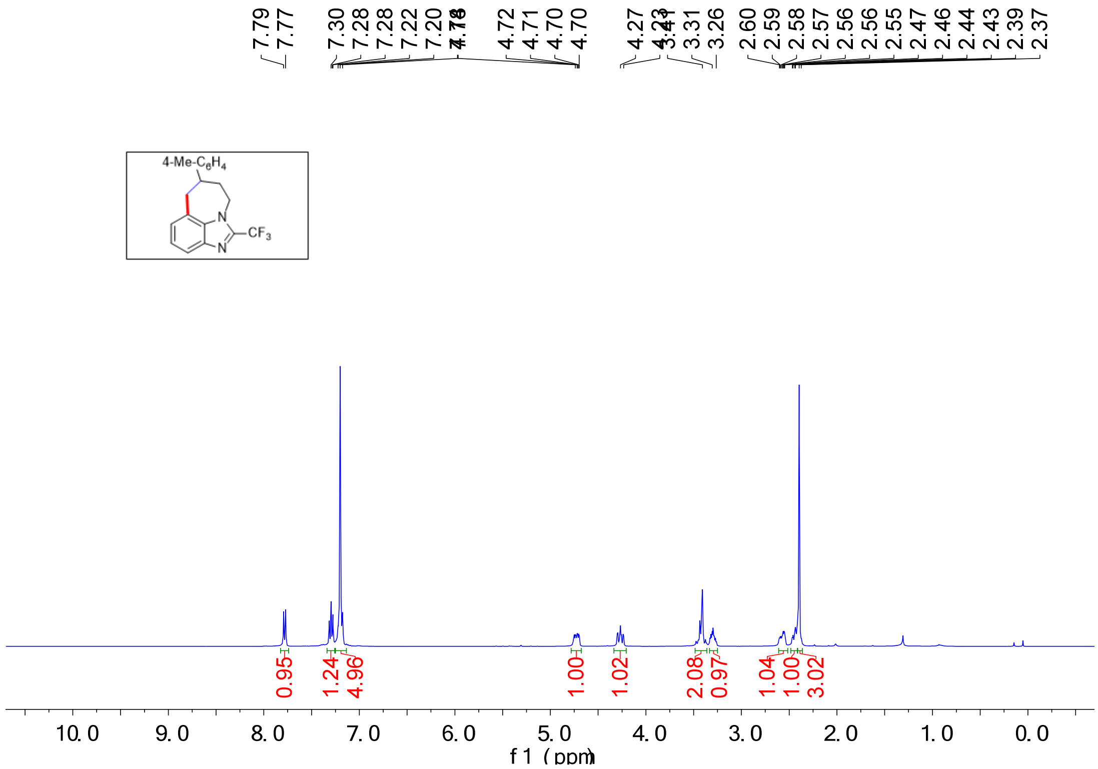

Supplementary Figure 121. ^{1}H NMR spectrum of compound **3f** in $CDCl_3$.

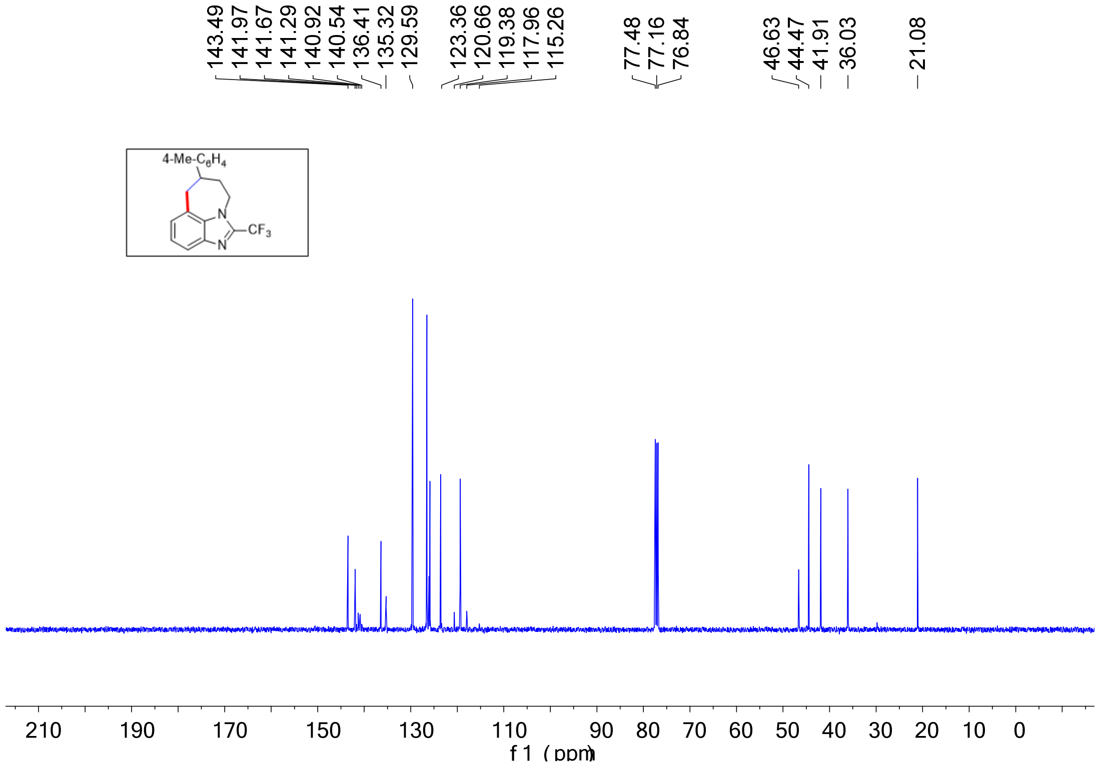

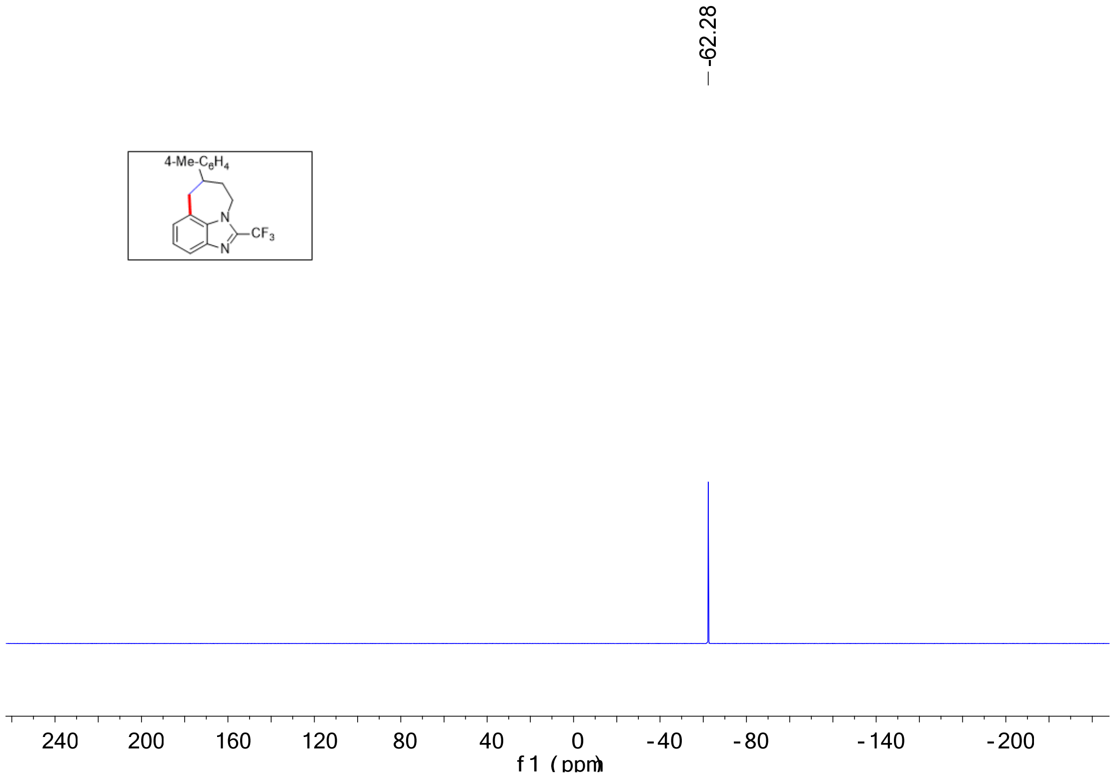

Supplementary Figure 122. ^{13}C and ^{19}F NMR spectra of compound **3f** in $CDCl_3$.

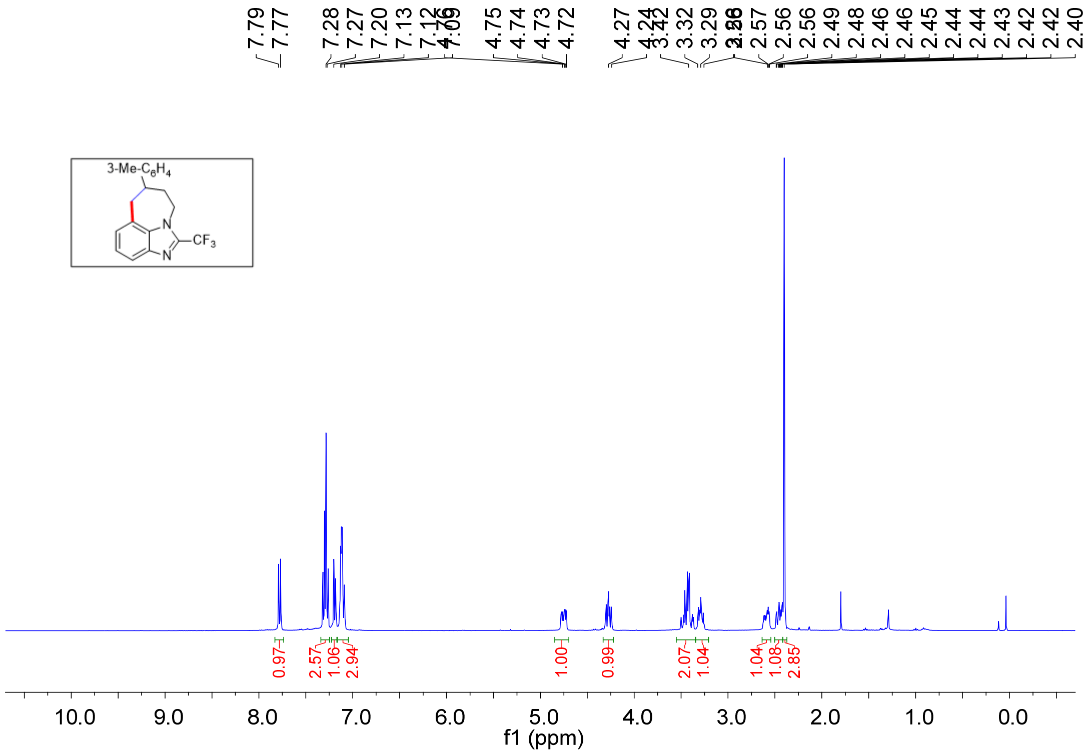

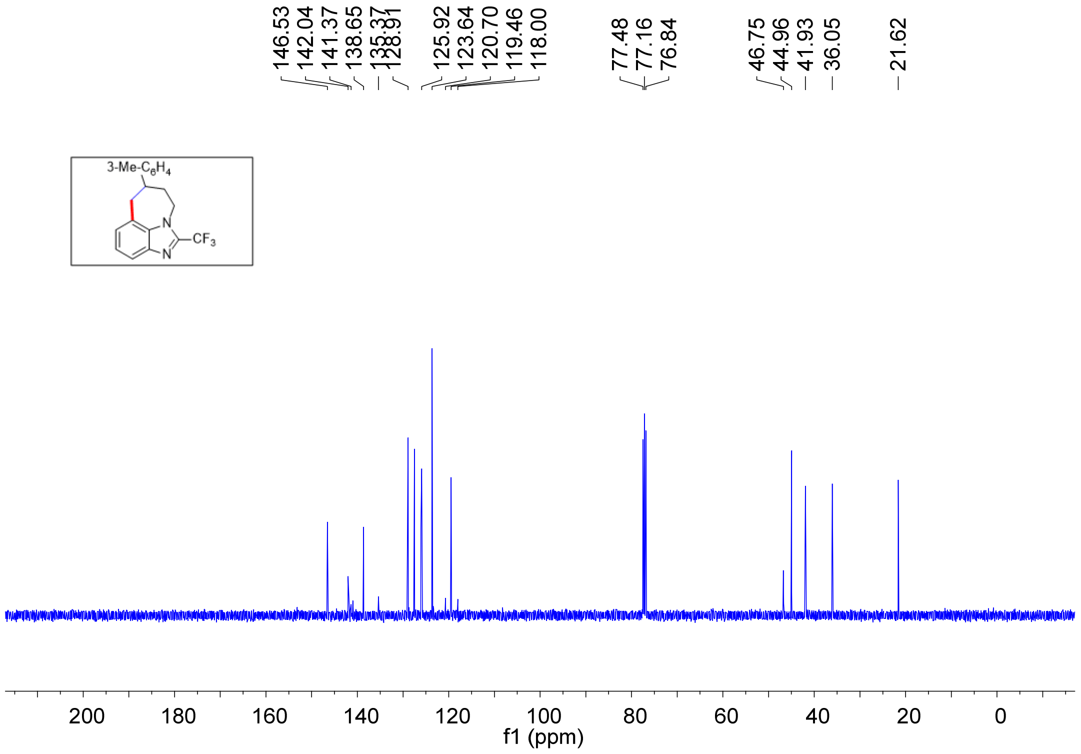

Supplementary Figure 123. ^1H and ^{13}C NMR spectra of compound **3g** in $CDCl_3$.

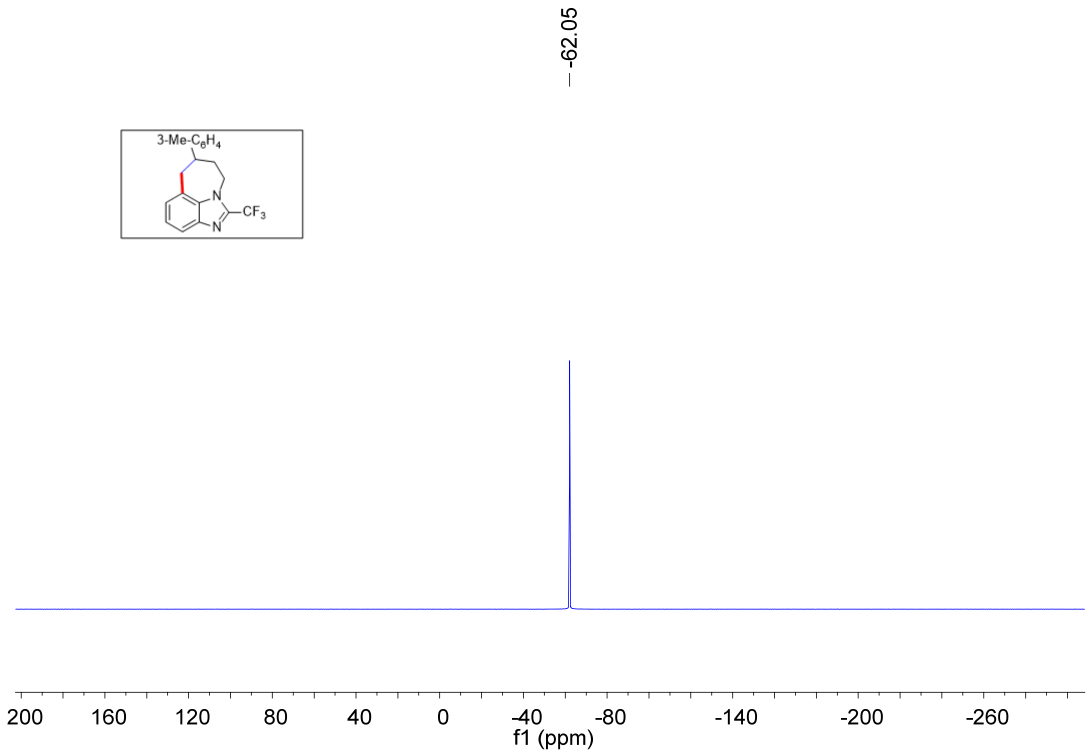

Supplementary Figure 124. ^{19}F NMR spectrum of compound **3g** in $CDCl_3$.

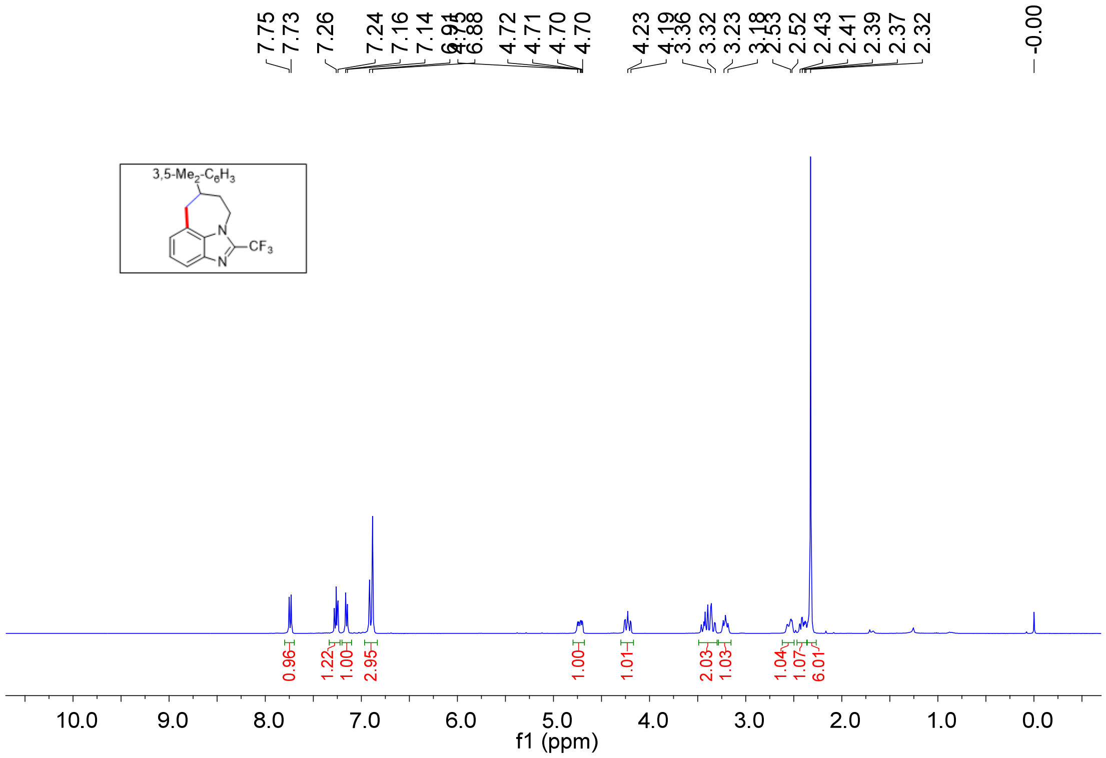

Supplementary Figure 125. ^{1}H spectrum of compound **3h** in $CDCl_3$.

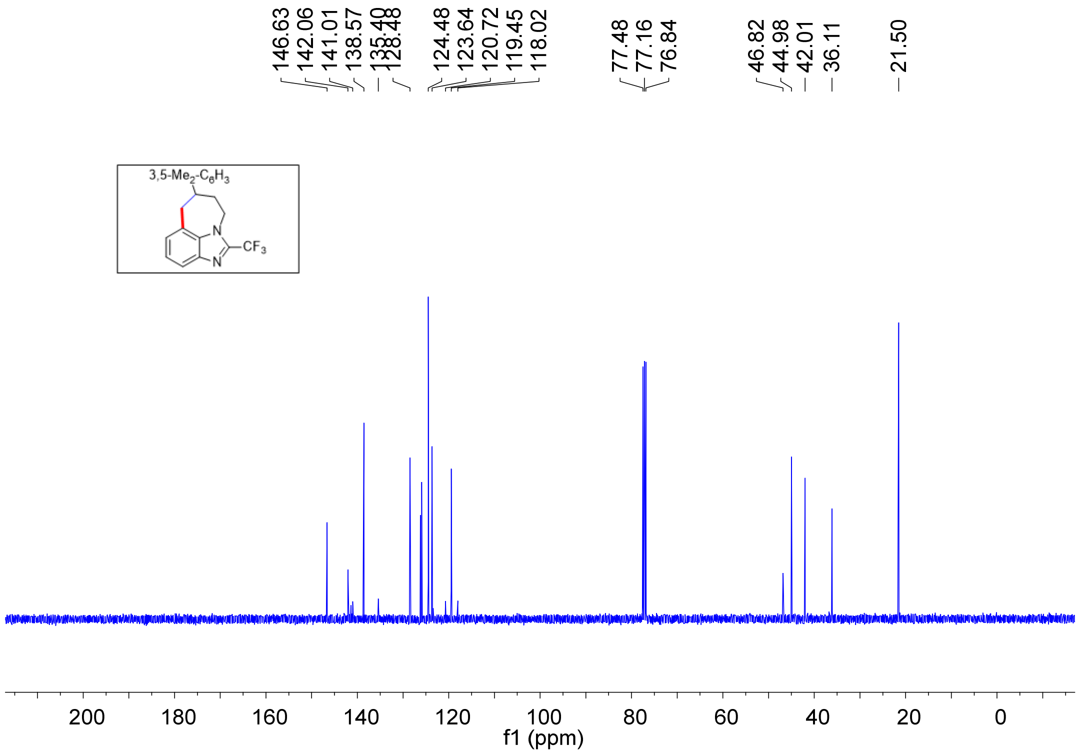

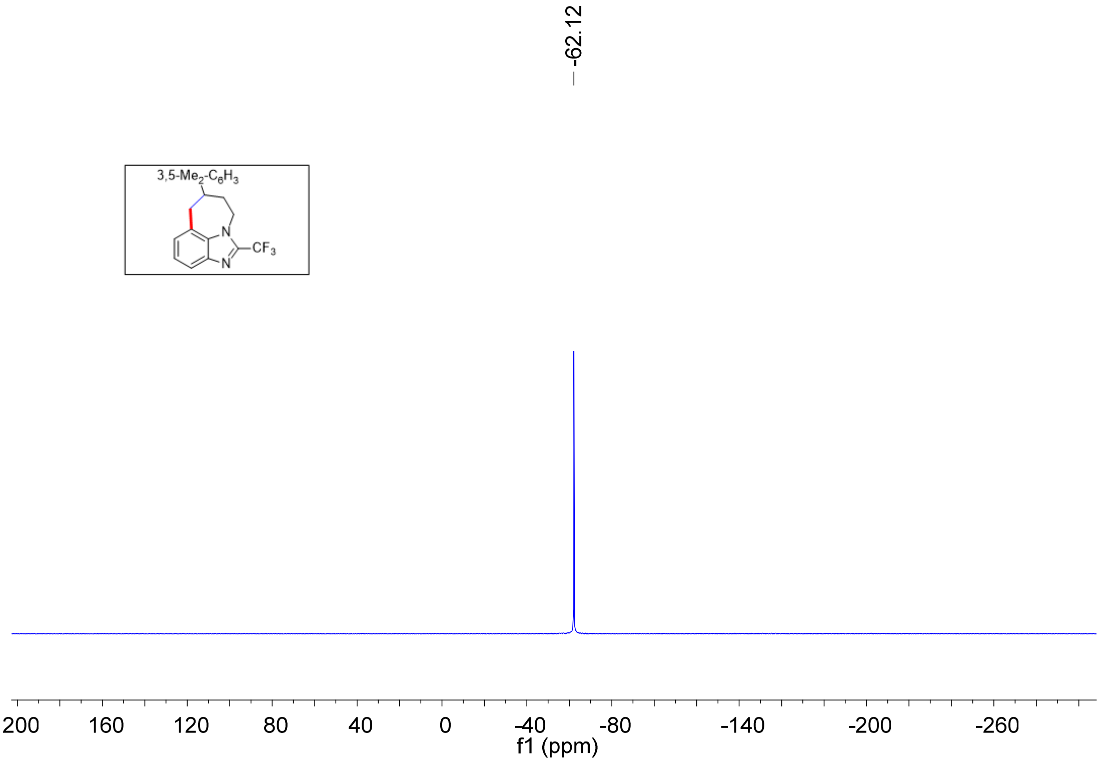

Supplementary Figure 126. ^{13}C and ^{19}F NMR spectra of compound **3h** in $CDCl_3$.

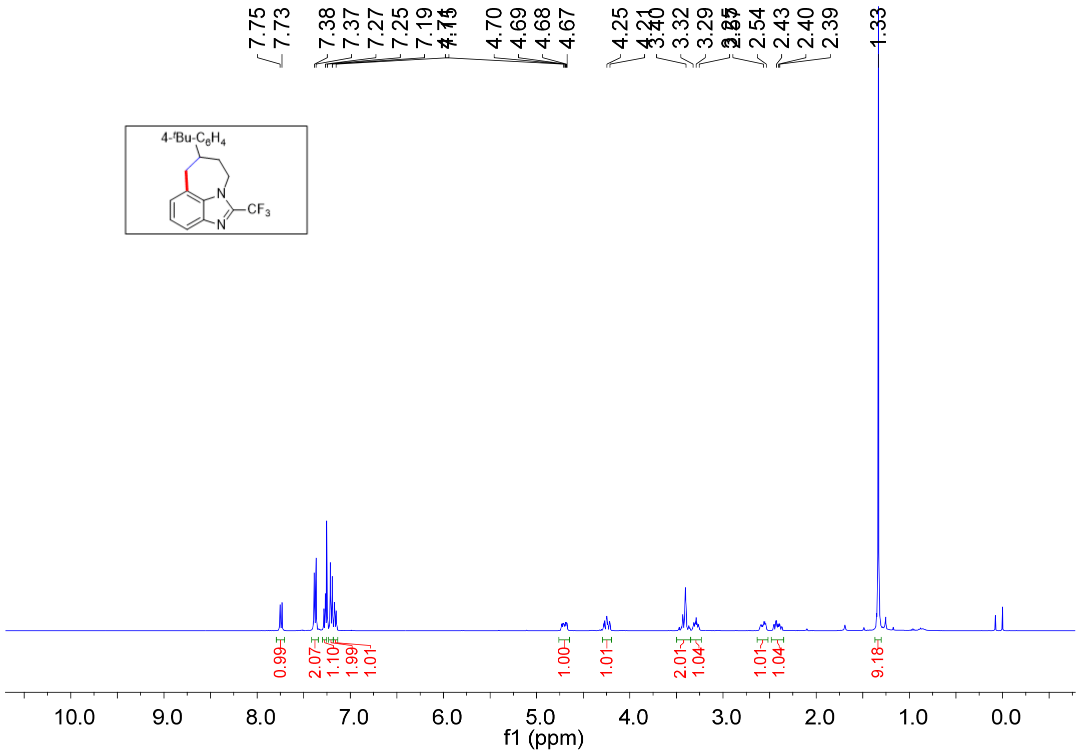

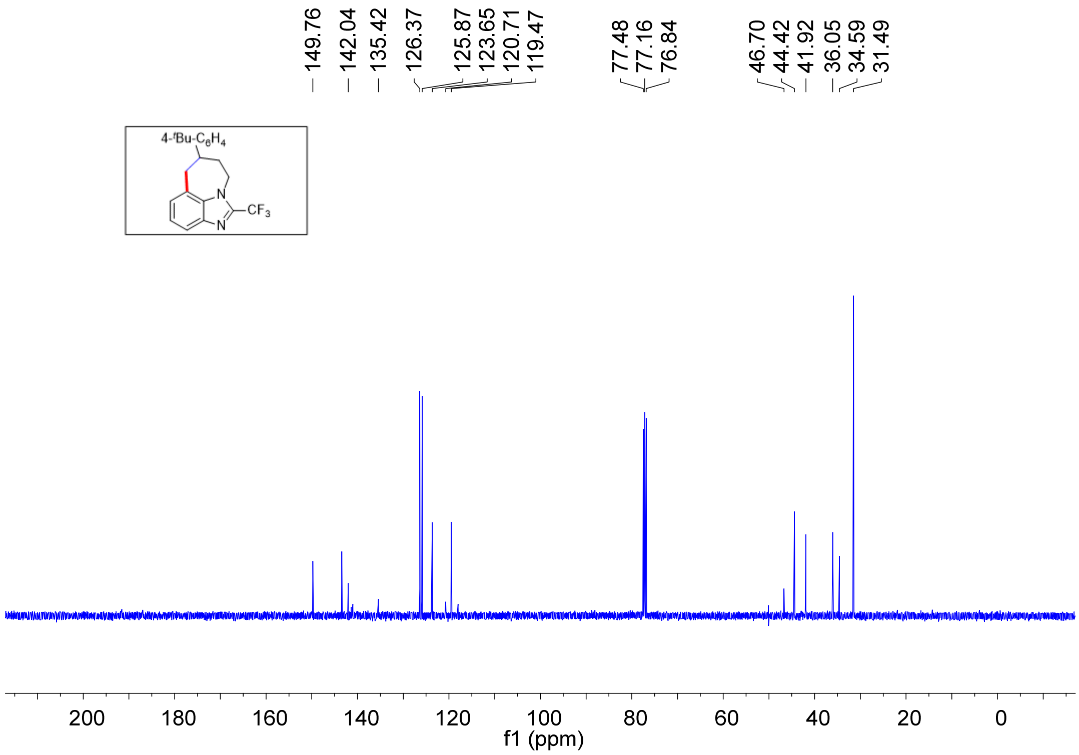

Supplementary Figure 127. ^{1}H and ^{13}C NMR spectra of compound **3i** in $CDCl_3$.

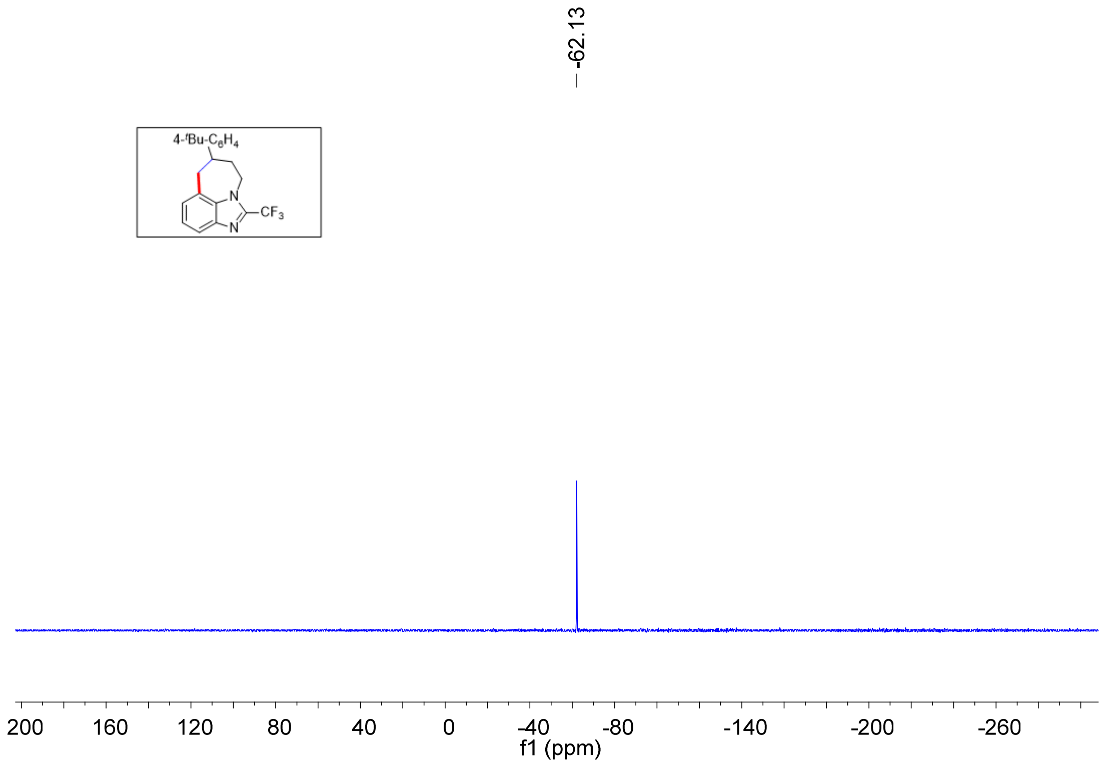

Supplementary Figure 128. ^{19}F NMR spectrum of compound **3i** in $CDCl_3$.

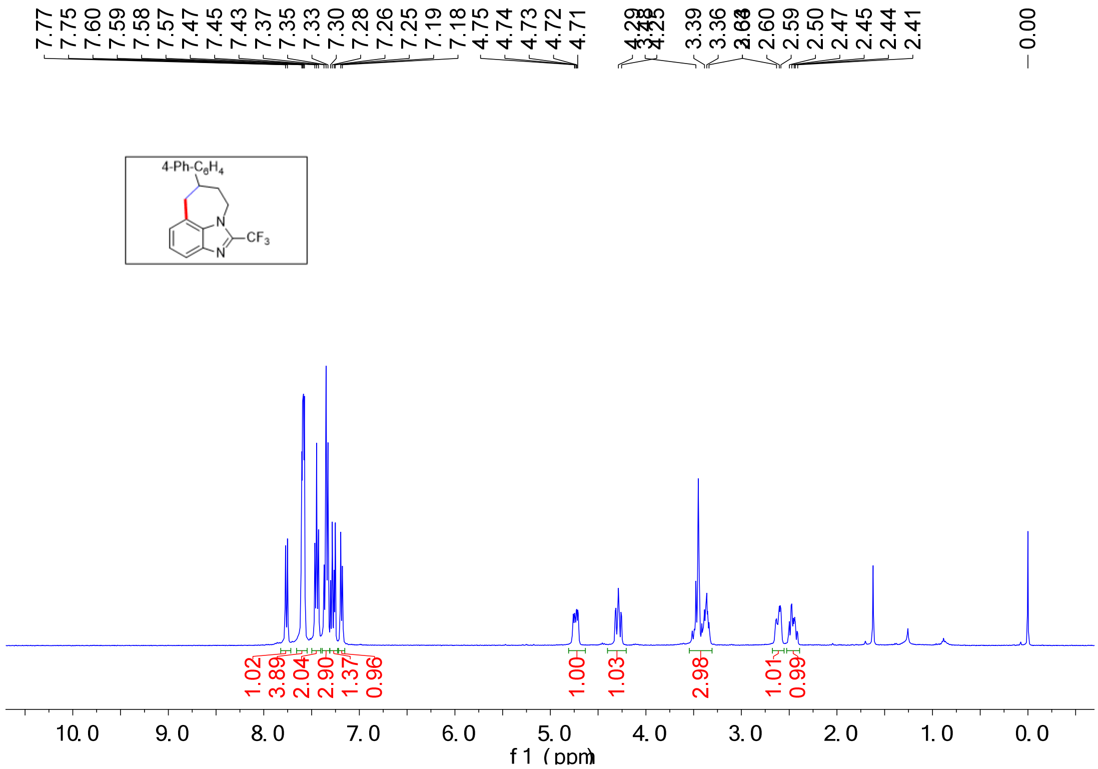

Supplementary Figure 129. ^{1}H spectrum of compound **3j** in $CDCl_3$.

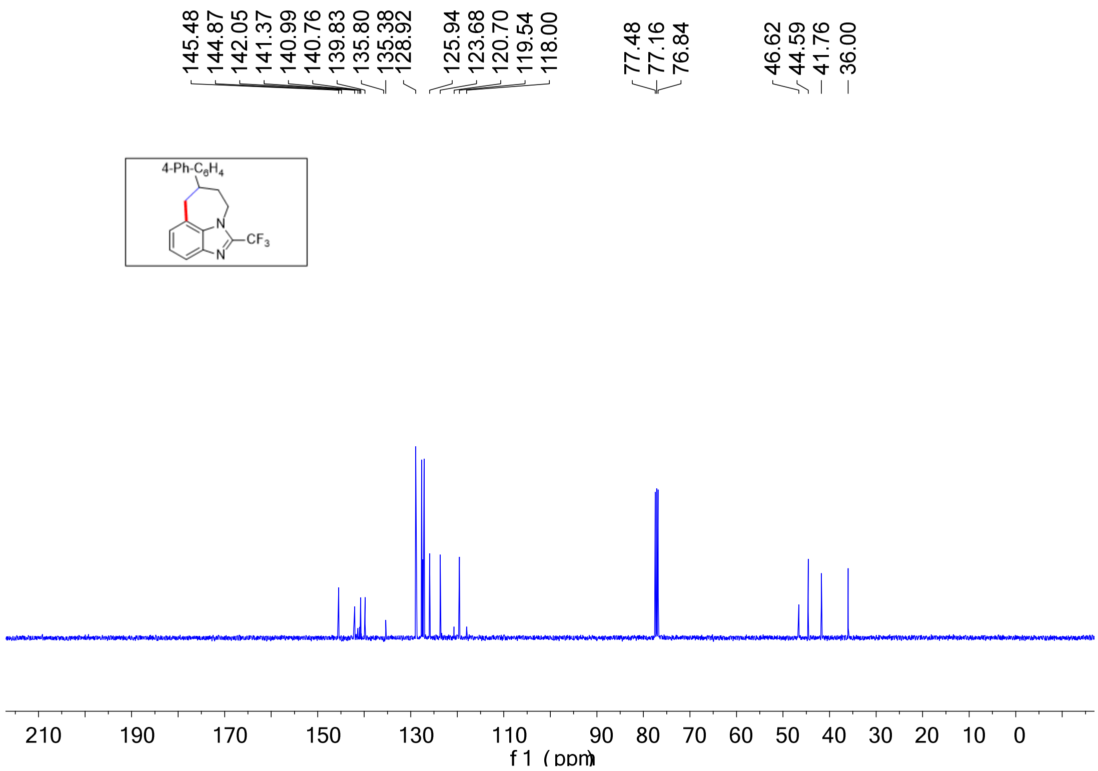

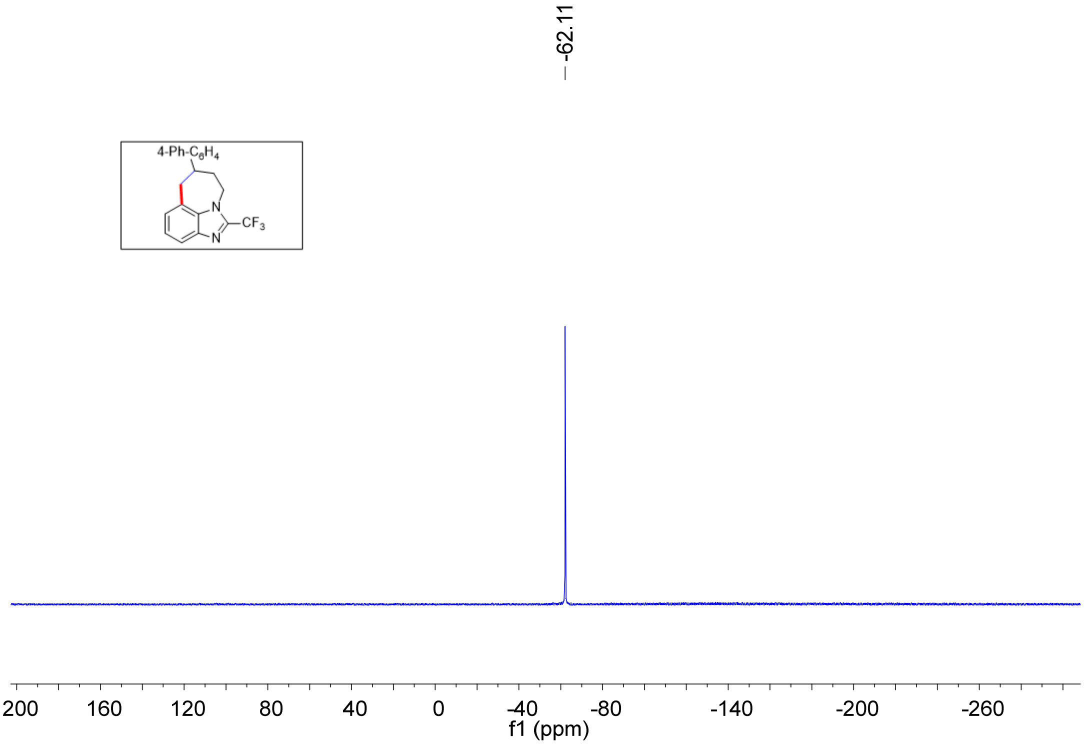

Supplementary Figure 130. ^{13}C and ^{19}F NMR spectra of compound **3j** in $CDCl_3$.

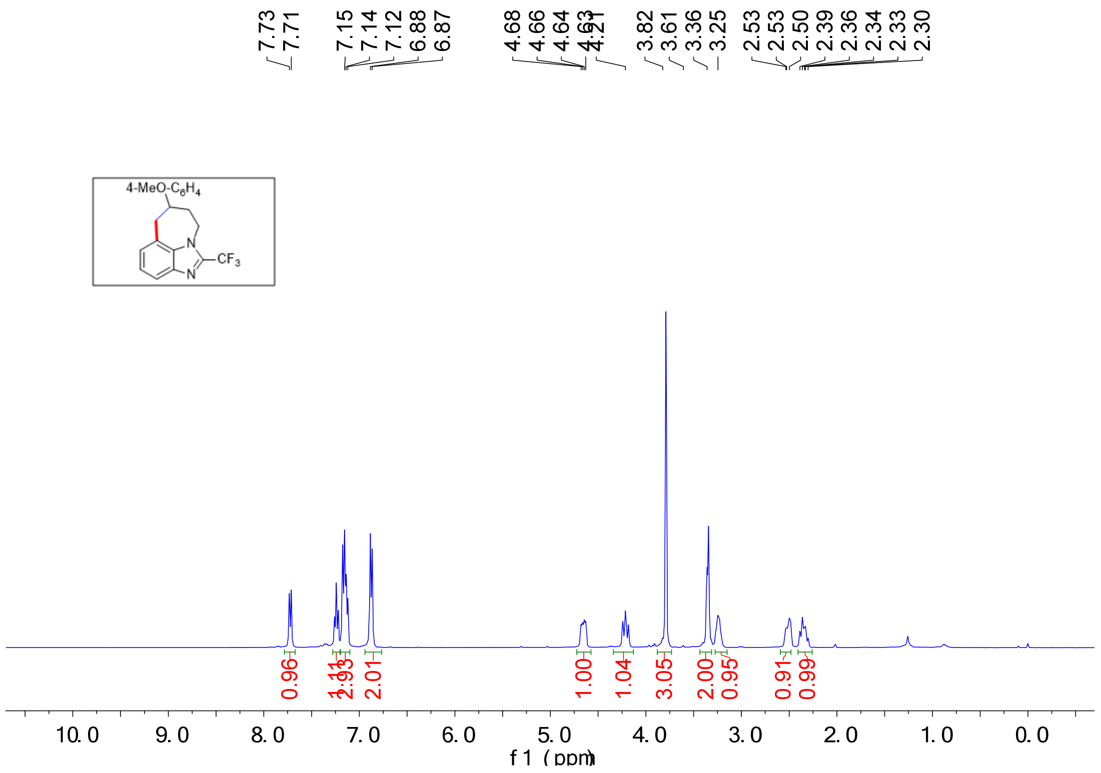

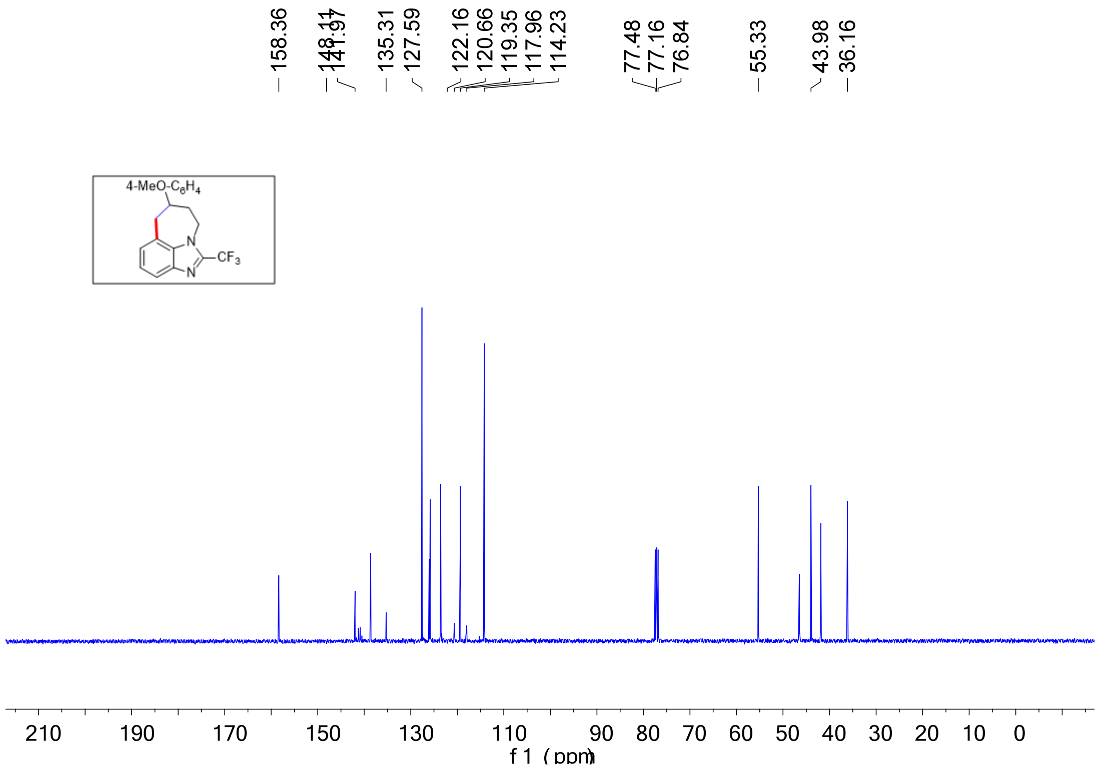

Supplementary Figure 131. ^{1}H and ^{13}C NMR spectra of compound **3k** in $CDCl_3$.

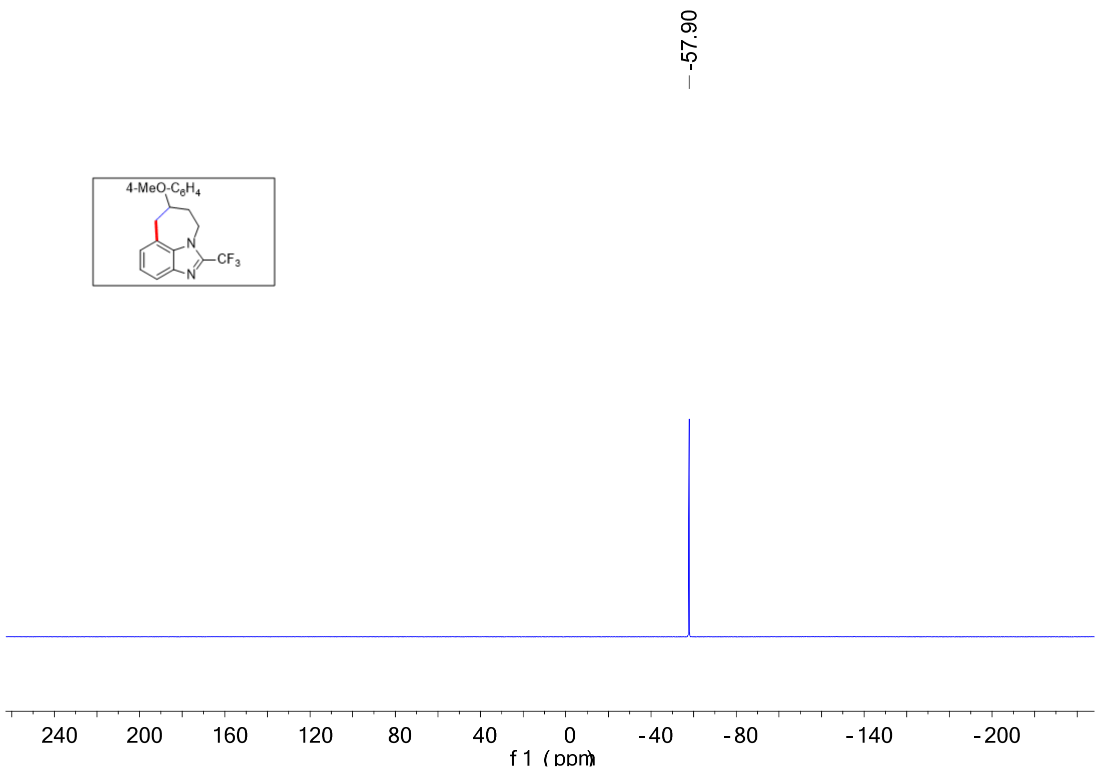

Supplementary Figure 132. ^{19}F NMR spectrum of compound **3k** in $CDCl_3$.

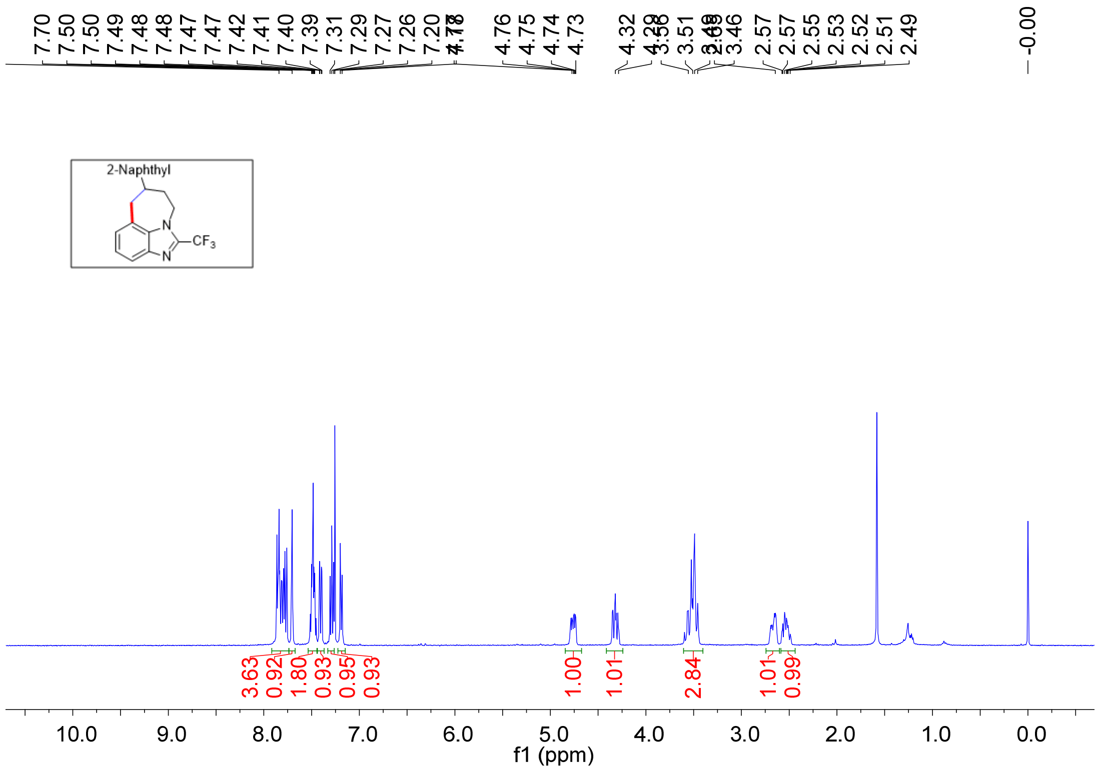

Supplementary Figure 133. ^{1}H NMR spectrum of compound **3l** in $CDCl_3$.

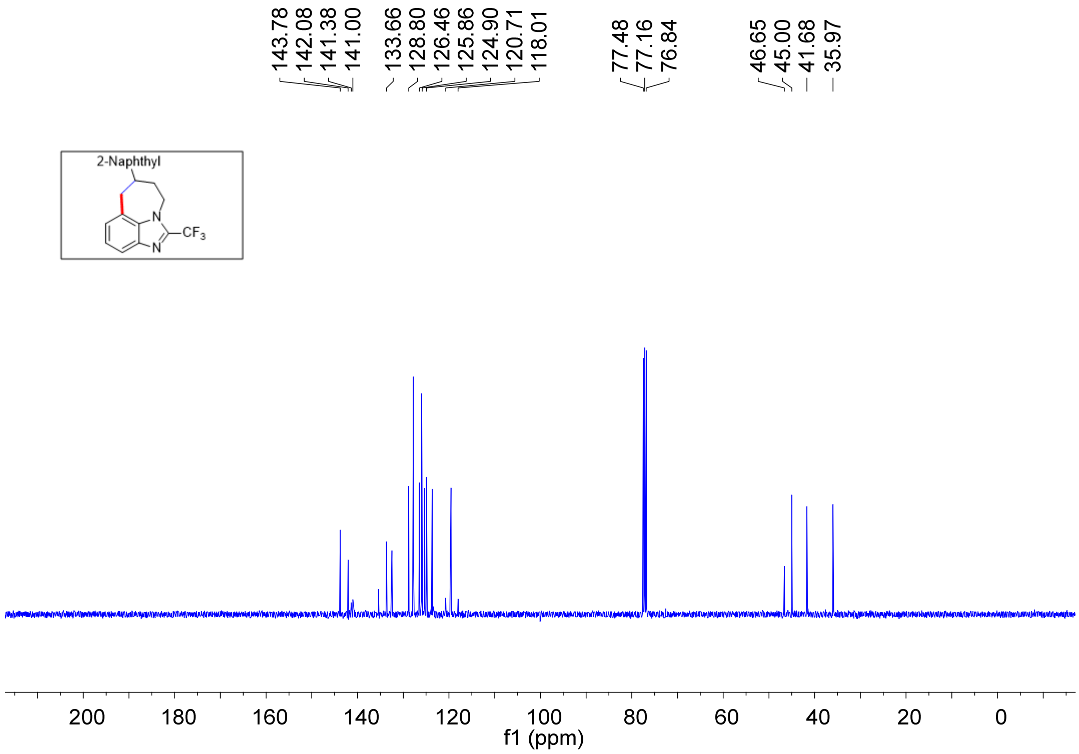

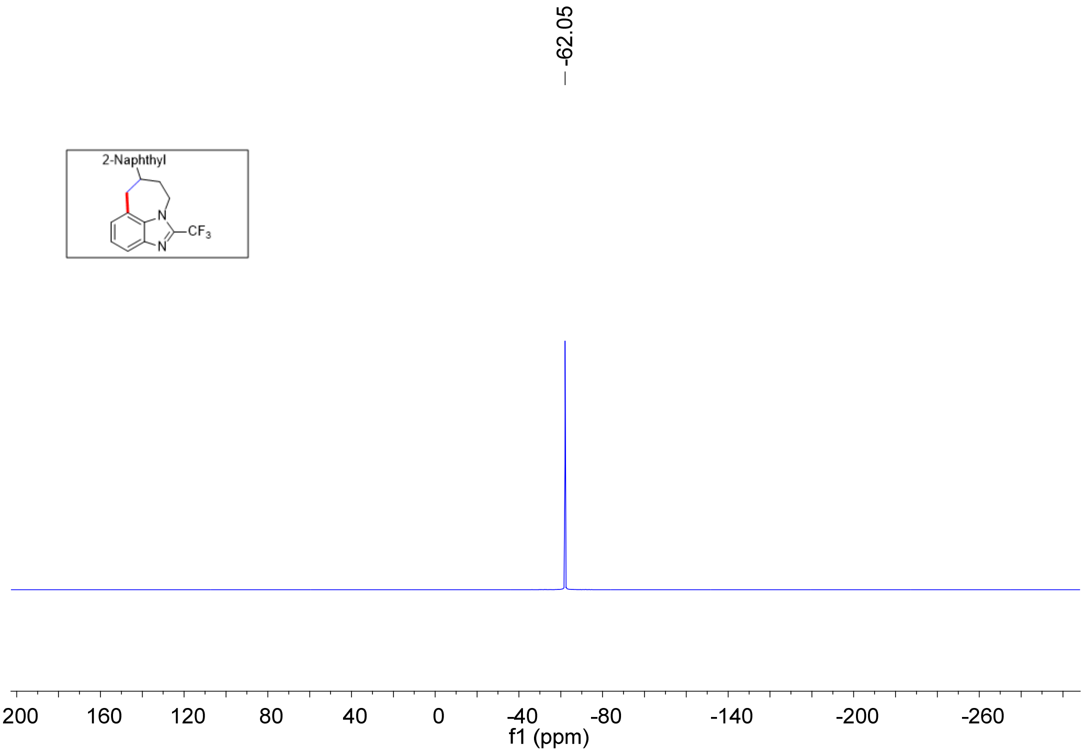

Supplementary Figure 134. ^{13}C and ^{19}F NMR spectra of compound **3l** in $CDCl_3$.

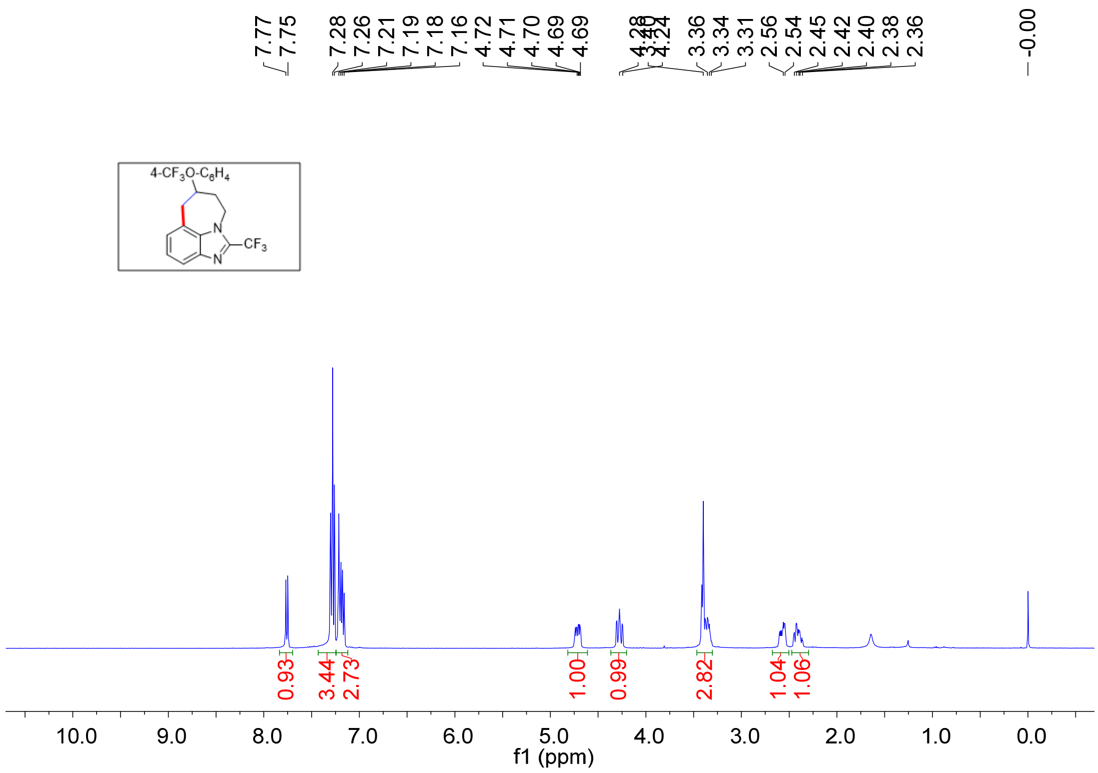

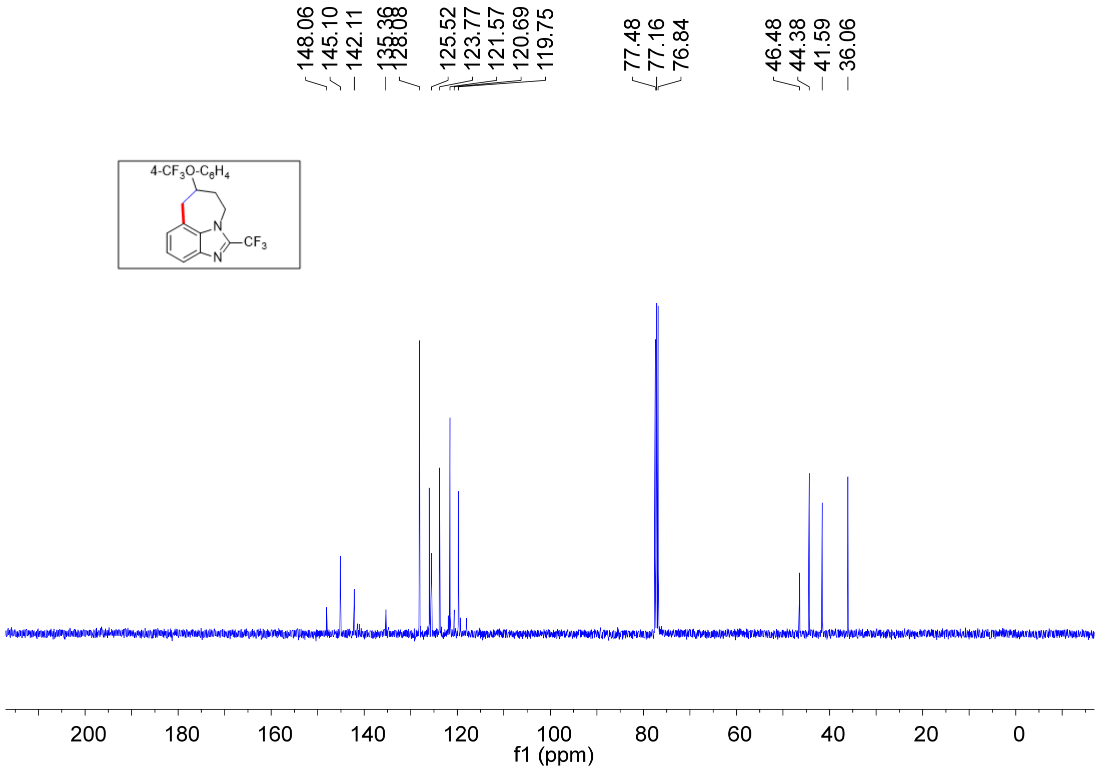

Supplementary Figure 135. ^{1}H and ^{13}C NMR spectra of compound **3m** in $CDCl_3$.

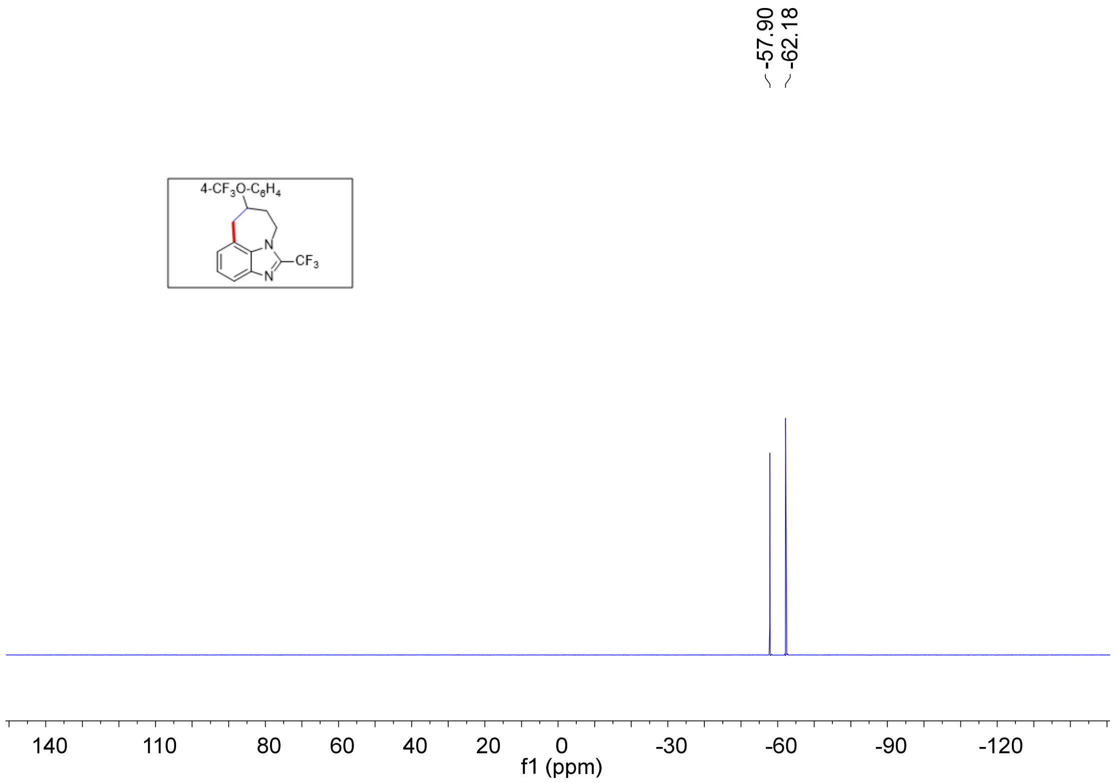

Supplementary Figure 136. ^{19}F NMR spectrum of compound **3m** in $CDCl_3$.

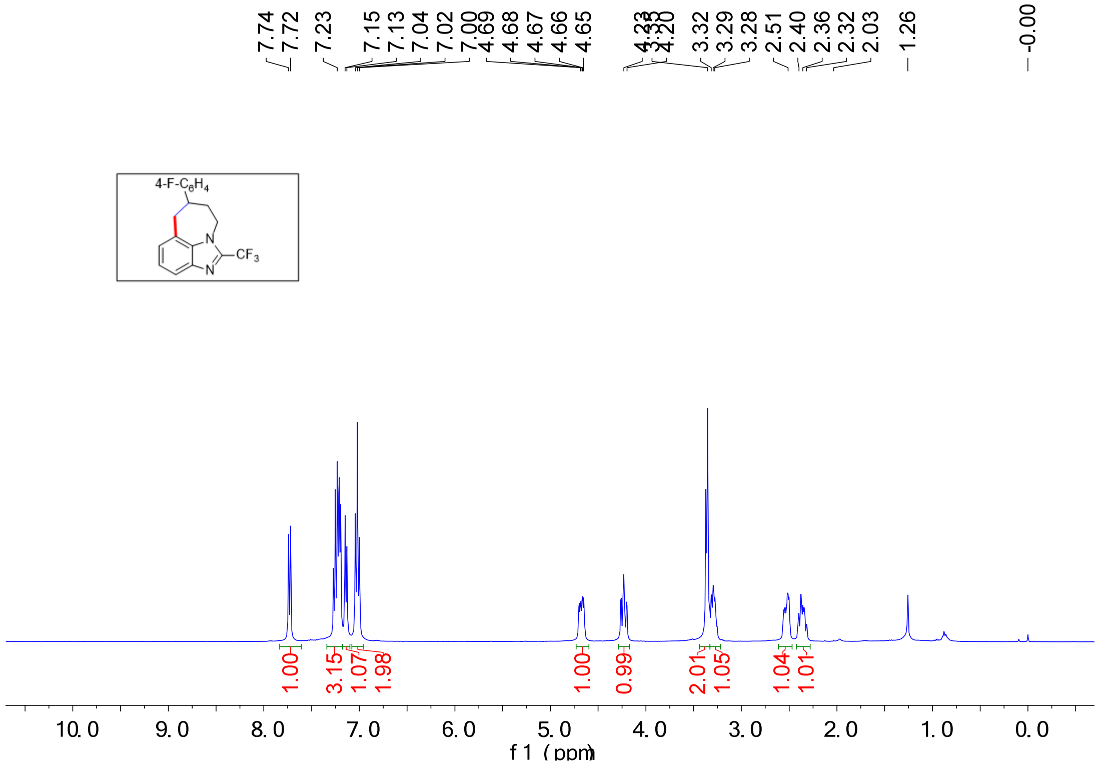

Supplementary Figure 137. ^{1}H NMR spectrum of compound **3n** in $CDCl_3$.

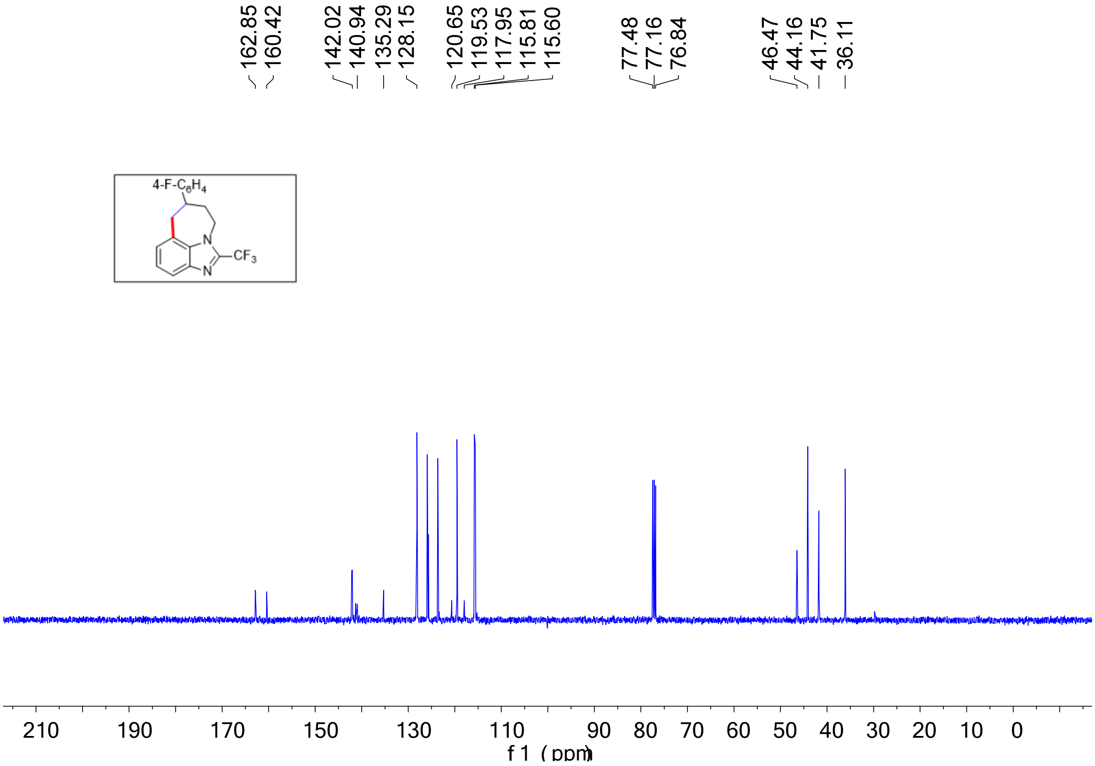

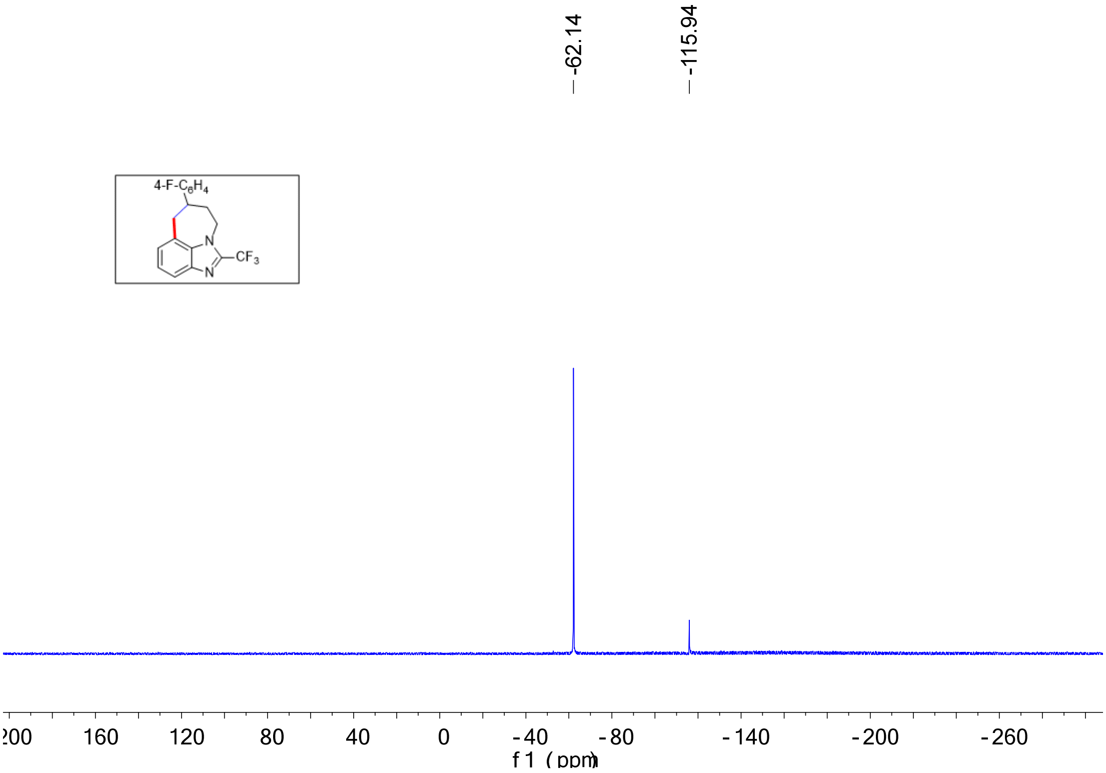

Supplementary Figure 138. ^{13}C and ^{19}F NMR spectra of compound **3n** in $CDCl_3$.

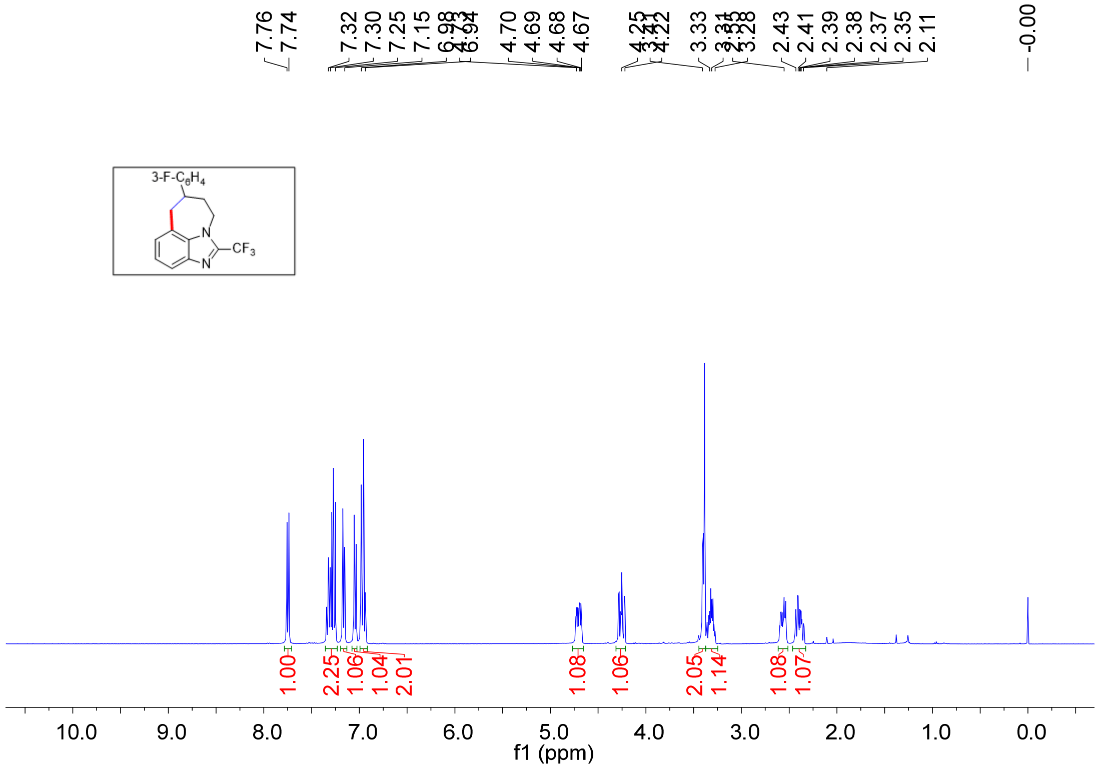

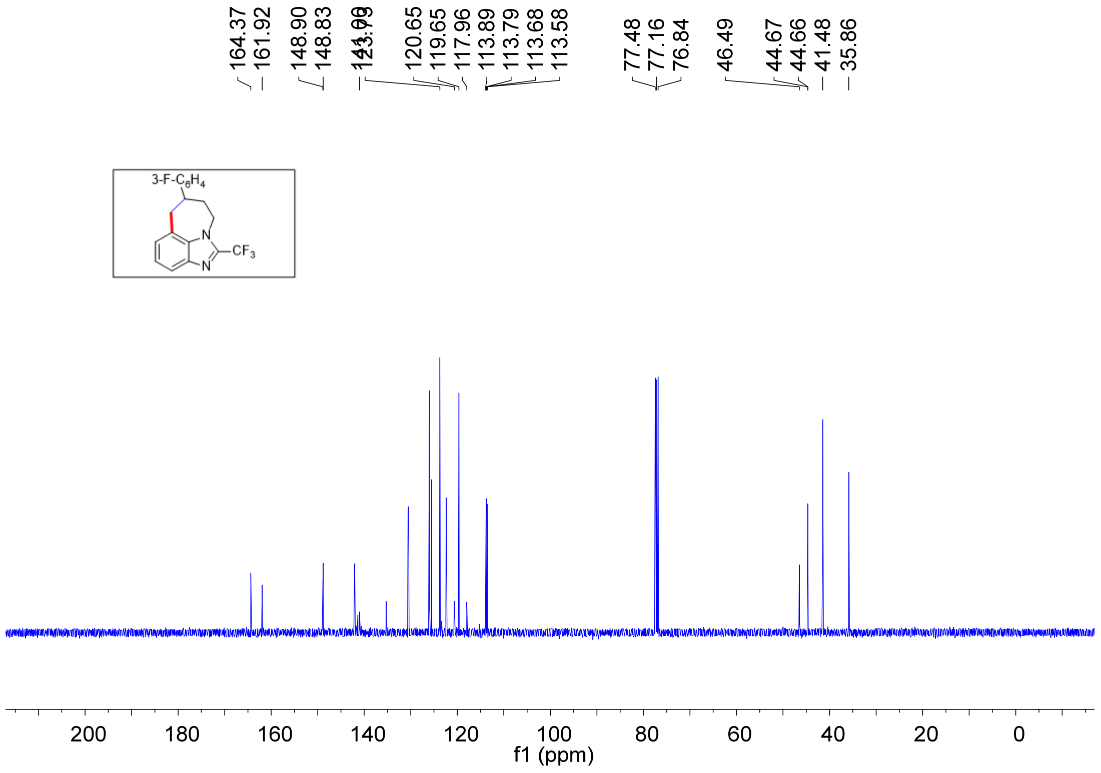

Supplementary Figure 139. ^{1}H and ^{13}C NMR spectra of compound **3o** in $CDCl_3$.

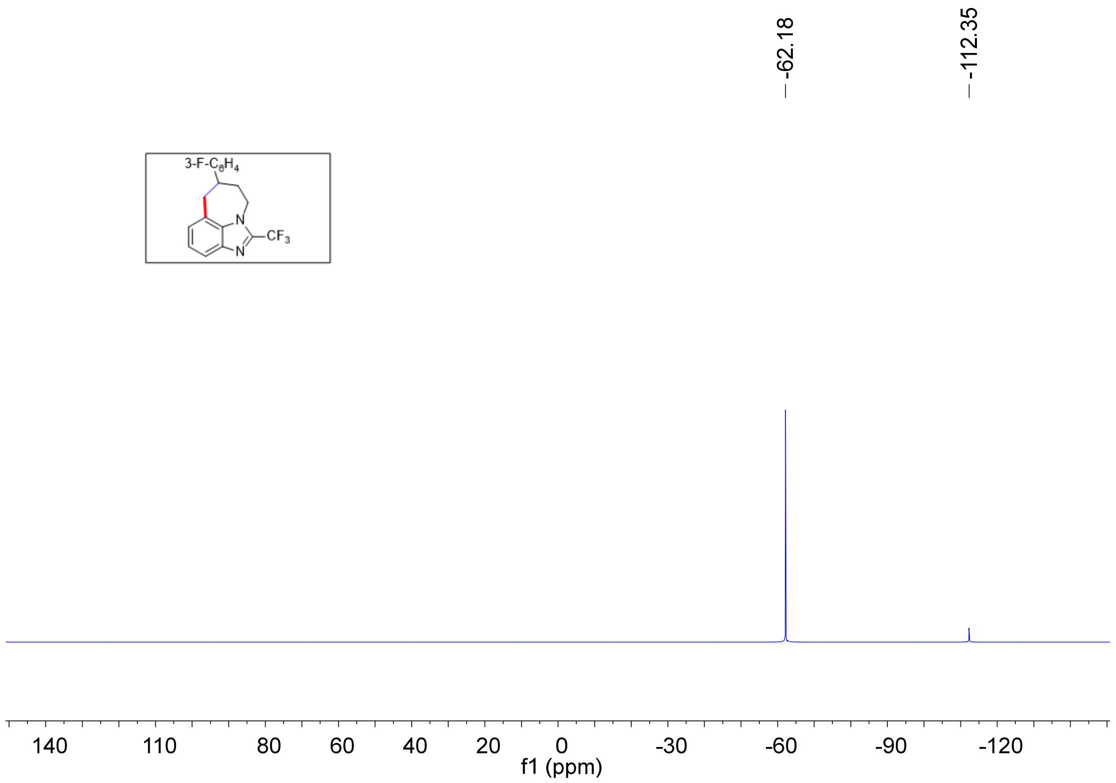

Supplementary Figure 140. ^{19}F NMR spectrum of compound **3o** in $CDCl_3$.

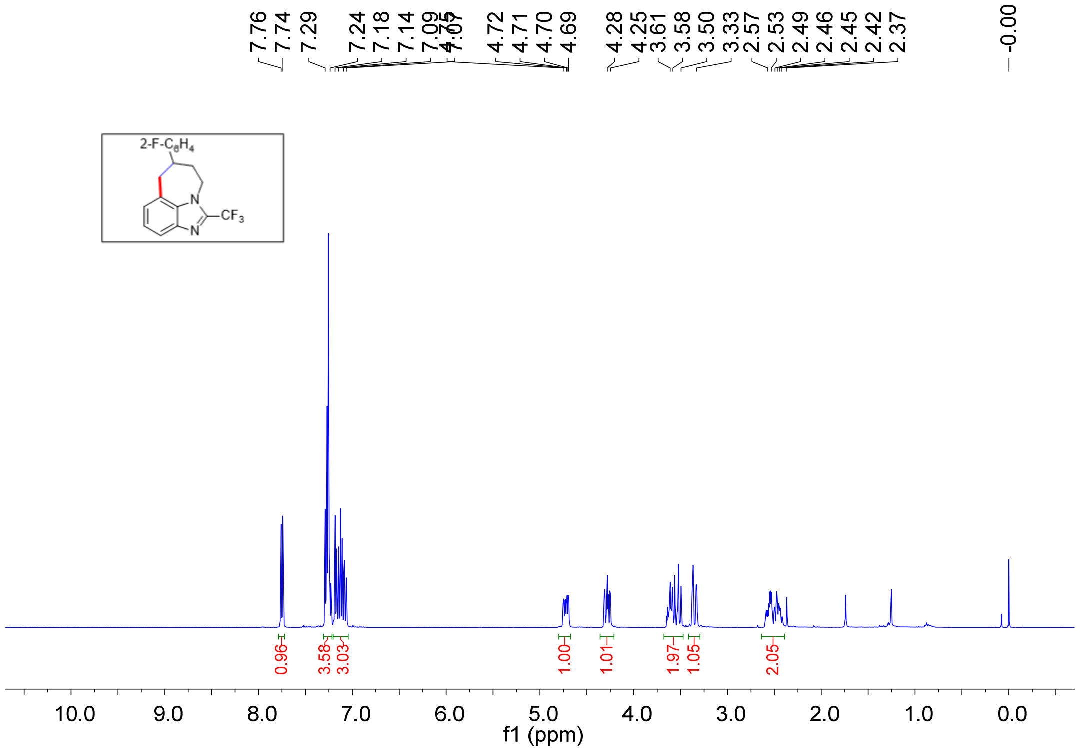

Supplementary Figure 141. ^{1}H NMR spectrum of compound **3p** in $CDCl_3$.

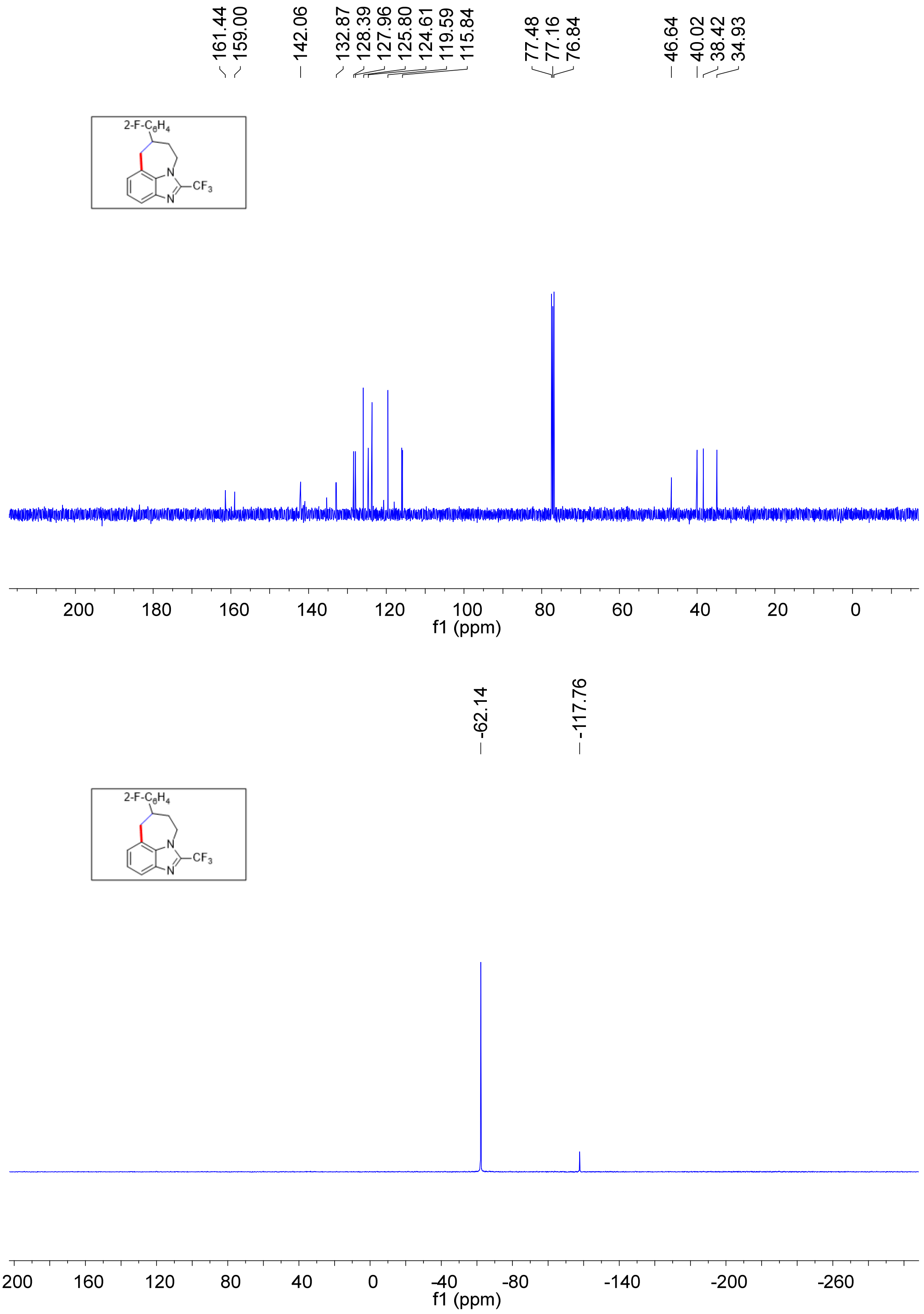

Supplementary Figure 142. ^{13}C and ^{19}F NMR spectra of compound **3p** in $CDCl_3$.

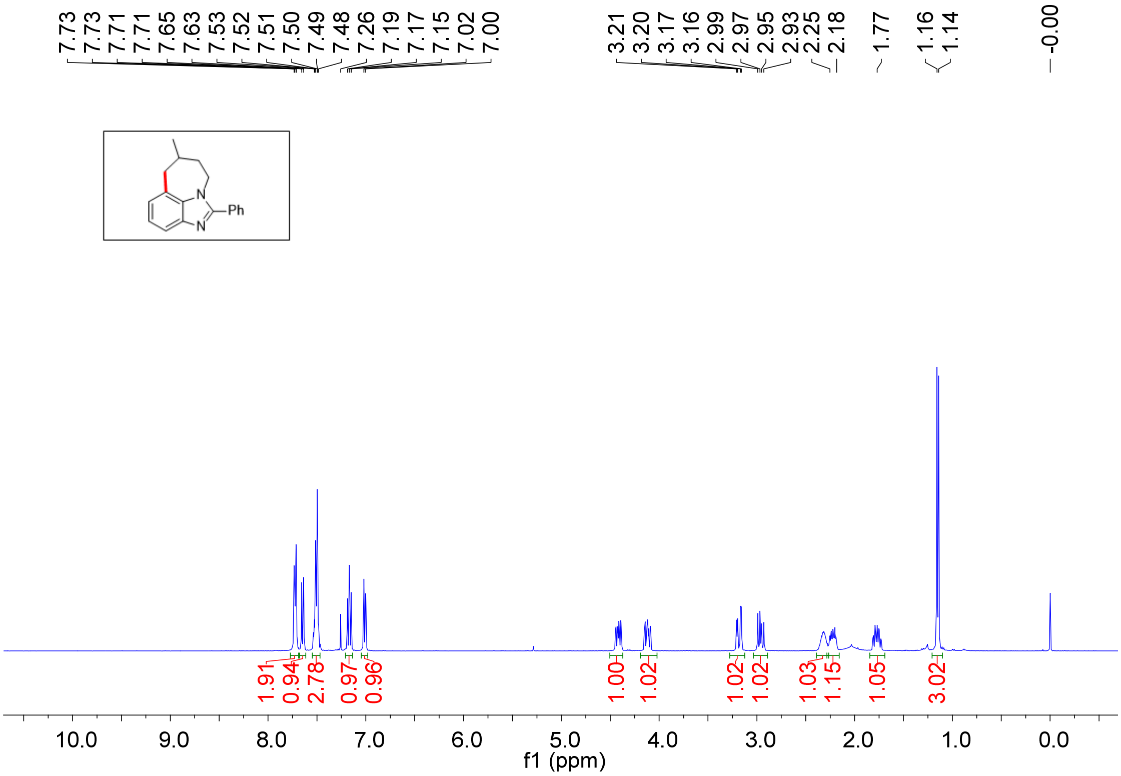

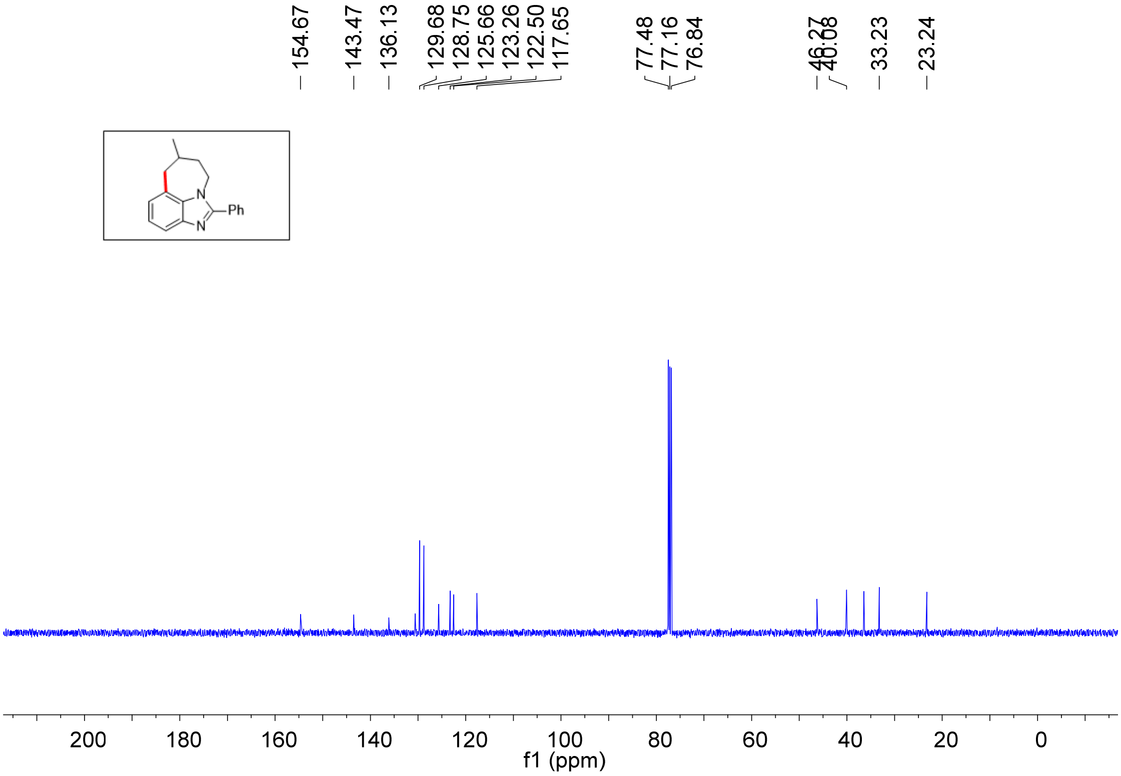

Supplementary Figure 143. ^{1}H and ^{13}C NMR spectra of compound **3r** in $CDCl_3$.

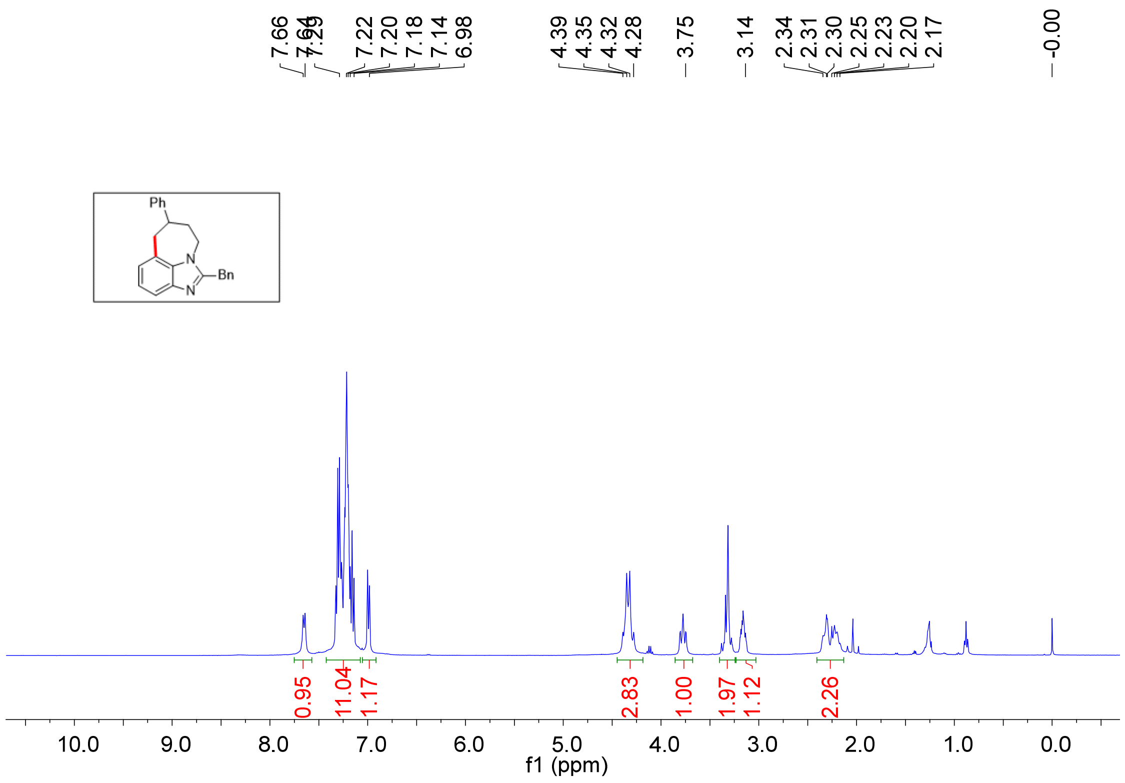

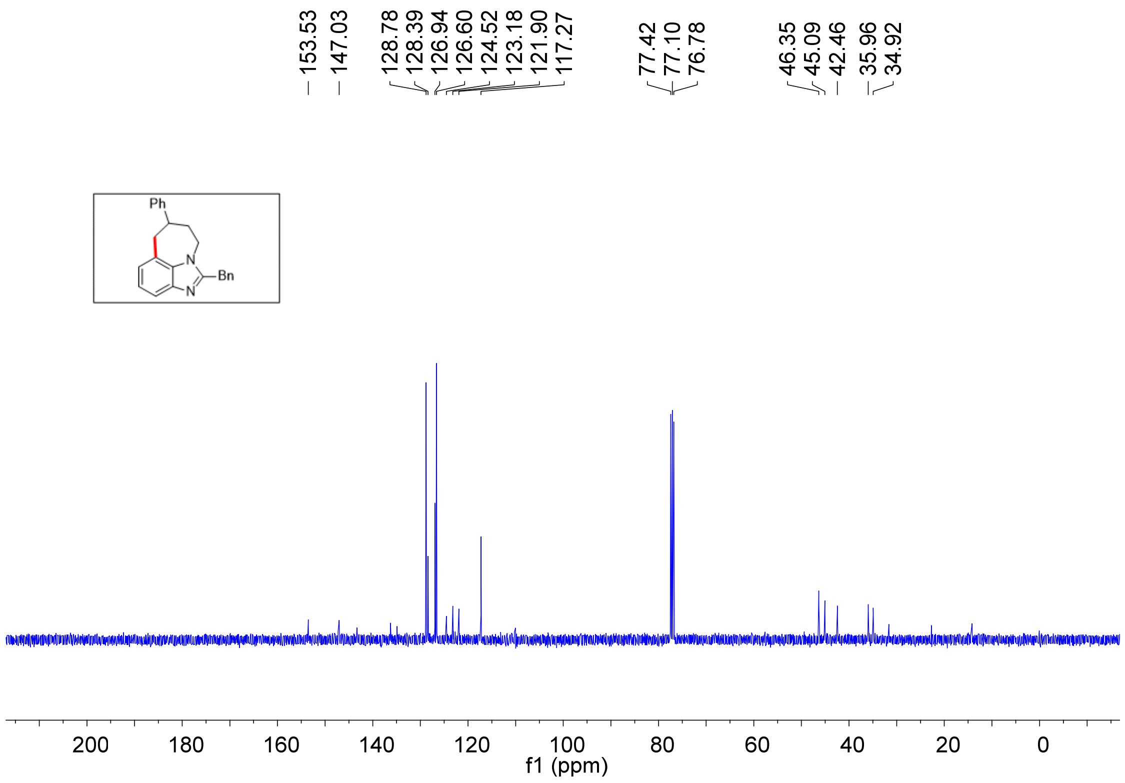

Supplementary Figure 144. ^{1}H and ^{13}C NMR spectra of compound **3s** in $CDCl_3$.

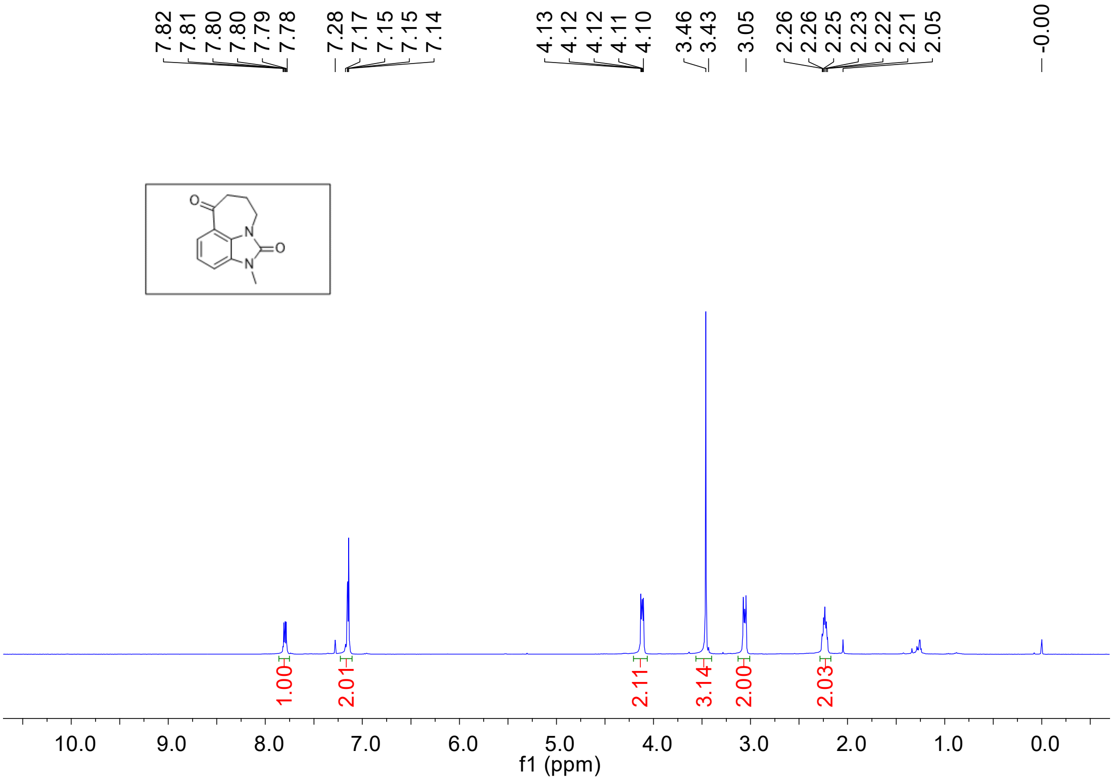

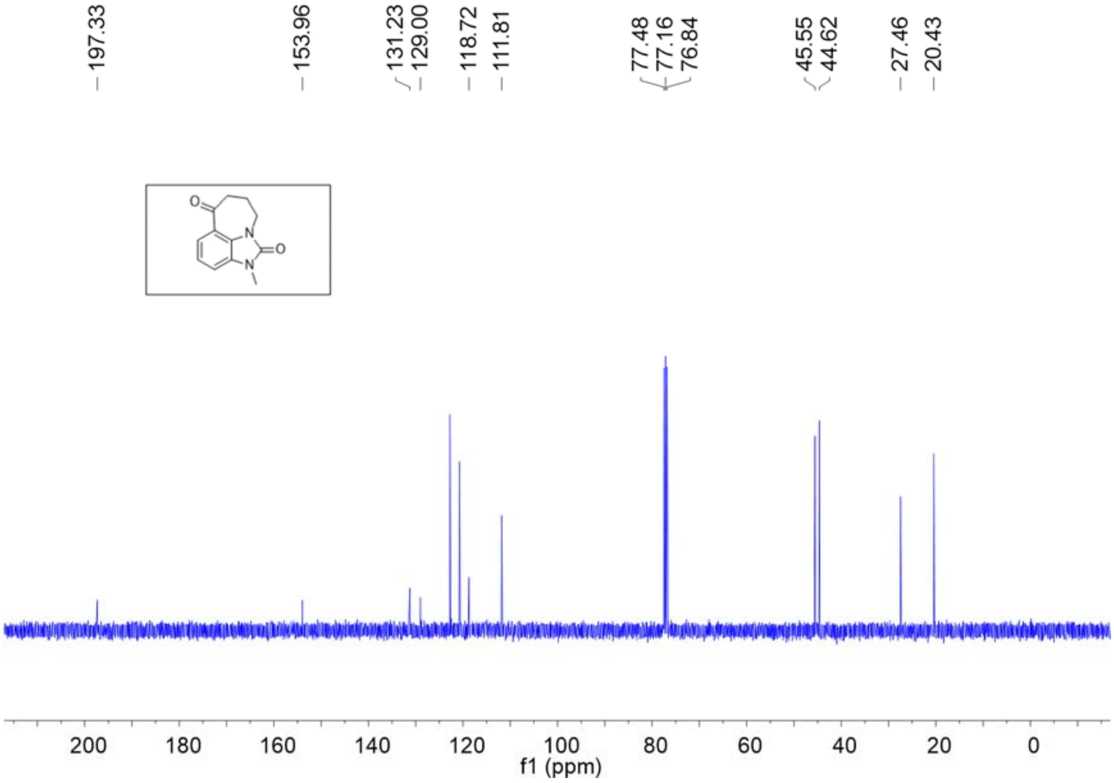

Supplementary Figure 145. ^{1}H and ^{13}C NMR spectra of compound **4** in $CDCl_3$

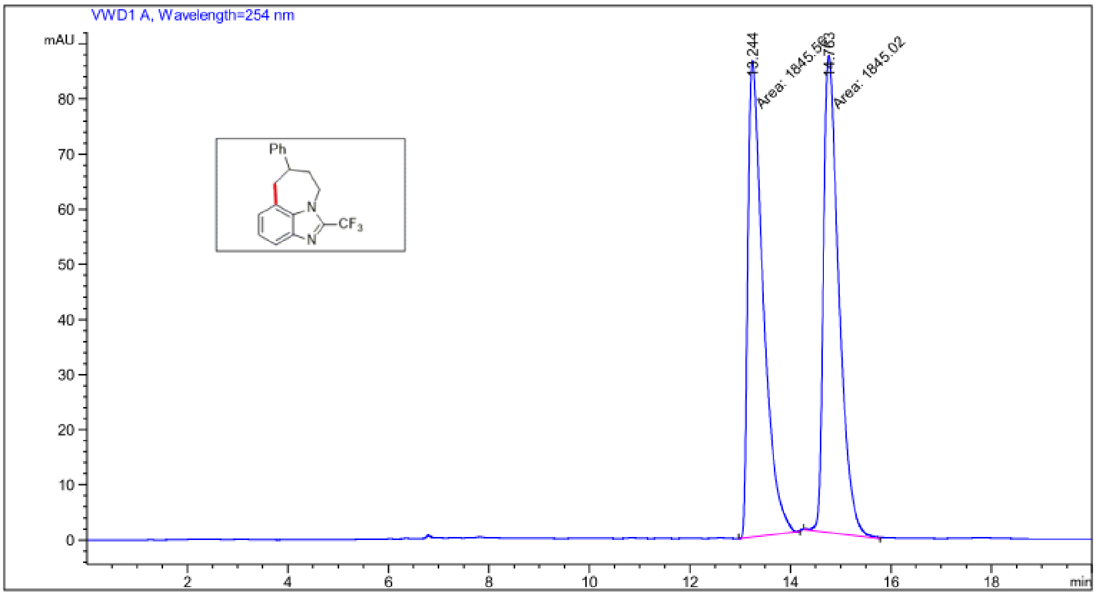

Peak #	RetTime [min]	Type	Width [min]	Area [mAU*s]	Height [mAU]	Area %
1	13.244	MM	0.3559	1845.56396	86.42306	50.0074
2	14.763	MM	0.3555	1845.02014	86.49442	49.9926

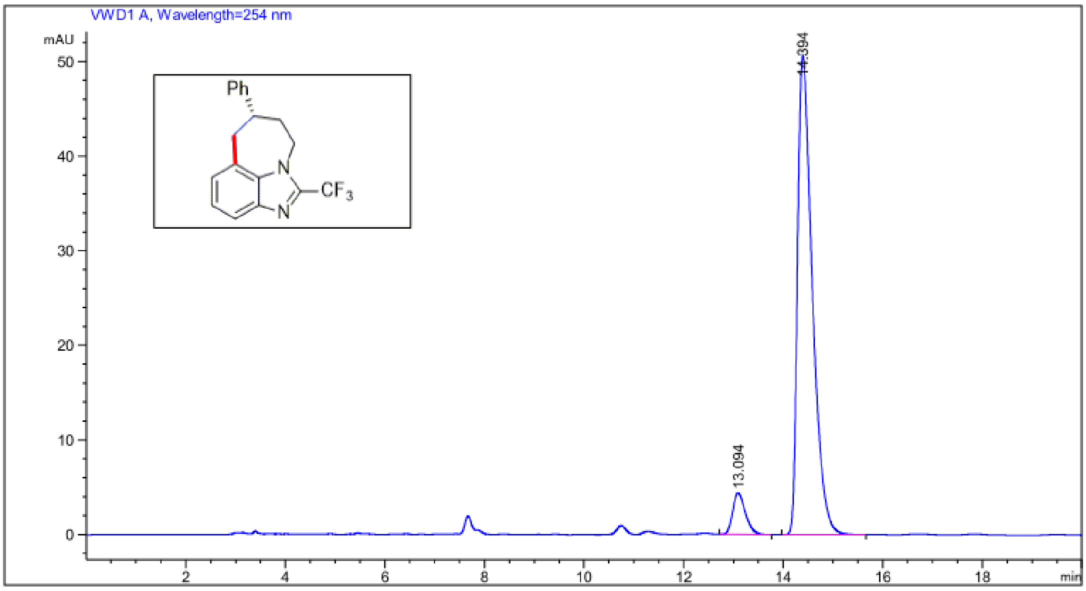

Peak #	RetTime [min]	Type	Width [min]	Area [mAU*s]	Height [mAU]	Area %
1	13.094	BB	0.2761	78.02151	4.37478	6.9723
2	14.394	BB	0.3127	1040.99158	50.67624	93.0277

Supplementary Figure 146. HPLC spectra for racemic and chiral **3e**

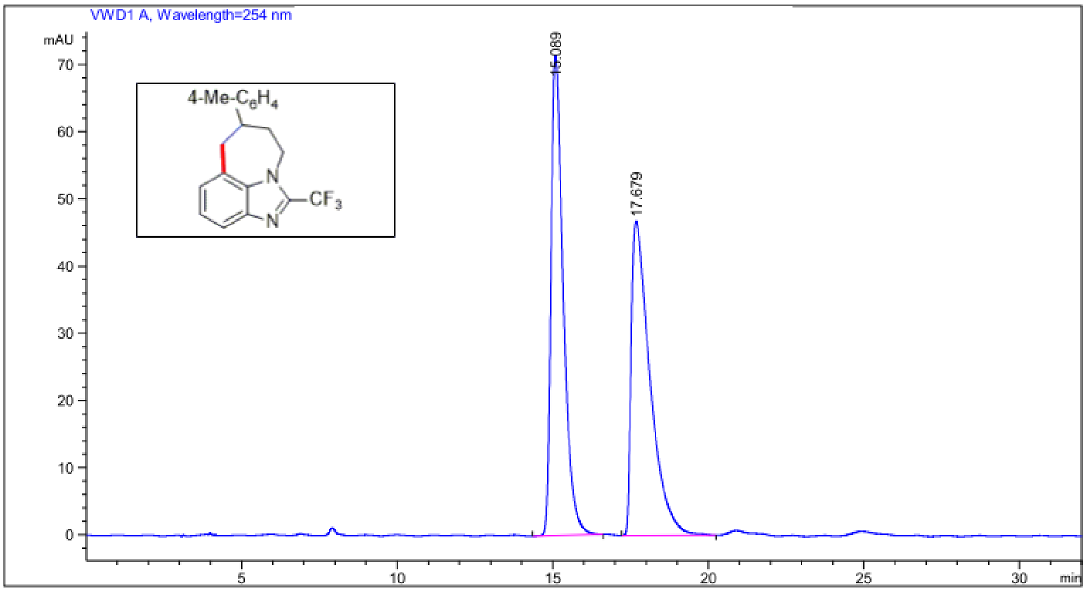

Peak #	RetTime [min]	Type	Width [min]	Area [mAU*s]	Height [mAU]	Area %
1	15.089	BB	0.4145	1954.80847	71.38284	49.9035
2	17.679	BB	0.6174	1962.36743	46.77768	50.0965

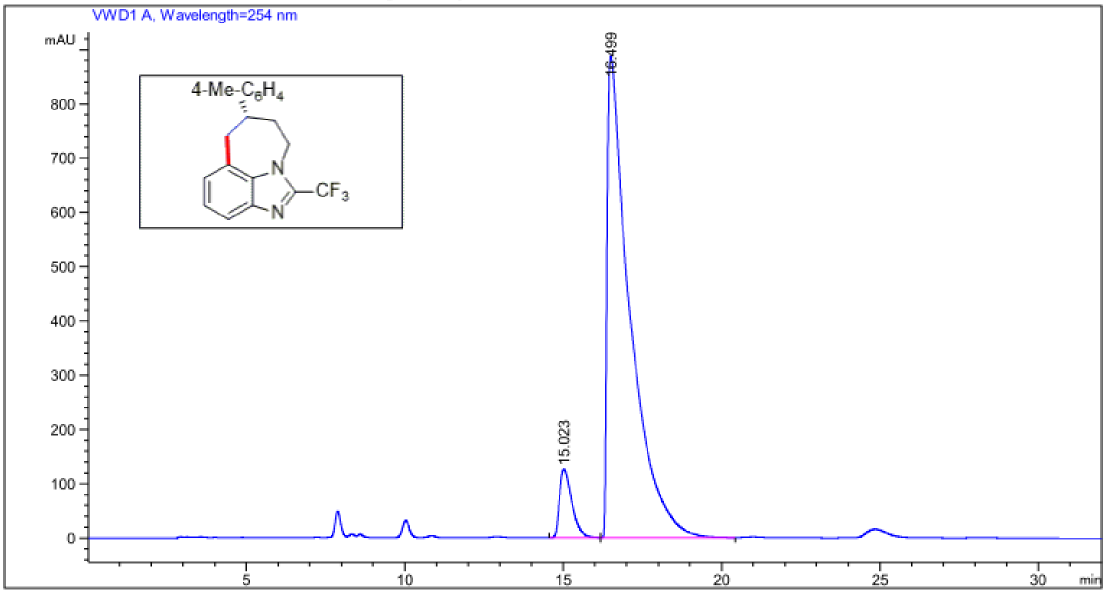

Peak #	RetTime [min]	Type	Width [min]	Area [mAU*s]	Height [mAU]	Area %
1	15.023	BB	0.4175	3429.17480	126.42043	7.1570
2	16.499	BB	0.6887	4.44846e4	887.66357	92.8430

Supplementary Figure 147. HPLC spectra for racemic and chiral **3f**

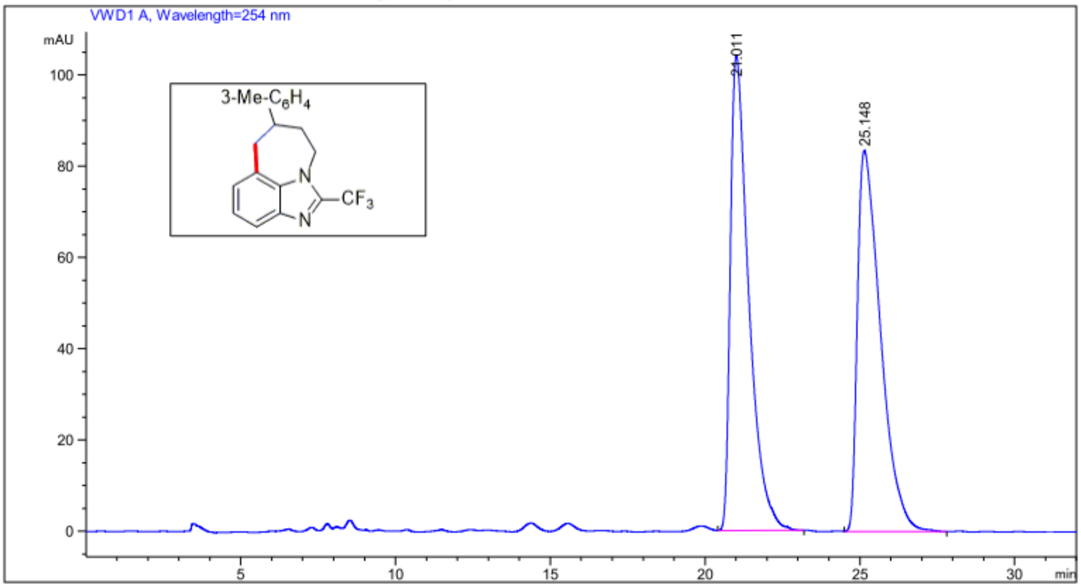

Peak #	RetTime [min]	Type	Width [min]	Area [mAU*s]	Height [mAU]	Area %
1	21.011	BV R	0.6133	4285.27100	104.08074	49.8362
2	25.148	BB	0.8058	4313.43457	83.47752	50.1638

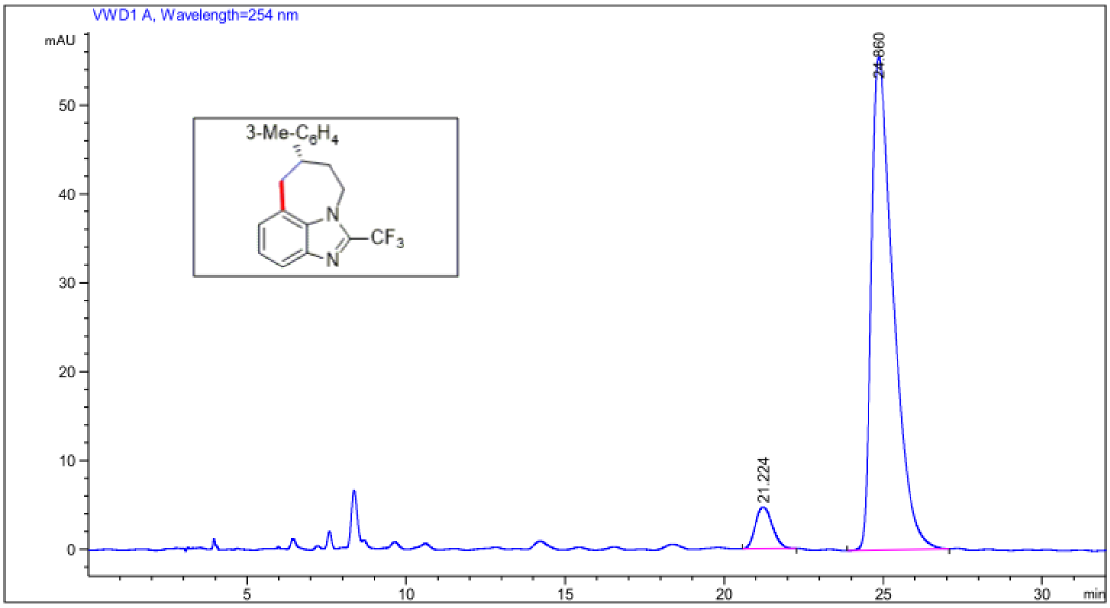

Peak #	RetTime [min]	Type	Width [min]	Area [mAU*s]	Height [mAU]	Area %
1	21.224	BB	0.5796	174.96202	4.69682	6.1836
2	24.860	BB	0.7080	2654.49219	55.49596	93.8164

Supplementary Figure 148. HPLC spectra for racemic and chiral **3g**

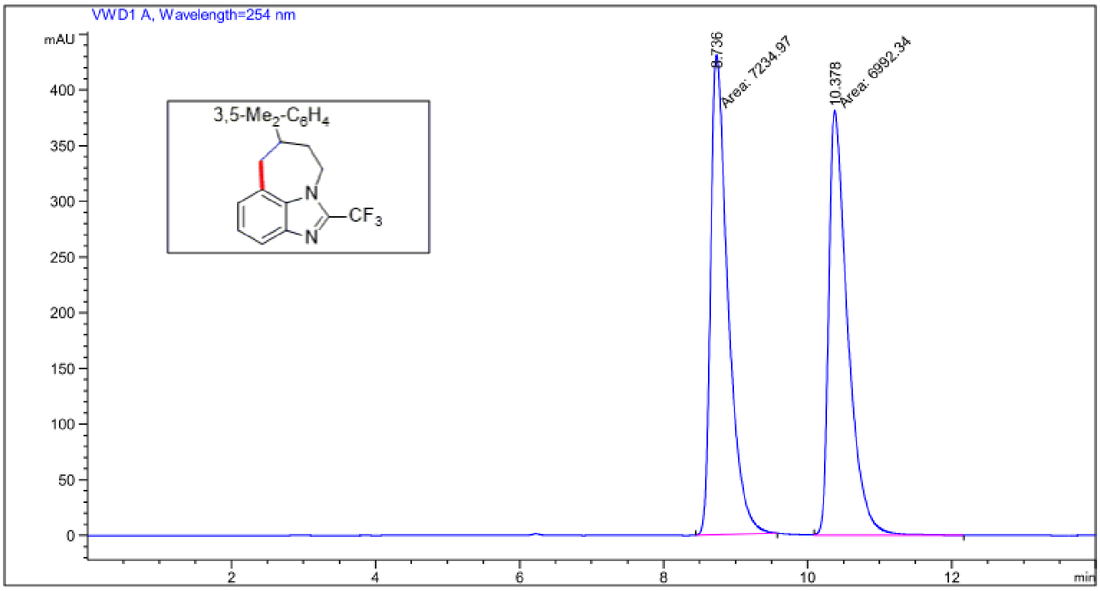

Peak #	RetTime [min]	Type	Width [min]	Area [mAU*s]	Height [mAU]	Area %
1	8.736	MM	0.2801	7234.97119	430.45563	50.8527
2	10.378	MM	0.3057	6992.33789	381.23758	49.1473

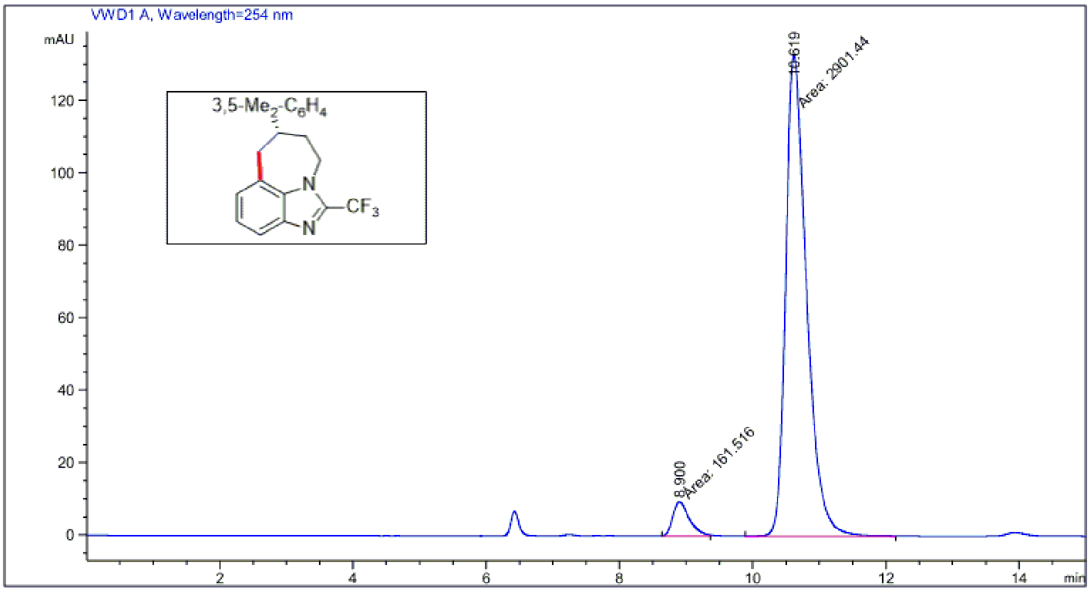

Peak #	RetTime [min]	Type	Width [min]	Area [mAU*s]	Height [mAU]	Area %
1	8.900	MM	0.2863	161.51624	9.40114	5.2732
2	10.619	MM	0.3639	2901.43652	132.88994	94.7268

Supplementary Figure 149. HPLC spectra for racemic and chiral **3h**

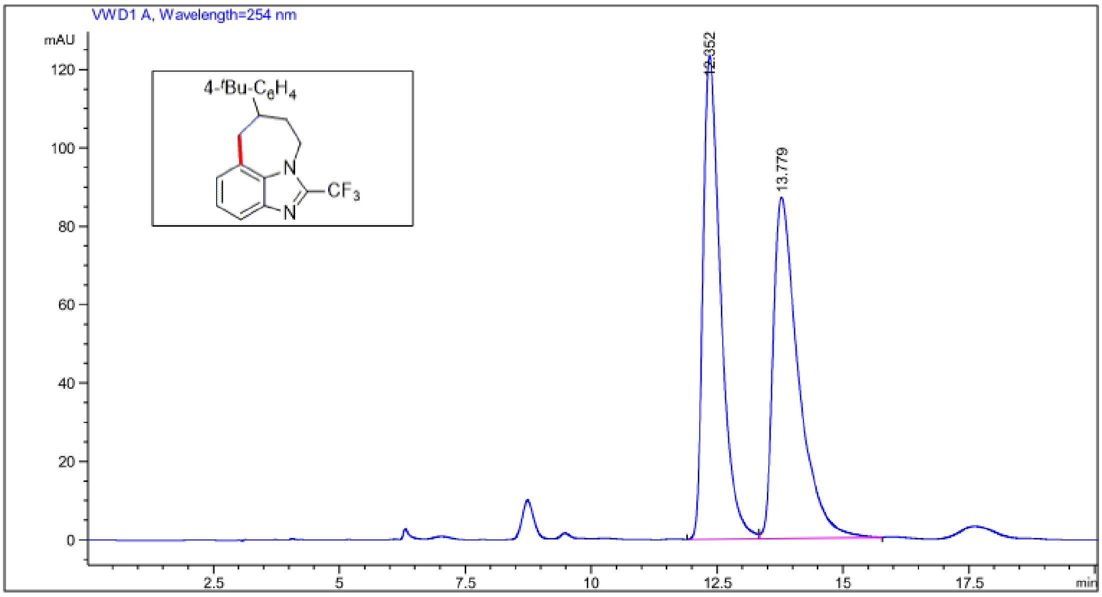

Peak #	RetTime [min]	Type	Width [min]	Area [mAU*s]	Height [mAU]	Area %
1	12.352	BV	0.3693	3013.77417	123.25938	49.8859
2	13.779	VB	0.5137	3027.56250	87.06028	50.1141

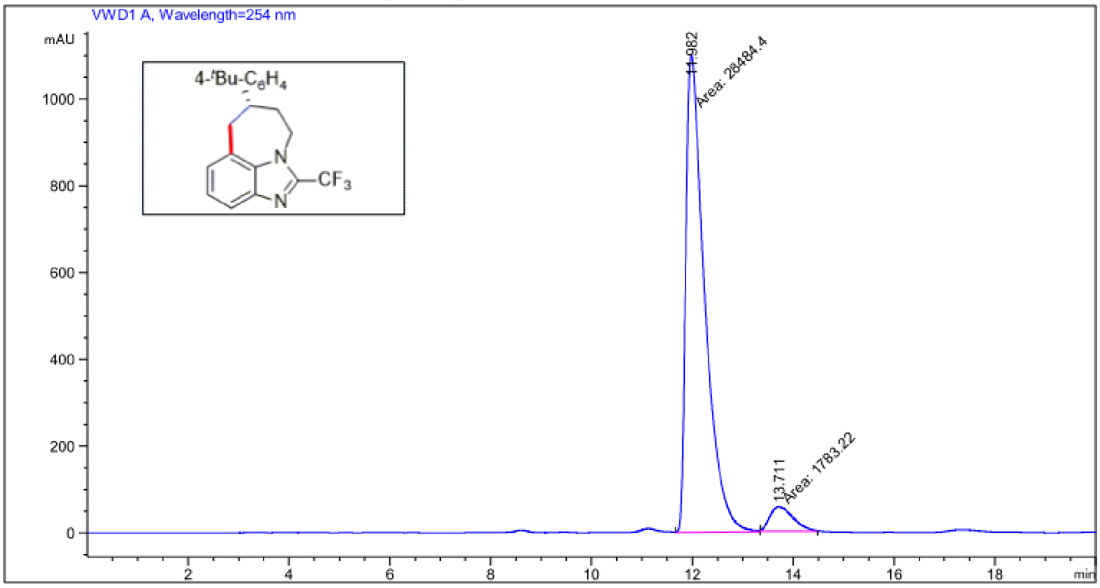

Peak #	RetTime [min]	Type	Width [min]	Area [mAU*s]	Height [mAU]	Area %
1	11.982	MM	0.4317	2.84844e4	1099.65588	94.1085
2	13.711	MM	0.5332	1783.21814	55.73542	5.8915

Supplementary Figure 150. HPLC spectra for racemic and chiral **3i**

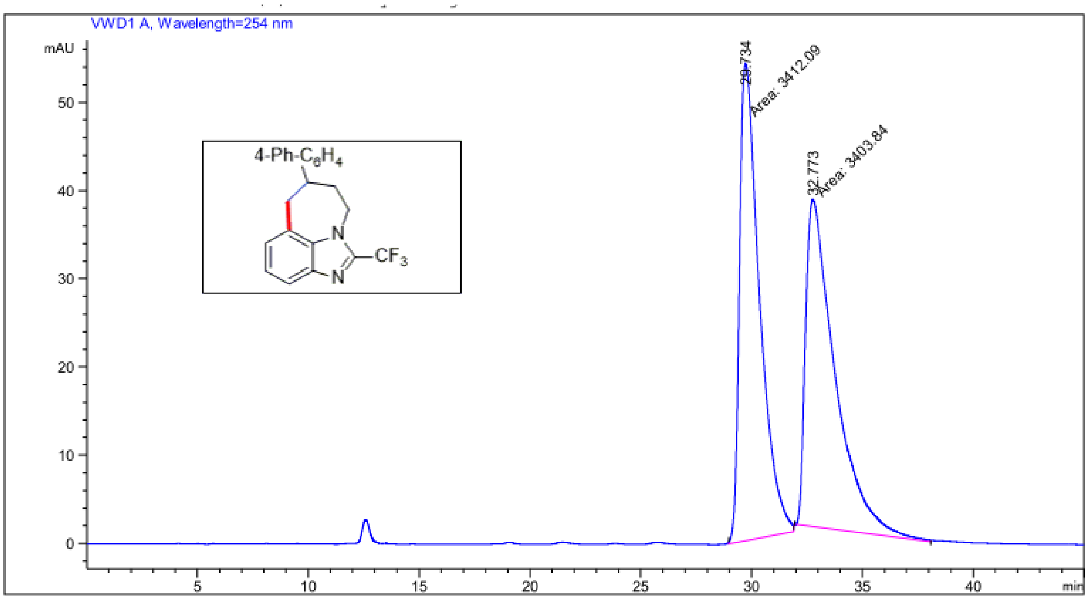

Peak #	RetTime [min]	Type	Width [min]	Area [mAU*s]	Height [mAU]	Area %
1	29.734	MM	1.0523	3412.08862	54.04036	50.0605
2	32.773	MM	1.5312	3403.83569	37.04925	49.9395

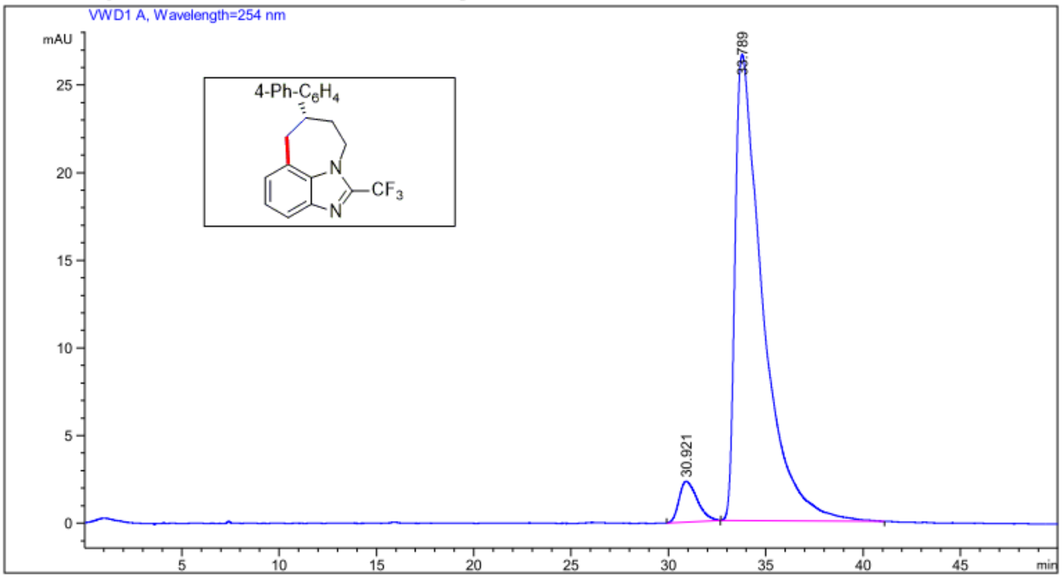

Peak #	RetTime [min]	Type	Width [min]	Area [mAU*s]	Height [mAU]	Area %
1	30.921	BB	0.7774	150.13560	2.32173	5.4215
2	33.789	BB	1.3499	2619.11279	26.57360	94.5785

Supplementary Figure 151. HPLC spectra for racemic and chiral **3j**

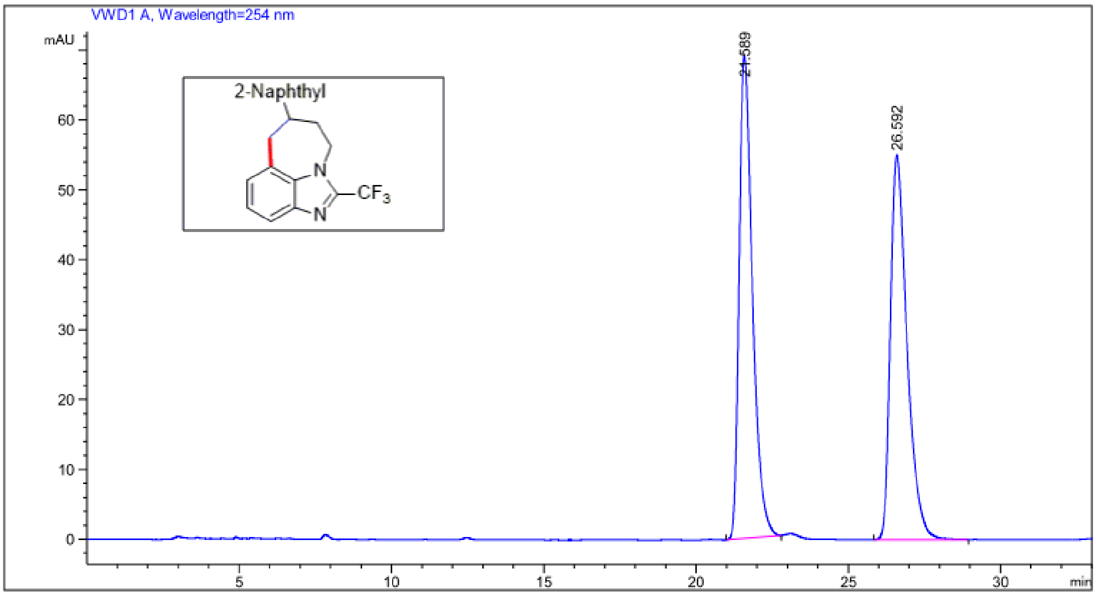

Peak #	RetTime [min]	Type	Width [min]	Area [mAU*s]	Height [mAU]	Area %
1	21.589	BB	0.4628	2100.35645	69.06347	50.2211
2	26.592	BB	0.5711	2081.85962	55.06849	49.7789

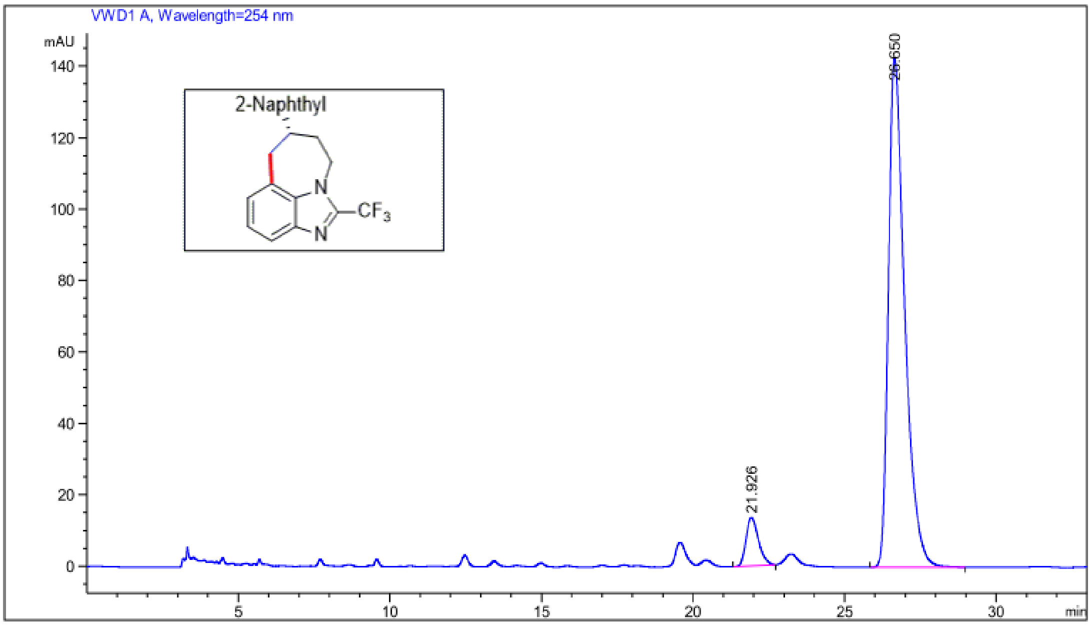

Peak #	RetTime [min]	Type	Width [min]	Area [mAU*s]	Height [mAU]	Area %
1	21.926	BB	0.4407	386.06647	13.45845	6.7703
2	26.650	BB	0.5435	5316.29785	142.40459	93.2297

Supplementary Figure 152. HPLC spectra for racemic and chiral **3l**

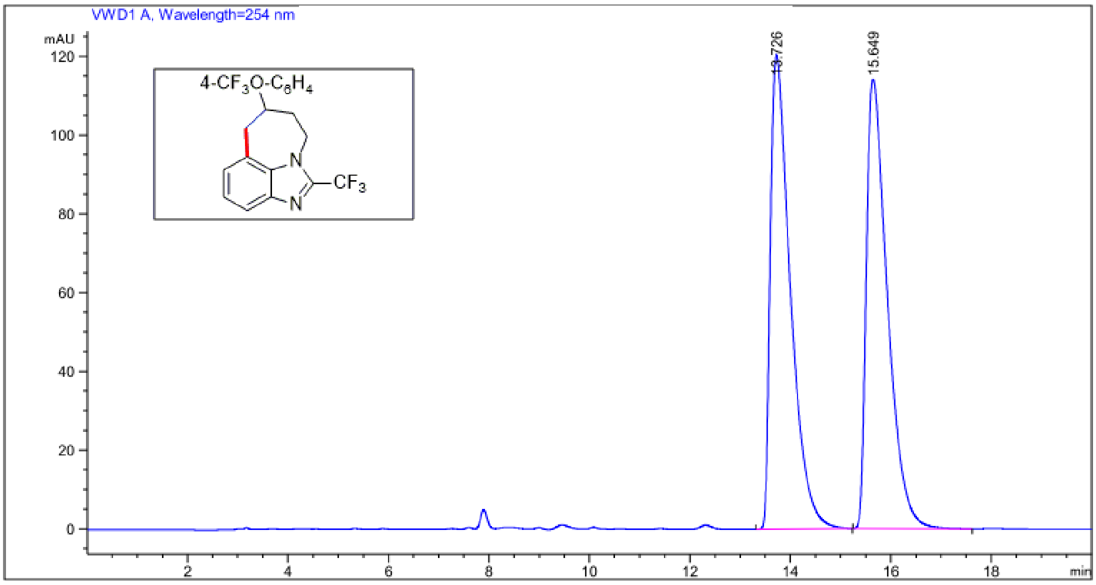

Peak #	RetTime [min]	Type	Width [min]	Area [mAU*s]	Height [mAU]	Area %
1	13.726	BB	0.4151	3337.86548	120.51654	50.1389
2	15.649	BB	0.4413	3319.37427	114.14256	49.8611

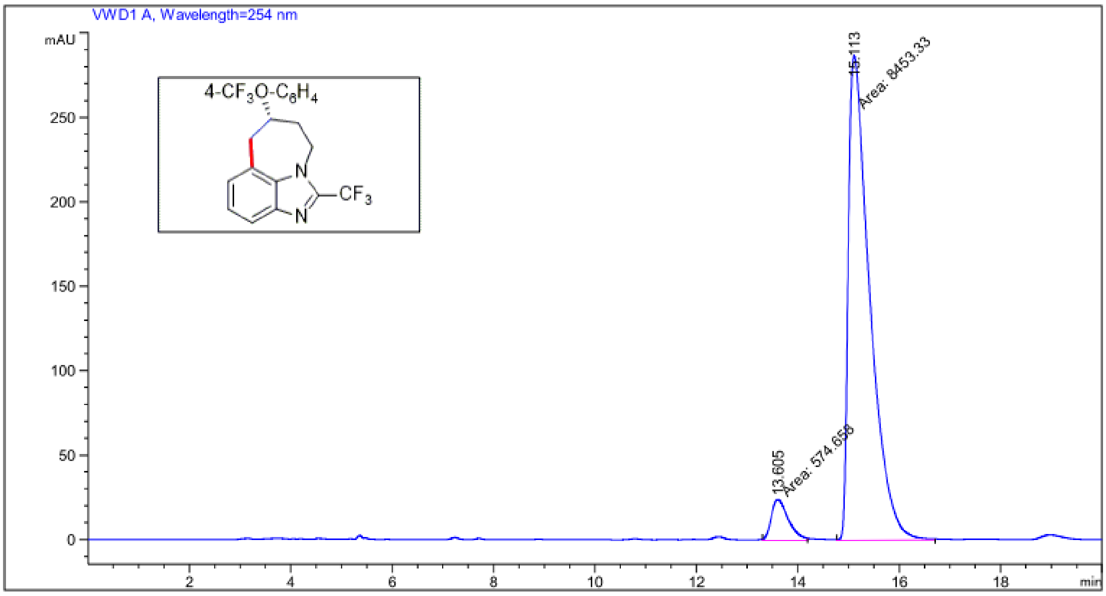

Peak #	RetTime [min]	Type	Width [min]	Area [mAU*s]	Height [mAU]	Area %
1	13.605	MM	0.3941	574.65839	24.30049	6.3653
2	15.113	MM	0.4908	8453.32715	287.08102	93.6347

Supplementary Figure 153. HPLC spectra for racemic and chiral **3m**

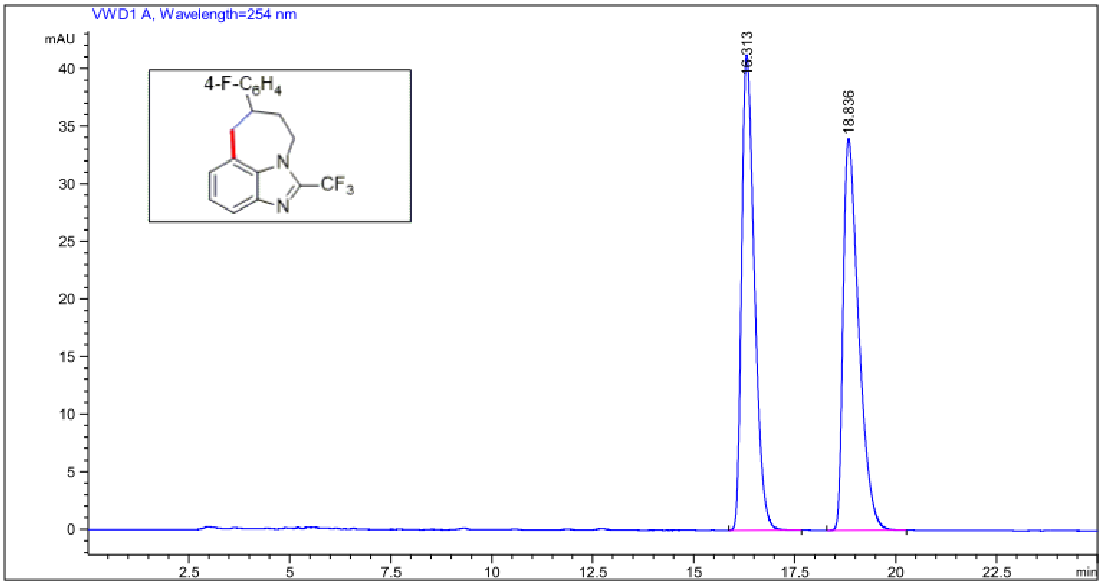

Peak #	RetTime [min]	Type	Width [min]	Area [mAU*s]	Height [mAU]	Area %
1	16.313	BB	0.3407	908.42725	41.28627	49.9682
2	18.836	BB	0.4033	909.58392	33.98219	50.0318

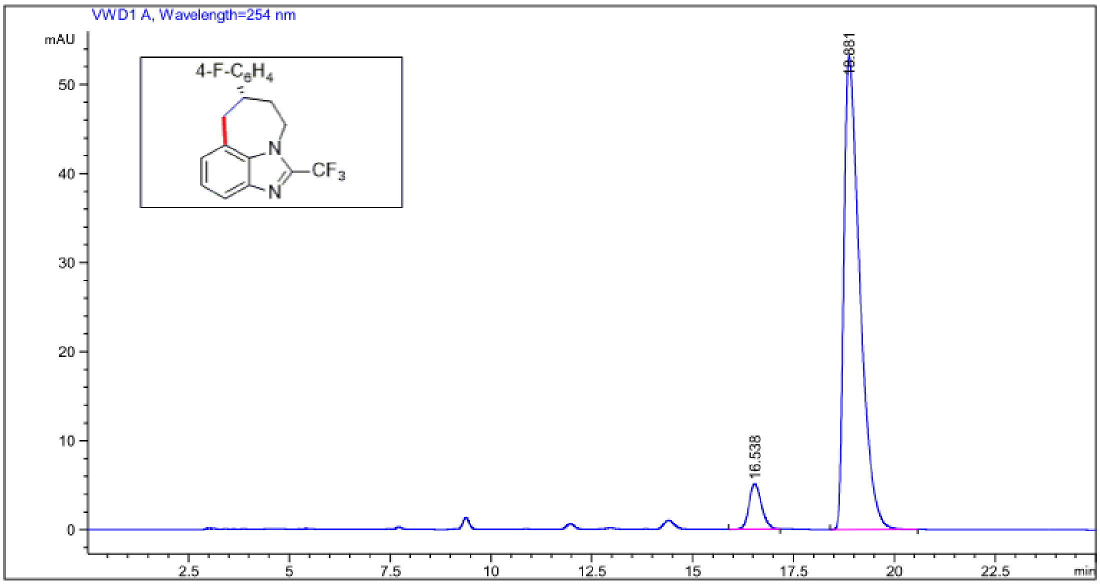

Peak #	RetTime [min]	Type	Width [min]	Area [mAU*s]	Height [mAU]	Area %
1	16.538	BB	0.3320	108.95206	5.08505	6.9264
2	18.881	BB	0.4146	1464.05469	53.27144	93.0736

Supplementary Figure 154. HPLC spectra for racemic and chiral **3n**

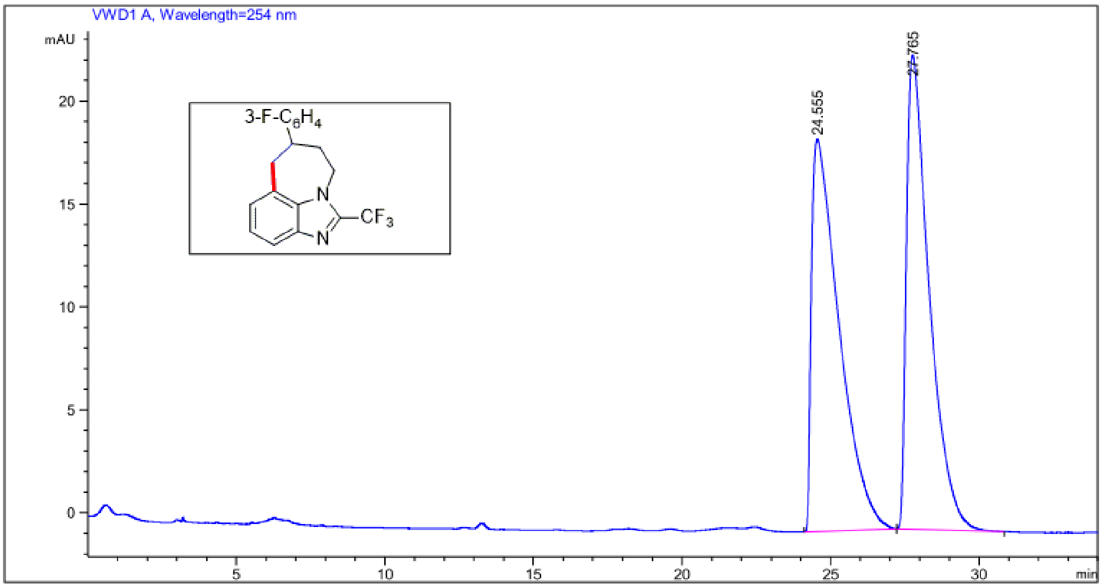

Peak #	RetTime [min]	Type	Width [min]	Area [mAU*s]	Height [mAU]	Area %
1	24.555	BB	0.8870	1230.77539	19.06970	50.0667
2	27.765	BB	0.7757	1227.49805	23.03379	49.9333

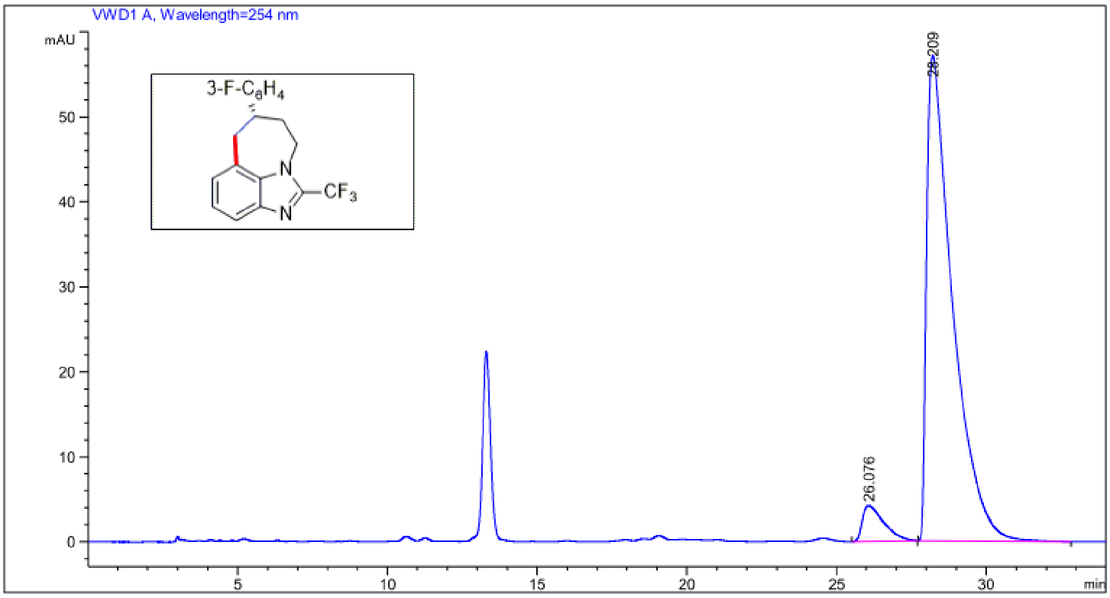

Peak #	RetTime [min]	Type	Width [min]	Area [mAU*s]	Height [mAU]	Area %
1	26.076	BB	0.7184	208.61751	4.19348	5.7400
2	28.209	BB	0.8708	3425.80688	57.03177	94.2600

Supplementary Figure 155. HPLC spectra for racemic and chiral **3o**

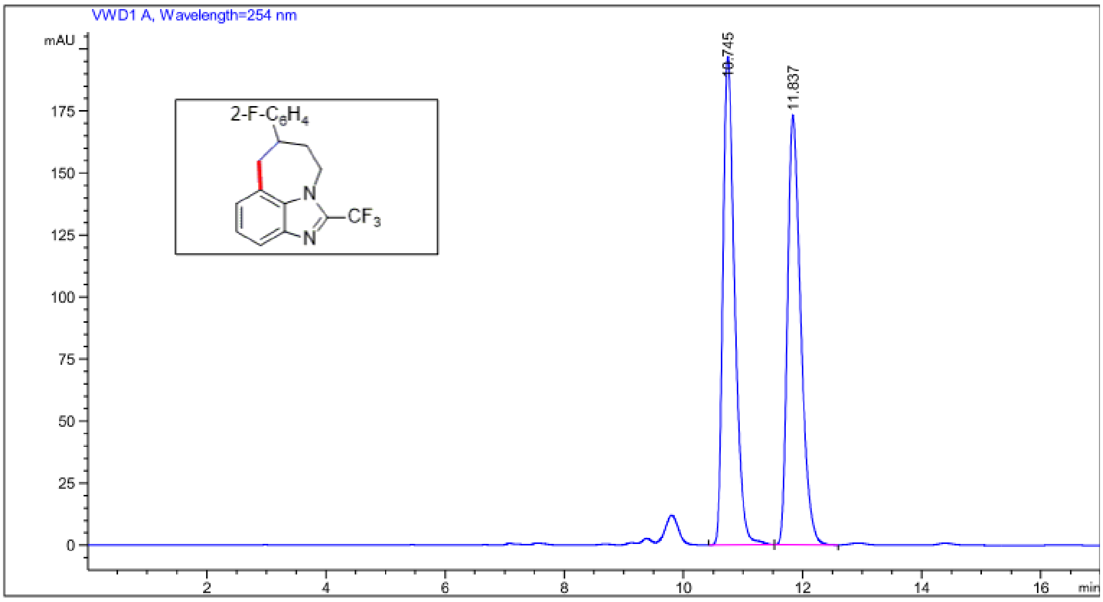

Peak #	RetTime [min]	Type	Width [min]	Area [mAU*s]	Height [mAU]	Area %
1	10.745	BB	0.2100	2701.43579	196.93842	50.1962
2	11.837	BB	0.2386	2680.31909	173.17233	49.8038

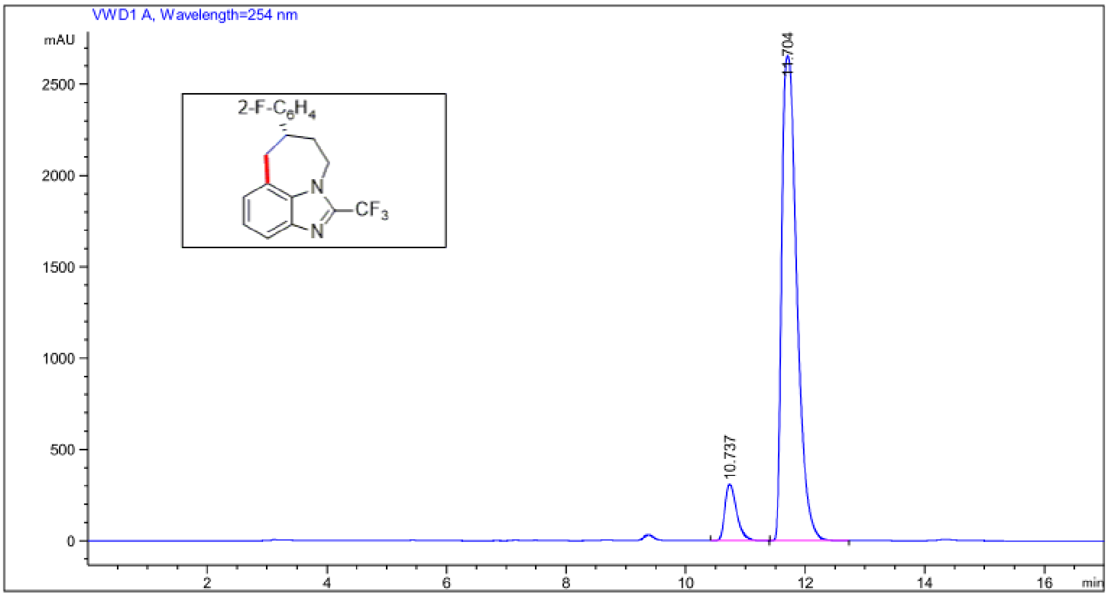

Peak #	RetTime [min]	Type	Width [min]	Area [mAU*s]	Height [mAU]	Area %
1	10.737	BB	0.2089	4207.35254	308.88367	8.2976
2	11.704	BB	0.2723	4.64983e4	2655.19531	91.7024

Supplementary Figure 156. HPLC spectra for racemic and chiral **3p**

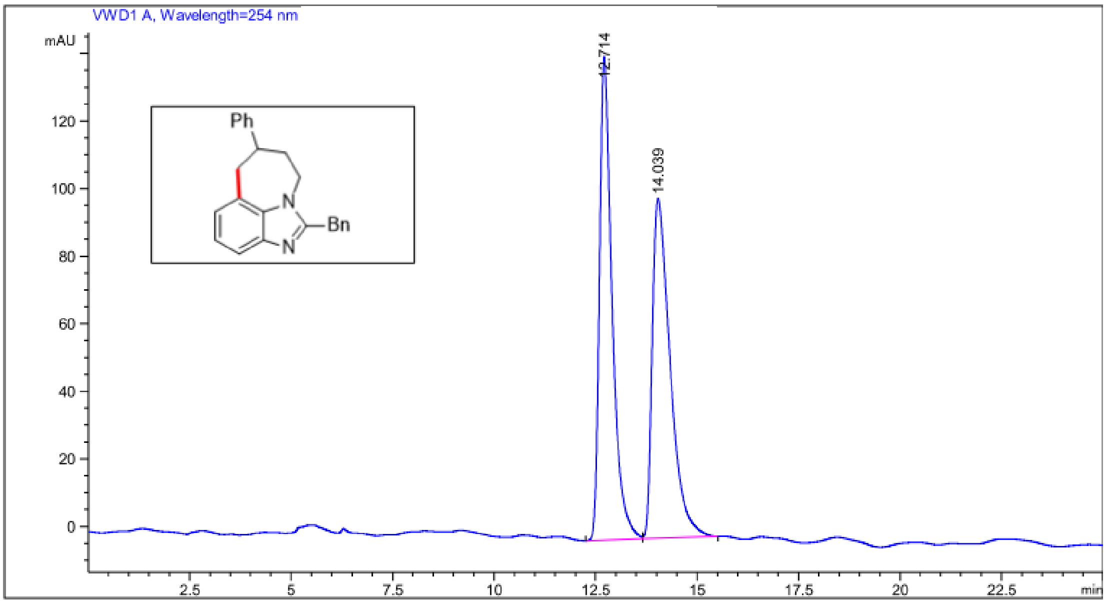

Peak #	RetTime [min]	Type	Width [min]	Area [mAU*s]	Height [mAU]	Area %
1	12.714	BV	0.3259	3101.64282	143.08839	50.1538
2	14.039	VB	0.4631	3082.61670	100.71576	49.8462

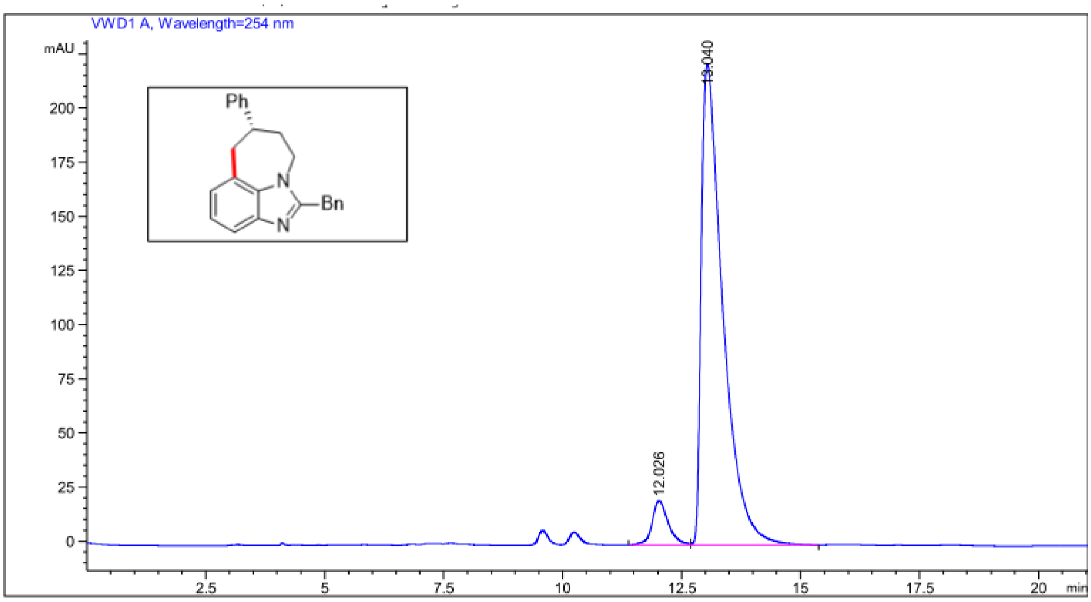

Peak #	RetTime [min]	Type	Width [min]	Area [mAU*s]	Height [mAU]	Area %
1	12.026	BV	0.3532	482.46353	20.37836	6.4640
2	13.040	VB	0.4660	6981.38916	221.93439	93.5360

Supplementary Figure 157. HPLC spectra for racemic and chiral **3q**

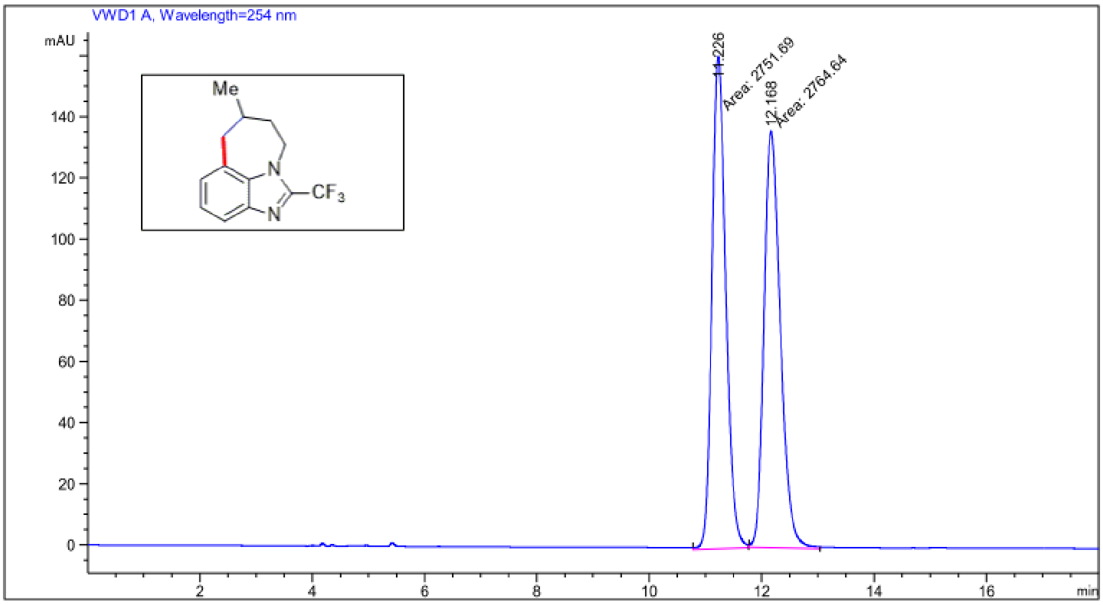

Peak #	RetTime [min]	Type	Width [min]	Area [mAU*s]	Height [mAU]	Area %
1	11.226	MM	0.2851	2751.68506	160.84126	49.8826
2	12.168	MM	0.3385	2764.63599	136.11655	50.1174

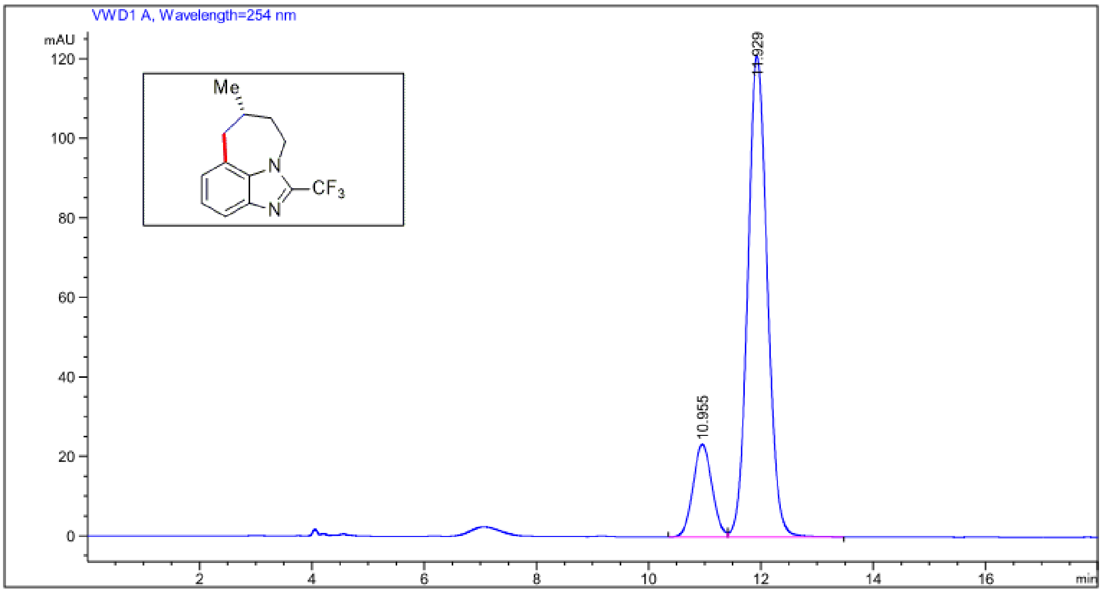

Peak #	RetTime [min]	Type	Width [min]	Area [mAU*s]	Height [mAU]	Area %
1	10.955	BV	0.3701	544.99219	23.19634	15.7439
2	11.929	VB	0.3723	2916.61060	120.95084	84.2561

Supplementary Figure 158. HPLC spectra for racemic and chiral **3a**

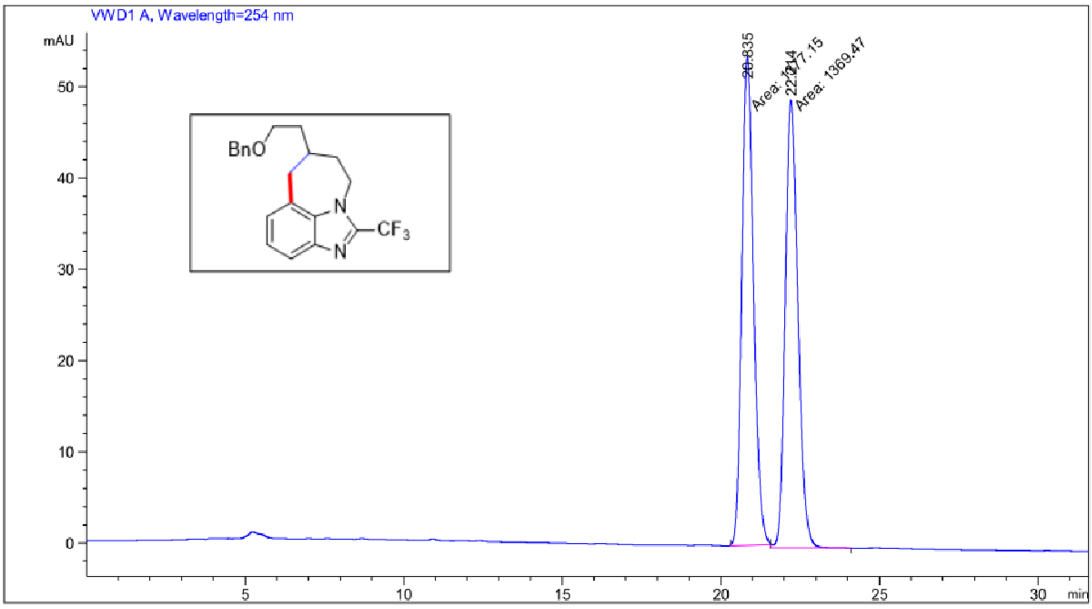

Peak #	RetTime [min]	Type	Width [min]	Area [mAU*s]	Height [mAU]	Area %
1	20.835	MM	0.4286	1377.14661	53.55211	50.1397
2	22.214	MM	0.4654	1369.47266	49.04802	49.8603

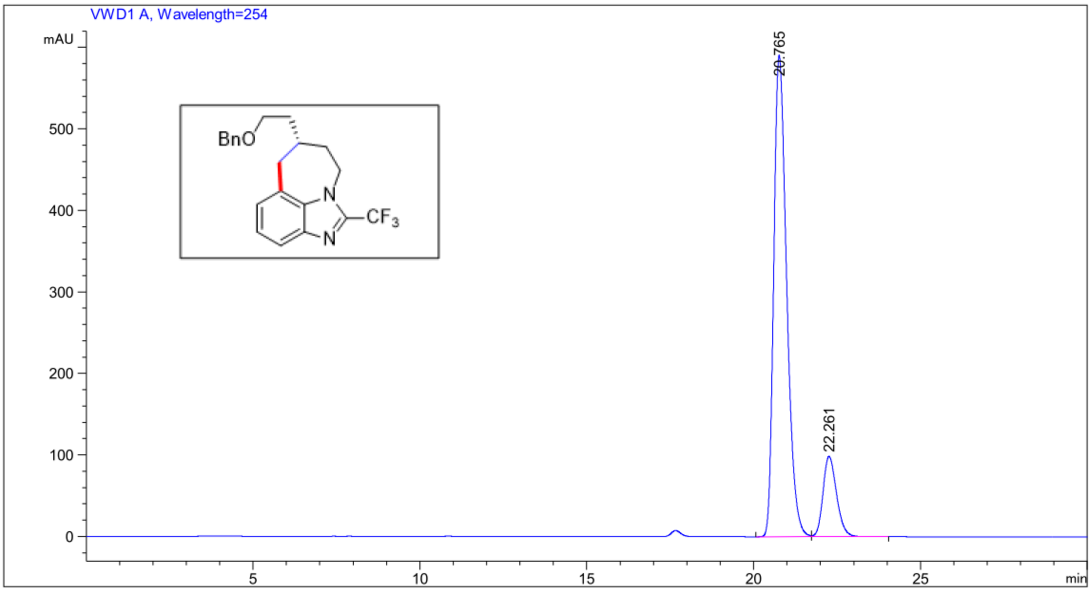

Peak #	RetTime [min]	Type	Width [min]	Area [mAU*s]	Height [mAU]	Area %
1	20.765	BV	0.4144	1.58227e4	590.90228	85.0285
2	22.261	VB	0.4378	2785.98999	98.56115	14.9715

Supplementary Figure 159. HPLC spectra for racemic and chiral **3d**

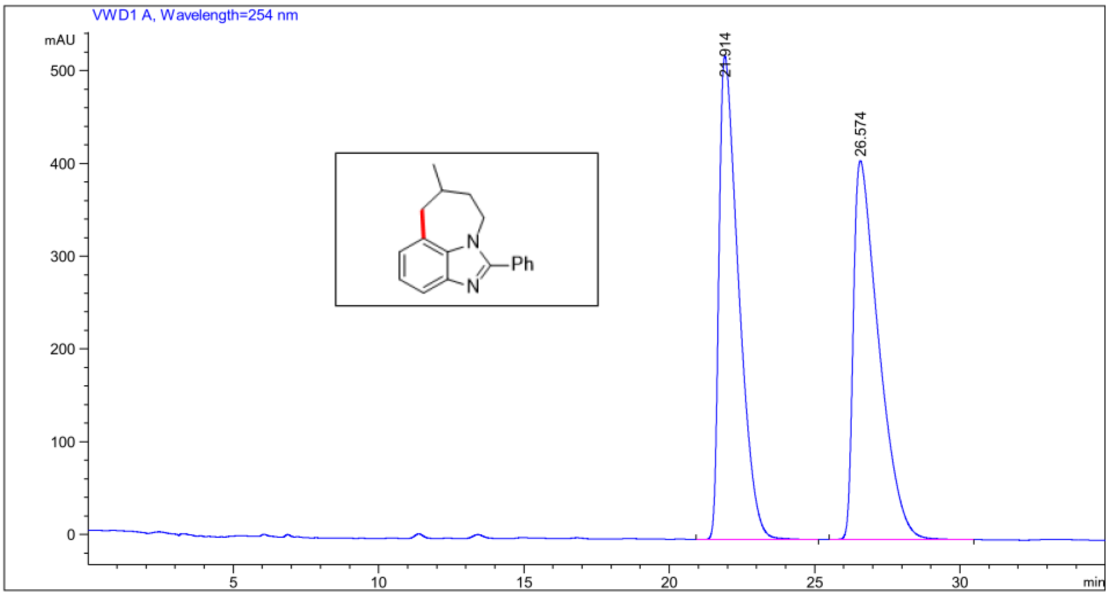

Peak #	RetTime [min]	Type	Width [min]	Area [mAU*s]	Height [mAU]	Area %
1	21.914	BB	0.7000	2.44851e4	521.23169	49.8076
2	26.574	BB	0.8891	2.46743e4	408.28601	50.1924

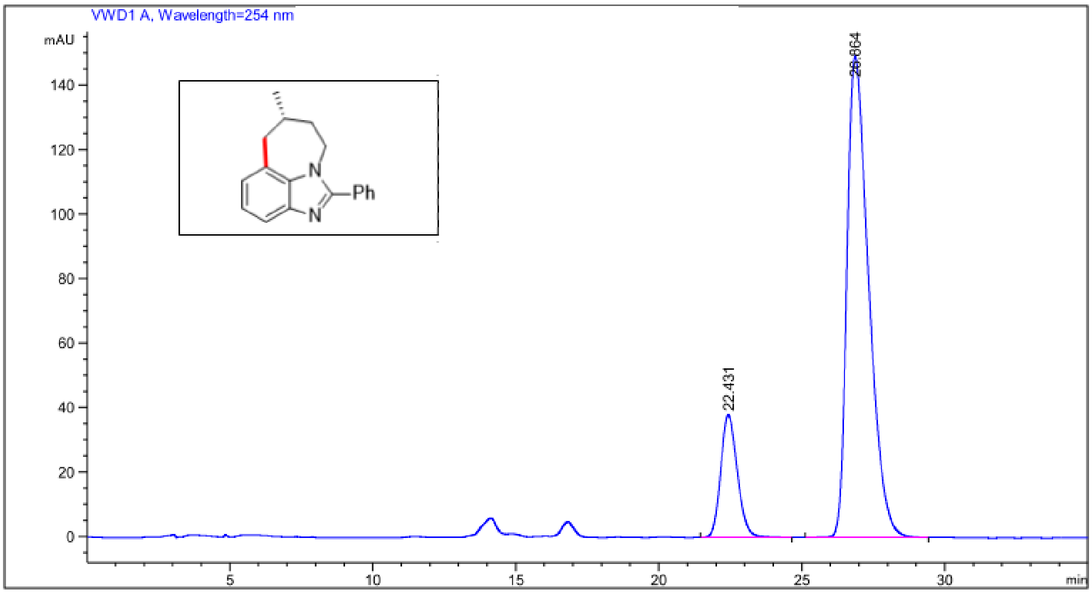

Peak #	RetTime [min]	Type	Width [min]	Area [mAU*s]	Height [mAU]	Area %
1	22.431	BB	0.6294	1545.38684	38.00102	16.4221
2	26.864	BB	0.8002	7865.03174	149.39319	83.5779

Supplementary Figure 160. HPLC spectra for racemic and chiral **3r**

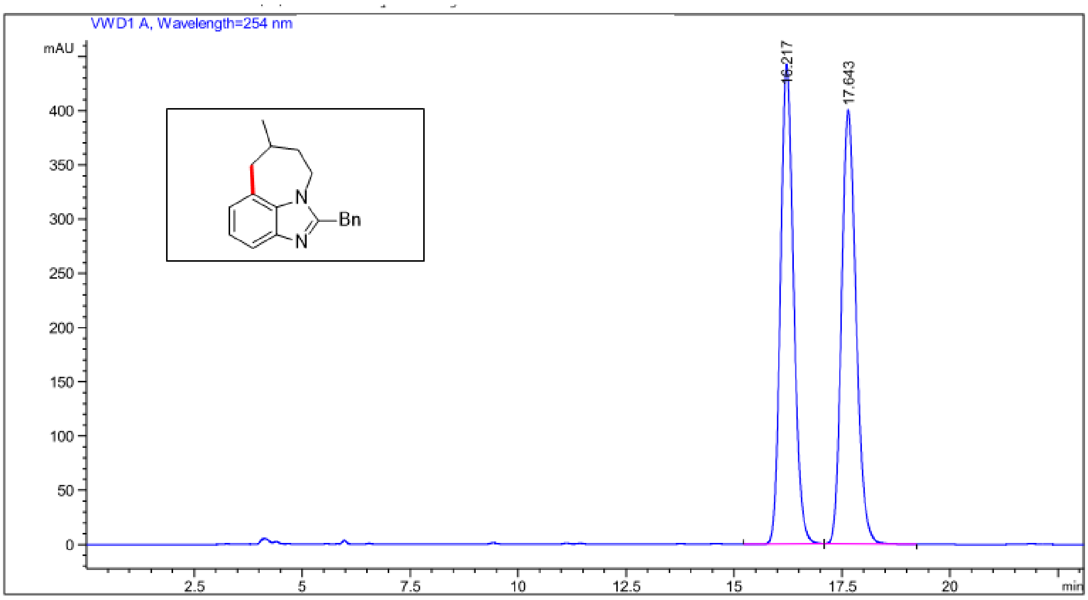

Peak #	RetTime [min]	Type	Width [min]	Area [mAU*s]	Height [mAU]	Area %
1	16.217	BB	0.3250	9334.26563	442.61957	49.9130
2	17.643	BB	0.3621	9366.78906	400.08405	50.0870

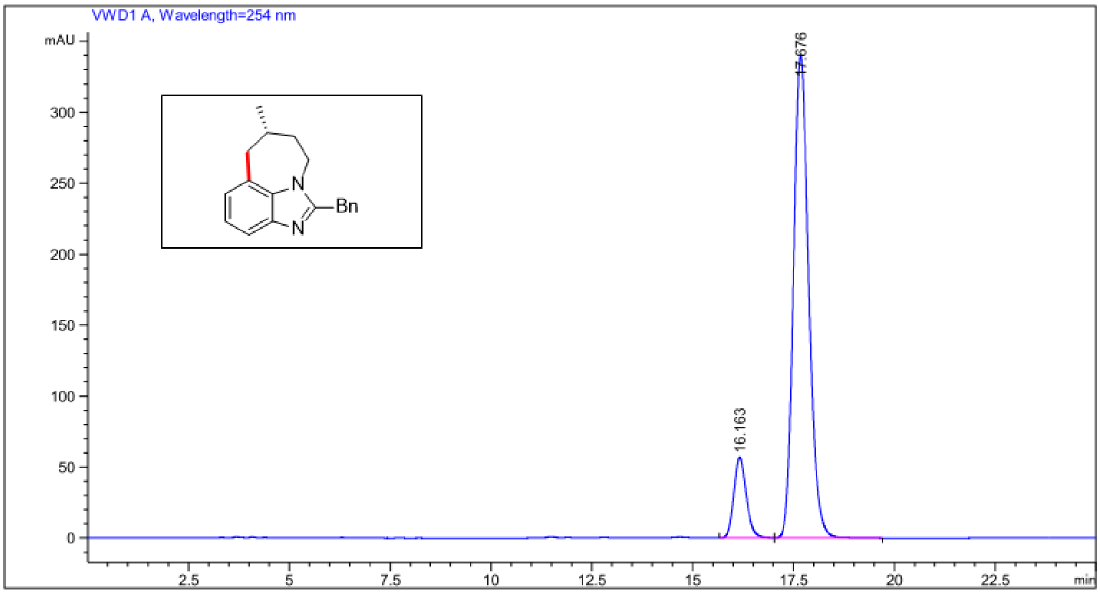

Peak #	RetTime [min]	Type	Width [min]	Area [mAU*s]	Height [mAU]	Area %
1	16.163	BB	0.3298	1222.40466	57.10241	12.0616
2	17.676	BB	0.4052	8912.29199	339.60861	87.9384

Supplementary Figure 161. HPLC spectra for racemic and chiral **3s**

Supplementary references

1 Chu, L.-L. & Qing, F.-L. Copper-Catalyzed Direct C–H Oxidative Trifluoromethylation of Heteroarenes. *J. Am. Chem. Soc.* **134**, 1298−1304 (2012).

2 Greenaway, R.-L., Campbell, C.-D., Holton, O.-T., Russell, C.-A. & Anderson, E.-A. Palladium-Catalyzed Cascade Cyclization of Ynamides to Azabicycles. *Chem. Eur. J.* **17**, 14366−14370 (2011).

3 Gan, P., Smith, M.-W., Braffman, N.-R. & Snyder, S.-A. Pyrone Diels-Alder Routes to Indolines and Hydroindolines: Syntheses of Gracilamine, Mesembrine, and Δ^7-Mesembrenone. *Angew. Chem. Int. Ed.* **55**, 3625−3630 (2016).

4 Brindle, C.-S., Yeung, C.-S. & Jacobsen, E.-N. Chiral β-iodoamines by urea-catalysed iodocyclization of trichloroacetimidates. *Chem. Sci.* **4**, 2100−2104 (2013).

5 Alonso, F., Rodríguez-Fernandez, M., Sánchez, D. & Yus, M. Synthesis of Perhydrofuro[2,3‐b]furans from Isopentenyl Alcohol through Carbonyl-Ene and Wacker-Type Reactions. *Eur. J. Org. Chem.* 6459−6469 (2011).

6 Diesel, J., Finogenova, A.-M. & Cramer, N. Nickel-Catalyzed Enantioselective Pyridone C–H Functionalizations Enabled by a Bulky N-Heterocyclic Carbene Ligand. *J. Am. Chem. Soc.* **140**, 4489−4493 (2018).

7 Hruszkewycz, D.-P., Miles, K.-C., Thiel, O.-R. & Stahl, S.-S. Co/NHPI-mediated aerobic oxygenation of benzylic C–H bonds in pharmaceutically relevant molecules. *Chem. Sci.* **8**, 1282−1287 (2017).

8 Fosu, S.-C., Hambira, C.-M., Chen, A.-D., Fuchs, J.-R. & Nagib, D.-A. Site-Selective C–H Functionalization of (Hetero)Arenes via Transient, Non-symmetric Iodanes *Chem*. **5**, 417−428 (2019).
